# Demographic predictors of trauma and depression in war-affected children from Poland and Ukraine: Implications for prevention of mental health problems

**DOI:** 10.1016/j.pmedr.2026.103422

**Published:** 2026-02-20

**Authors:** Maia Stanisławska-Kubiak, Dorota Wiśniewska-Szeplewicz, Mirosław Andrusiewicz, Halyna Katolyk, Monika Stanisławska, Roksana Malak, Bogusław Stelcer, Marta Czarnecka-Iwańczuk, Grażyna Teusz, Ewa Mojs

**Affiliations:** a Department of Clinical Psychology, Poznan University of Medical Sciences, Bukowska 70, 60-812 Poznań, Poland; b NeuroCare Medical Center, Rakoniewicka 23A, 60-111 Poznań, Poland; c Department of Cell Biology, Poznan University of Medical Sciences, Rokietnicka 5D, 60-806 Poznań, Poland; d Department of Theoretical Psychology, Institute of Management, Psychology and Safety, Lviv State University of Internal Affairs, Lviv, Ukraine; e Operational and Reconnaissance Department, Border Guard Academy, Marszałka Józefa Piłsudskiego 92, 75-531 Koszalin, Poland; f Department and Clinic of Rheumatology, Rehabilitation and Internal Medicine, Poznan University of Medical Sciences, 135/147 28 Czerwca 1956r., 61-545 Poznań, Poland; g Faculty of Educational Studies, Adam Mickiewicz University in Poznan, Szamarzewskiego 89, 60-568 Poznań, Poland

**Keywords:** Children, Adolescents, Trauma, Depression, War, Post-traumatic stress disorder (PTSD), Wartime displacement, Community-based proactive interventions

## Abstract

**Objective:**

The mental health consequences of war on children are well-documented, with early research emphasizing the critical role of caregiver presence in mitigating trauma. However, the ongoing conflict in Ukraine presents a novel context, requiring renewed investigation of the differential impact of direct war exposure and displacement on child and adolescent well-being.

**Methods:**

A cross-sectional study was conducted with 222 participants aged 7–21 years from Poland, Ukraine, and displaced Ukrainian populations (March 2024–March 2025, Poland/Ukraine). Depressive and trauma symptoms were assessed using standardized questionnaires.

**Results:**

Results showed that trauma symptoms linked to war witnessing varied by gender, country, and hobbies, with displaced children exhibiting the highest co-occurrence of depression and trauma. Engaging in hobbies was associated with fewer and weaker symptoms, suggesting a protective effect.

**Conclusions:**

This study underscores the urgent need for preventive mental health interventions targeting children affected by the war. Our findings suggest that displaced children are at particularly high risk for depression, highlighting the importance of early screening and intervention. Specifically, we propose implementing school-based programs in regions hosting refugees that incorporate hobby engagement, trauma-informed care training for teachers and caregivers, and family-based interventions to address cultural integration.

## Highlights

• War harms child mental health; Ukraine conflict and displacement effects need study.
• Displaced children had higher depression; displacement impacts mental health.
• Longer stays did not reduce symptoms; social and therapy support are important.
• Hobbies offered protection in trauma and depression relations.
• Tailored interventions are needed for war-affected children.

## 1 Introduction

The mental health consequences of war on children are well-documented, emphasizing the critical role of caregiver presence in mitigating trauma (Chudzicka-Czupała et al., 2025). However, the ongoing Russo-Ukrainian war presents a novel context, requiring renewed investigation of the differential impact of direct war exposure and displacement on child and adolescent well-being. Early intervention is vital to prevent long-term mental health issues and social problems. Interest in war's impact on children's mental health began during WWII, highlighting the protective role of caregivers (Freud and Burlingham, 1943). Separation from parents could cause severe psychological issues, with some children preferring danger over separation (Garmezy, 1983). The protective effect of the bond with caregivers is now regarded as one of the most critical findings in the literature concerning children in wartime situations.

Research on war's impact on children is a moral responsibility, as young lives may suffer long-lasting consequences from adult conflicts (Armitage, 2022). Modern psychology uses ethical, precise methods to understand children's responses to trauma without causing secondary harm. Early research shows trauma can have lasting, but not always permanent, effects, especially when loved ones are lost, and highlights the vital role of caregiver support (Garmezy, 1983; Garmezy and Rutter, 1985). Addressing the mental health needs of children affected by war is a critical aspect of preventive medicine, as early intervention can mitigate long-term risks for chronic mental and physical health conditions.

The Russo-Ukrainian war is Europe's first large-scale conflict in 80 years, making updated knowledge of its impact crucial. Armed conflict, migration, and loss of safety increase the risk of depression and PTSD in children/adolescents, leading to emotional dysregulation, identity and interpersonal issues, as well as disrupted functioning (Charlson et al., 2019). From the perspective of clinical psychology and psychotherapy, children who have had to change their place of residence due to armed conflict constitute one of the most vulnerable populations, with psychological needs that extend beyond standard crisis interventions. Childhood trauma exposure is associated with an increased risk of developing various forms of psychopathology in later life (McLaughlin and Lambert, 2017).

Studies of populations affected by war indicate variability in the level of psychological stress depending on the migration context; children who have not been resettled exhibit the lowest symptom severity, internally displaced children show higher levels, and refugee children experience the highest (Lushchak et al., 2024). The migration process, regardless of direct exposure to war trauma, can therefore be a significant risk factor for mental health (Ben-Ezra et al., 2023).

The high prevalence of PTSD and complex PTSD (CPTSD) among Ukrainian refugees remains associated with the intensity of war experiences and the degree of destabilization (Karstoft et al., 2024). Research highlights that stable housing, psychological care, education, and opportunities for social connection can mitigate the long-term effects of trauma (Marley and Mauki, 2019; Reed et al., 2012). Strong evidence supports the use of both individual and group therapy in reducing psychological harm (anxiety, depression, and PTSD symptoms) in children and adolescents with trauma (Wethington et al., 2008).

Poland responded to the refugee crisis by providing Ukrainian children and families with housing, educational, and psychological support. Public opinion surveys highlight strong solidarity and openness toward refugees (Karakiewicz-Krawczyk et al., 2022). Poland's refugee assistance includes direct support for families and children, initiatives to protect aid providers' mental health, a systemic approach to reducing secondary traumatization, and efforts to bolster community resilience (Zabłocka-Żytka and Lavdas, 2023).

This study identified key factors affecting mental health in children affected by the Russo-Ukrainian war, with implications for other conflict zones. While experiences vary, the underlying mechanisms are likely similar, helping inform prevention strategies worldwide that are adapted to local contexts.

## 2 Methods

### 2.1 Study design and population

Ukrainian and Polish children and adolescents (ages 7–21) were recruited via social media and academic networks for a cross-sectional study (March 2024–March 2025). The study included displaced Ukrainian youth and a Polish control group. Data collection was electronic, with questionnaires available in Polish or Ukrainian, depending on the respondents' native language. Inclusion criteria required participants to have Ukrainian or Polish nationality, to fall within the specified age range, to be able to independently complete the questionnaires, and to provide informed consent, either personally or through a guardian.

Respondents received information about the study's aims and procedures. The participation was entirely voluntary and anonymous. The collected data were handled in compliance with GDPR. The study received ethical approval from the Institutional Review Board (reference numbers 952/22 and 07/25; approval dates 12.01.2023 and 09.01.2025, respectively).

### 2.2 Measures

The Children's Depression Inventory 2 (CDI-2) was used to assess depressive symptoms (Wrocławska-Warchala et al., 2017). Symptoms of PTSD and complex PTSD were evaluated using the International Trauma Questionnaire – Child and Adolescent Version (ITQ-CA), aligned with ICD-11 diagnostic criteria (Cloitre et al., 2018). Strong psychometric properties characterize both instruments. The Polish versions of the instruments were based on officially validated adaptations developed by the Psychological Test Laboratory (PTP). Ukrainian-language versions of the CDI-2 and ITQ-CA were obtained from materials prepared by PTP for use with Ukrainian-speaking populations.

### 2.3 Statistical analyses

Statistical analyses included Mann-Whitney *U*, Kruskal-Wallis *H* (with Dunn's post hoc), Spearman's correlation, and Kendall's tau. Correlation strengths were categorized as very weak (|*R*| or |τ| < 0.1), weak (0.1–0.3), moderate (0.3–0.5), strong (0.5–0.7), and very strong (0.7–1). A large number of item-level correlation analyses across multiple subgroup stratifications (gender, country, displacement, war exposure, return status, hobbies) were hypothesis-generating exploratory analyses, not confirmatory findings. All statistical analyses were conducted using Statistica (version 13.5.0) (Dell Corp., Round Rock, TX, USA), with visualization in JMP Student Edition (version 18.0.2, 701896) (JMP Statistical Discovery LCC, Cary, NC, USA), and significance was set at *p* < 0.05.

## 3 Results

This study included 222 respondents aged 7–21 years (mean ± standard deviation: 15 ± 3.1 years). Due to high Cook's D distances (data points that disproportionately affected model parameters in the correlation analyses), four outliers were excluded. Finally, 155 females and 63 males from Ukraine, Poland, and displaced (*N* = 114, 81, and 23, respectively) participated in the study. CDI-2 and ITQ-CA scores did not differ between boys and girls (*p* > 0.05), indicating no gender differences.

### 3.1 Differences between Poland, Ukraine, and displaced children and war-witnessing

**Fig. 1:**
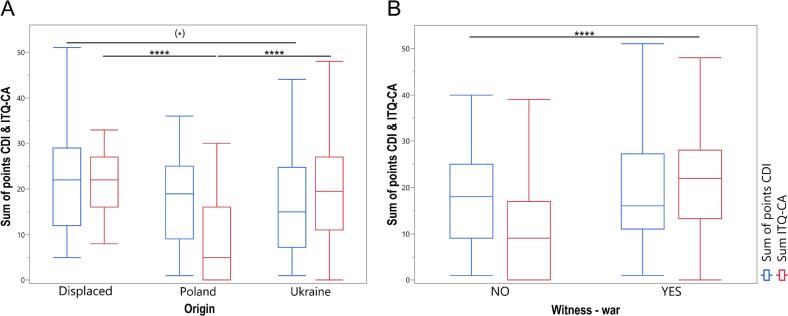
Boxplots showing the distribution of CDI-2 (Children's Depression Inventory 2) and ITQ-CA (International Trauma Questionnaire – Child and Adolescent Version) sum of scores across three origin groups of children and adolescents: Poland, Ukraine, and displaced (A), and by war-witnessing (B). ^(⁎)^*p* < 0.1, ^⁎⁎⁎⁎^*p* < 0.0001, March 2024–March 2025, Poland and Ukraine.

Displaced children showed higher CDI-2 scores compared to Polish and Ukrainian children (*p* = 0.0465), indicating greater depressive symptoms in this group (Fig. 1A).

Pairwise comparisons revealed no significant CDI-2 differences between Poland and Ukraine or Poland and displaced individuals (*p* > 0.05); Ukrainian and displaced individuals approached significance (*p* = 0.0597). ITQ-CA scores differed (*p* < 0.0001), with displaced individuals highest and Poles lowest; post hoc tests confirmed differences between Poland/Ukraine and Poland/displaced groups. War witnessing did not affect CDI-2 scores (*p* = 0.5930) but increased ITQ-CA scores (*p* < 0.0001) (Fig. 1B).

### 3.2 Residence duration and developmental or trauma-related scores

Residence duration showed no significant association with CDI-2 (*p* = 0.4967) or ITQ-CA scores (*p* = 0.1970).

**Fig. 2:**
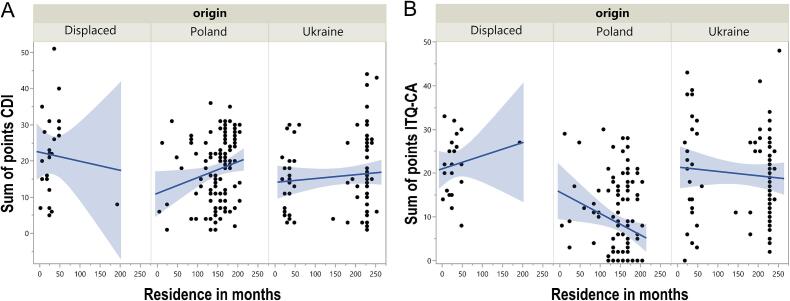
Scatterplots illustrating the relationship between residence duration (in months) and (A) CDI-2 (Children's Depression Inventory 2) scores, and (B) ITQ-CA (International Trauma Questionnaire – Child and Adolescent Version) scores across the three origin groups of children and adolescents: Poland, Ukraine, and Displaced, March 2024–March 2025, Poland and Ukraine.

In Poland, residence time weakly correlated with higher CDI-2 scores (*R* = 0.29, *p* = 0.0016) and lower ITQ-CA scores (*R* = -0.24, *p* = 0.0146; Fig. 2AB). No significant correlations were observed in Ukrainian or displaced children (*p* > 0.05).

For participants stratified by whether they witnessed war, among those who did not, the correlations between months of residence and CDI-2 and ITQ-CA scores were insignificant (*p* > 0.05). Similarly, for those who witnessed war, the correlations did not reach significance (*p* > 0.05). These results indicate no significant relationships between residence duration and either developmental or trauma-related scores, regardless of war witness status.

### 3.3 Correlations between single questions of CDI-2 and ITQ-CA

**Fig. 3:**
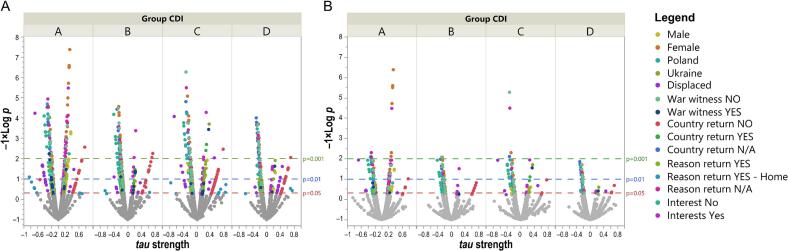
Correlations between single questions of CDI-2 (Children's Depression Inventory 2) and ITQ-CA (International Trauma Questionnaire – Child and Adolescent Version) in different groups of children and adolescents. Volcano plot of significant correlations between single questions of CDI-2 and ITQ-CA in the CDI-2 A, B, C, and D groups. The strength of *tau* correlation coefficient (x-axis) and the significance as the unadjusted *p*-values (panel A, shown as −1 × log *p*; y-axis) and Benjamini-Hochberg corrected *p*-values (panel B, shown as −1 × log *p*; y-axis). The dashed horizontal lines represent the *p* = 0.05 (red), *p* *=* 0.01 (blue), and *p* = 0.001 (green). Significant correlations are shown as colored spots, and non-significant correlations are grey, March 2024–March 2025, Poland and Ukraine. (For interpretation of the references to colour in this figure legend, the reader is referred to the web version of this article.)

Exploratory item-level correlation analyses revealed heterogeneous patterns of association between depressive and trauma-related symptoms across gender, country, migration status, war exposure, return status, and presence of hobbies (Fig. 3A). Due to the analytic density and subgroup sizes, particularly among displaced children, detailed item-by-item results are presented in the Supplementary Materials (Appendices S1–S6). At a conceptual level, these exploratory analyses suggested that: emotional domains (e.g., sadness, crying, mood) were more strongly linked to trauma symptoms in girls than boys; cognitive and somatic domains (e.g., concentration, sleep, eating) showed variable associations depending on migration and war exposure status; relational domains (e.g., family importance, peer relationships) differed across cultural and situational contexts.

After correction for multiple testing, the number of significant correlations was substantially reduced across all subgroups, indicating that many uncorrected associations should be interpreted cautiously (Fig. 3B; Appendices S1–S6). These findings highlight the complex and context-dependent nature of the relationship between depressive and trauma-related symptoms and should be considered hypothesis-generating rather than confirmatory.

The relationships between depression and trauma in children vary by gender, culture, displacement, war exposure, return, hobbies, and subgroup traits. Before correction, many significant links were identified, but after correction, some became insignificant, highlighting their complex, context-dependent nature.

## 4 Discussion

Our findings reveal a surprising yet clinically significant dichotomy between the levels of depressive symptoms (measured by CDI-2) and PTSD/CPTSD symptoms (measured by ITQ-CA), depending on the refugees' migration status. This pattern suggests that displacement may be associated with increased affective burden beyond trauma-related symptomatology, rather than trauma exposure alone accounting for depressive outcomes. This underscores the need for targeted interventions to address the unique stressors associated with displacement. It highlights the potential of multi-component interventions that combine mental health services with practical support for resettlement and integration. For example, programs that provide assistance with housing, employment, and education, while also offering culturally sensitive mental health counseling and peer support groups, may be particularly effective in reducing depression among displaced children. Additionally, it is important to address the social and economic inequalities that displaced children often face, as these can contribute to feelings of hopelessness and despair (Andrade et al., 2023). Rather than implying a direct causal mechanism, these results point to displacement-related stressors, such as uncertainty, social disruption, and loss of familiar routines, as contextual factors that may coincide with elevated depressive symptoms. This underscores the importance of interventions that address both trauma-related symptoms and broader psychosocial stressors experienced during displacement.

Previous literature has described partially distinct neuropsychological correlates of PTSD and depression (Kozlowska et al., 2015; Liu et al., 2017). Displacement worsens neurobiological changes due to chronic uncertainty, social disruption, and loss of safety, along with limited access to support systems like family, education, and culture. While such models provide useful background, the present study does not allow direct inferences about underlying neurobiological mechanisms. Therefore, references to neuropsychological processes should be understood as a theoretical context, rather than explanatory findings derived from the current data.

Children in war zones may show short-term adaptive responses that may limit anhedonia and depression, whereas displaced children, facing helplessness, loss of control, and overload during transition, are at higher risk of depression and apathy; adolescents and young adults are particularly vulnerable to these effects (Cairns, 1994). Beyond limited educational, professional, and psychological support opportunities, they experience not only post-traumatic stress symptoms but also anxiety and worry, significant indicators of their fear (Frounfelker et al., 2019; Borkovec, 1994).

Furthermore, our data verify previous findings that depression in children and adolescents may be more susceptible to environmental influences, such as migration, social isolation, and lack of integration, whereas PTSD symptoms tend to remain closely linked to the character of the traumatic event itself (Karstoft et al., 2024; Reed et al., 2012). This indicates the necessity of tailoring trauma-focused therapies for PTSD. In contrast, treatment for depression should incorporate identity-related, integrative, and systemic aspects to address the broader psychosocial context. Children remaining in active conflict zones may display short-term adaptive responses that temporarily buffer depressive symptoms, whereas displaced children may encounter prolonged stress related to uncertainty, acculturation, and perceived loss of control. These interpretations remain speculative and should be tested in longitudinal designs.

We assessed the effects of direct trauma exposure. Consistent with prior research, ITQ-CA scores indicated that direct exposure to armed conflict was associated with higher trauma-related symptoms, including re-experiencing, avoidance, and hyperarousal (Trickey et al., 2012). In contrast, war exposure was not directly associated with increased depressive symptoms, suggesting that depression may be more closely related to cumulative or chronic stressors rather than immediate traumatic events.

This distinction should be interpreted cautiously, as pre-existing mental health conditions, prior trauma exposure, parental mental health, and socioeconomic factors were not assessed and may confound observed associations. The absence of data on potential confounders, such as baseline psychological functioning, family mental health, or socioeconomic stability, limits causal interpretation and may partially explain group differences observed in depressive symptom severity.

We also examined how the duration of stay, together with war exposure, relates to depressive and trauma symptoms. Longer stays in safe environments did not reduce depression or PTSD/CPTSD severity, suggesting time alone may not relieve distress; displaced children showed the highest co-occurrence of depressive and trauma symptoms, marking them as a priority for proactive screening and tailored prevention addressing acculturation, isolation, and social-support loss.

In Poland, longer residence is linked to increased depression and decreased trauma symptoms, suggesting a shift to chronic depression. Ukrainian children show no symptom change over time, possibly due to neuropsychological factors like emotional blunting or limbic hyperactivation. This highlights the need to customize mental health support, taking demographics into account, and strengthening community-based support.

No significant links were found between residence duration and depression or trauma symptoms, regardless of war exposure. Symptoms remained stable over time, even months after displacement.

The link between depression and trauma varies by gender, war experience, migration, return, and interests. Children not witnessing war showed clearer mood-trauma links, indicating better processing, while war-exposed children had fewer connections, possibly due to emotional detachment or dissociation (Brom, 2014; Hadar et al., 2020). Although displaced children exhibited higher co-occurrence of depressive and trauma symptoms, this subgroup was relatively small. Therefore, findings should be interpreted as descriptive and associative rather than indicative of stable or generalizable mechanisms.

Gender differences suggest girls internalize trauma, processing it emotionally and relationally, while boys often show external or less obvious symptoms, aligning with existing research (Søegaard et al., 2021). Identification of gender-specific perception and expression of trauma is an important factor in the treatment of children who have been trauma-exposed and are presenting post-trauma exposure disturbances (Craig and Sprang, 2014).

We next considered how cultural and situational differences shape the manifestation and co-occurrence of depressive and trauma-related symptoms. Children with both high depression and PTSD/CPTSD symptoms face compounded psychological pain. Depression affects mood, self-esteem, and feelings of guilt and helplessness, while PTSD/CPTSD involves fear, intrusive memories, irritability, emotional numbness, and avoidance. When these overlap, they create a complex, ongoing suffering that impacts their thinking, emotions, and social life beyond just trauma or mood issues.

In Polish children, trauma is linked to thoughts about pain, mood, and family, affecting guilt. Ukrainian children show somatic signs like eating problems and crying. Displaced children experience sleep, learning, and school attitude issues due to ongoing stress, impairing concentration and motivation. Interventions should address both PTSD and depression, with protective factors like family, education, and hobbies helping moderate symptoms. Challenges such as limited resources, cultural barriers, and a shortage of trained professionals hinder the delivery of psychological interventions. Solutions include training refugee health workers, enhancing cultural competency, and collaborating with local agencies to improve healthcare access (Johnson et al., 2009). Hobbies buffer emotional symptoms. In Polish children, lack correlates with more severe symptoms; in Ukrainian children, they aid expression; and in displaced children, they help maintain routines and function. Across all groups, hobbies support emotion regulation and control. Our findings regarding the role of hobbies as a protective factor have significant implications for the design of preventive interventions. There is evidence that engaging in hobbies has a positive impact on children's well-being (Cleary et al., 2025; Steinberg and Simon, 2019). Across groups, engagement in hobbies was associated with differences in symptom presentation. In the present study, hobbies were assessed as a self-reported presence or absence of regular activities, rather than as a quantified or experimentally manipulated factor.

It is therefore possible that children who reported having hobbies already differed in baseline functioning, resilience, family support, or socioeconomic conditions, which may confound the observed associations between hobbies and mental health outcomes.

Rather than functioning as established protective factors, hobbies may serve as indicators of preserved routines, social engagement, or environmental stability. These associations should not be interpreted causally. These findings suggest that school and community programs, like hobby clubs, arts, crafts, and sports, can boost resilience and reduce war-related mental health issues. These activities should be culturally relevant and accessible, offering social, creative, and skill-building opportunities.

These results highlight the need for personalized intervention strategies considering gender, exposure history, culture, reintegration experiences, and individual developmental resources. Therapies should target symptom mitigation, facilitate emotional expression, support the storytelling of experiences, and strengthen self-regulation mechanisms.

There are limitations in this study. In addition to the relatively small size of the displaced subgroup and the cross-sectional design, the lack of control for pre-existing mental health conditions, parental mental health, socioeconomic status, and prior trauma exposure limits the internal validity of the findings. Standardized self-report measures may also be subject to cultural biases in interpretation. Using child/adolescent instruments with participants aged 18+ may compromise measurement validity and age appropriateness, potentially affecting the accuracy and generalizability of findings for adult respondents. Despite these limitations, the study provides initial evidence highlighting heterogeneity in mental health outcomes among war-affected children.

## 5 Conclusions

Trauma-related and depressive symptoms appear to represent overlapping but distinct dimensions of psychological distress in war-affected children. While trauma symptoms are closely associated with war exposure, depressive symptoms appear more strongly associated with displacement-related and contextual stressors. Displaced children may therefore represent a particularly vulnerable group requiring early screening and tailored support. Associations with hobbies and social engagement highlight potential avenues for supportive interventions, although causal inferences cannot be drawn. Future longitudinal studies are needed to inform the development of evidence-based policies and interventions. Exposure to safety alone may be insufficient to reduce psychological distress in war-affected children. Mental health assessments should differentiate between trauma-related and depressive symptom profiles and consider contextual stressors such as migration and social disruption. Activities and hobbies may serve as accessible components of supportive care, but should be integrated cautiously and evaluated within comprehensive, culturally sensitive intervention frameworks.

## Ethical information

The study was conducted in accordance with the guidelines of the Declaration of Helsinki. Approval was granted by the Ethics Committee of the Poznan University of Medical Sciences Institutional Review Board (IRB) (reference numbers: 952/22 and 07/25, dates of approval 12.01.2023 and 09.01.2025, respectively). Informed consent was obtained from all participants or their parents/legal guardians/next of kin (when underage) to participate in the study.

## Clinical trial registry

Not applicable.

## CRediT authorship contribution statement

**Maia Stanisławska-Kubiak:** Writing – review & editing, Writing – original draft, Validation, Methodology, Investigation, Formal analysis, Data curation, Conceptualization. **Dorota Wiśniewska-Szeplewicz:** Writing – review & editing, Validation, Methodology, Investigation, Formal analysis, Data curation, Conceptualization. **Mirosław Andrusiewicz:** Writing – review & editing, Writing – original draft, Visualization, Validation, Methodology, Formal analysis, Data curation. **Halyna Katolyk:** Writing – review & editing, Validation, Investigation, Data curation. **Monika Stanisławska:** Writing – review & editing, Validation, Methodology, Formal analysis. **Roksana Malak:** Writing – review & editing, Validation, Data curation. **Bogusław Stelcer:** Investigation, Data curation. **Marta Czarnecka-Iwańczuk:** Investigation, Data curation. **Grażyna Teusz:** Writing – review & editing, Validation, Methodology, Data curation. **Ewa Mojs:** Writing – review & editing, Validation, Supervision, Resources, Project administration, Funding acquisition, Conceptualization.

## Declaration of generative AI and AI-assisted technologies in the writing process

During the preparation of this work, the authors used Grammarly and ChatGPT in order to improve the language. After using this tool, the authors reviewed and edited the content as needed and take full responsibility for the publication's content.

## Funding

This research did not receive any specific grant from funding agencies in the public, commercial, or not-for-profit sectors. This study was supported by internal funding from the Department of Clinical Psychology, 10.13039/501100010438Poznan University of Medical Sciences.

## Declaration of competing interest

The authors declare that they have no known competing financial interests or personal relationships that could have appeared to influence the work reported in this paper.

## Data Availability

Data will be made available on request.

## Appendix A Supplementary data

Supplementary material 1 Image 1

Supplementary material 2 Image 2

Supplementary material 3 Image 3

Supplementary material 4 Image 4

Supplementary material 5 Image 5

Supplementary material 6 Image 6
