# Supplementary material for: Demographic predictors of trauma and depression in war-affected children from Poland and Ukraine: Implications for prevention of mental health problems
Source: Prev Med Rep. 2026 Feb 20;63:103422. doi: 10.1016/j.pmedr.2026.103422 (PMC12945576; doi:10.1016/j.pmedr.2026.103422)
Supplement: Supplementary file 1 — Supplementary material 1 [file mmc1.docx]

**CDI (panels A-D) and ITQ-CA correlations in gender dependent groups**

**CDI2 – group A**

Among the girls, 36 significant correlations in CDI2–group A were observed (Figure S1), most significant correlations between CDI2-1 “Sadness” and all 12 ITQ-CA categories (all positive). The remaining 24 correlations were negative and very weak or weak in strength (Table S1). The strongest correlations of moderate, positive strength were between CDI2-1 “Sadness” and ITQ-CA categories: “Avoiding thoughts”, “Nervousness”, “Calming difficulty”, “Emotional numbness”, “Social difficulty”. After the Benjamini-Hochberg correction, 21 significant correlations remained. In the boys, 37 correlations were significant and similarly to girls, most of them were between CDI2-1 (10 correlations), and CDI2-9 “Tendency to cry” and “CDI2-10 “Bad mood” (6 significant correlations) followed by CDI2-15 “Sleep quality” and CDI2-17 “Eating attitude” (5 and 4 significant correlations, respectively”). Still, among all significant correlations, only three were negative (between CDI2-18 “Pain thoughts” and both ITQ-CA “Reliving events in mind” and “Avoiding thoughts”, and CDI2-27 “Eating problems” and ITQ-CA “Reliving events in mind”). After the Benjamini-Hochberg correction, eight significant correlations remained in the group of boys (moderate to weak strength, all positive).

**CDI2 – group B**

In the CDI2 – group B among the females participants 31 significant correlations (Figure S1) were observed, most significant correlations between CDI2-2 “Hopeful”, CDI2-13 “Self-perception”, CDI2-24 “Love awareness” and ITQ-CA “Sense of failure” (all negative and weak), Additionally (also weak and negatively) CDI-13 was correlated with ITQ-CA “Nervousness” Emotional numbness”. Still, all observed correlations were negative, weak, or very weak (Table S1). After the Benjamini-Hochberg correction, 13 significant correlations remained. In the group of boys, only six correlations were significant in the CDI-B, all weak or very weak. Three were negative and three were positive. None of them remains significant after Benjamini-Hochberg correction.

**CDI2 – group C**

In the CDI2 – group C among the females participants 21 significant correlations (Figure S1) were observed, most significant correlations between CDI2-4 “Enjoyment”, and ITQ-CA “Nervousness”, “Emotional mumbling” and “Sence of failure”, and additionally between CDI2-20 “School fun” and ITQ-CA “Overlay cautions, Nervousness”, Calming difficulty” and “Sense of failure” (all negative and weak or very weak). The remaining significant correlations were also very weak negative (Table S1). After the Benjamini-Hochberg correction, seven significant correlations remained. In the males, we observed 11 significant correlations in the CDI2-C (6 negative and five positive). Most of them were between CDI2-28 “Memorization” and ITQ-CA “Bad dreams”, “Reliving events in mind”, “Avoiding thoughts”, and “Nervousness” (all negative and weak or very weak). Only 3 of them, of moderate strength, remain significant after Benjamini-Hochberg correction – “Memorization” with “Reliving events in mind” (negative), “Self-comparison to others” with “Social difficulty, and “Dealing with school tasks” with “Avoiding physically” (both positive).

**CDI2 – group D**

Among the girls, 20 significant correlations in CDI2–group D (Figure S1) were observed, most significant correlations between CDI2-5 “Importance for family” and 8 ITQ-CA categories (all negative), and CDI2-19 “Loneliness feeling” and 5 ITQ-CA categories. Still, all correlations, in the mentioned but remaining categories, were weak or very weak and negative. After Benjamini-Hochberg correction, five significant correlations remain (CDI2-5 “Importance for family” and both “Emotional numbness” and “Sense of failure”, CDI2-11 “Company” and CDI2-19 “Loneliness feeling” with ITQ-CA “Sense of failure”, and lastly CDI2-25 Peer arguing with “Emotional numbness”). In the boys group we observed five positive correlations (between CDI2-19 “Loneliness feeling” with ITQ-CA “Bad dreams”, “Calming difficulty”, “Emotional numbness”, “Sense of failure”, and “Social difficulty”), and three negative (CDI2-21 “Friends” with “Relieving events in mind”, and CDI2-25 “Peer arguing” with both “Relieving events in mind” and Nervousness”). None of them remains significant after Benjamini-Hochberg correction (Table S1).


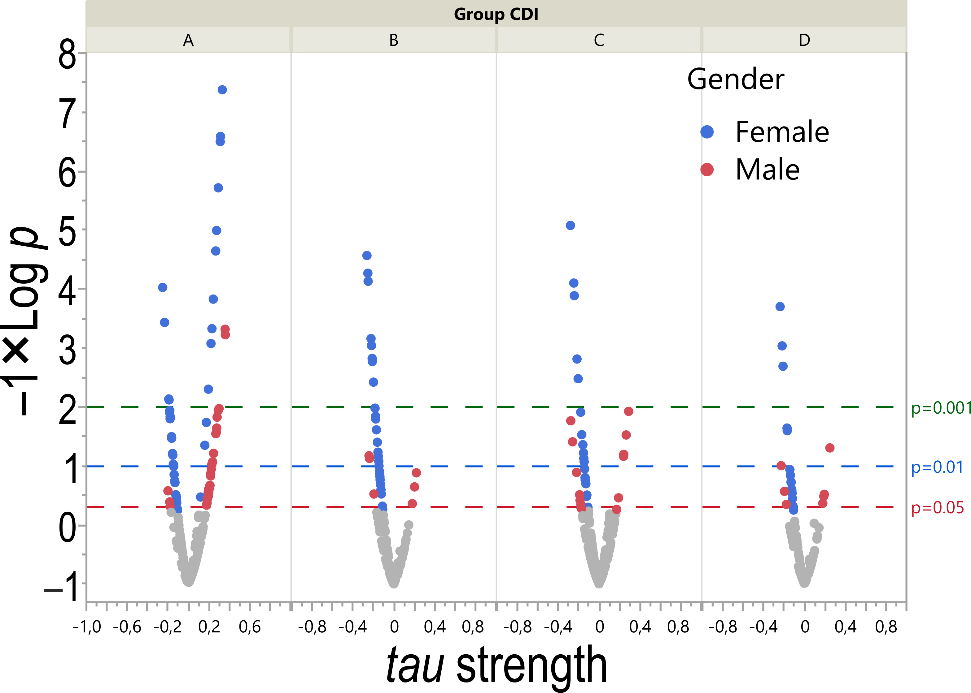


**Figure S1.** Correlations between single questions of CDI-2 (Children's Depression Inventory 2) and ITQ-CA (International Trauma Questionnaire – Child and Adolescent Version) in gender dependent groups of children and adolescents from Poland and Ukraine. Volcano plot of significant correlations between single questions of CDI and ITQ-CA in the CDI A, B, C, and D groups. The strength of *tau* correlation coefficient (x-axis) and the significance as the unadjusted *p*-values (shown as −1×log *p*; y-axis). The dashed horizontal lines represent the *p* = 0.05 (red), *p* = 0.01 (blue), and *p* = 0.001 (green). Significant correlations are shown as colored spots, and non-significant correlations are grey, March 2024-March 2025, Poland and Ukraine.

**Table S1**. Children's Depression Inventory 2 (CDI-2) (panels A-D) and International Trauma Questionnaire – Child and Adolescent Version (ITQ-CA) correlations in gender dependent groups children and adolescents from Poland and Ukraine, March 2024-March 2025, Poland and Ukraine.

| **CDI2** | **ITQ-CA** | **Group CDI** | **N** | ***Tau*** | ***p*-value** | ***p*-value ^BH^** | **N** | ***Tau*** | ***p*-value** | ***p*-value ^BH^** |
| --- | --- | --- | --- | --- | --- | --- | --- | --- | --- | --- |
|  |  | **Females** | | | | | **Males** | | | |
| 1 Sadness | 1 Bad dreams | A | 144 | 0.18 | 0.0018 | 0.0326 | 58 | 0.29 | 0.0011 | 0.0245 |
|  | 2 Reliving events in mind | A | 143 | 0.22 | 0.0001 | 0.0047 | 58 | 0.04 | 0.6393 | 0.8362 |
|  | 3 Avoiding thoughts | A | 142 | 0.32 | <0.0001 | <0.0001 | 57 | 0.28 | 0.0023 | 0.0377 |
|  | 4 Avoiding physically | A | 141 | 0.23 | <0.0001 | 0.0033 | 57 | 0.23 | 0.0114 | 0.1046 |
|  | 5 Overly cautious | A | 142 | 0.16 | 0.0044 | 0.0567 | 56 | 0.30 | 0.0011 | 0.0235 |
|  | 6 Nervousness | A | 143 | 0.29 | <0.0001 | <0.0001 | 58 | 0.14 | 0.1340 | 0.3889 |
|  | 7 Calming difficulty | A | 142 | 0.33 | <0.0001 | <0.0001 | 57 | 0.20 | 0.0287 | 0.1726 |
|  | 8 Emotional numbness | A | 143 | 0.31 | <0.0001 | <0.0001 | 56 | 0.21 | 0.0212 | 0.1473 |
|  | 9 Sense of failure | A | 142 | 0.27 | <0.0001 | 0.0008 | 58 | 0.24 | 0.0085 | 0.0865 |
|  | 10 Self-doubt | A | 143 | 0.24 | <0.0001 | 0.0018 | 57 | 0.27 | 0.0027 | 0.0420 |
|  | 11 Disconnection to others | A | 143 | 0.20 | 0.0005 | 0.0143 | 57 | 0.19 | 0.0325 | 0.1821 |
|  | 12 Social difficulty | A | 142 | 0.28 | <0.0001 | 0.0005 | 58 | 0.36 | 0.0001 | 0.0037 |
| 2 Hopeful | 1 Bad dreams | B | 148 | -0.09 | 0.0919 | 0.3238 | 60 | -0.01 | 0.8996 | 0.9604 |
|  | 2 Reliving events in mind | B | 147 | -0.07 | 0.1784 | 0.4456 | 60 | -0.24 | 0.0074 | 0.0799 |
|  | 3 Avoiding thoughts | B | 146 | -0.06 | 0.2884 | 0.5635 | 58 | -0.06 | 0.5125 | 0.7538 |
|  | 4 Avoiding physically | B | 145 | -0.03 | 0.5751 | 0.7955 | 59 | 0.20 | 0.0225 | 0.1534 |
|  | 5 Overly cautious | B | 146 | -0.16 | 0.0039 | 0.0525 | 58 | -0.03 | 0.7738 | 0.9057 |
|  | 6 Nervousness | B | 147 | -0.18 | 0.0014 | 0.0283 | 60 | -0.14 | 0.1098 | 0.3549 |
|  | 7 Calming difficulty | B | 146 | -0.10 | 0.0677 | 0.2734 | 59 | 0.07 | 0.4588 | 0.7171 |
|  | 8 Emotional numbness | B | 146 | -0.14 | 0.0123 | 0.1083 | 58 | 0.18 | 0.0434 | 0.2146 |
|  | 9 Sense of failure | B | 146 | -0.22 | 0.0001 | 0.0050 | 60 | 0.03 | 0.7044 | 0.8751 |
|  | 10 Self-doubt | B | 147 | -0.15 | 0.0083 | 0.0853 | 59 | 0.03 | 0.7128 | 0.8793 |
|  | 11 Disconnection to others | B | 147 | -0.12 | 0.0297 | 0.1754 | 59 | 0.01 | 0.9218 | 0.9716 |
|  | 12 Social difficulty | B | 146 | -0.08 | 0.1303 | 0.3832 | 60 | 0.12 | 0.1751 | 0.4408 |
| 3 Self-confidence | 1 Bad dreams | C | 148 | 0.08 | 0.1608 | 0.4240 | 60 | -0.07 | 0.4389 | 0.6995 |
|  | 2 Reliving events in mind | C | 147 | 0.01 | 0.9043 | 0.9635 | 60 | -0.16 | 0.0693 | 0.2766 |
|  | 3 Avoiding thoughts | C | 146 | 0.05 | 0.3398 | 0.6155 | 58 | -0.06 | 0.5311 | 0.7684 |
|  | 4 Avoiding physically | C | 145 | 0.04 | 0.4277 | 0.6911 | 59 | 0.00 | 0.9654 | 0.9914 |
|  | 5 Overly cautious | C | 146 | -0.04 | 0.4593 | 0.7174 | 58 | -0.08 | 0.3683 | 0.6400 |
|  | 6 Nervousness | C | 147 | -0.08 | 0.1367 | 0.3921 | 60 | -0.12 | 0.1625 | 0.4258 |
|  | 7 Calming difficulty | C | 146 | 0.05 | 0.3593 | 0.6332 | 59 | -0.05 | 0.5453 | 0.7767 |
|  | 8 Emotional numbness | C | 146 | -0.03 | 0.6397 | 0.8362 | 58 | 0.03 | 0.7502 | 0.8934 |
|  | 9 Sense of failure | C | 146 | -0.04 | 0.4933 | 0.7423 | 60 | -0.09 | 0.2842 | 0.5586 |
|  | 10 Self-doubt | C | 147 | -0.05 | 0.3616 | 0.6348 | 59 | -0.12 | 0.1744 | 0.4408 |
|  | 11 Disconnection to others | C | 147 | -0.02 | 0.7202 | 0.8824 | 59 | -0.04 | 0.6731 | 0.8553 |
|  | 12 Social difficulty | C | 146 | 0.00 | 0.9359 | 0.9758 | 60 | 0.11 | 0.1971 | 0.4676 |
| 4 Enjoyment | 1 Bad dreams | C | 148 | -0.08 | 0.1423 | 0.3989 | 59 | -0.03 | 0.7032 | 0.8747 |
|  | 2 Reliving events in mind | C | 147 | -0.12 | 0.0317 | 0.1793 | 59 | -0.07 | 0.4222 | 0.6866 |
|  | 3 Avoiding thoughts | C | 146 | -0.04 | 0.4729 | 0.7293 | 57 | 0.05 | 0.5483 | 0.7772 |
|  | 4 Avoiding physically | C | 145 | 0.02 | 0.7843 | 0.9121 | 58 | -0.01 | 0.9382 | 0.9762 |
|  | 5 Overly cautious | C | 146 | -0.10 | 0.0654 | 0.2679 | 57 | -0.18 | 0.0508 | 0.2326 |
|  | 6 Nervousness | C | 147 | -0.25 | <0.0001 | 0.0012 | 59 | -0.14 | 0.1272 | 0.3800 |
|  | 7 Calming difficulty | C | 146 | -0.16 | 0.0043 | 0.0561 | 58 | -0.02 | 0.8077 | 0.9228 |
|  | 8 Emotional numbness | C | 146 | -0.24 | <0.0001 | 0.0017 | 57 | 0.00 | 0.9875 | 1.0000 |
|  | 9 Sense of failure | C | 146 | -0.18 | 0.0012 | 0.0254 | 59 | -0.03 | 0.7626 | 0.8998 |
|  | 10 Self-doubt | C | 147 | -0.10 | 0.0607 | 0.2560 | 58 | -0.14 | 0.1164 | 0.3656 |
|  | 11 Disconnection to others | C | 147 | -0.11 | 0.0580 | 0.2499 | 58 | 0.00 | 0.9870 | 1.0000 |
|  | 12 Social difficulty | C | 146 | -0.14 | 0.0112 | 0.1039 | 59 | -0.05 | 0.5464 | 0.7767 |
| 5 Importance for family | 1 Bad dreams | D | 147 | -0.12 | 0.0289 | 0.1728 | 60 | 0.01 | 0.9549 | 0.9861 |
|  | 2 Reliving events in mind | D | 146 | -0.13 | 0.0189 | 0.1375 | 60 | -0.12 | 0.1870 | 0.4553 |
|  | 3 Avoiding thoughts | D | 145 | -0.06 | 0.2933 | 0.5686 | 58 | -0.03 | 0.7398 | 0.8895 |
|  | 4 Avoiding physically | D | 144 | -0.07 | 0.2005 | 0.4717 | 59 | 0.05 | 0.6056 | 0.8180 |
|  | 5 Overly cautious | D | 145 | -0.09 | 0.1106 | 0.3559 | 58 | -0.14 | 0.1316 | 0.3849 |
|  | 6 Nervousness | D | 146 | -0.13 | 0.0184 | 0.1356 | 60 | 0.01 | 0.8757 | 0.9480 |
|  | 7 Calming difficulty | D | 145 | -0.08 | 0.1802 | 0.4472 | 59 | 0.08 | 0.3972 | 0.6654 |
|  | 8 Emotional numbness | D | 145 | -0.22 | 0.0001 | 0.0050 | 58 | 0.02 | 0.8200 | 0.9290 |
|  | 9 Sense of failure | D | 145 | -0.17 | 0.0023 | 0.0377 | 60 | 0.04 | 0.6213 | 0.8285 |
|  | 10 Self-doubt | D | 146 | -0.14 | 0.0113 | 0.1040 | 59 | 0.05 | 0.5863 | 0.8044 |
|  | 11 Disconnection to others | D | 146 | -0.11 | 0.0498 | 0.2293 | 59 | 0.10 | 0.2730 | 0.5496 |
|  | 12 Social difficulty | D | 145 | -0.12 | 0.0357 | 0.1906 | 60 | 0.13 | 0.1506 | 0.4086 |
| 6 Self-acceptance | 1 Bad dreams | B | 148 | -0.02 | 0.7713 | 0.9044 | 59 | 0.01 | 0.9136 | 0.9670 |
|  | 2 Reliving events in mind | B | 147 | -0.01 | 0.9070 | 0.9652 | 59 | -0.19 | 0.0297 | 0.1755 |
|  | 3 Avoiding thoughts | B | 146 | -0.01 | 0.8966 | 0.9594 | 57 | 0.00 | 0.9562 | 0.9871 |
|  | 4 Avoiding physically | B | 145 | -0.02 | 0.7103 | 0.8779 | 58 | -0.02 | 0.8234 | 0.9290 |
|  | 5 Overly cautious | B | 146 | 0.00 | 0.9348 | 0.9757 | 57 | -0.11 | 0.2174 | 0.4904 |
|  | 6 Nervousness | B | 147 | -0.13 | 0.0209 | 0.1456 | 59 | -0.14 | 0.1301 | 0.3832 |
|  | 7 Calming difficulty | B | 146 | -0.10 | 0.0842 | 0.3076 | 58 | -0.06 | 0.5179 | 0.7583 |
|  | 8 Emotional numbness | B | 146 | -0.07 | 0.1914 | 0.4608 | 57 | 0.00 | 0.9914 | 1.0000 |
|  | 9 Sense of failure | B | 146 | -0.15 | 0.0069 | 0.0757 | 59 | -0.03 | 0.7568 | 0.8969 |
|  | 10 Self-doubt | B | 147 | -0.06 | 0.3160 | 0.5923 | 58 | 0.09 | 0.3022 | 0.5799 |
|  | 11 Disconnection to others | B | 147 | -0.06 | 0.2925 | 0.5679 | 58 | 0.00 | 0.9911 | 1.0000 |
|  | 12 Social difficulty | B | 146 | -0.06 | 0.3143 | 0.5907 | 59 | 0.13 | 0.1351 | 0.3900 |
| 7 Blame | 1 Bad dreams | B | 147 | 0.01 | 0.9127 | 0.9669 | 59 | -0.12 | 0.1861 | 0.4544 |
|  | 2 Reliving events in mind | B | 146 | 0.00 | 0.9625 | 0.9902 | 59 | -0.24 | 0.0067 | 0.0744 |
|  | 3 Avoiding thoughts | B | 145 | 0.00 | 0.9473 | 0.9824 | 57 | -0.06 | 0.4857 | 0.7382 |
|  | 4 Avoiding physically | B | 144 | -0.04 | 0.4637 | 0.7211 | 58 | 0.08 | 0.3655 | 0.6381 |
|  | 5 Overly cautious | B | 145 | -0.02 | 0.7397 | 0.8895 | 57 | -0.03 | 0.7017 | 0.8737 |
|  | 6 Nervousness | B | 146 | -0.11 | 0.0548 | 0.2406 | 59 | -0.16 | 0.0729 | 0.2855 |
|  | 7 Calming difficulty | B | 145 | -0.09 | 0.1188 | 0.3686 | 58 | -0.03 | 0.7163 | 0.8809 |
|  | 8 Emotional numbness | B | 145 | -0.09 | 0.1261 | 0.3784 | 57 | 0.02 | 0.8458 | 0.9343 |
|  | 9 Sense of failure | B | 145 | -0.18 | 0.0010 | 0.0233 | 59 | 0.01 | 0.9121 | 0.9667 |
|  | 10 Self-doubt | B | 146 | -0.11 | 0.0483 | 0.2250 | 58 | -0.03 | 0.7421 | 0.8906 |
|  | 11 Disconnection to others | B | 146 | -0.13 | 0.0203 | 0.1426 | 58 | -0.11 | 0.2276 | 0.5022 |
|  | 12 Social difficulty | B | 145 | -0.13 | 0.0252 | 0.1621 | 59 | -0.03 | 0.7552 | 0.8964 |
| 8 Suicide | 1 Bad dreams | B | 146 | 0.03 | 0.5667 | 0.7893 | 59 | 0.04 | 0.6587 | 0.8474 |
|  | 2 Reliving events in mind | B | 145 | -0.04 | 0.5032 | 0.7466 | 59 | -0.15 | 0.0925 | 0.3244 |
|  | 3 Avoiding thoughts | B | 144 | 0.04 | 0.4346 | 0.6972 | 57 | 0.07 | 0.4453 | 0.7052 |
|  | 4 Avoiding physically | B | 143 | 0.02 | 0.7765 | 0.9075 | 58 | -0.01 | 0.8760 | 0.9480 |
|  | 5 Overly cautious | B | 144 | 0.00 | 0.9850 | 1.0000 | 57 | -0.04 | 0.6507 | 0.8418 |
|  | 6 Nervousness | B | 145 | -0.03 | 0.6233 | 0.8295 | 59 | -0.11 | 0.2080 | 0.4817 |
|  | 7 Calming difficulty | B | 144 | 0.00 | 0.9675 | 0.9926 | 58 | -0.04 | 0.6371 | 0.8355 |
|  | 8 Emotional numbness | B | 144 | -0.05 | 0.3506 | 0.6253 | 57 | 0.04 | 0.6256 | 0.8318 |
|  | 9 Sense of failure | B | 144 | -0.14 | 0.0100 | 0.0964 | 59 | 0.05 | 0.5745 | 0.7951 |
|  | 10 Self-doubt | B | 145 | -0.09 | 0.1272 | 0.3800 | 58 | 0.05 | 0.6063 | 0.8181 |
|  | 11 Disconnection to others | B | 145 | 0.00 | 0.9495 | 0.9833 | 58 | 0.06 | 0.4956 | 0.7436 |
|  | 12 Social difficulty | B | 144 | 0.06 | 0.2770 | 0.5526 | 59 | 0.22 | 0.0130 | 0.1108 |
| 9 Tendency to cry | 1 Bad dreams | A | 146 | -0.04 | 0.5034 | 0.7466 | 60 | 0.14 | 0.1101 | 0.3553 |
|  | 2 Reliving events in mind | A | 145 | -0.09 | 0.1257 | 0.3777 | 60 | -0.14 | 0.1091 | 0.3537 |
|  | 3 Avoiding thoughts | A | 144 | -0.02 | 0.7562 | 0.8969 | 58 | 0.06 | 0.5347 | 0.7703 |
|  | 4 Avoiding physically | A | 143 | -0.04 | 0.5323 | 0.7689 | 59 | 0.14 | 0.1239 | 0.3743 |
|  | 5 Overly cautious | A | 144 | -0.10 | 0.0882 | 0.3164 | 58 | 0.19 | 0.0369 | 0.1945 |
|  | 6 Nervousness | A | 145 | -0.18 | 0.0014 | 0.0285 | 60 | -0.01 | 0.8741 | 0.9478 |
|  | 7 Calming difficulty | A | 144 | -0.10 | 0.0740 | 0.2883 | 59 | 0.14 | 0.1220 | 0.3723 |
|  | 8 Emotional numbness | A | 144 | -0.07 | 0.2188 | 0.4920 | 58 | 0.20 | 0.0265 | 0.1643 |
|  | 9 Sense of failure | A | 144 | -0.19 | 0.0008 | 0.0192 | 60 | 0.23 | 0.0093 | 0.0918 |
|  | 10 Self-doubt | A | 145 | -0.12 | 0.0324 | 0.1818 | 59 | 0.18 | 0.0447 | 0.2171 |
|  | 11 Disconnection to others | A | 145 | -0.07 | 0.2284 | 0.5034 | 59 | 0.18 | 0.0459 | 0.2197 |
|  | 12 Social difficulty | A | 144 | -0.06 | 0.2902 | 0.5648 | 60 | 0.36 | <0.0001 | 0.0033 |
| 10 Bad mood | 1 Bad dreams | A | 148 | 0.03 | 0.5477 | 0.7772 | 60 | 0.16 | 0.0781 | 0.2957 |
|  | 2 Reliving events in mind | A | 147 | -0.02 | 0.7388 | 0.8895 | 60 | -0.12 | 0.1848 | 0.4535 |
|  | 3 Avoiding thoughts | A | 146 | 0.03 | 0.6016 | 0.8158 | 58 | 0.18 | 0.0425 | 0.2122 |
|  | 4 Avoiding physically | A | 145 | 0.01 | 0.8259 | 0.9291 | 59 | 0.19 | 0.0373 | 0.1953 |
|  | 5 Overly cautious | A | 146 | -0.02 | 0.6891 | 0.8645 | 58 | 0.16 | 0.0683 | 0.2748 |
|  | 6 Nervousness | A | 147 | -0.14 | 0.0097 | 0.0943 | 60 | -0.02 | 0.7968 | 0.9189 |
|  | 7 Calming difficulty | A | 146 | -0.07 | 0.1794 | 0.4465 | 59 | 0.22 | 0.0147 | 0.1197 |
|  | 8 Emotional numbness | A | 146 | -0.07 | 0.2208 | 0.4938 | 58 | 0.27 | 0.0028 | 0.0428 |
|  | 9 Sense of failure | A | 146 | -0.16 | 0.0034 | 0.0478 | 60 | 0.19 | 0.0365 | 0.1938 |
|  | 10 Self-doubt | A | 147 | -0.06 | 0.3001 | 0.5778 | 59 | 0.14 | 0.1061 | 0.3496 |
|  | 11 Disconnection to others | A | 147 | -0.05 | 0.3632 | 0.6360 | 59 | 0.13 | 0.1392 | 0.3957 |
|  | 12 Social difficulty | A | 146 | -0.01 | 0.8435 | 0.9336 | 60 | 0.28 | 0.0015 | 0.0288 |
| 11 Company | 1 Bad dreams | D | 147 | 0.01 | 0.8349 | 0.9318 | 60 | 0.05 | 0.5913 | 0.8075 |
|  | 2 Reliving events in mind | D | 146 | -0.01 | 0.8406 | 0.9329 | 60 | -0.01 | 0.9041 | 0.9635 |
|  | 3 Avoiding thoughts | D | 145 | 0.02 | 0.7634 | 0.9000 | 58 | 0.10 | 0.2702 | 0.5473 |
|  | 4 Avoiding physically | D | 144 | 0.02 | 0.6690 | 0.8531 | 59 | 0.12 | 0.1725 | 0.4398 |
|  | 5 Overly cautious | D | 145 | -0.09 | 0.1120 | 0.3585 | 58 | 0.09 | 0.3258 | 0.6025 |
|  | 6 Nervousness | D | 146 | -0.12 | 0.0260 | 0.1643 | 60 | 0.12 | 0.1646 | 0.4293 |
|  | 7 Calming difficulty | D | 145 | -0.08 | 0.1655 | 0.4302 | 59 | 0.10 | 0.2794 | 0.5549 |
|  | 8 Emotional numbness | D | 145 | -0.05 | 0.3854 | 0.6552 | 58 | 0.09 | 0.3015 | 0.5794 |
|  | 9 Sense of failure | D | 145 | -0.24 | <0.0001 | 0.0020 | 60 | -0.02 | 0.8216 | 0.9290 |
|  | 10 Self-doubt | D | 146 | -0.11 | 0.0556 | 0.2436 | 59 | -0.03 | 0.7508 | 0.8935 |
|  | 11 Disconnection to others | D | 146 | -0.05 | 0.3859 | 0.6557 | 59 | 0.04 | 0.6314 | 0.8336 |
|  | 12 Social difficulty | D | 145 | -0.03 | 0.5597 | 0.7853 | 60 | 0.04 | 0.6175 | 0.8257 |
| 12 Self-determination | 1 Bad dreams | C | 147 | -0.06 | 0.2717 | 0.5489 | 60 | -0.10 | 0.2686 | 0.5470 |
|  | 2 Reliving events in mind | C | 146 | -0.07 | 0.2226 | 0.4961 | 60 | -0.11 | 0.2294 | 0.5050 |
|  | 3 Avoiding thoughts | C | 145 | -0.02 | 0.6926 | 0.8667 | 58 | -0.26 | 0.0039 | 0.0520 |
|  | 4 Avoiding physically | C | 144 | -0.01 | 0.8323 | 0.9307 | 59 | -0.07 | 0.4079 | 0.6722 |
|  | 5 Overly cautious | C | 145 | -0.08 | 0.1776 | 0.4440 | 58 | -0.05 | 0.6171 | 0.8257 |
|  | 6 Nervousness | C | 146 | -0.15 | 0.0060 | 0.0703 | 60 | -0.04 | 0.6456 | 0.8395 |
|  | 7 Calming difficulty | C | 145 | -0.08 | 0.1477 | 0.4058 | 59 | -0.03 | 0.7675 | 0.9031 |
|  | 8 Emotional numbness | C | 145 | -0.07 | 0.2315 | 0.5073 | 58 | -0.09 | 0.3261 | 0.6027 |
|  | 9 Sense of failure | C | 145 | -0.08 | 0.1709 | 0.4373 | 60 | -0.06 | 0.4968 | 0.7437 |
|  | 10 Self-doubt | C | 146 | -0.15 | 0.0084 | 0.0863 | 59 | -0.03 | 0.7562 | 0.8969 |
|  | 11 Disconnection to others | C | 146 | -0.03 | 0.5325 | 0.7689 | 59 | 0.11 | 0.2204 | 0.4938 |
|  | 12 Social difficulty | C | 145 | -0.06 | 0.2957 | 0.5716 | 60 | 0.04 | 0.6288 | 0.8325 |
| 13 Self-perception | 1 Bad dreams | B | 147 | -0.18 | 0.0015 | 0.0297 | 60 | -0.06 | 0.4825 | 0.7368 |
|  | 2 Reliving events in mind | B | 146 | -0.13 | 0.0170 | 0.1302 | 60 | -0.17 | 0.0608 | 0.2561 |
|  | 3 Avoiding thoughts | B | 145 | -0.15 | 0.0058 | 0.0687 | 58 | -0.06 | 0.4946 | 0.7432 |
|  | 4 Avoiding physically | B | 144 | -0.14 | 0.0133 | 0.1115 | 59 | 0.03 | 0.7048 | 0.8754 |
|  | 5 Overly cautious | B | 145 | -0.17 | 0.0024 | 0.0392 | 58 | -0.08 | 0.3902 | 0.6589 |
|  | 6 Nervousness | B | 146 | -0.25 | <0.0001 | 0.0011 | 60 | -0.10 | 0.2699 | 0.5472 |
|  | 7 Calming difficulty | B | 145 | -0.21 | 0.0002 | 0.0071 | 59 | -0.02 | 0.8193 | 0.9286 |
|  | 8 Emotional numbness | B | 145 | -0.26 | <0.0001 | 0.0008 | 58 | 0.04 | 0.6928 | 0.8667 |
|  | 9 Sense of failure | B | 145 | -0.25 | <0.0001 | 0.0012 | 60 | -0.07 | 0.4302 | 0.6928 |
|  | 10 Self-doubt | B | 146 | -0.21 | 0.0001 | 0.0066 | 59 | 0.04 | 0.6698 | 0.8533 |
|  | 11 Disconnection to others | B | 146 | -0.14 | 0.0129 | 0.1103 | 59 | -0.01 | 0.9348 | 0.9757 |
|  | 12 Social difficulty | B | 145 | -0.14 | 0.0138 | 0.1147 | 60 | 0.08 | 0.3434 | 0.6186 |
| 14 Learning attitude | 1 Bad dreams | C | 147 | 0.08 | 0.1472 | 0.4051 | 60 | 0.04 | 0.6781 | 0.8586 |
|  | 2 Reliving events in mind | C | 146 | 0.09 | 0.0935 | 0.3254 | 60 | -0.14 | 0.1172 | 0.3662 |
|  | 3 Avoiding thoughts | C | 145 | 0.10 | 0.0648 | 0.2664 | 58 | -0.02 | 0.8316 | 0.9305 |
|  | 4 Avoiding physically | C | 144 | 0.00 | 0.9666 | 0.9923 | 59 | 0.13 | 0.1425 | 0.3991 |
|  | 5 Overly cautious | C | 145 | 0.01 | 0.9098 | 0.9661 | 58 | 0.15 | 0.0925 | 0.3244 |
|  | 6 Nervousness | C | 146 | -0.09 | 0.0925 | 0.3244 | 60 | -0.04 | 0.6629 | 0.8509 |
|  | 7 Calming difficulty | C | 145 | 0.00 | 0.9858 | 1.0000 | 59 | 0.08 | 0.3587 | 0.6326 |
|  | 8 Emotional numbness | C | 145 | -0.07 | 0.2419 | 0.5195 | 58 | 0.10 | 0.2695 | 0.5470 |
|  | 9 Sense of failure | C | 145 | -0.06 | 0.2567 | 0.5346 | 60 | 0.24 | 0.0063 | 0.0722 |
|  | 10 Self-doubt | C | 146 | -0.13 | 0.0174 | 0.1311 | 59 | 0.09 | 0.3391 | 0.6148 |
|  | 11 Disconnection to others | C | 146 | -0.04 | 0.4743 | 0.7307 | 59 | 0.11 | 0.1987 | 0.4698 |
|  | 12 Social difficulty | C | 145 | -0.05 | 0.3545 | 0.6286 | 60 | 0.15 | 0.0853 | 0.3103 |
| 15 Sleep quality | 1 Bad dreams | A | 147 | 0.02 | 0.7824 | 0.9110 | 60 | 0.22 | 0.0134 | 0.1120 |
|  | 2 Reliving events in mind | A | 146 | 0.10 | 0.0746 | 0.2893 | 60 | 0.06 | 0.5070 | 0.7497 |
|  | 3 Avoiding thoughts | A | 145 | 0.07 | 0.2050 | 0.4789 | 58 | 0.07 | 0.4581 | 0.7166 |
|  | 4 Avoiding physically | A | 144 | -0.03 | 0.5464 | 0.7767 | 59 | 0.13 | 0.1488 | 0.4061 |
|  | 5 Overly cautious | A | 145 | -0.03 | 0.5872 | 0.8047 | 58 | 0.25 | 0.0061 | 0.0707 |
|  | 6 Nervousness | A | 146 | -0.16 | 0.0032 | 0.0457 | 60 | 0.13 | 0.1301 | 0.3832 |
|  | 7 Calming difficulty | A | 145 | -0.08 | 0.1658 | 0.4306 | 59 | 0.11 | 0.2206 | 0.4938 |
|  | 8 Emotional numbness | A | 145 | 0.03 | 0.6297 | 0.8331 | 58 | 0.09 | 0.3162 | 0.5923 |
|  | 9 Sense of failure | A | 145 | -0.15 | 0.0065 | 0.0737 | 60 | 0.20 | 0.0273 | 0.1670 |
|  | 10 Self-doubt | A | 146 | -0.04 | 0.4540 | 0.7131 | 59 | 0.13 | 0.1365 | 0.3921 |
|  | 11 Disconnection to others | A | 146 | -0.12 | 0.0339 | 0.1858 | 59 | 0.19 | 0.0347 | 0.1870 |
|  | 12 Social difficulty | A | 145 | -0.02 | 0.7002 | 0.8723 | 60 | 0.19 | 0.0281 | 0.1702 |
| 16 Tiredness level | 1 Bad dreams | A | 145 | 0.00 | 0.9387 | 0.9764 | 60 | 0.11 | 0.2266 | 0.5004 |
|  | 2 Reliving events in mind | A | 144 | -0.04 | 0.4983 | 0.7449 | 60 | -0.03 | 0.7360 | 0.8887 |
|  | 3 Avoiding thoughts | A | 143 | 0.04 | 0.5014 | 0.7458 | 58 | 0.05 | 0.5921 | 0.8078 |
|  | 4 Avoiding physically | A | 142 | -0.01 | 0.8669 | 0.9443 | 59 | 0.05 | 0.6135 | 0.8238 |
|  | 5 Overly cautious | A | 143 | -0.08 | 0.1739 | 0.4408 | 58 | 0.07 | 0.4281 | 0.6915 |
|  | 6 Nervousness | A | 144 | -0.12 | 0.0369 | 0.1945 | 60 | 0.04 | 0.6676 | 0.8526 |
|  | 7 Calming difficulty | A | 143 | -0.05 | 0.3668 | 0.6392 | 59 | 0.02 | 0.8107 | 0.9242 |
|  | 8 Emotional numbness | A | 143 | -0.07 | 0.2470 | 0.5257 | 58 | 0.13 | 0.1592 | 0.4218 |
|  | 9 Sense of failure | A | 143 | -0.11 | 0.0526 | 0.2374 | 60 | 0.10 | 0.2471 | 0.5257 |
|  | 10 Self-doubt | A | 144 | -0.11 | 0.0564 | 0.2458 | 59 | -0.03 | 0.7207 | 0.8824 |
|  | 11 Disconnection to others | A | 144 | -0.08 | 0.1452 | 0.4035 | 59 | 0.09 | 0.3034 | 0.5804 |
|  | 12 Social difficulty | A | 143 | -0.07 | 0.2005 | 0.4717 | 60 | 0.20 | 0.0247 | 0.1602 |
| 17 Eating attitude | 1 Bad dreams | A | 147 | -0.01 | 0.8363 | 0.9327 | 58 | 0.13 | 0.1422 | 0.3988 |
|  | 2 Reliving events in mind | A | 146 | -0.03 | 0.5659 | 0.7888 | 58 | -0.06 | 0.5180 | 0.7583 |
|  | 3 Avoiding thoughts | A | 145 | -0.03 | 0.6360 | 0.8355 | 56 | 0.12 | 0.2025 | 0.4754 |
|  | 4 Avoiding physically | A | 144 | -0.01 | 0.8939 | 0.9579 | 57 | 0.18 | 0.0459 | 0.2197 |
|  | 5 Overly cautious | A | 145 | -0.05 | 0.4032 | 0.6689 | 56 | 0.21 | 0.0230 | 0.1553 |
|  | 6 Nervousness | A | 146 | -0.14 | 0.0101 | 0.0972 | 58 | 0.07 | 0.4129 | 0.6770 |
|  | 7 Calming difficulty | A | 145 | -0.13 | 0.0191 | 0.1375 | 57 | 0.21 | 0.0223 | 0.1526 |
|  | 8 Emotional numbness | A | 145 | -0.07 | 0.2132 | 0.4868 | 56 | 0.08 | 0.3697 | 0.6413 |
|  | 9 Sense of failure | A | 145 | -0.18 | 0.0011 | 0.0247 | 58 | 0.04 | 0.6808 | 0.8599 |
|  | 10 Self-doubt | A | 146 | -0.04 | 0.5198 | 0.7599 | 57 | 0.03 | 0.7626 | 0.8998 |
|  | 11 Disconnection to others | A | 146 | -0.07 | 0.1926 | 0.4624 | 57 | 0.14 | 0.1221 | 0.3723 |
|  | 12 Social difficulty | A | 145 | -0.04 | 0.5303 | 0.7684 | 58 | 0.23 | 0.0095 | 0.0930 |
| 18 Pain thoughts | 1 Bad dreams | A | 143 | -0.23 | <0.0001 | 0.0029 | 59 | -0.12 | 0.1947 | 0.4646 |
|  | 2 Reliving events in mind | A | 142 | -0.18 | 0.0016 | 0.0299 | 59 | -0.18 | 0.0404 | 0.2062 |
|  | 3 Avoiding thoughts | A | 141 | -0.19 | 0.0007 | 0.0186 | 57 | -0.18 | 0.0483 | 0.2250 |
|  | 4 Avoiding physically | A | 140 | -0.12 | 0.0306 | 0.1773 | 58 | 0.04 | 0.6921 | 0.8666 |
|  | 5 Overly cautious | A | 141 | -0.25 | <0.0001 | 0.0013 | 57 | 0.02 | 0.8598 | 0.9402 |
|  | 6 Nervousness | A | 142 | -0.16 | 0.0061 | 0.0710 | 59 | -0.10 | 0.2462 | 0.5247 |
|  | 7 Calming difficulty | A | 141 | -0.09 | 0.1201 | 0.3698 | 58 | -0.02 | 0.8585 | 0.9402 |
|  | 8 Emotional numbness | A | 141 | -0.15 | 0.0093 | 0.0918 | 57 | 0.13 | 0.1607 | 0.4240 |
|  | 9 Sense of failure | A | 141 | -0.14 | 0.0139 | 0.1154 | 59 | 0.06 | 0.5031 | 0.7466 |
|  | 10 Self-doubt | A | 143 | -0.13 | 0.0178 | 0.1323 | 58 | -0.17 | 0.0607 | 0.2560 |
|  | 11 Disconnection to others | A | 142 | -0.09 | 0.1013 | 0.3406 | 58 | 0.05 | 0.5451 | 0.7767 |
|  | 12 Social difficulty | A | 141 | -0.18 | 0.0012 | 0.0255 | 59 | 0.06 | 0.5299 | 0.7684 |
| 19 Loneliness feeling | 1 Bad dreams | D | 143 | -0.06 | 0.2988 | 0.5758 | 60 | 0.18 | 0.0427 | 0.2123 |
|  | 2 Reliving events in mind | D | 142 | -0.06 | 0.3195 | 0.5955 | 60 | -0.01 | 0.9256 | 0.9720 |
|  | 3 Avoiding thoughts | D | 141 | -0.01 | 0.8750 | 0.9478 | 58 | 0.06 | 0.4753 | 0.7315 |
|  | 4 Avoiding physically | D | 140 | 0.01 | 0.7985 | 0.9192 | 59 | 0.06 | 0.5309 | 0.7684 |
|  | 5 Overly cautious | D | 141 | -0.09 | 0.1085 | 0.3530 | 58 | 0.07 | 0.4355 | 0.6975 |
|  | 6 Nervousness | D | 142 | -0.14 | 0.0114 | 0.1046 | 60 | 0.08 | 0.3869 | 0.6557 |
|  | 7 Calming difficulty | D | 141 | -0.10 | 0.0931 | 0.3254 | 59 | 0.19 | 0.0301 | 0.1765 |
|  | 8 Emotional numbness | D | 141 | -0.13 | 0.0263 | 0.1643 | 58 | 0.18 | 0.0422 | 0.2113 |
|  | 9 Sense of failure | D | 142 | -0.21 | 0.0002 | 0.0080 | 60 | 0.19 | 0.0326 | 0.1822 |
|  | 10 Self-doubt | D | 142 | -0.12 | 0.0275 | 0.1675 | 59 | 0.01 | 0.8858 | 0.9533 |
|  | 11 Disconnection to others | D | 142 | -0.11 | 0.0480 | 0.2250 | 59 | 0.14 | 0.1109 | 0.3564 |
|  | 12 Social difficulty | D | 141 | -0.10 | 0.0909 | 0.3226 | 60 | 0.25 | 0.0049 | 0.0607 |
| 20 School fun | 1 Bad dreams | C | 141 | -0.05 | 0.4099 | 0.6739 | 59 | 0.10 | 0.2423 | 0.5195 |
|  | 2 Reliving events in mind | C | 140 | -0.05 | 0.3798 | 0.6510 | 59 | -0.18 | 0.0451 | 0.2183 |
|  | 3 Avoiding thoughts | C | 139 | -0.04 | 0.5224 | 0.7614 | 57 | -0.13 | 0.1570 | 0.4180 |
|  | 4 Avoiding physically | C | 138 | -0.04 | 0.4766 | 0.7326 | 58 | 0.02 | 0.8141 | 0.9264 |
|  | 5 Overly cautious | C | 139 | -0.17 | 0.0029 | 0.0437 | 57 | -0.12 | 0.1735 | 0.4408 |
|  | 6 Nervousness | C | 140 | -0.28 | <0.0001 | 0.0005 | 59 | -0.07 | 0.4524 | 0.7112 |
|  | 7 Calming difficulty | C | 139 | -0.21 | 0.0003 | 0.0108 | 58 | 0.10 | 0.2549 | 0.5324 |
|  | 8 Emotional numbness | C | 139 | -0.14 | 0.0172 | 0.1306 | 57 | 0.02 | 0.8109 | 0.9242 |
|  | 9 Sense of failure | C | 139 | -0.22 | 0.0002 | 0.0066 | 59 | -0.05 | 0.5903 | 0.8070 |
|  | 10 Self-doubt | C | 140 | -0.14 | 0.0158 | 0.1251 | 58 | -0.10 | 0.2759 | 0.5511 |
|  | 11 Disconnection to others | C | 140 | -0.14 | 0.0156 | 0.1241 | 58 | 0.06 | 0.4999 | 0.7458 |
|  | 12 Social difficulty | C | 139 | -0.12 | 0.0326 | 0.1822 | 59 | -0.05 | 0.5711 | 0.7921 |
| 21 Friends | 1 Bad dreams | D | 144 | 0.05 | 0.3777 | 0.6491 | 60 | 0.04 | 0.6428 | 0.8387 |
|  | 2 Reliving events in mind | D | 143 | -0.06 | 0.3235 | 0.6001 | 60 | -0.18 | 0.0445 | 0.2166 |
|  | 3 Avoiding thoughts | D | 142 | 0.10 | 0.0932 | 0.3254 | 58 | -0.05 | 0.5768 | 0.7969 |
|  | 4 Avoiding physically | D | 141 | 0.05 | 0.4150 | 0.6793 | 59 | 0.07 | 0.4573 | 0.7163 |
|  | 5 Overly cautious | D | 142 | -0.07 | 0.1958 | 0.4653 | 58 | -0.04 | 0.6314 | 0.8336 |
|  | 6 Nervousness | D | 143 | -0.14 | 0.0143 | 0.1172 | 60 | -0.03 | 0.7032 | 0.8747 |
|  | 7 Calming difficulty | D | 142 | -0.07 | 0.2355 | 0.5123 | 59 | -0.06 | 0.5028 | 0.7466 |
|  | 8 Emotional numbness | D | 142 | -0.07 | 0.2441 | 0.5219 | 58 | 0.04 | 0.6792 | 0.8593 |
|  | 9 Sense of failure | D | 142 | -0.13 | 0.0272 | 0.1669 | 60 | 0.04 | 0.6528 | 0.8429 |
|  | 10 Self-doubt | D | 143 | -0.10 | 0.0892 | 0.3184 | 59 | 0.00 | 0.9669 | 0.9925 |
|  | 11 Disconnection to others | D | 143 | -0.07 | 0.2238 | 0.4974 | 59 | 0.09 | 0.3065 | 0.5827 |
|  | 12 Social difficulty | D | 142 | -0.04 | 0.5064 | 0.7497 | 60 | 0.13 | 0.1515 | 0.4095 |
| 22 Dealing with school tasks | 1 Bad dreams | C | 140 | 0.06 | 0.3141 | 0.5906 | 60 | 0.08 | 0.3867 | 0.6557 |
|  | 2 Reliving events in mind | C | 139 | 0.06 | 0.3135 | 0.5903 | 60 | -0.04 | 0.6880 | 0.8639 |
|  | 3 Avoiding thoughts | C | 138 | 0.10 | 0.0753 | 0.2903 | 58 | 0.14 | 0.1178 | 0.3671 |
|  | 4 Avoiding physically | C | 137 | 0.09 | 0.1227 | 0.3733 | 59 | 0.29 | 0.0012 | 0.0251 |
|  | 5 Overly cautious | C | 138 | -0.03 | 0.6245 | 0.8306 | 58 | 0.09 | 0.3247 | 0.6019 |
|  | 6 Nervousness | C | 139 | -0.02 | 0.7401 | 0.8895 | 60 | -0.03 | 0.7236 | 0.8834 |
|  | 7 Calming difficulty | C | 138 | 0.06 | 0.2629 | 0.5412 | 59 | 0.08 | 0.3880 | 0.6572 |
|  | 8 Emotional numbness | C | 138 | 0.04 | 0.4651 | 0.7227 | 58 | 0.19 | 0.0345 | 0.1870 |
|  | 9 Sense of failure | C | 138 | -0.02 | 0.7207 | 0.8824 | 60 | 0.16 | 0.0644 | 0.2652 |
|  | 10 Self-doubt | C | 139 | 0.00 | 0.9459 | 0.9817 | 59 | 0.00 | 0.9677 | 0.9926 |
|  | 11 Disconnection to others | C | 139 | -0.01 | 0.8217 | 0.9290 | 59 | 0.17 | 0.0540 | 0.2394 |
|  | 12 Social difficulty | C | 138 | -0.02 | 0.7691 | 0.9034 | 60 | 0.24 | 0.0068 | 0.0755 |
| 23 Self-comparison to others | 1 Bad dreams | C | 147 | -0.05 | 0.3436 | 0.6186 | 59 | 0.05 | 0.5462 | 0.7767 |
|  | 2 Reliving events in mind | C | 146 | -0.07 | 0.1834 | 0.4515 | 59 | -0.13 | 0.1371 | 0.3923 |
|  | 3 Avoiding thoughts | C | 145 | 0.01 | 0.8317 | 0.9305 | 57 | -0.05 | 0.5565 | 0.7819 |
|  | 4 Avoiding physically | C | 144 | -0.04 | 0.5020 | 0.7460 | 58 | 0.01 | 0.8731 | 0.9471 |
|  | 5 Overly cautious | C | 145 | -0.10 | 0.0835 | 0.3057 | 57 | 0.09 | 0.3165 | 0.5923 |
|  | 6 Nervousness | C | 146 | -0.13 | 0.0199 | 0.1404 | 59 | -0.04 | 0.6220 | 0.8288 |
|  | 7 Calming difficulty | C | 145 | -0.08 | 0.1635 | 0.4272 | 58 | 0.04 | 0.6485 | 0.8407 |
|  | 8 Emotional numbness | C | 145 | -0.10 | 0.0861 | 0.3116 | 57 | -0.01 | 0.8790 | 0.9495 |
|  | 9 Sense of failure | C | 145 | -0.15 | 0.0074 | 0.0799 | 59 | 0.04 | 0.6523 | 0.8429 |
|  | 10 Self-doubt | C | 146 | -0.11 | 0.0499 | 0.2293 | 58 | -0.06 | 0.4931 | 0.7423 |
|  | 11 Disconnection to others | C | 146 | -0.08 | 0.1603 | 0.4238 | 58 | 0.08 | 0.3729 | 0.6445 |
|  | 12 Social difficulty | C | 145 | -0.07 | 0.2237 | 0.4974 | 59 | 0.27 | 0.0030 | 0.0439 |
| 24 Love-awareness | 1 Bad dreams | B | 147 | -0.07 | 0.1985 | 0.4696 | 60 | 0.02 | 0.8564 | 0.9402 |
|  | 2 Reliving events in mind | B | 146 | -0.06 | 0.2521 | 0.5314 | 60 | -0.07 | 0.4286 | 0.6917 |
|  | 3 Avoiding thoughts | B | 145 | -0.06 | 0.3034 | 0.5804 | 58 | -0.08 | 0.3749 | 0.6463 |
|  | 4 Avoiding physically | B | 144 | -0.05 | 0.3455 | 0.6205 | 59 | 0.15 | 0.0996 | 0.3377 |
|  | 5 Overly cautious | B | 145 | -0.11 | 0.0478 | 0.2250 | 58 | 0.11 | 0.2398 | 0.5169 |
|  | 6 Nervousness | B | 146 | -0.20 | 0.0004 | 0.0117 | 60 | 0.05 | 0.5782 | 0.7981 |
|  | 7 Calming difficulty | B | 145 | -0.13 | 0.0171 | 0.1302 | 59 | 0.13 | 0.1367 | 0.3921 |
|  | 8 Emotional numbness | B | 145 | -0.18 | 0.0016 | 0.0299 | 58 | 0.10 | 0.2663 | 0.5463 |
|  | 9 Sense of failure | B | 145 | -0.22 | 0.0001 | 0.0042 | 60 | 0.08 | 0.3496 | 0.6240 |
|  | 10 Self-doubt | B | 146 | -0.14 | 0.0151 | 0.1219 | 59 | -0.04 | 0.6801 | 0.8596 |
|  | 11 Disconnection to others | B | 146 | -0.07 | 0.1903 | 0.4598 | 59 | 0.06 | 0.4801 | 0.7357 |
|  | 12 Social difficulty | B | 145 | -0.11 | 0.0535 | 0.2377 | 60 | 0.11 | 0.1948 | 0.4646 |
| 25 Peer arguing | 1 Bad dreams | D | 147 | -0.04 | 0.4748 | 0.7311 | 60 | -0.13 | 0.1508 | 0.4089 |
|  | 2 Reliving events in mind | D | 146 | -0.08 | 0.1748 | 0.4408 | 60 | -0.23 | 0.0098 | 0.0955 |
|  | 3 Avoiding thoughts | D | 145 | -0.01 | 0.8621 | 0.9411 | 58 | -0.03 | 0.7400 | 0.8895 |
|  | 4 Avoiding physically | D | 144 | -0.05 | 0.3941 | 0.6621 | 59 | 0.01 | 0.9247 | 0.9720 |
|  | 5 Overly cautious | D | 145 | -0.08 | 0.1516 | 0.4097 | 58 | -0.15 | 0.1050 | 0.3476 |
|  | 6 Nervousness | D | 146 | -0.13 | 0.0246 | 0.1602 | 60 | -0.20 | 0.0268 | 0.1655 |
|  | 7 Calming difficulty | D | 145 | -0.05 | 0.3536 | 0.6281 | 59 | -0.01 | 0.9079 | 0.9652 |
|  | 8 Emotional numbness | D | 145 | -0.17 | 0.0025 | 0.0401 | 58 | -0.01 | 0.9238 | 0.9720 |
|  | 9 Sense of failure | D | 145 | -0.10 | 0.0860 | 0.3116 | 60 | -0.13 | 0.1552 | 0.4149 |
|  | 10 Self-doubt | D | 146 | -0.11 | 0.0488 | 0.2265 | 59 | -0.11 | 0.2312 | 0.5069 |
|  | 11 Disconnection to others | D | 146 | -0.01 | 0.8175 | 0.9281 | 59 | -0.12 | 0.1875 | 0.4560 |
|  | 12 Social difficulty | D | 145 | -0.05 | 0.3493 | 0.6237 | 60 | 0.06 | 0.4637 | 0.7211 |
| 26 Napping/dozing | 1 Bad dreams | A | 147 | -0.01 | 0.8513 | 0.9373 | 60 | 0.12 | 0.1597 | 0.4226 |
|  | 2 Reliving events in mind | A | 146 | -0.02 | 0.7456 | 0.8920 | 60 | 0.01 | 0.9246 | 0.9720 |
|  | 3 Avoiding thoughts | A | 145 | 0.01 | 0.7994 | 0.9192 | 58 | -0.02 | 0.8019 | 0.9199 |
|  | 4 Avoiding physically | A | 144 | 0.12 | 0.0335 | 0.1848 | 59 | -0.01 | 0.9067 | 0.9652 |
|  | 5 Overly cautious | A | 145 | 0.02 | 0.6915 | 0.8663 | 58 | 0.07 | 0.4237 | 0.6886 |
|  | 6 Nervousness | A | 146 | -0.04 | 0.4392 | 0.6995 | 60 | 0.06 | 0.4853 | 0.7382 |
|  | 7 Calming difficulty | A | 145 | 0.01 | 0.9266 | 0.9727 | 59 | 0.08 | 0.3475 | 0.6217 |
|  | 8 Emotional numbness | A | 145 | -0.01 | 0.8382 | 0.9329 | 58 | 0.05 | 0.6020 | 0.8158 |
|  | 9 Sense of failure | A | 145 | -0.11 | 0.0418 | 0.2107 | 60 | 0.04 | 0.6440 | 0.8391 |
|  | 10 Self-doubt | A | 146 | -0.05 | 0.3561 | 0.6305 | 59 | 0.02 | 0.8415 | 0.9329 |
|  | 11 Disconnection to others | A | 146 | -0.03 | 0.6010 | 0.8155 | 59 | 0.03 | 0.7535 | 0.8947 |
|  | 12 Social difficulty | A | 145 | 0.00 | 0.9316 | 0.9748 | 60 | 0.22 | 0.0113 | 0.1040 |
| 27 Eating problems | 1 Bad dreams | A | 146 | 0.01 | 0.9129 | 0.9669 | 58 | 0.07 | 0.4230 | 0.6877 |
|  | 2 Reliving events in mind | A | 146 | 0.10 | 0.0679 | 0.2740 | 58 | -0.20 | 0.0262 | 0.1643 |
|  | 3 Avoiding thoughts | A | 145 | 0.09 | 0.1281 | 0.3808 | 56 | 0.08 | 0.3724 | 0.6442 |
|  | 4 Avoiding physically | A | 144 | 0.10 | 0.0669 | 0.2720 | 57 | 0.14 | 0.1167 | 0.3656 |
|  | 5 Overly cautious | A | 145 | 0.01 | 0.8465 | 0.9343 | 56 | 0.06 | 0.5270 | 0.7661 |
|  | 6 Nervousness | A | 145 | -0.01 | 0.9195 | 0.9709 | 58 | -0.07 | 0.4460 | 0.7060 |
|  | 7 Calming difficulty | A | 144 | 0.07 | 0.2402 | 0.5170 | 57 | 0.06 | 0.5454 | 0.7767 |
|  | 8 Emotional numbness | A | 144 | 0.05 | 0.4006 | 0.6677 | 56 | 0.05 | 0.5539 | 0.7815 |
|  | 9 Sense of failure | A | 144 | -0.06 | 0.2644 | 0.5431 | 58 | 0.10 | 0.2830 | 0.5585 |
|  | 10 Self-doubt | A | 145 | -0.08 | 0.1769 | 0.4434 | 57 | 0.08 | 0.3763 | 0.6477 |
|  | 11 Disconnection to others | A | 145 | 0.03 | 0.5640 | 0.7871 | 57 | 0.02 | 0.8413 | 0.9329 |
|  | 12 Social difficulty | A | 144 | 0.08 | 0.1529 | 0.4119 | 58 | 0.20 | 0.0247 | 0.1602 |
| 28 Memorization | 1 Bad dreams | C | 148 | -0.02 | 0.7679 | 0.9032 | 60 | -0.19 | 0.0306 | 0.1773 |
|  | 2 Reliving events in mind | C | 147 | 0.00 | 0.9535 | 0.9857 | 60 | -0.28 | 0.0017 | 0.0312 |
|  | 3 Avoiding thoughts | C | 146 | 0.00 | 0.9734 | 0.9956 | 58 | -0.19 | 0.0380 | 0.1972 |
|  | 4 Avoiding physically | C | 145 | 0.02 | 0.7155 | 0.8802 | 59 | -0.10 | 0.2532 | 0.5324 |
|  | 5 Overly cautious | C | 146 | -0.05 | 0.3259 | 0.6025 | 58 | -0.11 | 0.2368 | 0.5135 |
|  | 6 Nervousness | C | 147 | -0.07 | 0.2046 | 0.4787 | 60 | -0.22 | 0.0128 | 0.1101 |
|  | 7 Calming difficulty | C | 146 | 0.01 | 0.8485 | 0.9350 | 59 | -0.05 | 0.5678 | 0.7898 |
|  | 8 Emotional numbness | C | 146 | -0.01 | 0.7909 | 0.9160 | 58 | -0.02 | 0.8254 | 0.9290 |
|  | 9 Sense of failure | C | 146 | -0.04 | 0.4207 | 0.6851 | 60 | -0.16 | 0.0754 | 0.2905 |
|  | 10 Self-doubt | C | 147 | -0.12 | 0.0310 | 0.1777 | 59 | -0.16 | 0.0774 | 0.2952 |
|  | 11 Disconnection to others | C | 147 | -0.08 | 0.1746 | 0.4408 | 59 | -0.17 | 0.0530 | 0.2374 |
|  | 12 Social difficulty | C | 146 | -0.05 | 0.4184 | 0.6831 | 60 | -0.13 | 0.1457 | 0.4043 |

*B-H* – Benjamini-Hochberg correction for multiple comparisons, *p*-values < 0.05 are indicated in red, N – number of participants, Tau – Tau correlation coefficient
