# Supplementary material for: Demographic predictors of trauma and depression in war-affected children from Poland and Ukraine: Implications for prevention of mental health problems
Source: Prev Med Rep. 2026 Feb 20;63:103422. doi: 10.1016/j.pmedr.2026.103422 (PMC12945576; doi:10.1016/j.pmedr.2026.103422)
Supplement: Supplementary file 2 — Supplementary material 2 [file mmc2.docx]

**CDI (panels A-D) and ITQ-CA correlations in origin-dependent groups**

**CDI2 – group A**

Among the Polish children 31 significant correlations in CDI2 – group A (Figure S2) were observed, most significant correlations between CDI2-18 “Pain thoughts” with 9 ITQ-CA categories, and CDI2-10 “Bad mood” and 7 ITQ-CA categories, followed by CDI2-17 “Eating attitude” and CDI2-26 “Napping/dozing” with 5 ITQ-CA categories (all negative and weak to moderate). The remaining five correlations were negative and very weak (Table S2). The strongest correlations of moderate, negative strength were between CDI2-18 “Pain thoughts” and ITQ-CA categories: “Bad dreams”, “Avoiding thoughts”, and “Nervousness”. After Benjamini-Hochberg correction, 11 significant correlations remain, mostly between CDI2-18 “Pain thoughts”, and “Bad mood” with 5 and 3 ITQ-CA categories, respectively. In Ukrainian children, we observed six significant correlations between CDI2-27 “Eating problems” with ITQ-CA “Avoiding thoughts”, “Avoiding physically”, and “Social difficulty”, CDI2-9 “Tendency to cry” with ITQ-CA “Reliving events in mind”, CDI2-18 “Pain thoughts” with ITQ-CA “overlay cautions”, and CDI2-26 “Napping/dozing” with ITQ-CA “Avoiding physically” – all of very weak strength, four positive and two negative. After the Benjamini-Hochberg correction, none of the correlations remained significant. In the displaced group, we observed 15 significant correlations (12 negative and three positive). The strength of correlations was moderate to strong, most of them between CDI2-15 “Sleep quality” with 4 ITQ-CA categories. After correction for multiple comparisons, five correlations remail significant, one positive (CDI2-1 “Sadness” with ITQ-CA “Avoiding thoughts), and four negative (between CDI2-15 “Sleep quality” with ITQ-CA “Nervousness”, “Emotional numbles” and “Disconnection to others”, and finally CDI2-27 “Eating problems” with ITQ-CA “Nervousness”).

**CDI2 – group B**

In the CDI2 – group B among the Polish participants, 27 significant correlations were observed (all negative and very weak to moderate, Figure S2; Table S2). Most significant correlations were observed between CDI2-2 “Hopeful” and CDI2-13 “Self-perception” (both seven correlations with ITQ-CA), CDI2-7 “Blame”, CDI2-24 “Love awareness”, and ITQ-CA (both two correlations with ITQ-CA; Table S2). After the Benjamini-Hochberg correction, 13 significant correlations remained. In the group of boys, 12 correlations remain significant in the CDI2-2 “Hopeful”, CDI2-7 “Blame”, CDI2-13 “Self-perception” and CDI2-24 “Love awareness” (4, 2, 4, and 2 respectively”. In the Ukrainian group we observed five significant, weak correlations (2 negative between CDI2-7 “Blame” with ITQ-CA “Sense of failure” and “Disconnection to others”, and three negative, regarding CDI2-2 “Hopeful” with “Avoiding physically”, CDI2-8 “Suicide” with “Social difficulty and CDI2-13 “Self-perception” and ITQ-CA “Avoiding physically”. None of them remains significant after Benjamini-Hochberg correction. Seven significant, moderate correlations were observed in the displaced group (Figure S2). Four of them were negative between CDI2-2 “Hopeful” with ITQ-CA “Revealing events in mind” and “Nervousness”, CDI2-6 “Self-acceptance” with “Nervousness”, and CDI2-7 “Blame” with “Nervousness”. The remaining three positive correlations we observed between CDI2-8 ”Suicide” with ITQ-CA “Social difficulty” and CDI2-13 “Self-perception” with ITQ-CA “Self-doubt” and with “Social difficulty”. None of them remains significant after Benjamini-Hochberg correction (Table S2).

**CDI2 – group C**

In the CDI2 – group C among the Polish participants, 23 significant correlations were observed (Figure S2). Most of them were negative and weak to moderate (mostly between CDI2-20 “School fun” with 9 ITQ-CA categories, and CDI2-12 “Self-determination” with 6 ITQ-CA categories). Three correlations were positive and weak (between CDI2-4 “Enjoyment” with ITQ-CA “Avoiding thoughts” and “Overlay cautions”, also with CDI2-14 “Learning attitude” with ITQ-CA “Disconnection to others”). After Benjamini-Hochberg correction, four significant correlations remain between CDI2-12 “Self-determination” with ITQ-CA “Overlay caution”, and CDI2-20 “School fun” with ITQ-CA “Nervousness”, “Calming difficulty”, and “Sense of failure” (Table S2). In the participants from Ukraine, 11 significant correlations were observed (Figure S2). All of them were positive and weak to moderate (between CDI2-4 “Enjoyment” with ITQ-CA “Avoiding thoughts” and “Avoiding physically, CDI2-12 “Self-determination” with “Overlay cautions”, “Disconnection to others” and “Social difficulty”, CDI2-14 “Learning attitude” with ITQ-CA “Bad dreams” and “Overlay cautions”, CDI2-22 “Sealing with school tasks” with “Avoiding thoughts”, “Avoiding physically” and “Calming difficulties”, and CDI2-28 “Memorization” with ITQ-CA “Calming difficulty”. After Benjamini-Hochberg correction, two significant correlations remains between CDI2-22 “Dealing with school tasks” with ITQ-CA “Avoiding physically” and “Avoiding thoughts”. In the displaced group we observed 10 significant moderate to strong correlations – 7 negative, most between CDI2-14 “Learning attitude” and 5 ITQ-CA categories, and three positive (between CDI2-20 “School fun” with ITQ-CA “Calming difficulty” and CDI2-28 “Memorization” with ITQ-CA “Self-doubt” and “Social difficulty”). After Benjamini-Hochberg correction, three significant correlations remain between CDI2-14 “Learning attitude” with ITQ-CA “Nervousness” and “Emotional numbness”, and CDI2-28 “Memorization” and ITQ-CA “Social difficulty” (Table S2).

**CDI2 – group D**

Among the Polish citizens, in CDI2–group D, 17 significant correlations were observed (Figure S2). All significant correlations were negative and weak to moderate, mostly between CDI2-5 “Importance for family” and 10 ITQ-CA categories. After Benjamini-Hochberg correction, six significant correlations remains (CDI2-5 “Importance for family with ITQ-CA “Bad dreams”, “Reliving events in mind”, “Sense for failure”, “Self-doubt” and “Disconnection to others”, and CDI2-11 “Company” and CDI2-19 “Loneliness feeling” with ITQ-CA “Sense of failure” (Table S2). In the Ukrainian group we observed 10 significant, and weak correlations – 7 negative between CDI2-5 “Importance for family” with “Emotional numbness”, CDI2-11 “Company” with ITQ-CA “Sense of failure”, CDI-2-19 “Loneliness feeling” with ITQ-CA “Overlay cautions”, “Sense of failure”, “Self-doubt” and “Disconnection to others”, CDI2-21 “Friends” with “Self-doubt”, and CDI2-25 “Peer arguing” with ITQ-CA “Nervousness” (Figure S2). None of them remains significant after Benjamini-Hochberg correction (Table S2). The displaced children were characterized by 10 significant and moderate correlations Figure S2). Two of them were negative (between CDI2-21 “Friends” with ITQ-CA “Relieving events in mind” and “Nervousness”). The remaining 8 were positive (between CDI2-5 “Importance for family”, “ CDI2-19 “Lineless feeling” and CDI2-25 “Peer arguing” all with ITQ-CA “Self-doubt” and “Social difficulty”. Additionally, CDI2-25 was correlated with ITQ-CA “Avoiding thoughts” and “Overly cautious”. After correction for multiple comparisons, only CDI2-25 “Peer arguing” was significantly correlated with ITQ-CA “Avoiding thoughts” (Table S2).


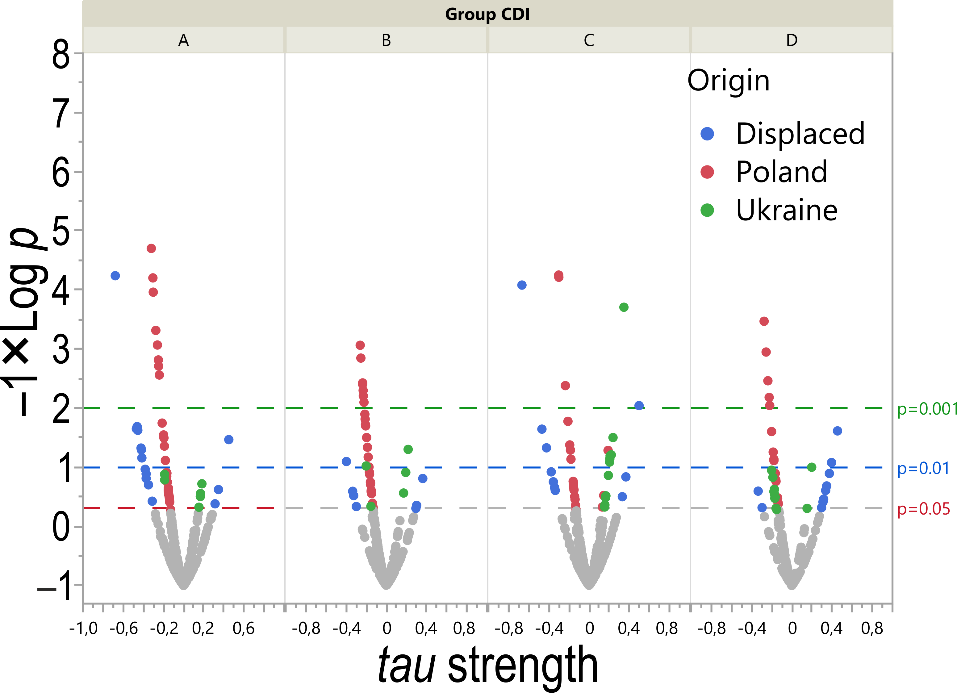


**Figure S2.** Correlations between single questions of CDI-2 (Children's Depression Inventory 2) and ITQ-CA (International Trauma Questionnaire – Child and Adolescent Version) in origin-dependent groups of children and adolescents from Poland and Ukraine. Volcano plot of significant correlations between single questions of CDI and ITQ-CA in the CDI A, B, C, and D groups. The strength of *tau* correlation coefficient (x-axis) and the significance as the unadjusted *p*-values (shown as −1×log *p*; y-axis). The dashed horizontal lines represent the *p* = 0.05 (red), *p* = 0.01 (blue), and *p* = 0.001 (green). Significant correlations are shown as colored spots, and non-significant correlations are grey, March 2024-March 2025, Poland and Ukraine.

**Table S2.** Children's Depression Inventory 2 (CDI-2) (panels A-D) and International Trauma Questionnaire – Child and Adolescent Version (ITQ-CA) correlations in origin dependent groups of children and adolescents from Poland and Ukraine, March 2024-March 2025, Poland and Ukraine.

| **CDI2** | **ITQ-CA** | **Group CDI** | **N** | ***Tau*** | ***p*-value** | ***p*-value ^BH^** | **N** | ***Tau*** | ***p*-value** | ***p*-value ^BH^** | **N** | ***Tau*** | ***p*-value** | ***p*-value ^BH^** |
| --- | --- | --- | --- | --- | --- | --- | --- | --- | --- | --- | --- | --- | --- | --- |
|  |  | **Poland** | | | | | **Ukraine** | | | | **Displaced** | | | |
| 1 Sadness | 1 Bad dreams | A | 101 | 0.05 | 0.4497 | 0.7085 | 79 | -0.01 | 0.9308 | 0.9748 | 22 | 0.01 | 0.9288 | 0.9738 |
|  | 2 Reliving events in mind | A | 100 | 0.00 | 0.9600 | 0.9888 | 79 | -0.09 | 0.2482 | 0.5265 | 22 | -0.19 | 0.2144 | 0.4875 |
|  | 3 Avoiding thoughts | A | 99 | 0.10 | 0.1432 | 0.3993 | 78 | 0.07 | 0.3619 | 0.6348 | 22 | 0.45 | 0.0034 | 0.0478 |
|  | 4 Avoiding physically | A | 99 | 0.09 | 0.1798 | 0.4466 | 77 | 0.02 | 0.8261 | 0.9292 | 22 | 0.17 | 0.2600 | 0.5368 |
|  | 5 Overly cautious | A | 99 | 0.02 | 0.8231 | 0.9290 | 77 | -0.01 | 0.9479 | 0.9825 | 22 | 0.07 | 0.6393 | 0.8362 |
|  | 6 Nervousness | A | 101 | -0.04 | 0.5870 | 0.8047 | 78 | 0.04 | 0.6081 | 0.8192 | 22 | -0.12 | 0.4182 | 0.6830 |
|  | 7 Calming difficulty | A | 100 | 0.04 | 0.5554 | 0.7818 | 78 | 0.09 | 0.2435 | 0.5213 | 21 | 0.13 | 0.3998 | 0.6671 |
|  | 8 Emotional numbness | A | 99 | 0.07 | 0.3349 | 0.6111 | 78 | 0.08 | 0.3229 | 0.5994 | 22 | -0.07 | 0.6677 | 0.8526 |
|  | 9 Sense of failure | A | 101 | -0.03 | 0.6942 | 0.8680 | 77 | 0.14 | 0.0724 | 0.2839 | 22 | 0.17 | 0.2547 | 0.5324 |
|  | 10 Self-doubt | A | 101 | 0.03 | 0.6769 | 0.8577 | 77 | 0.06 | 0.4038 | 0.6692 | 22 | 0.19 | 0.2251 | 0.4988 |
|  | 11 Disconnection to others | A | 100 | -0.02 | 0.7259 | 0.8841 | 78 | 0.09 | 0.2707 | 0.5477 | 22 | -0.07 | 0.6367 | 0.8355 |
|  | 12 Social difficulty | A | 101 | 0.11 | 0.1194 | 0.3686 | 77 | 0.15 | 0.0604 | 0.2555 | 22 | 0.22 | 0.1550 | 0.4149 |
| 2 Hopeful | 1 Bad dreams | B | 105 | -0.20 | 0.0031 | 0.0455 | 80 | 0.04 | 0.6301 | 0.8332 | 23 | -0.03 | 0.8367 | 0.9327 |
|  | 2 Reliving events in mind | B | 104 | -0.24 | 0.0004 | 0.0120 | 80 | -0.04 | 0.6214 | 0.8285 | 23 | -0.30 | 0.0462 | 0.2204 |
|  | 3 Avoiding thoughts | B | 102 | -0.10 | 0.1286 | 0.3812 | 79 | 0.03 | 0.7392 | 0.8895 | 23 | -0.01 | 0.9364 | 0.9759 |
|  | 4 Avoiding physically | B | 103 | -0.13 | 0.0566 | 0.2460 | 78 | 0.22 | 0.0050 | 0.0612 | 23 | 0.14 | 0.3360 | 0.6122 |
|  | 5 Overly cautious | B | 103 | -0.15 | 0.0247 | 0.1602 | 78 | -0.07 | 0.3376 | 0.6134 | 23 | -0.10 | 0.4943 | 0.7429 |
|  | 6 Nervousness | B | 105 | -0.21 | 0.0013 | 0.0260 | 79 | -0.02 | 0.7576 | 0.8972 | 23 | -0.40 | 0.0080 | 0.0836 |
|  | 7 Calming difficulty | B | 104 | -0.15 | 0.0279 | 0.1697 | 79 | 0.11 | 0.1476 | 0.4057 | 22 | 0.04 | 0.8024 | 0.9202 |
|  | 8 Emotional numbness | B | 103 | -0.06 | 0.3729 | 0.6445 | 79 | -0.06 | 0.4019 | 0.6688 | 22 | 0.17 | 0.2712 | 0.5483 |
|  | 9 Sense of failure | B | 105 | -0.23 | 0.0005 | 0.0144 | 78 | -0.09 | 0.2538 | 0.5324 | 23 | 0.03 | 0.8424 | 0.9329 |
|  | 10 Self-doubt | B | 105 | -0.15 | 0.0265 | 0.1643 | 78 | -0.08 | 0.3122 | 0.5894 | 23 | 0.06 | 0.6977 | 0.8705 |
|  | 11 Disconnection to others | B | 104 | -0.11 | 0.0883 | 0.3164 | 79 | -0.07 | 0.3432 | 0.6186 | 23 | -0.04 | 0.8089 | 0.9228 |
|  | 12 Social difficulty | B | 105 | 0.01 | 0.8936 | 0.9577 | 78 | -0.09 | 0.2244 | 0.4981 | 23 | 0.22 | 0.1430 | 0.3993 |
| 3 Self-confidence | 1 Bad dreams | C | 105 | 0.04 | 0.5416 | 0.7744 | 80 | 0.03 | 0.6863 | 0.8627 | 23 | 0.07 | 0.6555 | 0.8447 |
|  | 2 Reliving events in mind | C | 104 | -0.07 | 0.3105 | 0.5874 | 80 | -0.07 | 0.3801 | 0.6513 | 23 | -0.14 | 0.3465 | 0.6210 |
|  | 3 Avoiding thoughts | C | 102 | -0.01 | 0.9015 | 0.9615 | 79 | 0.06 | 0.4156 | 0.6799 | 23 | 0.25 | 0.0917 | 0.3238 |
|  | 4 Avoiding physically | C | 103 | 0.03 | 0.7040 | 0.8749 | 78 | 0.05 | 0.5482 | 0.7772 | 23 | 0.09 | 0.5541 | 0.7815 |
|  | 5 Overly cautious | C | 103 | -0.04 | 0.5415 | 0.7744 | 78 | -0.09 | 0.2197 | 0.4935 | 23 | 0.16 | 0.2934 | 0.5686 |
|  | 6 Nervousness | C | 105 | -0.16 | 0.0188 | 0.1369 | 79 | 0.04 | 0.5845 | 0.8032 | 23 | -0.19 | 0.2050 | 0.4789 |
|  | 7 Calming difficulty | C | 104 | 0.00 | 0.9722 | 0.9948 | 79 | 0.13 | 0.0949 | 0.3289 | 22 | -0.11 | 0.4845 | 0.7376 |
|  | 8 Emotional numbness | C | 103 | 0.01 | 0.9163 | 0.9688 | 79 | 0.02 | 0.7499 | 0.8934 | 22 | -0.12 | 0.4462 | 0.7060 |
|  | 9 Sense of failure | C | 105 | -0.14 | 0.0356 | 0.1903 | 78 | 0.05 | 0.5071 | 0.7497 | 23 | -0.08 | 0.5839 | 0.8025 |
|  | 10 Self-doubt | C | 105 | -0.06 | 0.3724 | 0.6442 | 78 | -0.10 | 0.1859 | 0.4543 | 23 | 0.11 | 0.4678 | 0.7245 |
|  | 11 Disconnection to others | C | 104 | -0.08 | 0.2459 | 0.5243 | 79 | 0.04 | 0.5863 | 0.8044 | 23 | -0.05 | 0.7511 | 0.8935 |
|  | 12 Social difficulty | C | 105 | 0.00 | 0.9918 | 1.0000 | 78 | 0.07 | 0.3954 | 0.6635 | 23 | 0.22 | 0.1391 | 0.3957 |
| 4 Enjoyment | 1 Bad dreams | C | 104 | 0.11 | 0.0971 | 0.3330 | 80 | 0.06 | 0.4450 | 0.7049 | 23 | 0.19 | 0.2054 | 0.4792 |
|  | 2 Reliving events in mind | C | 103 | 0.04 | 0.5151 | 0.7566 | 80 | 0.04 | 0.5640 | 0.7871 | 23 | -0.26 | 0.0792 | 0.2977 |
|  | 3 Avoiding thoughts | C | 101 | 0.19 | 0.0052 | 0.0629 | 79 | 0.21 | 0.0064 | 0.0732 | 23 | 0.04 | 0.7750 | 0.9066 |
|  | 4 Avoiding physically | C | 102 | 0.11 | 0.1129 | 0.3593 | 78 | 0.20 | 0.0082 | 0.0846 | 23 | 0.27 | 0.0709 | 0.2803 |
|  | 5 Overly cautious | C | 102 | 0.15 | 0.0300 | 0.1765 | 78 | -0.05 | 0.4812 | 0.7363 | 23 | 0.26 | 0.0880 | 0.3162 |
|  | 6 Nervousness | C | 104 | 0.03 | 0.6462 | 0.8395 | 79 | -0.04 | 0.5703 | 0.7917 | 23 | 0.03 | 0.8159 | 0.9273 |
|  | 7 Calming difficulty | C | 103 | 0.12 | 0.0798 | 0.2987 | 79 | 0.13 | 0.0919 | 0.3238 | 22 | -0.15 | 0.3337 | 0.6097 |
|  | 8 Emotional numbness | C | 102 | -0.09 | 0.1909 | 0.4601 | 79 | 0.02 | 0.8225 | 0.9290 | 22 | -0.11 | 0.4622 | 0.7201 |
|  | 9 Sense of failure | C | 104 | 0.04 | 0.5924 | 0.8079 | 78 | 0.00 | 0.9785 | 0.9993 | 23 | -0.01 | 0.9351 | 0.9757 |
|  | 10 Self-doubt | C | 104 | -0.08 | 0.2232 | 0.4966 | 78 | 0.04 | 0.5803 | 0.7994 | 23 | 0.24 | 0.1116 | 0.3579 |
|  | 11 Disconnection to others | C | 103 | 0.03 | 0.6817 | 0.8601 | 79 | 0.03 | 0.6554 | 0.8447 | 23 | 0.15 | 0.3219 | 0.5983 |
|  | 12 Social difficulty | C | 104 | -0.01 | 0.8963 | 0.9594 | 78 | 0.04 | 0.5683 | 0.7898 | 23 | 0.18 | 0.2211 | 0.4942 |
| 5 Importance for family | 1 Bad dreams | D | 105 | -0.22 | 0.0009 | 0.0212 | 79 | -0.04 | 0.5929 | 0.8079 | 23 | 0.19 | 0.1937 | 0.4646 |
|  | 2 Reliving events in mind | D | 104 | -0.20 | 0.0025 | 0.0400 | 79 | -0.13 | 0.0793 | 0.2980 | 23 | -0.10 | 0.5165 | 0.7574 |
|  | 3 Avoiding thoughts | D | 102 | -0.05 | 0.4162 | 0.6807 | 78 | 0.00 | 0.9489 | 0.9833 | 23 | -0.23 | 0.1234 | 0.3736 |
|  | 4 Avoiding physically | D | 103 | -0.14 | 0.0396 | 0.2032 | 77 | 0.08 | 0.2890 | 0.5637 | 23 | 0.00 | 1.0000 | 1.0000 |
|  | 5 Overly cautious | D | 103 | -0.19 | 0.0055 | 0.0661 | 77 | -0.06 | 0.4177 | 0.6826 | 23 | 0.07 | 0.6639 | 0.8517 |
|  | 6 Nervousness | D | 105 | -0.16 | 0.0173 | 0.1309 | 78 | -0.03 | 0.6863 | 0.8627 | 23 | -0.10 | 0.5125 | 0.7538 |
|  | 7 Calming difficulty | D | 104 | -0.13 | 0.0587 | 0.2516 | 78 | 0.07 | 0.3518 | 0.6262 | 22 | 0.00 | 1.0000 | 1.0000 |
|  | 8 Emotional numbness | D | 103 | -0.18 | 0.0077 | 0.0819 | 78 | -0.19 | 0.0148 | 0.1203 | 22 | -0.09 | 0.5387 | 0.7728 |
|  | 9 Sense of failure | D | 105 | -0.23 | 0.0007 | 0.0173 | 77 | -0.01 | 0.9477 | 0.9825 | 23 | -0.05 | 0.7463 | 0.8927 |
|  | 10 Self-doubt | D | 105 | -0.26 | 0.0001 | 0.0055 | 77 | -0.02 | 0.8368 | 0.9327 | 23 | 0.30 | 0.0483 | 0.2250 |
|  | 11 Disconnection to others | D | 104 | -0.28 | <0.0001 | 0.0027 | 78 | 0.09 | 0.2534 | 0.5324 | 23 | 0.26 | 0.0780 | 0.2956 |
|  | 12 Social difficulty | D | 105 | -0.16 | 0.0168 | 0.1295 | 77 | 0.01 | 0.8951 | 0.9590 | 23 | 0.35 | 0.0207 | 0.1455 |
| 6 Self-acceptance | 1 Bad dreams | B | 104 | -0.10 | 0.1270 | 0.3800 | 80 | 0.05 | 0.5373 | 0.7717 | 23 | 0.02 | 0.8750 | 0.9478 |
|  | 2 Reliving events in mind | B | 103 | -0.10 | 0.1324 | 0.3857 | 80 | -0.06 | 0.4252 | 0.6902 | 23 | -0.23 | 0.1190 | 0.3686 |
|  | 3 Avoiding thoughts | B | 101 | 0.01 | 0.8410 | 0.9329 | 79 | 0.00 | 0.9881 | 1.0000 | 23 | 0.10 | 0.4937 | 0.7424 |
|  | 4 Avoiding physically | B | 102 | -0.08 | 0.2506 | 0.5296 | 78 | 0.09 | 0.2306 | 0.5063 | 23 | -0.06 | 0.6741 | 0.8556 |
|  | 5 Overly cautious | B | 102 | -0.06 | 0.3602 | 0.6333 | 78 | -0.02 | 0.7738 | 0.9057 | 23 | 0.14 | 0.3487 | 0.6233 |
|  | 6 Nervousness | B | 104 | -0.13 | 0.0493 | 0.2284 | 79 | -0.07 | 0.3311 | 0.6078 | 23 | -0.32 | 0.0302 | 0.1769 |
|  | 7 Calming difficulty | B | 103 | -0.04 | 0.5433 | 0.7766 | 79 | -0.14 | 0.0680 | 0.2741 | 22 | -0.10 | 0.5218 | 0.7611 |
|  | 8 Emotional numbness | B | 102 | -0.01 | 0.8388 | 0.9329 | 79 | -0.08 | 0.3165 | 0.5923 | 22 | -0.02 | 0.9027 | 0.9622 |
|  | 9 Sense of failure | B | 104 | -0.13 | 0.0475 | 0.2241 | 78 | -0.10 | 0.2095 | 0.4844 | 23 | -0.14 | 0.3627 | 0.6355 |
|  | 10 Self-doubt | B | 104 | -0.01 | 0.9215 | 0.9716 | 78 | -0.01 | 0.8682 | 0.9449 | 23 | 0.06 | 0.6837 | 0.8606 |
|  | 11 Disconnection to others | B | 103 | -0.10 | 0.1392 | 0.3957 | 79 | 0.05 | 0.5230 | 0.7621 | 23 | -0.13 | 0.3977 | 0.6656 |
|  | 12 Social difficulty | B | 104 | 0.00 | 0.9842 | 1.0000 | 78 | 0.03 | 0.7408 | 0.8899 | 23 | 0.13 | 0.3776 | 0.6491 |
| 7 Blame | 1 Bad dreams | B | 103 | -0.21 | 0.0019 | 0.0336 | 80 | 0.09 | 0.2126 | 0.4863 | 23 | 0.06 | 0.6663 | 0.8526 |
|  | 2 Reliving events in mind | B | 102 | -0.17 | 0.0122 | 0.1076 | 80 | -0.05 | 0.5217 | 0.7611 | 23 | -0.05 | 0.7329 | 0.8877 |
|  | 3 Avoiding thoughts | B | 100 | -0.12 | 0.0840 | 0.3072 | 79 | 0.01 | 0.8596 | 0.9402 | 23 | -0.07 | 0.6491 | 0.8413 |
|  | 4 Avoiding physically | B | 101 | -0.11 | 0.0904 | 0.3215 | 78 | 0.03 | 0.7387 | 0.8895 | 23 | 0.26 | 0.0795 | 0.2983 |
|  | 5 Overly cautious | B | 101 | -0.16 | 0.0194 | 0.1384 | 78 | 0.11 | 0.1370 | 0.3923 | 23 | 0.08 | 0.5974 | 0.8120 |
|  | 6 Nervousness | B | 103 | -0.23 | 0.0006 | 0.0166 | 79 | -0.02 | 0.7493 | 0.8934 | 23 | -0.33 | 0.0255 | 0.1630 |
|  | 7 Calming difficulty | B | 102 | -0.10 | 0.1227 | 0.3733 | 79 | -0.08 | 0.2731 | 0.5496 | 22 | 0.01 | 0.9366 | 0.9759 |
|  | 8 Emotional numbness | B | 101 | -0.05 | 0.4366 | 0.6983 | 79 | -0.11 | 0.1371 | 0.3923 | 22 | -0.03 | 0.8701 | 0.9460 |
|  | 9 Sense of failure | B | 103 | -0.13 | 0.0523 | 0.2362 | 78 | -0.20 | 0.0095 | 0.0933 | 23 | -0.10 | 0.4961 | 0.7437 |
|  | 10 Self-doubt | B | 103 | -0.10 | 0.1502 | 0.4083 | 78 | -0.10 | 0.1847 | 0.4535 | 23 | 0.01 | 0.9705 | 0.9936 |
|  | 11 Disconnection to others | B | 102 | -0.17 | 0.0131 | 0.1111 | 79 | -0.15 | 0.0458 | 0.2197 | 23 | 0.02 | 0.8782 | 0.9494 |
|  | 12 Social difficulty | B | 103 | -0.12 | 0.0746 | 0.2893 | 78 | -0.14 | 0.0750 | 0.2900 | 23 | 0.10 | 0.4862 | 0.7382 |
| 8 Suicide | 1 Bad dreams | B | 102 | -0.06 | 0.3333 | 0.6095 | 80 | 0.08 | 0.2694 | 0.5470 | 23 | 0.22 | 0.1487 | 0.4061 |
|  | 2 Reliving events in mind | B | 101 | -0.09 | 0.1710 | 0.4373 | 80 | -0.12 | 0.1209 | 0.3708 | 23 | -0.14 | 0.3518 | 0.6262 |
|  | 3 Avoiding thoughts | B | 99 | -0.02 | 0.7386 | 0.8895 | 79 | 0.09 | 0.2162 | 0.4892 | 23 | 0.23 | 0.1276 | 0.3800 |
|  | 4 Avoiding physically | B | 100 | -0.08 | 0.2225 | 0.4961 | 78 | 0.09 | 0.2309 | 0.5064 | 23 | 0.07 | 0.6397 | 0.8362 |
|  | 5 Overly cautious | B | 100 | -0.05 | 0.4679 | 0.7245 | 78 | -0.05 | 0.5064 | 0.7497 | 23 | 0.14 | 0.3644 | 0.6375 |
|  | 6 Nervousness | B | 102 | -0.09 | 0.2050 | 0.4789 | 79 | -0.02 | 0.8145 | 0.9264 | 23 | -0.17 | 0.2601 | 0.5368 |
|  | 7 Calming difficulty | B | 101 | -0.02 | 0.7444 | 0.8917 | 79 | -0.05 | 0.5440 | 0.7766 | 22 | 0.03 | 0.8247 | 0.9290 |
|  | 8 Emotional numbness | B | 100 | -0.08 | 0.2285 | 0.5034 | 79 | 0.05 | 0.5441 | 0.7766 | 22 | -0.13 | 0.4070 | 0.6715 |
|  | 9 Sense of failure | B | 102 | -0.14 | 0.0406 | 0.2064 | 78 | -0.10 | 0.1887 | 0.4575 | 23 | 0.04 | 0.8001 | 0.9192 |
|  | 10 Self-doubt | B | 102 | -0.11 | 0.0955 | 0.3301 | 78 | -0.09 | 0.2517 | 0.5313 | 23 | 0.27 | 0.0697 | 0.2776 |
|  | 11 Disconnection to others | B | 101 | -0.07 | 0.2899 | 0.5645 | 79 | 0.07 | 0.3435 | 0.6186 | 23 | -0.02 | 0.8979 | 0.9599 |
|  | 12 Social difficulty | B | 102 | 0.02 | 0.7828 | 0.9113 | 78 | 0.19 | 0.0122 | 0.1081 | 23 | 0.29 | 0.0495 | 0.2288 |
| 9 Tendency to cry | 1 Bad dreams | A | 103 | -0.11 | 0.0969 | 0.3328 | 80 | 0.05 | 0.5396 | 0.7729 | 23 | 0.02 | 0.8779 | 0.9493 |
|  | 2 Reliving events in mind | A | 102 | -0.15 | 0.0294 | 0.1754 | 80 | -0.19 | 0.0132 | 0.1111 | 23 | -0.31 | 0.0375 | 0.1960 |
|  | 3 Avoiding thoughts | A | 100 | -0.10 | 0.1388 | 0.3957 | 79 | 0.04 | 0.6084 | 0.8194 | 23 | 0.24 | 0.1022 | 0.3421 |
|  | 4 Avoiding physically | A | 101 | -0.05 | 0.4872 | 0.7388 | 78 | 0.01 | 0.8918 | 0.9568 | 23 | 0.06 | 0.6812 | 0.8601 |
|  | 5 Overly cautious | A | 101 | -0.08 | 0.2613 | 0.5382 | 78 | -0.02 | 0.8451 | 0.9342 | 23 | -0.05 | 0.7202 | 0.8824 |
|  | 6 Nervousness | A | 103 | -0.19 | 0.0044 | 0.0563 | 79 | -0.12 | 0.1230 | 0.3736 | 23 | -0.35 | 0.0198 | 0.1401 |
|  | 7 Calming difficulty | A | 102 | -0.12 | 0.0860 | 0.3116 | 79 | -0.08 | 0.3234 | 0.6001 | 22 | 0.12 | 0.4293 | 0.6920 |
|  | 8 Emotional numbness | A | 101 | -0.05 | 0.5046 | 0.7482 | 79 | 0.00 | 0.9588 | 0.9886 | 22 | -0.10 | 0.4994 | 0.7458 |
|  | 9 Sense of failure | A | 103 | -0.13 | 0.0535 | 0.2377 | 78 | -0.11 | 0.1481 | 0.4061 | 23 | -0.06 | 0.6837 | 0.8606 |
|  | 10 Self-doubt | A | 103 | -0.12 | 0.0777 | 0.2954 | 78 | -0.01 | 0.9001 | 0.9606 | 23 | -0.02 | 0.9136 | 0.9670 |
|  | 11 Disconnection to others | A | 102 | -0.01 | 0.8318 | 0.9305 | 79 | 0.03 | 0.6721 | 0.8546 | 23 | -0.25 | 0.0911 | 0.3230 |
|  | 12 Social difficulty | A | 103 | 0.08 | 0.2489 | 0.5271 | 78 | 0.01 | 0.9063 | 0.9652 | 23 | 0.09 | 0.5656 | 0.7886 |
| 10 Bad mood | 1 Bad dreams | A | 105 | -0.09 | 0.1850 | 0.4535 | 80 | 0.10 | 0.1983 | 0.4695 | 23 | 0.02 | 0.8772 | 0.9490 |
|  | 2 Reliving events in mind | A | 104 | -0.14 | 0.0314 | 0.1782 | 80 | -0.06 | 0.4095 | 0.6739 | 23 | -0.42 | 0.0051 | 0.0621 |
|  | 3 Avoiding thoughts | A | 102 | -0.06 | 0.3416 | 0.6166 | 79 | 0.09 | 0.2502 | 0.5292 | 23 | 0.13 | 0.3902 | 0.6589 |
|  | 4 Avoiding physically | A | 103 | -0.14 | 0.0370 | 0.1946 | 78 | 0.10 | 0.1905 | 0.4598 | 23 | 0.13 | 0.3674 | 0.6396 |
|  | 5 Overly cautious | A | 103 | -0.10 | 0.1345 | 0.3898 | 78 | 0.05 | 0.4831 | 0.7372 | 23 | -0.10 | 0.5219 | 0.7611 |
|  | 6 Nervousness | A | 105 | -0.25 | 0.0002 | 0.0066 | 79 | -0.08 | 0.2863 | 0.5611 | 23 | -0.37 | 0.0130 | 0.1107 |
|  | 7 Calming difficulty | A | 104 | -0.16 | 0.0178 | 0.1323 | 79 | 0.03 | 0.7088 | 0.8769 | 22 | 0.20 | 0.1893 | 0.4583 |
|  | 8 Emotional numbness | A | 103 | -0.15 | 0.0241 | 0.1594 | 79 | 0.08 | 0.2835 | 0.5586 | 22 | 0.01 | 0.9357 | 0.9757 |
|  | 9 Sense of failure | A | 105 | -0.26 | 0.0001 | 0.0048 | 78 | -0.02 | 0.8082 | 0.9228 | 23 | 0.00 | 1.0000 | 1.0000 |
|  | 10 Self-doubt | A | 105 | -0.24 | 0.0003 | 0.0098 | 78 | 0.06 | 0.4602 | 0.7182 | 23 | 0.19 | 0.2160 | 0.4890 |
|  | 11 Disconnection to others | A | 104 | -0.13 | 0.0600 | 0.2549 | 79 | 0.05 | 0.4897 | 0.7404 | 23 | -0.08 | 0.5711 | 0.7921 |
|  | 12 Social difficulty | A | 105 | -0.10 | 0.1250 | 0.3762 | 78 | 0.12 | 0.1349 | 0.3900 | 23 | 0.26 | 0.0768 | 0.2941 |
| 11 Company | 1 Bad dreams | D | 104 | -0.05 | 0.4125 | 0.6768 | 80 | 0.09 | 0.2253 | 0.4989 | 23 | 0.07 | 0.6596 | 0.8480 |
|  | 2 Reliving events in mind | D | 103 | -0.09 | 0.2014 | 0.4731 | 80 | 0.08 | 0.3085 | 0.5847 | 23 | -0.27 | 0.0687 | 0.2751 |
|  | 3 Avoiding thoughts | D | 101 | -0.09 | 0.1612 | 0.4246 | 79 | 0.20 | 0.0100 | 0.0964 | 23 | 0.21 | 0.1632 | 0.4269 |
|  | 4 Avoiding physically | D | 102 | -0.04 | 0.5155 | 0.7569 | 78 | 0.15 | 0.0497 | 0.2292 | 23 | 0.03 | 0.8153 | 0.9269 |
|  | 5 Overly cautious | D | 102 | -0.07 | 0.3086 | 0.5847 | 78 | -0.01 | 0.9344 | 0.9757 | 23 | 0.05 | 0.7399 | 0.8895 |
|  | 6 Nervousness | D | 104 | -0.03 | 0.6018 | 0.8158 | 79 | -0.05 | 0.4903 | 0.7409 | 23 | 0.08 | 0.5809 | 0.7997 |
|  | 7 Calming difficulty | D | 103 | -0.02 | 0.7316 | 0.8866 | 79 | 0.01 | 0.9338 | 0.9757 | 22 | -0.08 | 0.5916 | 0.8076 |
|  | 8 Emotional numbness | D | 102 | -0.02 | 0.7361 | 0.8887 | 79 | 0.04 | 0.6230 | 0.8295 | 22 | -0.14 | 0.3651 | 0.6379 |
|  | 9 Sense of failure | D | 104 | -0.24 | 0.0003 | 0.0110 | 78 | -0.17 | 0.0320 | 0.1804 | 23 | 0.12 | 0.4398 | 0.6998 |
|  | 10 Self-doubt | D | 104 | -0.08 | 0.2454 | 0.5243 | 78 | -0.07 | 0.3843 | 0.6551 | 23 | 0.02 | 0.9099 | 0.9661 |
|  | 11 Disconnection to others | D | 103 | -0.10 | 0.1418 | 0.3983 | 79 | 0.07 | 0.3739 | 0.6451 | 23 | -0.01 | 0.9688 | 0.9929 |
|  | 12 Social difficulty | D | 104 | -0.02 | 0.8015 | 0.9196 | 78 | 0.01 | 0.9347 | 0.9757 | 23 | 0.12 | 0.4316 | 0.6944 |
| 12 Self-determination | 1 Bad dreams | C | 105 | -0.14 | 0.0312 | 0.1782 | 79 | 0.02 | 0.7922 | 0.9160 | 23 | 0.09 | 0.5543 | 0.7815 |
|  | 2 Reliving events in mind | C | 104 | -0.14 | 0.0314 | 0.1782 | 79 | -0.02 | 0.7711 | 0.9044 | 23 | 0.15 | 0.3116 | 0.5886 |
|  | 3 Avoiding thoughts | C | 102 | -0.15 | 0.0220 | 0.1513 | 78 | 0.05 | 0.5105 | 0.7526 | 23 | 0.04 | 0.7918 | 0.9160 |
|  | 4 Avoiding physically | C | 103 | -0.13 | 0.0442 | 0.2158 | 77 | 0.13 | 0.0981 | 0.3347 | 23 | 0.06 | 0.6905 | 0.8660 |
|  | 5 Overly cautious | C | 103 | -0.24 | 0.0004 | 0.0126 | 77 | 0.15 | 0.0471 | 0.2227 | 23 | 0.06 | 0.6713 | 0.8544 |
|  | 6 Nervousness | C | 105 | -0.13 | 0.0548 | 0.2406 | 78 | 0.01 | 0.9229 | 0.9719 | 23 | -0.24 | 0.1121 | 0.3586 |
|  | 7 Calming difficulty | C | 104 | -0.13 | 0.0522 | 0.2360 | 78 | 0.07 | 0.3403 | 0.6157 | 22 | 0.05 | 0.7486 | 0.8934 |
|  | 8 Emotional numbness | C | 103 | -0.08 | 0.2043 | 0.4781 | 78 | 0.07 | 0.3649 | 0.6379 | 22 | 0.05 | 0.7450 | 0.8919 |
|  | 9 Sense of failure | C | 105 | -0.14 | 0.0350 | 0.1884 | 77 | 0.04 | 0.5633 | 0.7869 | 23 | 0.09 | 0.5390 | 0.7728 |
|  | 10 Self-doubt | C | 105 | -0.10 | 0.1239 | 0.3743 | 77 | -0.02 | 0.8425 | 0.9329 | 23 | 0.02 | 0.8977 | 0.9599 |
|  | 11 Disconnection to others | C | 104 | -0.08 | 0.2178 | 0.4907 | 78 | 0.17 | 0.0305 | 0.1771 | 23 | -0.03 | 0.8588 | 0.9402 |
|  | 12 Social difficulty | C | 105 | -0.12 | 0.0788 | 0.2970 | 77 | 0.19 | 0.0137 | 0.1144 | 23 | -0.04 | 0.7986 | 0.9192 |
| 13 Self-perception | 1 Bad dreams | B | 105 | -0.26 | 0.0001 | 0.0048 | 79 | 0.09 | 0.2673 | 0.5470 | 23 | 0.17 | 0.2673 | 0.5470 |
|  | 2 Reliving events in mind | B | 104 | -0.13 | 0.0508 | 0.2326 | 79 | 0.00 | 1.0000 | 1.0000 | 23 | -0.21 | 0.1525 | 0.4114 |
|  | 3 Avoiding thoughts | B | 102 | -0.13 | 0.0541 | 0.2394 | 78 | 0.14 | 0.0800 | 0.2987 | 23 | -0.15 | 0.3015 | 0.5794 |
|  | 4 Avoiding physically | B | 103 | -0.16 | 0.0178 | 0.1323 | 77 | 0.17 | 0.0273 | 0.1670 | 23 | -0.02 | 0.8730 | 0.9471 |
|  | 5 Overly cautious | B | 103 | -0.10 | 0.1194 | 0.3686 | 77 | -0.05 | 0.5201 | 0.7601 | 23 | 0.22 | 0.1361 | 0.3919 |
|  | 6 Nervousness | B | 105 | -0.22 | 0.0008 | 0.0200 | 78 | 0.03 | 0.7299 | 0.8861 | 23 | -0.15 | 0.3262 | 0.6027 |
|  | 7 Calming difficulty | B | 104 | -0.21 | 0.0015 | 0.0296 | 78 | 0.12 | 0.1324 | 0.3857 | 22 | 0.04 | 0.7695 | 0.9034 |
|  | 8 Emotional numbness | B | 103 | -0.17 | 0.0099 | 0.0961 | 78 | -0.02 | 0.7960 | 0.9183 | 22 | 0.21 | 0.1726 | 0.4398 |
|  | 9 Sense of failure | B | 105 | -0.24 | 0.0004 | 0.0117 | 77 | -0.05 | 0.5240 | 0.7630 | 23 | 0.03 | 0.8429 | 0.9332 |
|  | 10 Self-doubt | B | 105 | -0.19 | 0.0046 | 0.0581 | 77 | 0.00 | 0.9649 | 0.9912 | 23 | 0.30 | 0.0441 | 0.2156 |
|  | 11 Disconnection to others | B | 104 | -0.11 | 0.0868 | 0.3130 | 78 | 0.01 | 0.9306 | 0.9748 | 23 | 0.15 | 0.3152 | 0.5915 |
|  | 12 Social difficulty | B | 105 | -0.06 | 0.3844 | 0.6551 | 77 | 0.00 | 0.9599 | 0.9888 | 23 | 0.36 | 0.0155 | 0.1240 |
| 14 Learning attitude | 1 Bad dreams | C | 105 | 0.02 | 0.7959 | 0.9183 | 79 | 0.16 | 0.0326 | 0.1822 | 23 | -0.07 | 0.6410 | 0.8373 |
|  | 2 Reliving events in mind | C | 104 | 0.01 | 0.8510 | 0.9372 | 79 | 0.08 | 0.2887 | 0.5637 | 23 | -0.20 | 0.1909 | 0.4601 |
|  | 3 Avoiding thoughts | C | 102 | 0.06 | 0.4089 | 0.6735 | 78 | 0.12 | 0.1273 | 0.3800 | 23 | 0.19 | 0.2066 | 0.4808 |
|  | 4 Avoiding physically | C | 103 | 0.08 | 0.2598 | 0.5368 | 77 | 0.05 | 0.4934 | 0.7423 | 23 | -0.24 | 0.1125 | 0.3588 |
|  | 5 Overly cautious | C | 103 | 0.08 | 0.2222 | 0.4961 | 77 | 0.16 | 0.0433 | 0.2146 | 23 | -0.38 | 0.0120 | 0.1068 |
|  | 6 Nervousness | C | 105 | -0.01 | 0.8848 | 0.9529 | 78 | -0.01 | 0.9118 | 0.9667 | 23 | -0.67 | <0.0001 | 0.0012 |
|  | 7 Calming difficulty | C | 104 | 0.01 | 0.8357 | 0.9324 | 78 | 0.10 | 0.1912 | 0.4604 | 22 | -0.20 | 0.2001 | 0.4715 |
|  | 8 Emotional numbness | C | 103 | -0.03 | 0.6828 | 0.8604 | 78 | 0.04 | 0.5883 | 0.8059 | 22 | -0.47 | 0.0023 | 0.0377 |
|  | 9 Sense of failure | C | 105 | 0.07 | 0.2823 | 0.5585 | 77 | 0.04 | 0.6326 | 0.8345 | 23 | -0.24 | 0.1054 | 0.3484 |
|  | 10 Self-doubt | C | 105 | -0.10 | 0.1201 | 0.3698 | 77 | 0.03 | 0.6846 | 0.8614 | 23 | -0.36 | 0.0177 | 0.1320 |
|  | 11 Disconnection to others | C | 104 | 0.13 | 0.0471 | 0.2227 | 78 | -0.03 | 0.7053 | 0.8755 | 23 | -0.42 | 0.0047 | 0.0589 |
|  | 12 Social difficulty | C | 105 | 0.04 | 0.5166 | 0.7574 | 77 | 0.05 | 0.4860 | 0.7382 | 23 | -0.22 | 0.1502 | 0.4083 |
| 15 Sleep quality | 1 Bad dreams | A | 104 | 0.01 | 0.8547 | 0.9390 | 80 | 0.01 | 0.9099 | 0.9661 | 23 | 0.07 | 0.6411 | 0.8373 |
|  | 2 Reliving events in mind | A | 103 | 0.04 | 0.5754 | 0.7955 | 80 | 0.01 | 0.8788 | 0.9495 | 23 | -0.04 | 0.7925 | 0.9160 |
|  | 3 Avoiding thoughts | A | 101 | -0.08 | 0.2386 | 0.5155 | 79 | 0.01 | 0.8919 | 0.9568 | 23 | 0.22 | 0.1428 | 0.3993 |
|  | 4 Avoiding physically | A | 102 | -0.06 | 0.4012 | 0.6682 | 78 | -0.05 | 0.4806 | 0.7357 | 23 | -0.07 | 0.6233 | 0.8295 |
|  | 5 Overly cautious | A | 102 | -0.05 | 0.4137 | 0.6781 | 78 | 0.07 | 0.3374 | 0.6134 | 23 | -0.17 | 0.2596 | 0.5368 |
|  | 6 Nervousness | A | 104 | -0.09 | 0.1854 | 0.4535 | 79 | -0.13 | 0.0991 | 0.3367 | 23 | -0.68 | <0.0001 | 0.0012 |
|  | 7 Calming difficulty | A | 103 | -0.11 | 0.0999 | 0.3380 | 79 | -0.05 | 0.5069 | 0.7497 | 22 | -0.20 | 0.1893 | 0.4583 |
|  | 8 Emotional numbness | A | 102 | -0.06 | 0.3573 | 0.6314 | 79 | 0.09 | 0.2363 | 0.5132 | 22 | -0.47 | 0.0022 | 0.0377 |
|  | 9 Sense of failure | A | 104 | -0.13 | 0.0522 | 0.2360 | 78 | -0.08 | 0.3139 | 0.5904 | 23 | -0.38 | 0.0108 | 0.1018 |
|  | 10 Self-doubt | A | 104 | -0.17 | 0.0126 | 0.1092 | 78 | 0.08 | 0.2797 | 0.5553 | 23 | -0.14 | 0.3601 | 0.6333 |
|  | 11 Disconnection to others | A | 103 | -0.03 | 0.6087 | 0.8195 | 79 | -0.02 | 0.7684 | 0.9034 | 23 | -0.45 | 0.0024 | 0.0389 |
|  | 12 Social difficulty | A | 104 | -0.12 | 0.0805 | 0.2987 | 78 | 0.11 | 0.1690 | 0.4364 | 23 | -0.18 | 0.2168 | 0.4898 |
| 16 Tiredness level | 1 Bad dreams | A | 103 | -0.01 | 0.9253 | 0.9720 | 79 | 0.06 | 0.4558 | 0.7146 | 23 | 0.07 | 0.6391 | 0.8362 |
|  | 2 Reliving events in mind | A | 102 | 0.00 | 0.9921 | 1.0000 | 79 | -0.10 | 0.2041 | 0.4781 | 23 | -0.21 | 0.1619 | 0.4254 |
|  | 3 Avoiding thoughts | A | 100 | -0.01 | 0.8865 | 0.9537 | 78 | 0.11 | 0.1673 | 0.4334 | 23 | 0.23 | 0.1305 | 0.3834 |
|  | 4 Avoiding physically | A | 101 | -0.02 | 0.7497 | 0.8934 | 77 | 0.05 | 0.5622 | 0.7861 | 23 | 0.02 | 0.9092 | 0.9661 |
|  | 5 Overly cautious | A | 101 | -0.05 | 0.4464 | 0.7061 | 77 | -0.04 | 0.6079 | 0.8192 | 23 | 0.12 | 0.4416 | 0.7015 |
|  | 6 Nervousness | A | 103 | -0.10 | 0.1530 | 0.4120 | 78 | -0.03 | 0.6913 | 0.8663 | 23 | -0.10 | 0.4950 | 0.7435 |
|  | 7 Calming difficulty | A | 102 | -0.08 | 0.2478 | 0.5264 | 78 | 0.07 | 0.3354 | 0.6116 | 22 | -0.27 | 0.0744 | 0.2892 |
|  | 8 Emotional numbness | A | 101 | 0.02 | 0.7790 | 0.9096 | 78 | 0.03 | 0.7401 | 0.8895 | 22 | -0.41 | 0.0070 | 0.0766 |
|  | 9 Sense of failure | A | 103 | -0.02 | 0.8136 | 0.9264 | 77 | -0.07 | 0.3922 | 0.6605 | 23 | -0.19 | 0.2135 | 0.4868 |
|  | 10 Self-doubt | A | 103 | -0.08 | 0.2401 | 0.5170 | 77 | -0.13 | 0.0934 | 0.3254 | 23 | 0.01 | 0.9706 | 0.9936 |
|  | 11 Disconnection to others | A | 102 | -0.02 | 0.7511 | 0.8935 | 78 | -0.04 | 0.6457 | 0.8395 | 23 | -0.08 | 0.5927 | 0.8079 |
|  | 12 Social difficulty | A | 103 | -0.06 | 0.3739 | 0.6451 | 77 | 0.05 | 0.5214 | 0.7611 | 23 | 0.23 | 0.1248 | 0.3761 |
| 17 Eating attitude | 1 Bad dreams | A | 104 | -0.12 | 0.0642 | 0.2648 | 79 | 0.10 | 0.1738 | 0.4408 | 22 | 0.27 | 0.0752 | 0.2901 |
|  | 2 Reliving events in mind | A | 103 | -0.16 | 0.0178 | 0.1323 | 79 | 0.01 | 0.8752 | 0.9478 | 22 | -0.10 | 0.5204 | 0.7601 |
|  | 3 Avoiding thoughts | A | 101 | -0.17 | 0.0124 | 0.1085 | 78 | 0.12 | 0.1156 | 0.3654 | 22 | 0.31 | 0.0415 | 0.2099 |
|  | 4 Avoiding physically | A | 102 | -0.11 | 0.1169 | 0.3660 | 77 | 0.13 | 0.0825 | 0.3038 | 22 | 0.29 | 0.0631 | 0.2621 |
|  | 5 Overly cautious | A | 102 | -0.12 | 0.0787 | 0.2970 | 77 | 0.08 | 0.3217 | 0.5983 | 22 | 0.24 | 0.1117 | 0.3579 |
|  | 6 Nervousness | A | 104 | -0.25 | 0.0002 | 0.0078 | 78 | 0.05 | 0.5306 | 0.7684 | 22 | -0.07 | 0.6692 | 0.8531 |
|  | 7 Calming difficulty | A | 103 | -0.11 | 0.0968 | 0.3328 | 78 | 0.01 | 0.9328 | 0.9751 | 21 | 0.06 | 0.7265 | 0.8841 |
|  | 8 Emotional numbness | A | 102 | -0.12 | 0.0642 | 0.2648 | 78 | 0.06 | 0.4391 | 0.6995 | 21 | -0.23 | 0.1482 | 0.4061 |
|  | 9 Sense of failure | A | 104 | -0.20 | 0.0028 | 0.0428 | 77 | -0.11 | 0.1462 | 0.4044 | 22 | 0.02 | 0.8727 | 0.9471 |
|  | 10 Self-doubt | A | 104 | -0.10 | 0.1249 | 0.3761 | 77 | -0.02 | 0.7896 | 0.9158 | 22 | 0.21 | 0.1794 | 0.4465 |
|  | 11 Disconnection to others | A | 103 | -0.14 | 0.0389 | 0.2007 | 78 | 0.06 | 0.4385 | 0.6995 | 22 | -0.03 | 0.8677 | 0.9447 |
|  | 12 Social difficulty | A | 104 | -0.09 | 0.1940 | 0.4646 | 77 | 0.08 | 0.3103 | 0.5873 | 22 | 0.35 | 0.0239 | 0.1584 |
| 18 Pain thoughts | 1 Bad dreams | A | 101 | -0.32 | <0.0001 | 0.0008 | 78 | -0.09 | 0.2655 | 0.5449 | 23 | -0.19 | 0.2135 | 0.4868 |
|  | 2 Reliving events in mind | A | 100 | -0.28 | <0.0001 | 0.0033 | 78 | -0.03 | 0.6545 | 0.8440 | 23 | -0.07 | 0.6364 | 0.8355 |
|  | 3 Avoiding thoughts | A | 98 | -0.30 | <0.0001 | 0.0015 | 77 | -0.02 | 0.7522 | 0.8943 | 23 | -0.23 | 0.1219 | 0.3723 |
|  | 4 Avoiding physically | A | 99 | -0.17 | 0.0142 | 0.1170 | 76 | 0.06 | 0.4743 | 0.7307 | 23 | 0.03 | 0.8290 | 0.9297 |
|  | 5 Overly cautious | A | 99 | -0.21 | 0.0018 | 0.0324 | 76 | -0.19 | 0.0164 | 0.1275 | 23 | 0.07 | 0.6452 | 0.8394 |
|  | 6 Nervousness | A | 101 | -0.30 | <0.0001 | 0.0012 | 77 | -0.02 | 0.8248 | 0.9290 | 23 | 0.27 | 0.0662 | 0.2701 |
|  | 7 Calming difficulty | A | 100 | -0.18 | 0.0077 | 0.0817 | 77 | 0.12 | 0.1282 | 0.3808 | 22 | -0.04 | 0.8189 | 0.9285 |
|  | 8 Emotional numbness | A | 99 | -0.16 | 0.0178 | 0.1323 | 77 | -0.04 | 0.6287 | 0.8325 | 22 | 0.17 | 0.2709 | 0.5479 |
|  | 9 Sense of failure | A | 101 | -0.13 | 0.0602 | 0.2550 | 76 | -0.01 | 0.8659 | 0.9436 | 23 | -0.16 | 0.2840 | 0.5586 |
|  | 10 Self-doubt | A | 101 | -0.15 | 0.0252 | 0.1621 | 77 | -0.12 | 0.1192 | 0.3686 | 23 | -0.13 | 0.4025 | 0.6689 |
|  | 11 Disconnection to others | A | 100 | -0.12 | 0.0774 | 0.2952 | 77 | 0.03 | 0.7431 | 0.8910 | 23 | 0.10 | 0.5147 | 0.7561 |
|  | 12 Social difficulty | A | 101 | -0.13 | 0.0617 | 0.2584 | 76 | -0.03 | 0.6880 | 0.8639 | 23 | -0.28 | 0.0616 | 0.2581 |
| 19 Loneliness feeling | 1 Bad dreams | D | 105 | -0.03 | 0.6046 | 0.8171 | 75 | 0.00 | 0.9828 | 1.0000 | 23 | 0.11 | 0.4467 | 0.7064 |
|  | 2 Reliving events in mind | D | 104 | -0.01 | 0.9152 | 0.9682 | 75 | -0.13 | 0.1048 | 0.3474 | 23 | -0.23 | 0.1309 | 0.3834 |
|  | 3 Avoiding thoughts | D | 102 | -0.06 | 0.3776 | 0.6491 | 74 | 0.08 | 0.3413 | 0.6166 | 23 | 0.01 | 0.9383 | 0.9762 |
|  | 4 Avoiding physically | D | 103 | -0.05 | 0.4660 | 0.7234 | 73 | 0.07 | 0.3592 | 0.6332 | 23 | 0.05 | 0.7260 | 0.8841 |
|  | 5 Overly cautious | D | 103 | -0.04 | 0.5547 | 0.7815 | 73 | -0.16 | 0.0496 | 0.2288 | 23 | 0.12 | 0.4065 | 0.6714 |
|  | 6 Nervousness | D | 105 | -0.13 | 0.0416 | 0.2099 | 74 | -0.09 | 0.2437 | 0.5213 | 23 | -0.09 | 0.5559 | 0.7819 |
|  | 7 Calming difficulty | D | 104 | -0.05 | 0.4261 | 0.6905 | 74 | -0.02 | 0.7848 | 0.9125 | 22 | 0.06 | 0.7150 | 0.8800 |
|  | 8 Emotional numbness | D | 103 | -0.09 | 0.1606 | 0.4240 | 74 | -0.04 | 0.6467 | 0.8395 | 22 | 0.05 | 0.7376 | 0.8895 |
|  | 9 Sense of failure | D | 105 | -0.11 | 0.0977 | 0.3339 | 74 | -0.20 | 0.0112 | 0.1040 | 23 | 0.05 | 0.7281 | 0.8850 |
|  | 10 Self-doubt | D | 105 | -0.16 | 0.0127 | 0.1096 | 73 | -0.18 | 0.0258 | 0.1638 | 23 | 0.32 | 0.0346 | 0.1870 |
|  | 11 Disconnection to others | D | 104 | -0.02 | 0.7248 | 0.8841 | 74 | -0.17 | 0.0336 | 0.1848 | 23 | 0.13 | 0.3678 | 0.6400 |
|  | 12 Social difficulty | D | 105 | -0.08 | 0.2559 | 0.5333 | 73 | -0.03 | 0.7201 | 0.8824 | 23 | 0.34 | 0.0247 | 0.1602 |
| 20 School fun | 1 Bad dreams | C | 104 | -0.07 | 0.3062 | 0.5827 | 73 | 0.06 | 0.4492 | 0.7080 | 23 | 0.16 | 0.2724 | 0.5496 |
|  | 2 Reliving events in mind | C | 103 | -0.12 | 0.0658 | 0.2690 | 73 | -0.08 | 0.3128 | 0.5901 | 23 | -0.22 | 0.1497 | 0.4074 |
|  | 3 Avoiding thoughts | C | 101 | -0.11 | 0.1021 | 0.3421 | 72 | 0.04 | 0.6105 | 0.8209 | 23 | -0.02 | 0.8795 | 0.9497 |
|  | 4 Avoiding physically | C | 102 | -0.15 | 0.0210 | 0.1464 | 71 | 0.12 | 0.1363 | 0.3921 | 23 | 0.17 | 0.2528 | 0.5319 |
|  | 5 Overly cautious | C | 102 | -0.19 | 0.0042 | 0.0552 | 71 | -0.12 | 0.1244 | 0.3751 | 23 | -0.05 | 0.7147 | 0.8800 |
|  | 6 Nervousness | C | 104 | -0.30 | <0.0001 | 0.0012 | 72 | -0.07 | 0.4000 | 0.6672 | 23 | -0.19 | 0.1947 | 0.4646 |
|  | 7 Calming difficulty | C | 103 | -0.21 | 0.0017 | 0.0310 | 72 | -0.01 | 0.9291 | 0.9740 | 22 | 0.33 | 0.0315 | 0.1787 |
|  | 8 Emotional numbness | C | 102 | -0.18 | 0.0073 | 0.0795 | 72 | 0.04 | 0.6195 | 0.8274 | 22 | -0.01 | 0.9678 | 0.9926 |
|  | 9 Sense of failure | C | 104 | -0.30 | <0.0001 | 0.0012 | 71 | -0.06 | 0.4664 | 0.7237 | 23 | 0.26 | 0.0820 | 0.3025 |
|  | 10 Self-doubt | C | 104 | -0.16 | 0.0174 | 0.1309 | 71 | -0.14 | 0.0748 | 0.2895 | 23 | 0.19 | 0.2092 | 0.4842 |
|  | 11 Disconnection to others | C | 103 | -0.19 | 0.0042 | 0.0553 | 72 | 0.01 | 0.9239 | 0.9720 | 23 | 0.06 | 0.6733 | 0.8553 |
|  | 12 Social difficulty | C | 104 | -0.15 | 0.0241 | 0.1594 | 71 | -0.04 | 0.6606 | 0.8487 | 23 | 0.10 | 0.5094 | 0.7515 |
| 21 Friends | 1 Bad dreams | D | 104 | 0.02 | 0.8065 | 0.9222 | 77 | 0.04 | 0.6435 | 0.8391 | 23 | 0.15 | 0.3304 | 0.6074 |
|  | 2 Reliving events in mind | D | 103 | -0.08 | 0.2251 | 0.4988 | 77 | -0.13 | 0.0864 | 0.3122 | 23 | -0.33 | 0.0254 | 0.1628 |
|  | 3 Avoiding thoughts | D | 101 | -0.05 | 0.4827 | 0.7368 | 76 | 0.13 | 0.1100 | 0.3552 | 23 | 0.23 | 0.1166 | 0.3656 |
|  | 4 Avoiding physically | D | 102 | -0.01 | 0.8928 | 0.9574 | 75 | 0.12 | 0.1273 | 0.3800 | 23 | -0.14 | 0.3439 | 0.6188 |
|  | 5 Overly cautious | D | 102 | -0.05 | 0.4710 | 0.7280 | 75 | -0.09 | 0.2401 | 0.5170 | 23 | -0.07 | 0.6438 | 0.8391 |
|  | 6 Nervousness | D | 104 | -0.14 | 0.0338 | 0.1853 | 76 | -0.09 | 0.2736 | 0.5496 | 23 | -0.30 | 0.0482 | 0.2250 |
|  | 7 Calming difficulty | D | 103 | -0.11 | 0.0956 | 0.3301 | 76 | -0.03 | 0.7470 | 0.8928 | 22 | -0.17 | 0.2668 | 0.5470 |
|  | 8 Emotional numbness | D | 102 | -0.10 | 0.1474 | 0.4054 | 76 | -0.03 | 0.6960 | 0.8692 | 22 | -0.09 | 0.5590 | 0.7849 |
|  | 9 Sense of failure | D | 104 | -0.10 | 0.1362 | 0.3919 | 75 | -0.13 | 0.0905 | 0.3215 | 23 | -0.03 | 0.8449 | 0.9342 |
|  | 10 Self-doubt | D | 104 | -0.04 | 0.5338 | 0.7697 | 75 | -0.17 | 0.0288 | 0.1726 | 23 | 0.15 | 0.3022 | 0.5799 |
|  | 11 Disconnection to others | D | 103 | -0.03 | 0.6269 | 0.8320 | 76 | 0.07 | 0.3760 | 0.6477 | 23 | -0.23 | 0.1318 | 0.3850 |
|  | 12 Social difficulty | D | 104 | -0.03 | 0.6732 | 0.8553 | 75 | 0.08 | 0.3203 | 0.5963 | 23 | -0.07 | 0.6220 | 0.8288 |
| 22 Dealing with school tasks | 1 Bad dreams | C | 104 | -0.02 | 0.7754 | 0.9067 | 73 | 0.14 | 0.0822 | 0.3029 | 23 | 0.17 | 0.2607 | 0.5372 |
|  | 2 Reliving events in mind | C | 103 | 0.02 | 0.7469 | 0.8928 | 73 | 0.06 | 0.4771 | 0.7329 | 23 | -0.05 | 0.7266 | 0.8841 |
|  | 3 Avoiding thoughts | C | 101 | 0.08 | 0.2519 | 0.5314 | 72 | 0.24 | 0.0031 | 0.0455 | 23 | 0.13 | 0.3715 | 0.6434 |
|  | 4 Avoiding physically | C | 102 | 0.01 | 0.8528 | 0.9377 | 71 | 0.35 | <0.0001 | 0.0020 | 23 | 0.09 | 0.5319 | 0.7689 |
|  | 5 Overly cautious | C | 102 | 0.03 | 0.6827 | 0.8604 | 71 | -0.01 | 0.8732 | 0.9471 | 23 | 0.04 | 0.8027 | 0.9204 |
|  | 6 Nervousness | C | 104 | -0.06 | 0.3846 | 0.6551 | 72 | 0.08 | 0.3373 | 0.6134 | 23 | -0.34 | 0.0221 | 0.1518 |
|  | 7 Calming difficulty | C | 103 | 0.00 | 0.9492 | 0.9833 | 72 | 0.22 | 0.0062 | 0.0713 | 22 | -0.05 | 0.7438 | 0.8912 |
|  | 8 Emotional numbness | C | 102 | 0.07 | 0.3318 | 0.6079 | 72 | 0.13 | 0.1028 | 0.3431 | 22 | -0.02 | 0.9010 | 0.9613 |
|  | 9 Sense of failure | C | 104 | -0.01 | 0.8423 | 0.9329 | 71 | 0.10 | 0.2035 | 0.4775 | 23 | -0.02 | 0.9075 | 0.9652 |
|  | 10 Self-doubt | C | 104 | -0.12 | 0.0801 | 0.2987 | 71 | 0.07 | 0.3980 | 0.6658 | 23 | 0.19 | 0.2116 | 0.4861 |
|  | 11 Disconnection to others | C | 103 | 0.00 | 0.9424 | 0.9795 | 72 | 0.11 | 0.1571 | 0.4181 | 23 | -0.08 | 0.5824 | 0.8009 |
|  | 12 Social difficulty | C | 104 | 0.06 | 0.3436 | 0.6186 | 71 | 0.13 | 0.1187 | 0.3686 | 23 | -0.09 | 0.5478 | 0.7772 |
| 23 Self-comparison to others | 1 Bad dreams | C | 104 | -0.08 | 0.2545 | 0.5324 | 79 | 0.04 | 0.6465 | 0.8395 | 23 | -0.08 | 0.5750 | 0.7955 |
|  | 2 Reliving events in mind | C | 103 | -0.05 | 0.4529 | 0.7117 | 79 | -0.12 | 0.1325 | 0.3857 | 23 | -0.34 | 0.0247 | 0.1602 |
|  | 3 Avoiding thoughts | C | 101 | 0.00 | 0.9690 | 0.9929 | 78 | 0.08 | 0.3137 | 0.5903 | 23 | -0.07 | 0.6421 | 0.8379 |
|  | 4 Avoiding physically | C | 102 | -0.03 | 0.6683 | 0.8529 | 77 | 0.02 | 0.8326 | 0.9308 | 23 | -0.06 | 0.6970 | 0.8702 |
|  | 5 Overly cautious | C | 102 | 0.01 | 0.8679 | 0.9447 | 77 | 0.01 | 0.8566 | 0.9402 | 23 | -0.27 | 0.0743 | 0.2892 |
|  | 6 Nervousness | C | 104 | -0.05 | 0.4967 | 0.7437 | 78 | -0.06 | 0.4471 | 0.7064 | 23 | -0.21 | 0.1512 | 0.4095 |
|  | 7 Calming difficulty | C | 103 | -0.03 | 0.6688 | 0.8530 | 78 | -0.04 | 0.6155 | 0.8243 | 22 | 0.11 | 0.4904 | 0.7409 |
|  | 8 Emotional numbness | C | 102 | 0.03 | 0.6744 | 0.8556 | 78 | -0.12 | 0.1106 | 0.3559 | 22 | -0.10 | 0.5299 | 0.7684 |
|  | 9 Sense of failure | C | 104 | -0.11 | 0.1126 | 0.3588 | 77 | -0.09 | 0.2619 | 0.5393 | 23 | -0.01 | 0.9692 | 0.9929 |
|  | 10 Self-doubt | C | 104 | -0.06 | 0.3490 | 0.6235 | 77 | -0.11 | 0.1759 | 0.4418 | 23 | 0.03 | 0.8209 | 0.9290 |
|  | 11 Disconnection to others | C | 103 | -0.02 | 0.8049 | 0.9214 | 78 | -0.03 | 0.6803 | 0.8596 | 23 | 0.01 | 0.9376 | 0.9762 |
|  | 12 Social difficulty | C | 104 | 0.06 | 0.3885 | 0.6574 | 77 | 0.07 | 0.3460 | 0.6210 | 23 | 0.15 | 0.3120 | 0.5892 |
| 24 Love-awareness | 1 Bad dreams | B | 104 | -0.25 | 0.0001 | 0.0064 | 80 | 0.12 | 0.1228 | 0.3734 | 23 | 0.21 | 0.1570 | 0.4180 |
|  | 2 Reliving events in mind | B | 103 | -0.10 | 0.1345 | 0.3898 | 80 | -0.08 | 0.2845 | 0.5587 | 23 | 0.06 | 0.7037 | 0.8749 |
|  | 3 Avoiding thoughts | B | 101 | -0.15 | 0.0260 | 0.1643 | 79 | 0.05 | 0.4875 | 0.7389 | 23 | 0.05 | 0.7322 | 0.8872 |
|  | 4 Avoiding physically | B | 102 | -0.04 | 0.5352 | 0.7703 | 78 | 0.11 | 0.1489 | 0.4061 | 23 | 0.10 | 0.4913 | 0.7416 |
|  | 5 Overly cautious | B | 102 | -0.09 | 0.1824 | 0.4502 | 78 | 0.05 | 0.5502 | 0.7791 | 23 | 0.02 | 0.8705 | 0.9460 |
|  | 6 Nervousness | B | 104 | -0.16 | 0.0186 | 0.1362 | 79 | -0.01 | 0.8887 | 0.9546 | 23 | -0.24 | 0.1119 | 0.3582 |
|  | 7 Calming difficulty | B | 103 | -0.11 | 0.0940 | 0.3269 | 79 | 0.06 | 0.4524 | 0.7112 | 22 | 0.04 | 0.8064 | 0.9222 |
|  | 8 Emotional numbness | B | 102 | -0.07 | 0.2938 | 0.5691 | 79 | -0.05 | 0.5337 | 0.7697 | 22 | -0.13 | 0.3920 | 0.6605 |
|  | 9 Sense of failure | B | 104 | -0.21 | 0.0020 | 0.0349 | 78 | -0.06 | 0.4175 | 0.6826 | 23 | 0.05 | 0.7329 | 0.8877 |
|  | 10 Self-doubt | B | 104 | -0.18 | 0.0067 | 0.0747 | 78 | -0.05 | 0.5163 | 0.7574 | 23 | 0.12 | 0.4366 | 0.6983 |
|  | 11 Disconnection to others | B | 103 | -0.13 | 0.0592 | 0.2527 | 79 | 0.06 | 0.4714 | 0.7281 | 23 | -0.01 | 0.9693 | 0.9929 |
|  | 12 Social difficulty | B | 104 | -0.05 | 0.4394 | 0.6995 | 78 | 0.02 | 0.7759 | 0.9070 | 23 | 0.08 | 0.5813 | 0.8001 |
| 25 Peer arguing | 1 Bad dreams | D | 104 | -0.08 | 0.2539 | 0.5324 | 80 | -0.04 | 0.5754 | 0.7955 | 23 | 0.19 | 0.2099 | 0.4847 |
|  | 2 Reliving events in mind | D | 103 | -0.09 | 0.1920 | 0.4616 | 80 | -0.10 | 0.1882 | 0.4571 | 23 | -0.22 | 0.1428 | 0.3993 |
|  | 3 Avoiding thoughts | D | 101 | -0.09 | 0.2068 | 0.4808 | 79 | 0.08 | 0.2806 | 0.5565 | 23 | 0.45 | 0.0024 | 0.0392 |
|  | 4 Avoiding physically | D | 102 | -0.05 | 0.4930 | 0.7423 | 78 | -0.02 | 0.7598 | 0.8988 | 23 | 0.27 | 0.0674 | 0.2729 |
|  | 5 Overly cautious | D | 102 | -0.12 | 0.0791 | 0.2977 | 78 | -0.15 | 0.0519 | 0.2354 | 23 | 0.37 | 0.0127 | 0.1096 |
|  | 6 Nervousness | D | 104 | -0.14 | 0.0328 | 0.1824 | 79 | -0.17 | 0.0236 | 0.1579 | 23 | -0.18 | 0.2300 | 0.5056 |
|  | 7 Calming difficulty | D | 103 | -0.04 | 0.5343 | 0.7700 | 79 | 0.02 | 0.7975 | 0.9192 | 22 | 0.17 | 0.2703 | 0.5474 |
|  | 8 Emotional numbness | D | 102 | -0.13 | 0.0541 | 0.2394 | 79 | -0.11 | 0.1708 | 0.4373 | 22 | 0.08 | 0.6015 | 0.8158 |
|  | 9 Sense of failure | D | 104 | -0.18 | 0.0073 | 0.0795 | 78 | -0.04 | 0.6175 | 0.8257 | 23 | 0.26 | 0.0866 | 0.3127 |
|  | 10 Self-doubt | D | 104 | -0.16 | 0.0164 | 0.1277 | 78 | -0.11 | 0.1588 | 0.4214 | 23 | 0.31 | 0.0387 | 0.2000 |
|  | 11 Disconnection to others | D | 103 | -0.12 | 0.0739 | 0.2882 | 79 | 0.04 | 0.6146 | 0.8243 | 23 | 0.00 | 1.0000 | 1.0000 |
|  | 12 Social difficulty | D | 104 | -0.07 | 0.2682 | 0.5470 | 78 | 0.04 | 0.6181 | 0.8260 | 23 | 0.39 | 0.0083 | 0.0855 |
| 26 Napping/dozing | 1 Bad dreams | A | 105 | -0.14 | 0.0333 | 0.1844 | 79 | 0.11 | 0.1684 | 0.4353 | 23 | 0.17 | 0.2592 | 0.5368 |
|  | 2 Reliving events in mind | A | 104 | -0.07 | 0.2750 | 0.5508 | 79 | -0.01 | 0.8599 | 0.9402 | 23 | -0.12 | 0.4069 | 0.6715 |
|  | 3 Avoiding thoughts | A | 102 | -0.17 | 0.0124 | 0.1089 | 78 | 0.08 | 0.3031 | 0.5804 | 23 | 0.21 | 0.1625 | 0.4258 |
|  | 4 Avoiding physically | A | 103 | -0.06 | 0.3829 | 0.6534 | 77 | 0.18 | 0.0191 | 0.1375 | 23 | 0.09 | 0.5390 | 0.7728 |
|  | 5 Overly cautious | A | 103 | -0.11 | 0.1033 | 0.3438 | 77 | 0.10 | 0.1925 | 0.4624 | 23 | -0.05 | 0.7434 | 0.8911 |
|  | 6 Nervousness | A | 105 | -0.14 | 0.0340 | 0.1860 | 78 | -0.02 | 0.7635 | 0.9000 | 23 | -0.14 | 0.3401 | 0.6157 |
|  | 7 Calming difficulty | A | 104 | -0.06 | 0.3651 | 0.6379 | 78 | 0.09 | 0.2366 | 0.5134 | 22 | -0.01 | 0.9254 | 0.9720 |
|  | 8 Emotional numbness | A | 103 | -0.05 | 0.4678 | 0.7245 | 78 | 0.06 | 0.4696 | 0.7261 | 22 | -0.37 | 0.0154 | 0.1237 |
|  | 9 Sense of failure | A | 105 | -0.20 | 0.0032 | 0.0457 | 77 | -0.02 | 0.7799 | 0.9102 | 23 | -0.11 | 0.4593 | 0.7174 |
|  | 10 Self-doubt | A | 105 | -0.15 | 0.0225 | 0.1534 | 77 | -0.08 | 0.3137 | 0.5903 | 23 | 0.08 | 0.5804 | 0.7994 |
|  | 11 Disconnection to others | A | 104 | -0.05 | 0.4443 | 0.7041 | 78 | -0.06 | 0.4099 | 0.6739 | 23 | -0.20 | 0.1714 | 0.4379 |
|  | 12 Social difficulty | A | 105 | -0.06 | 0.4046 | 0.6699 | 77 | 0.07 | 0.3861 | 0.6557 | 23 | 0.24 | 0.1088 | 0.3532 |
| 27 Eating problems | 1 Bad dreams | A | 101 | 0.00 | 0.9773 | 0.9986 | 80 | 0.03 | 0.6907 | 0.8660 | 23 | -0.20 | 0.1798 | 0.4466 |
|  | 2 Reliving events in mind | A | 101 | 0.09 | 0.1955 | 0.4653 | 80 | -0.10 | 0.1880 | 0.4570 | 23 | -0.23 | 0.1234 | 0.3736 |
|  | 3 Avoiding thoughts | A | 99 | 0.03 | 0.6524 | 0.8429 | 79 | 0.17 | 0.0279 | 0.1697 | 23 | -0.12 | 0.4411 | 0.7012 |
|  | 4 Avoiding physically | A | 100 | 0.06 | 0.3525 | 0.6268 | 78 | 0.15 | 0.0475 | 0.2241 | 23 | -0.20 | 0.1920 | 0.4616 |
|  | 5 Overly cautious | A | 100 | 0.05 | 0.5010 | 0.7458 | 78 | 0.05 | 0.5523 | 0.7803 | 23 | -0.42 | 0.0047 | 0.0593 |
|  | 6 Nervousness | A | 101 | -0.05 | 0.4733 | 0.7298 | 79 | 0.02 | 0.8231 | 0.9290 | 23 | -0.46 | 0.0021 | 0.0355 |
|  | 7 Calming difficulty | A | 100 | 0.00 | 0.9618 | 0.9898 | 79 | 0.07 | 0.3685 | 0.6400 | 22 | 0.06 | 0.6936 | 0.8675 |
|  | 8 Emotional numbness | A | 99 | 0.04 | 0.5394 | 0.7729 | 79 | 0.01 | 0.8747 | 0.9478 | 22 | -0.09 | 0.5478 | 0.7772 |
|  | 9 Sense of failure | A | 101 | -0.15 | 0.0261 | 0.1643 | 78 | 0.07 | 0.3924 | 0.6605 | 23 | 0.01 | 0.9677 | 0.9926 |
|  | 10 Self-doubt | A | 101 | -0.15 | 0.0246 | 0.1602 | 78 | 0.04 | 0.6096 | 0.8203 | 23 | -0.19 | 0.2062 | 0.4804 |
|  | 11 Disconnection to others | A | 100 | -0.10 | 0.1487 | 0.4061 | 79 | 0.11 | 0.1596 | 0.4224 | 23 | -0.10 | 0.5119 | 0.7538 |
|  | 12 Social difficulty | A | 101 | 0.03 | 0.7003 | 0.8723 | 78 | 0.17 | 0.0313 | 0.1782 | 23 | 0.06 | 0.6663 | 0.8526 |
| 28 Memorization | 1 Bad dreams | C | 105 | -0.19 | 0.0051 | 0.0625 | 80 | 0.07 | 0.3574 | 0.6314 | 23 | 0.12 | 0.4129 | 0.6770 |
|  | 2 Reliving events in mind | C | 104 | -0.06 | 0.3347 | 0.6109 | 80 | -0.07 | 0.3453 | 0.6205 | 23 | -0.17 | 0.2526 | 0.5318 |
|  | 3 Avoiding thoughts | C | 102 | -0.13 | 0.0542 | 0.2394 | 79 | 0.09 | 0.2672 | 0.5470 | 23 | 0.21 | 0.1592 | 0.4218 |
|  | 4 Avoiding physically | C | 103 | -0.06 | 0.3854 | 0.6552 | 78 | 0.11 | 0.1482 | 0.4061 | 23 | 0.05 | 0.7568 | 0.8969 |
|  | 5 Overly cautious | C | 103 | -0.09 | 0.1940 | 0.4646 | 78 | 0.02 | 0.7722 | 0.9053 | 23 | 0.05 | 0.7414 | 0.8903 |
|  | 6 Nervousness | C | 105 | -0.12 | 0.0775 | 0.2952 | 79 | 0.02 | 0.7737 | 0.9057 | 23 | -0.13 | 0.4031 | 0.6689 |
|  | 7 Calming difficulty | C | 104 | -0.09 | 0.1681 | 0.4348 | 79 | 0.21 | 0.0072 | 0.0787 | 22 | 0.22 | 0.1429 | 0.3993 |
|  | 8 Emotional numbness | C | 103 | -0.05 | 0.4510 | 0.7098 | 79 | 0.08 | 0.3192 | 0.5951 | 22 | 0.21 | 0.1779 | 0.4446 |
|  | 9 Sense of failure | C | 105 | -0.10 | 0.1278 | 0.3804 | 78 | -0.04 | 0.6018 | 0.8158 | 23 | 0.20 | 0.1737 | 0.4408 |
|  | 10 Self-doubt | C | 105 | -0.12 | 0.0611 | 0.2567 | 78 | -0.14 | 0.0619 | 0.2592 | 23 | 0.37 | 0.0146 | 0.1189 |
|  | 11 Disconnection to others | C | 104 | -0.12 | 0.0660 | 0.2698 | 79 | -0.02 | 0.8416 | 0.9329 | 23 | 0.01 | 0.9645 | 0.9912 |
|  | 12 Social difficulty | C | 105 | -0.15 | 0.0194 | 0.1384 | 78 | 0.02 | 0.8177 | 0.9281 | 23 | 0.50 | 0.0009 | 0.0212 |

*B-H* – Benjamini-Hochberg correction for multiple comparisons, *p*-values < 0.05 are indicated in red, N – number of participants, Tau – Tau correlation coefficient
