# Supplementary material for: Demographic predictors of trauma and depression in war-affected children from Poland and Ukraine: Implications for prevention of mental health problems
Source: Prev Med Rep. 2026 Feb 20;63:103422. doi: 10.1016/j.pmedr.2026.103422 (PMC12945576; doi:10.1016/j.pmedr.2026.103422)
Supplement: Supplementary file 3 — Supplementary material 3 [file mmc3.docx]

**CDI (panels A-D) and ITQ-CA correlations in war-witnessing dependent groups**

**CDI2 – group A**

In the group of children who were not war witnesses, 33 significant correlations in CDI2–group A were observed (Figure S3). Most significant positive, very weak, or weak correlations were observed between CDI2-1 “Sadness” with 10 ITQ-CA categories or CDI2-27 “Eating problems” with 4 ITQ-CA categories. The remaining 20 correlations were negative and weak or very weak (Table S3). Most negative correlations were observed between CDI2-18 “Pain thoughts” and 11 ITQ-CA categories. After Benjamini-Hochberg correction, in the group of no-war witnesses 14 significant correlations remains (Table S3) – 6 positive between CDI2-1 “Sadness” with ITQ-CA (Avoiding thoughts”, “Calming difficulty”, “Emotional numbness”, and “Self-doubt” and CDI2-27 “Eating problems” with ITQ-CA “Social difficulty”), and eight negative correlations (between CDI2-10 “Bad mood” with ITQ-CA “Nervousness”, CDI2-17 “Eating problems” with ITQ-CA “Nervousness” and “Sense of failure”, CDI2-18 “Pain thoughts” with ITQ-CA “Bad dreams”, “Relieving events in mind”, “Avoiding thoughts”, “Overlay cautions” and “Nervousness”).

Among the war-witnesses in the group of children who did not witness war, we observed 13 significant correlations, all very weak or weak (Figure S3). One correlation was positive between CDI2-1 “Sadness” with ITQ-CA “Sense of failure”. Other correlations were negative, and most of them were between CDI2-15 “Sleep quality” with ITQ-CA “Nervousness”, “Calming difficulty”, “Sense of failure”, and “Disconnection to others”. After Benjamini-Hochberg correction, in the group of war witnesses, three significant correlations remain (Table S3), between CDI2-9 “Tendency to cry” with ITQ-CA “Relieving events in mind”, CDI2-16 “Sleep quality” with ITQ-CA “Nervousness”, and between CDI2-17 “Eating attitude” with ITQ-CA “Social difficulty” (Table S3).

**CDI2 – group B**

In the CDI2–group B, 33 significant correlations were observed among participants who had not experienced war (Figure S3). All of them were negative, weak to moderate in strength. Most significant correlations between CDI2-13 “Self-perception” with 10 ITQ-CA categories, CDI2-7 “Blame” with 8 ITQ-CA categories, CDI2-2 “Hopeful” and CDI-24 “Love awareness”, both with 5 ITQ-CA categories. Others were CDI2-6 “Self-acceptance” and CDI2-8 “Suicide” with ITQ-CA 2 and 3 categories, respectively. After Benjamini-Hochberg correction, 15 correlations remain significant (between CDI2-13 “Self-perception” and 7 ITQ-CA categories, CDI2-24 “Love awareness” with 4 ITQ-CA categories, and CDI2-2 “Hopeful” and CDI2-7 “Blame” with 2 ITQ-CA categories (Table S3). Six significant weak correlations were observed in the respondents' group who were war witnesses. Two were negative (between CDI2-2 “Hopeful” and CDI2-7 “Blame” with ITQ-CA “Overlay cautions” and “Sense of failure”, respectively. The four positive correlations were between CDI2-8 “Suicide” with ITQ-CA “Social difficulty” and CDI2-13 “Self-perception” with ITQ-CA “Bad dreams”, Avoiding physically” and “Self-doubt”. None of them remains significant after Benjamini-Hochberg correction (Table S3).

**CDI2 – group C**

In the CDI2 – group C children who did not experience war, 34 significant correlations (very weak and weak) were observed (Figure S3), 32 negative and two positive. The positive correlations were observed between CDI2-14 “Learning attitude” with ITQ-CA “Sense of failure” and “Disconnection to others”. Most of the positive correlations were observed between CDI2-20 “School fun”, CDI2-28 “Memorization”, CDI2-12 “Self-determination”, CDI2-3 “Self-confidence”, and CDI2-4 “Enjoyment” with ITQ-CA 9, 7, 5, and also five categories, respectively. After Benjamini-Hochberg correction, 11 negative correlations remains significant between CDI2-3 “Self-confidence” with ITQ-CA “Nervousness” and “Sense of failure”, “CDI2-4 “Enjoyment” and “Nervousness”, CDI2-12 “Self-determination” with ITQ-CA “Overlay caution” and “Nervousness”, CDI2-20 “School fun” and ITQ-CA “Overlay caution”, “Nervousness”, “Emotional numbness”, “Sense of failure”, and “Self-doubt”, and finally CDI2-28 “Memorization” with ITQ-CA “Self-doubt” (Table S3). Among the children who experienced war, 7 significant and weak correlation were observed (Figure S3). The CDI2-14 “Learning attitude” was negatively correlated with ITQ-CA “Disconnection to others”. The others correlations were positive (between CDI2-4 “Enjoyment” with ITQ-CA “Bad dreams”, “Avoiding physically”, CDI2-22 “Dealing with school tasks” with ITQ-CA “Bad dreams”, “Avoiding thoughts” and “Avoiding physically”, and CDI2-28 “Memorization” with ITQ-CA “Calming difficulty”. After Benjamini-Hochberg correction, one positive correlation remain significant between CDI2-22 “Dealing with school tasks” with ITQ-CA “avoiding physically” (Table S3).

**CDI2 – group D**

Among the children who did not witness the war, 22 significant correlations were observed in CDI2–group D (Figure S3). All the correlations were negative and very weak or weak. Most are between CDI2-5 “Importance for family” with 10 ITQ-CA categories and CDI2-19 “Peer arguing” with 8 ITQ-CA categories. After Benjamini-Hochberg correction, 11 correlations remains significant between CDI2-5 “Importance for family” with ITQ-CA “Bad dreams”, “Relieving events in mind”, “Overlay cautions”, “Emotional numbness”, “Sence of failure”, “Self-doubt” and “Disconnection to others”, CDI2-11 “Company” with ITQ-CA “Sense of failure”, and CDI2-25 “Peer arguing” with ITQ-CA “Emotional numbness”, “Sense of failure”, and “Self-doubt”. In the children who witnessed war, we observed three significant and weak correlations, negative between CDI2-21 “Friends” with ITQ-CA “Relieving events in mind”, and positive CDI2-5 “Importance for family” and CDI2-25 “Peer arguing” with ITQ-CA “Disconnection to others” and “Social difficulty”, respectively. The last one remains significant after correction for multiple comparisons (Table S3).


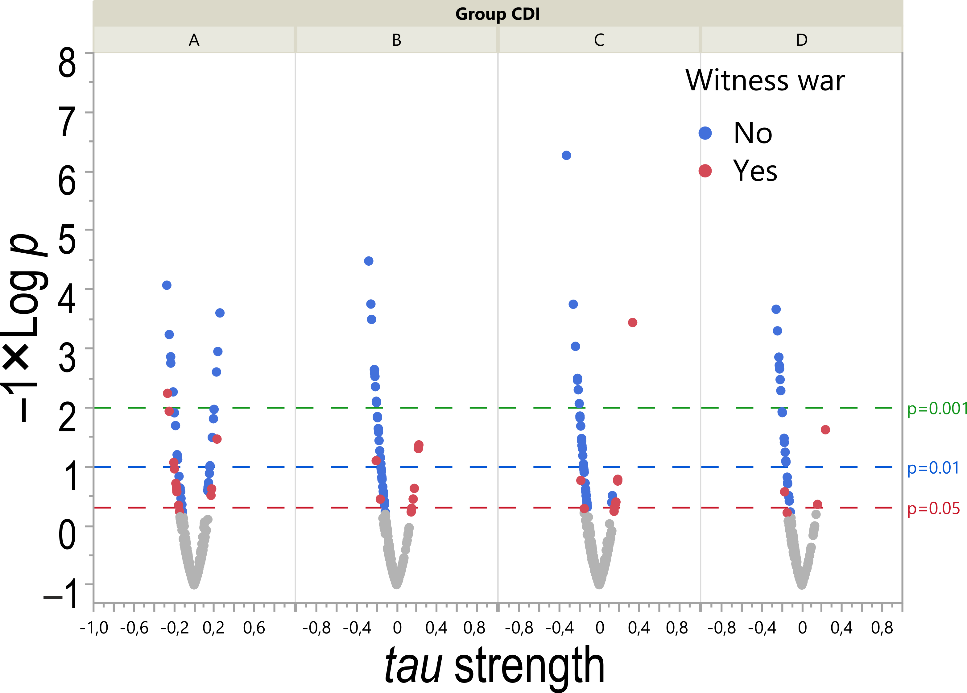


**Figure S3.** Correlations between single questions CDI-2 (Children's Depression Inventory 2) and ITQ-CA (International Trauma Questionnaire – Child and Adolescent Version) in war-witness dependent groups of children and adolescents from Poland and Ukraine. Volcano plot of significant correlations between single questions of CDI and ITQ-CA in the CDI A, B, C, and D groups. The strength of *tau* correlation coefficient (x-axis) and the significance as the unadjusted *p*-values (shown as −1×log *p*; y-axis). The dashed horizontal lines represent the *p* = 0.05 (red), *p* = 0.01 (blue), and *p* = 0.001 (green). Significant correlations are shown as colored spots, and non-significant correlations are grey, March 2024-March 2025, Poland and Ukraine.

**Table S3.** Children's Depression Inventory 2 (CDI-2) (panels A-D) and International Trauma Questionnaire – Child and Adolescent Version (ITQ-CA) correlations in war-witnessing dependent groups of children and adolescents from Poland and Ukraine, March 2024-March 2025, Poland and Ukraine.

| **CDI2** | **ITQ-CA** | **Group CDI** | **N** | ***Tau*** | ***p*-value** | ***p*-value ^BH^** | **N** | ***Tau*** | ***p*-value** | ***p*-value ^BH^** |
| --- | --- | --- | --- | --- | --- | --- | --- | --- | --- | --- |
|  |  | **Witness war - NO** | | | | | **Witness war - YES** | | | |
| 1 Sadness | 1 Bad dreams | A | 124 | 0.16 | 0.0101 | 0.0967 | 78 | -0.03 | 0.6562 | 0.8449 |
|  | 2 Reliving events in mind | A | 123 | 0.10 | 0.0858 | 0.3116 | 78 | -0.13 | 0.0878 | 0.3160 |
|  | 3 Avoiding thoughts | A | 122 | 0.24 | 0.0001 | 0.0055 | 77 | 0.09 | 0.2572 | 0.5349 |
|  | 4 Avoiding physically | A | 121 | 0.14 | 0.0197 | 0.1395 | 77 | 0.03 | 0.7071 | 0.8765 |
|  | 5 Overly cautious | A | 122 | 0.15 | 0.0129 | 0.1106 | 76 | -0.06 | 0.4681 | 0.7246 |
|  | 6 Nervousness | A | 124 | 0.14 | 0.0221 | 0.1519 | 77 | 0.03 | 0.6988 | 0.8711 |
|  | 7 Calming difficulty | A | 123 | 0.26 | <0.0001 | 0.0022 | 76 | 0.03 | 0.6954 | 0.8688 |
|  | 8 Emotional numbness | A | 122 | 0.18 | 0.0032 | 0.0457 | 77 | 0.09 | 0.2731 | 0.5496 |
|  | 9 Sense of failure | A | 123 | 0.14 | 0.0185 | 0.1359 | 77 | 0.17 | 0.0305 | 0.1771 |
|  | 10 Self-doubt | A | 124 | 0.20 | 0.0011 | 0.0235 | 76 | 0.08 | 0.2949 | 0.5705 |
|  | 11 Disconnection to others | A | 123 | 0.08 | 0.2010 | 0.4725 | 77 | 0.03 | 0.6816 | 0.8601 |
|  | 12 Social difficulty | A | 124 | 0.22 | 0.0002 | 0.0092 | 76 | 0.10 | 0.2147 | 0.4875 |
| 2 Hopeful | 1 Bad dreams | B | 128 | -0.11 | 0.0676 | 0.2732 | 80 | -0.02 | 0.7919 | 0.9160 |
|  | 2 Reliving events in mind | B | 127 | -0.15 | 0.0119 | 0.1067 | 80 | -0.11 | 0.1350 | 0.3900 |
|  | 3 Avoiding thoughts | B | 125 | -0.07 | 0.2736 | 0.5496 | 79 | -0.05 | 0.5258 | 0.7646 |
|  | 4 Avoiding physically | B | 125 | -0.04 | 0.5353 | 0.7703 | 79 | 0.12 | 0.1070 | 0.3506 |
|  | 5 Overly cautious | B | 126 | -0.13 | 0.0261 | 0.1643 | 78 | -0.16 | 0.0352 | 0.1890 |
|  | 6 Nervousness | B | 128 | -0.22 | 0.0003 | 0.0102 | 79 | -0.14 | 0.0744 | 0.2892 |
|  | 7 Calming difficulty | B | 127 | -0.10 | 0.1123 | 0.3588 | 78 | 0.01 | 0.8483 | 0.9350 |
|  | 8 Emotional numbness | B | 126 | -0.07 | 0.2192 | 0.4926 | 78 | 0.00 | 1.0000 | 1.0000 |
|  | 9 Sense of failure | B | 127 | -0.19 | 0.0014 | 0.0282 | 79 | -0.08 | 0.3275 | 0.6038 |
|  | 10 Self-doubt | B | 128 | -0.16 | 0.0070 | 0.0771 | 78 | -0.04 | 0.6279 | 0.8325 |
|  | 11 Disconnection to others | B | 127 | -0.08 | 0.2071 | 0.4808 | 79 | -0.12 | 0.1139 | 0.3613 |
|  | 12 Social difficulty | B | 128 | 0.03 | 0.6047 | 0.8171 | 78 | -0.12 | 0.1087 | 0.3531 |
| 3 Self-confidence | 1 Bad dreams | C | 128 | -0.02 | 0.7663 | 0.9022 | 80 | 0.05 | 0.5356 | 0.7703 |
|  | 2 Reliving events in mind | C | 127 | -0.11 | 0.0708 | 0.2803 | 80 | -0.04 | 0.6027 | 0.8164 |
|  | 3 Avoiding thoughts | C | 125 | -0.02 | 0.7359 | 0.8887 | 79 | 0.05 | 0.5466 | 0.7767 |
|  | 4 Avoiding physically | C | 125 | -0.04 | 0.5370 | 0.7716 | 79 | 0.08 | 0.2775 | 0.5528 |
|  | 5 Overly cautious | C | 126 | -0.13 | 0.0264 | 0.1643 | 78 | -0.03 | 0.7221 | 0.8832 |
|  | 6 Nervousness | C | 128 | -0.20 | 0.0009 | 0.0206 | 79 | -0.04 | 0.6174 | 0.8257 |
|  | 7 Calming difficulty | C | 127 | -0.07 | 0.2592 | 0.5368 | 78 | 0.08 | 0.2839 | 0.5586 |
|  | 8 Emotional numbness | C | 126 | -0.09 | 0.1207 | 0.3707 | 78 | 0.07 | 0.3735 | 0.6451 |
|  | 9 Sense of failure | C | 127 | -0.19 | 0.0014 | 0.0279 | 79 | 0.08 | 0.2875 | 0.5622 |
|  | 10 Self-doubt | C | 128 | -0.13 | 0.0238 | 0.1583 | 78 | -0.04 | 0.6035 | 0.8168 |
|  | 11 Disconnection to others | C | 127 | -0.14 | 0.0188 | 0.1369 | 79 | 0.05 | 0.4861 | 0.7382 |
|  | 12 Social difficulty | C | 128 | -0.08 | 0.1855 | 0.4536 | 78 | 0.15 | 0.0567 | 0.2460 |
| 4 Enjoyment | 1 Bad dreams | C | 127 | -0.12 | 0.0482 | 0.2250 | 80 | 0.15 | 0.0493 | 0.2284 |
|  | 2 Reliving events in mind | C | 126 | -0.04 | 0.5566 | 0.7819 | 80 | -0.03 | 0.7135 | 0.8796 |
|  | 3 Avoiding thoughts | C | 124 | 0.05 | 0.3848 | 0.6552 | 79 | 0.10 | 0.2151 | 0.4876 |
|  | 4 Avoiding physically | C | 124 | 0.03 | 0.5905 | 0.8070 | 79 | 0.18 | 0.0162 | 0.1274 |
|  | 5 Overly cautious | C | 125 | -0.15 | 0.0106 | 0.1007 | 78 | -0.01 | 0.8875 | 0.9537 |
|  | 6 Nervousness | C | 127 | -0.21 | 0.0005 | 0.0143 | 79 | -0.06 | 0.4014 | 0.6682 |
|  | 7 Calming difficulty | C | 126 | -0.08 | 0.1912 | 0.4604 | 78 | -0.01 | 0.8595 | 0.9402 |
|  | 8 Emotional numbness | C | 125 | -0.17 | 0.0044 | 0.0563 | 78 | -0.02 | 0.7860 | 0.9135 |
|  | 9 Sense of failure | C | 126 | -0.10 | 0.0991 | 0.3367 | 79 | -0.04 | 0.6214 | 0.8285 |
|  | 10 Self-doubt | C | 127 | -0.14 | 0.0237 | 0.1583 | 78 | 0.04 | 0.6375 | 0.8355 |
|  | 11 Disconnection to others | C | 126 | 0.04 | 0.5470 | 0.7769 | 79 | -0.05 | 0.5363 | 0.7711 |
|  | 12 Social difficulty | C | 127 | -0.07 | 0.2430 | 0.5207 | 78 | 0.04 | 0.6416 | 0.8375 |
| 5 Importance for family | 1 Bad dreams | D | 128 | -0.24 | 0.0001 | 0.0033 | 79 | 0.12 | 0.1235 | 0.3736 |
|  | 2 Reliving events in mind | D | 127 | -0.23 | 0.0001 | 0.0063 | 79 | -0.02 | 0.7885 | 0.9157 |
|  | 3 Avoiding thoughts | D | 125 | -0.06 | 0.3383 | 0.6138 | 78 | -0.05 | 0.4793 | 0.7354 |
|  | 4 Avoiding physically | D | 125 | -0.13 | 0.0307 | 0.1773 | 78 | 0.09 | 0.2345 | 0.5117 |
|  | 5 Overly cautious | D | 126 | -0.22 | 0.0003 | 0.0109 | 77 | 0.00 | 0.9760 | 0.9977 |
|  | 6 Nervousness | D | 128 | -0.16 | 0.0083 | 0.0853 | 78 | -0.03 | 0.7084 | 0.8769 |
|  | 7 Calming difficulty | D | 127 | -0.10 | 0.0879 | 0.3161 | 77 | 0.05 | 0.5512 | 0.7796 |
|  | 8 Emotional numbness | D | 126 | -0.21 | 0.0005 | 0.0145 | 77 | -0.12 | 0.1139 | 0.3613 |
|  | 9 Sense of failure | D | 127 | -0.19 | 0.0012 | 0.0254 | 78 | 0.00 | 0.9648 | 0.9912 |
|  | 10 Self-doubt | D | 128 | -0.19 | 0.0012 | 0.0255 | 77 | 0.05 | 0.5223 | 0.7614 |
|  | 11 Disconnection to others | D | 127 | -0.22 | 0.0002 | 0.0085 | 78 | 0.16 | 0.0432 | 0.2143 |
|  | 12 Social difficulty | D | 128 | -0.15 | 0.0150 | 0.1212 | 77 | 0.09 | 0.2340 | 0.5107 |
| 6 Self-acceptance | 1 Bad dreams | B | 127 | -0.09 | 0.1182 | 0.3679 | 80 | 0.11 | 0.1324 | 0.3857 |
|  | 2 Reliving events in mind | B | 126 | -0.10 | 0.0877 | 0.3160 | 80 | -0.02 | 0.8131 | 0.9261 |
|  | 3 Avoiding thoughts | B | 124 | -0.05 | 0.4507 | 0.7095 | 79 | 0.10 | 0.1952 | 0.4653 |
|  | 4 Avoiding physically | B | 124 | -0.11 | 0.0635 | 0.2636 | 79 | 0.12 | 0.1117 | 0.3579 |
|  | 5 Overly cautious | B | 125 | -0.07 | 0.2355 | 0.5123 | 78 | -0.03 | 0.7255 | 0.8841 |
|  | 6 Nervousness | B | 127 | -0.15 | 0.0114 | 0.1046 | 79 | -0.13 | 0.0936 | 0.3257 |
|  | 7 Calming difficulty | B | 126 | -0.07 | 0.2363 | 0.5132 | 78 | -0.12 | 0.1087 | 0.3531 |
|  | 8 Emotional numbness | B | 125 | -0.09 | 0.1499 | 0.4079 | 78 | 0.02 | 0.7898 | 0.9159 |
|  | 9 Sense of failure | B | 126 | -0.14 | 0.0218 | 0.1504 | 79 | -0.07 | 0.3596 | 0.6333 |
|  | 10 Self-doubt | B | 127 | -0.07 | 0.2326 | 0.5088 | 78 | 0.06 | 0.4063 | 0.6712 |
|  | 11 Disconnection to others | B | 126 | -0.10 | 0.1072 | 0.3506 | 79 | 0.01 | 0.8960 | 0.9594 |
|  | 12 Social difficulty | B | 127 | -0.02 | 0.7496 | 0.8934 | 78 | 0.04 | 0.5884 | 0.8059 |
| 7 Blame | 1 Bad dreams | B | 126 | -0.12 | 0.0439 | 0.2156 | 80 | 0.04 | 0.5776 | 0.7976 |
|  | 2 Reliving events in mind | B | 125 | -0.12 | 0.0426 | 0.2123 | 80 | -0.08 | 0.3028 | 0.5804 |
|  | 3 Avoiding thoughts | B | 123 | -0.04 | 0.5380 | 0.7725 | 79 | -0.09 | 0.2606 | 0.5372 |
|  | 4 Avoiding physically | B | 123 | -0.13 | 0.0396 | 0.2032 | 79 | 0.10 | 0.1755 | 0.4412 |
|  | 5 Overly cautious | B | 124 | -0.15 | 0.0164 | 0.1278 | 78 | 0.11 | 0.1432 | 0.3993 |
|  | 6 Nervousness | B | 126 | -0.21 | 0.0004 | 0.0132 | 79 | -0.09 | 0.2483 | 0.5265 |
|  | 7 Calming difficulty | B | 125 | -0.10 | 0.1073 | 0.3508 | 78 | -0.09 | 0.2380 | 0.5148 |
|  | 8 Emotional numbness | B | 124 | -0.10 | 0.0840 | 0.3072 | 78 | -0.05 | 0.4904 | 0.7409 |
|  | 9 Sense of failure | B | 125 | -0.11 | 0.0680 | 0.2740 | 79 | -0.20 | 0.0079 | 0.0826 |
|  | 10 Self-doubt | B | 126 | -0.13 | 0.0371 | 0.1950 | 78 | -0.05 | 0.4954 | 0.7436 |
|  | 11 Disconnection to others | B | 125 | -0.22 | 0.0003 | 0.0096 | 79 | -0.06 | 0.4336 | 0.6970 |
|  | 12 Social difficulty | B | 126 | -0.13 | 0.0298 | 0.1757 | 78 | -0.09 | 0.2466 | 0.5250 |
| 8 Suicide | 1 Bad dreams | B | 125 | -0.09 | 0.1339 | 0.3889 | 80 | 0.15 | 0.0503 | 0.2311 |
|  | 2 Reliving events in mind | B | 124 | -0.10 | 0.0927 | 0.3246 | 80 | -0.13 | 0.0970 | 0.3328 |
|  | 3 Avoiding thoughts | B | 122 | -0.01 | 0.8969 | 0.9595 | 79 | 0.06 | 0.4256 | 0.6905 |
|  | 4 Avoiding physically | B | 122 | -0.07 | 0.2841 | 0.5586 | 79 | 0.05 | 0.5536 | 0.7815 |
|  | 5 Overly cautious | B | 123 | -0.08 | 0.1832 | 0.4512 | 78 | -0.03 | 0.7218 | 0.8831 |
|  | 6 Nervousness | B | 125 | -0.15 | 0.0156 | 0.1240 | 79 | -0.02 | 0.7589 | 0.8981 |
|  | 7 Calming difficulty | B | 124 | -0.06 | 0.3107 | 0.5874 | 78 | -0.05 | 0.4846 | 0.7376 |
|  | 8 Emotional numbness | B | 123 | -0.09 | 0.1352 | 0.3901 | 78 | 0.00 | 0.9881 | 1.0000 |
|  | 9 Sense of failure | B | 124 | -0.16 | 0.0107 | 0.1008 | 79 | -0.06 | 0.4560 | 0.7147 |
|  | 10 Self-doubt | B | 125 | -0.12 | 0.0391 | 0.2013 | 78 | 0.02 | 0.8414 | 0.9329 |
|  | 11 Disconnection to others | B | 124 | -0.09 | 0.1282 | 0.3808 | 79 | 0.05 | 0.5339 | 0.7697 |
|  | 12 Social difficulty | B | 125 | 0.02 | 0.7699 | 0.9036 | 78 | 0.18 | 0.0232 | 0.1561 |
| 9 Tendency to cry | 1 Bad dreams | A | 126 | 0.01 | 0.9019 | 0.9616 | 80 | 0.01 | 0.9080 | 0.9652 |
|  | 2 Reliving events in mind | A | 125 | -0.05 | 0.3954 | 0.6635 | 80 | -0.25 | 0.0011 | 0.0247 |
|  | 3 Avoiding thoughts | A | 123 | -0.01 | 0.8316 | 0.9305 | 79 | 0.04 | 0.6031 | 0.8164 |
|  | 4 Avoiding physically | A | 123 | -0.03 | 0.6266 | 0.8319 | 79 | 0.05 | 0.5311 | 0.7684 |
|  | 5 Overly cautious | A | 124 | -0.01 | 0.8404 | 0.9329 | 78 | -0.09 | 0.2481 | 0.5265 |
|  | 6 Nervousness | A | 126 | -0.14 | 0.0226 | 0.1534 | 79 | -0.20 | 0.0108 | 0.1018 |
|  | 7 Calming difficulty | A | 125 | -0.03 | 0.6199 | 0.8276 | 78 | -0.08 | 0.2874 | 0.5622 |
|  | 8 Emotional numbness | A | 124 | -0.03 | 0.6742 | 0.8556 | 78 | 0.02 | 0.8179 | 0.9281 |
|  | 9 Sense of failure | A | 125 | -0.07 | 0.2781 | 0.5534 | 79 | -0.12 | 0.1179 | 0.3671 |
|  | 10 Self-doubt | A | 126 | -0.04 | 0.5563 | 0.7819 | 78 | -0.07 | 0.3713 | 0.6434 |
|  | 11 Disconnection to others | A | 125 | 0.01 | 0.8652 | 0.9433 | 79 | -0.04 | 0.5787 | 0.7985 |
|  | 12 Social difficulty | A | 126 | 0.09 | 0.1537 | 0.4133 | 78 | 0.02 | 0.7495 | 0.8934 |
| 10 Bad mood | 1 Bad dreams | A | 128 | 0.00 | 0.9538 | 0.9857 | 80 | 0.05 | 0.5354 | 0.7703 |
|  | 2 Reliving events in mind | A | 127 | -0.12 | 0.0435 | 0.2148 | 80 | -0.14 | 0.0571 | 0.2470 |
|  | 3 Avoiding thoughts | A | 125 | 0.01 | 0.8718 | 0.9466 | 79 | 0.04 | 0.6070 | 0.8184 |
|  | 4 Avoiding physically | A | 125 | -0.08 | 0.1681 | 0.4348 | 79 | 0.11 | 0.1552 | 0.4149 |
|  | 5 Overly cautious | A | 126 | -0.03 | 0.6307 | 0.8333 | 78 | -0.05 | 0.5459 | 0.7767 |
|  | 6 Nervousness | A | 128 | -0.18 | 0.0020 | 0.0350 | 79 | -0.17 | 0.0225 | 0.1534 |
|  | 7 Calming difficulty | A | 127 | -0.06 | 0.3032 | 0.5804 | 78 | 0.02 | 0.8259 | 0.9291 |
|  | 8 Emotional numbness | A | 126 | -0.05 | 0.4277 | 0.6911 | 78 | 0.02 | 0.7942 | 0.9175 |
|  | 9 Sense of failure | A | 127 | -0.16 | 0.0061 | 0.0707 | 79 | -0.05 | 0.5250 | 0.7644 |
|  | 10 Self-doubt | A | 128 | -0.08 | 0.1694 | 0.4367 | 78 | 0.00 | 0.9738 | 0.9959 |
|  | 11 Disconnection to others | A | 127 | -0.04 | 0.4839 | 0.7375 | 79 | -0.07 | 0.3869 | 0.6557 |
|  | 12 Social difficulty | A | 128 | -0.01 | 0.9216 | 0.9716 | 78 | 0.08 | 0.2998 | 0.5775 |
| 11 Company | 1 Bad dreams | D | 127 | -0.09 | 0.1286 | 0.3812 | 80 | 0.14 | 0.0639 | 0.2647 |
|  | 2 Reliving events in mind | D | 126 | -0.06 | 0.3229 | 0.5994 | 80 | 0.00 | 0.9496 | 0.9833 |
|  | 3 Avoiding thoughts | D | 124 | 0.00 | 0.9480 | 0.9825 | 79 | 0.09 | 0.2394 | 0.5162 |
|  | 4 Avoiding physically | D | 124 | -0.02 | 0.7362 | 0.8887 | 79 | 0.12 | 0.1172 | 0.3662 |
|  | 5 Overly cautious | D | 125 | -0.14 | 0.0194 | 0.1384 | 78 | 0.07 | 0.3790 | 0.6504 |
|  | 6 Nervousness | D | 127 | -0.08 | 0.1612 | 0.4246 | 79 | -0.01 | 0.8580 | 0.9402 |
|  | 7 Calming difficulty | D | 126 | -0.02 | 0.7113 | 0.8782 | 78 | -0.08 | 0.3063 | 0.5827 |
|  | 8 Emotional numbness | D | 125 | -0.06 | 0.3529 | 0.6270 | 78 | 0.03 | 0.7358 | 0.8887 |
|  | 9 Sense of failure | D | 126 | -0.26 | <0.0001 | 0.0020 | 79 | -0.06 | 0.4373 | 0.6985 |
|  | 10 Self-doubt | D | 127 | -0.14 | 0.0198 | 0.1401 | 78 | -0.02 | 0.7529 | 0.8947 |
|  | 11 Disconnection to others | D | 126 | -0.08 | 0.1661 | 0.4307 | 79 | 0.03 | 0.7332 | 0.8878 |
|  | 12 Social difficulty | D | 127 | -0.05 | 0.4069 | 0.6715 | 78 | 0.05 | 0.5279 | 0.7671 |
| 12 Self-determination | 1 Bad dreams | C | 128 | -0.05 | 0.3805 | 0.6515 | 79 | -0.07 | 0.3602 | 0.6333 |
|  | 2 Reliving events in mind | C | 127 | -0.10 | 0.1036 | 0.3445 | 79 | -0.03 | 0.7394 | 0.8895 |
|  | 3 Avoiding thoughts | C | 125 | -0.10 | 0.0999 | 0.3380 | 78 | 0.00 | 1.0000 | 1.0000 |
|  | 4 Avoiding physically | C | 125 | -0.08 | 0.1826 | 0.4503 | 78 | 0.06 | 0.4355 | 0.6975 |
|  | 5 Overly cautious | C | 126 | -0.19 | 0.0015 | 0.0288 | 77 | 0.05 | 0.5184 | 0.7586 |
|  | 6 Nervousness | C | 128 | -0.18 | 0.0020 | 0.0354 | 78 | -0.04 | 0.5682 | 0.7898 |
|  | 7 Calming difficulty | C | 127 | -0.13 | 0.0369 | 0.1945 | 77 | 0.02 | 0.7531 | 0.8947 |
|  | 8 Emotional numbness | C | 126 | -0.12 | 0.0458 | 0.2197 | 77 | 0.07 | 0.3796 | 0.6510 |
|  | 9 Sense of failure | C | 127 | -0.13 | 0.0303 | 0.1771 | 78 | 0.03 | 0.7353 | 0.8887 |
|  | 10 Self-doubt | C | 128 | -0.12 | 0.0407 | 0.2064 | 77 | -0.08 | 0.3275 | 0.6038 |
|  | 11 Disconnection to others | C | 127 | -0.05 | 0.4340 | 0.6972 | 78 | 0.05 | 0.4992 | 0.7458 |
|  | 12 Social difficulty | C | 128 | -0.07 | 0.2174 | 0.4904 | 77 | 0.09 | 0.2701 | 0.5473 |
| 13 Self-perception | 1 Bad dreams | B | 128 | -0.28 | <0.0001 | 0.0008 | 79 | 0.22 | 0.0049 | 0.0607 |
|  | 2 Reliving events in mind | B | 127 | -0.16 | 0.0089 | 0.0892 | 79 | 0.04 | 0.6450 | 0.8394 |
|  | 3 Avoiding thoughts | B | 125 | -0.15 | 0.0148 | 0.1204 | 78 | 0.07 | 0.3740 | 0.6451 |
|  | 4 Avoiding physically | B | 125 | -0.17 | 0.0054 | 0.0647 | 78 | 0.22 | 0.0043 | 0.0557 |
|  | 5 Overly cautious | B | 126 | -0.20 | 0.0008 | 0.0196 | 77 | 0.07 | 0.3854 | 0.6552 |
|  | 6 Nervousness | B | 128 | -0.22 | 0.0002 | 0.0087 | 78 | -0.05 | 0.4915 | 0.7416 |
|  | 7 Calming difficulty | B | 127 | -0.20 | 0.0008 | 0.0203 | 77 | 0.07 | 0.3947 | 0.6628 |
|  | 8 Emotional numbness | B | 126 | -0.22 | 0.0002 | 0.0086 | 77 | 0.11 | 0.1729 | 0.4400 |
|  | 9 Sense of failure | B | 127 | -0.25 | <0.0001 | 0.0026 | 78 | 0.01 | 0.8599 | 0.9402 |
|  | 10 Self-doubt | B | 128 | -0.26 | <0.0001 | 0.0019 | 77 | 0.16 | 0.0351 | 0.1885 |
|  | 11 Disconnection to others | B | 127 | -0.11 | 0.0582 | 0.2499 | 78 | 0.03 | 0.6884 | 0.8642 |
|  | 12 Social difficulty | B | 128 | -0.07 | 0.2385 | 0.5154 | 77 | 0.09 | 0.2532 | 0.5324 |
| 14 Learning attitude | 1 Bad dreams | C | 127 | 0.08 | 0.1943 | 0.4646 | 80 | 0.06 | 0.4671 | 0.7242 |
|  | 2 Reliving events in mind | C | 126 | 0.05 | 0.4032 | 0.6689 | 80 | -0.01 | 0.9430 | 0.9799 |
|  | 3 Avoiding thoughts | C | 124 | 0.07 | 0.2579 | 0.5361 | 79 | 0.08 | 0.3259 | 0.6025 |
|  | 4 Avoiding physically | C | 124 | 0.10 | 0.0923 | 0.3244 | 79 | -0.05 | 0.5513 | 0.7796 |
|  | 5 Overly cautious | C | 125 | 0.08 | 0.1772 | 0.4434 | 78 | 0.02 | 0.7793 | 0.9097 |
|  | 6 Nervousness | C | 127 | -0.04 | 0.5561 | 0.7819 | 79 | -0.15 | 0.0508 | 0.2326 |
|  | 7 Calming difficulty | C | 126 | 0.05 | 0.3685 | 0.6400 | 78 | -0.02 | 0.7688 | 0.9034 |
|  | 8 Emotional numbness | C | 125 | -0.05 | 0.4293 | 0.6920 | 78 | 0.01 | 0.8858 | 0.9533 |
|  | 9 Sense of failure | C | 126 | 0.12 | 0.0400 | 0.2042 | 79 | -0.11 | 0.1347 | 0.3900 |
|  | 10 Self-doubt | C | 127 | -0.07 | 0.2776 | 0.5528 | 78 | -0.10 | 0.2062 | 0.4804 |
|  | 11 Disconnection to others | C | 126 | 0.13 | 0.0307 | 0.1773 | 79 | -0.18 | 0.0170 | 0.1302 |
|  | 12 Social difficulty | C | 127 | 0.08 | 0.1820 | 0.4495 | 78 | -0.07 | 0.3618 | 0.6348 |
| 15 Sleep quality | 1 Bad dreams | A | 127 | 0.04 | 0.5297 | 0.7684 | 80 | 0.03 | 0.6675 | 0.8526 |
|  | 2 Reliving events in mind | A | 126 | 0.10 | 0.0912 | 0.3230 | 80 | -0.05 | 0.5083 | 0.7505 |
|  | 3 Avoiding thoughts | A | 124 | 0.04 | 0.5455 | 0.7767 | 79 | -0.02 | 0.8039 | 0.9211 |
|  | 4 Avoiding physically | A | 124 | -0.03 | 0.6718 | 0.8546 | 79 | -0.07 | 0.3899 | 0.6589 |
|  | 5 Overly cautious | A | 125 | 0.02 | 0.7913 | 0.9160 | 78 | 0.00 | 0.9614 | 0.9896 |
|  | 6 Nervousness | A | 127 | -0.05 | 0.4055 | 0.6705 | 79 | -0.26 | 0.0006 | 0.0155 |
|  | 7 Calming difficulty | A | 126 | 0.00 | 1.0000 | 1.0000 | 78 | -0.18 | 0.0233 | 0.1566 |
|  | 8 Emotional numbness | A | 125 | 0.00 | 0.9941 | 1.0000 | 78 | -0.02 | 0.8117 | 0.9250 |
|  | 9 Sense of failure | A | 126 | -0.03 | 0.6351 | 0.8355 | 79 | -0.20 | 0.0084 | 0.0863 |
|  | 10 Self-doubt | A | 127 | -0.05 | 0.4208 | 0.6851 | 78 | -0.01 | 0.9083 | 0.9652 |
|  | 11 Disconnection to others | A | 126 | 0.01 | 0.8828 | 0.9519 | 79 | -0.17 | 0.0238 | 0.1583 |
|  | 12 Social difficulty | A | 127 | -0.01 | 0.9140 | 0.9671 | 78 | -0.01 | 0.8959 | 0.9594 |
| 16 Tiredness level | 1 Bad dreams | A | 126 | -0.02 | 0.7636 | 0.9000 | 79 | 0.07 | 0.3642 | 0.6374 |
|  | 2 Reliving events in mind | A | 125 | -0.04 | 0.5137 | 0.7552 | 79 | -0.08 | 0.3058 | 0.5826 |
|  | 3 Avoiding thoughts | A | 123 | 0.07 | 0.2840 | 0.5586 | 78 | 0.02 | 0.7624 | 0.8998 |
|  | 4 Avoiding physically | A | 123 | 0.01 | 0.9047 | 0.9637 | 78 | 0.01 | 0.8673 | 0.9446 |
|  | 5 Overly cautious | A | 124 | -0.08 | 0.1956 | 0.4653 | 77 | -0.02 | 0.8006 | 0.9192 |
|  | 6 Nervousness | A | 126 | -0.06 | 0.3199 | 0.5959 | 78 | -0.11 | 0.1696 | 0.4367 |
|  | 7 Calming difficulty | A | 125 | -0.03 | 0.6299 | 0.8331 | 77 | -0.07 | 0.3408 | 0.6161 |
|  | 8 Emotional numbness | A | 124 | -0.01 | 0.8967 | 0.9594 | 77 | 0.01 | 0.8601 | 0.9402 |
|  | 9 Sense of failure | A | 125 | -0.04 | 0.4988 | 0.7455 | 78 | -0.08 | 0.2892 | 0.5637 |
|  | 10 Self-doubt | A | 126 | -0.13 | 0.0336 | 0.1848 | 77 | -0.06 | 0.4749 | 0.7311 |
|  | 11 Disconnection to others | A | 125 | -0.07 | 0.2420 | 0.5195 | 78 | 0.00 | 0.9692 | 0.9929 |
|  | 12 Social difficulty | A | 126 | -0.06 | 0.2895 | 0.5641 | 77 | 0.14 | 0.0778 | 0.2955 |
| 17 Eating attitude | 1 Bad dreams | A | 126 | -0.06 | 0.3150 | 0.5914 | 79 | 0.12 | 0.1233 | 0.3736 |
|  | 2 Reliving events in mind | A | 125 | -0.10 | 0.1009 | 0.3402 | 79 | -0.05 | 0.5517 | 0.7797 |
|  | 3 Avoiding thoughts | A | 123 | -0.08 | 0.1844 | 0.4535 | 78 | 0.12 | 0.1176 | 0.3670 |
|  | 4 Avoiding physically | A | 123 | -0.06 | 0.3402 | 0.6157 | 78 | 0.17 | 0.0236 | 0.1580 |
|  | 5 Overly cautious | A | 124 | -0.05 | 0.3931 | 0.6611 | 77 | 0.06 | 0.4116 | 0.6759 |
|  | 6 Nervousness | A | 126 | -0.21 | 0.0005 | 0.0151 | 78 | 0.04 | 0.6312 | 0.8336 |
|  | 7 Calming difficulty | A | 125 | -0.12 | 0.0569 | 0.2465 | 77 | 0.06 | 0.4059 | 0.6708 |
|  | 8 Emotional numbness | A | 124 | -0.11 | 0.0708 | 0.2802 | 77 | 0.04 | 0.5708 | 0.7921 |
|  | 9 Sense of failure | A | 125 | -0.20 | 0.0012 | 0.0254 | 78 | -0.06 | 0.4185 | 0.6831 |
|  | 10 Self-doubt | A | 126 | -0.10 | 0.0902 | 0.3212 | 77 | 0.03 | 0.6640 | 0.8517 |
|  | 11 Disconnection to others | A | 125 | -0.10 | 0.0899 | 0.3206 | 78 | 0.03 | 0.6823 | 0.8604 |
|  | 12 Social difficulty | A | 126 | -0.13 | 0.0263 | 0.1643 | 77 | 0.23 | 0.0034 | 0.0478 |
| 18 Pain thoughts | 1 Bad dreams | A | 123 | -0.27 | <0.0001 | 0.0012 | 79 | -0.15 | 0.0447 | 0.2171 |
|  | 2 Reliving events in mind | A | 122 | -0.25 | 0.0001 | 0.0037 | 79 | -0.09 | 0.2303 | 0.5059 |
|  | 3 Avoiding thoughts | A | 120 | -0.23 | 0.0002 | 0.0072 | 78 | -0.18 | 0.0191 | 0.1375 |
|  | 4 Avoiding physically | A | 120 | -0.17 | 0.0062 | 0.0718 | 78 | 0.08 | 0.3308 | 0.6076 |
|  | 5 Overly cautious | A | 121 | -0.25 | 0.0001 | 0.0037 | 77 | -0.10 | 0.2064 | 0.4804 |
|  | 6 Nervousness | A | 123 | -0.23 | 0.0001 | 0.0063 | 78 | -0.02 | 0.7480 | 0.8934 |
|  | 7 Calming difficulty | A | 122 | -0.14 | 0.0228 | 0.1546 | 77 | 0.04 | 0.6177 | 0.8257 |
|  | 8 Emotional numbness | A | 121 | -0.13 | 0.0342 | 0.1864 | 77 | -0.02 | 0.7500 | 0.8934 |
|  | 9 Sense of failure | A | 122 | -0.15 | 0.0145 | 0.1188 | 78 | -0.02 | 0.8037 | 0.9211 |
|  | 10 Self-doubt | A | 123 | -0.16 | 0.0075 | 0.0805 | 78 | -0.13 | 0.0986 | 0.3357 |
|  | 11 Disconnection to others | A | 122 | -0.11 | 0.0711 | 0.2807 | 78 | 0.04 | 0.5783 | 0.7981 |
|  | 12 Social difficulty | A | 123 | -0.16 | 0.0071 | 0.0778 | 77 | -0.03 | 0.6605 | 0.8487 |
| 19 Loneliness feeling | 1 Bad dreams | D | 126 | -0.08 | 0.1852 | 0.4535 | 77 | 0.08 | 0.3155 | 0.5918 |
|  | 2 Reliving events in mind | D | 125 | -0.06 | 0.2864 | 0.5611 | 77 | -0.11 | 0.1446 | 0.4025 |
|  | 3 Avoiding thoughts | D | 123 | -0.06 | 0.3150 | 0.5914 | 76 | 0.06 | 0.4357 | 0.6975 |
|  | 4 Avoiding physically | D | 123 | -0.01 | 0.9071 | 0.9652 | 76 | 0.00 | 1.0000 | 1.0000 |
|  | 5 Overly cautious | D | 124 | -0.06 | 0.3521 | 0.6265 | 75 | -0.12 | 0.1163 | 0.3656 |
|  | 6 Nervousness | D | 126 | -0.11 | 0.0588 | 0.2517 | 76 | -0.10 | 0.2172 | 0.4904 |
|  | 7 Calming difficulty | D | 125 | -0.04 | 0.5464 | 0.7767 | 75 | -0.03 | 0.7406 | 0.8899 |
|  | 8 Emotional numbness | D | 124 | -0.10 | 0.0951 | 0.3292 | 75 | -0.04 | 0.6024 | 0.8162 |
|  | 9 Sense of failure | D | 126 | -0.11 | 0.0581 | 0.2499 | 76 | -0.14 | 0.0828 | 0.3044 |
|  | 10 Self-doubt | D | 126 | -0.13 | 0.0327 | 0.1822 | 75 | -0.09 | 0.2770 | 0.5526 |
|  | 11 Disconnection to others | D | 125 | -0.07 | 0.2549 | 0.5324 | 76 | -0.08 | 0.3294 | 0.6066 |
|  | 12 Social difficulty | D | 126 | -0.07 | 0.2150 | 0.4876 | 75 | 0.05 | 0.5112 | 0.7532 |
| 20 School fun | 1 Bad dreams | C | 127 | -0.07 | 0.2571 | 0.5348 | 73 | 0.09 | 0.2510 | 0.5301 |
|  | 2 Reliving events in mind | C | 126 | -0.09 | 0.1160 | 0.3656 | 73 | -0.15 | 0.0605 | 0.2555 |
|  | 3 Avoiding thoughts | C | 124 | -0.10 | 0.1153 | 0.3644 | 72 | -0.03 | 0.7453 | 0.8919 |
|  | 4 Avoiding physically | C | 124 | -0.13 | 0.0312 | 0.1782 | 72 | 0.12 | 0.1206 | 0.3707 |
|  | 5 Overly cautious | C | 125 | -0.24 | 0.0001 | 0.0050 | 71 | -0.12 | 0.1411 | 0.3971 |
|  | 6 Nervousness | C | 127 | -0.33 | <0.0001 | <0.0001 | 72 | -0.07 | 0.3968 | 0.6650 |
|  | 7 Calming difficulty | C | 126 | -0.16 | 0.0065 | 0.0732 | 71 | -0.04 | 0.6377 | 0.8355 |
|  | 8 Emotional numbness | C | 125 | -0.22 | 0.0003 | 0.0110 | 71 | 0.07 | 0.4041 | 0.6694 |
|  | 9 Sense of failure | C | 126 | -0.26 | <0.0001 | 0.0019 | 79 | N/A | N/A | N/A |
|  | 10 Self-doubt | C | 127 | -0.22 | 0.0003 | 0.0106 | 79 | N/A | N/A | N/A |
|  | 11 Disconnection to others | C | 126 | -0.17 | 0.0037 | 0.0503 | 72 | 0.00 | 0.9546 | 0.9861 |
|  | 12 Social difficulty | C | 127 | -0.16 | 0.0090 | 0.0896 | 71 | -0.01 | 0.8831 | 0.9520 |
| 21 Friends | 1 Bad dreams | D | 126 | 0.00 | 0.9572 | 0.9877 | 78 | 0.09 | 0.2301 | 0.5056 |
|  | 2 Reliving events in mind | D | 125 | -0.09 | 0.1409 | 0.3969 | 78 | -0.17 | 0.0265 | 0.1643 |
|  | 3 Avoiding thoughts | D | 123 | 0.01 | 0.8373 | 0.9327 | 77 | 0.13 | 0.1001 | 0.3384 |
|  | 4 Avoiding physically | D | 123 | 0.01 | 0.8463 | 0.9343 | 77 | 0.09 | 0.2320 | 0.5082 |
|  | 5 Overly cautious | D | 124 | -0.07 | 0.2248 | 0.4988 | 76 | -0.10 | 0.2051 | 0.4789 |
|  | 6 Nervousness | D | 126 | -0.11 | 0.0728 | 0.2853 | 77 | -0.15 | 0.0599 | 0.2549 |
|  | 7 Calming difficulty | D | 125 | -0.06 | 0.3016 | 0.5794 | 76 | -0.11 | 0.1749 | 0.4408 |
|  | 8 Emotional numbness | D | 124 | -0.09 | 0.1470 | 0.4047 | 76 | -0.02 | 0.7893 | 0.9157 |
|  | 9 Sense of failure | D | 125 | -0.10 | 0.0864 | 0.3122 | 77 | -0.07 | 0.3467 | 0.6210 |
|  | 10 Self-doubt | D | 126 | -0.07 | 0.2331 | 0.5093 | 76 | -0.10 | 0.1897 | 0.4591 |
|  | 11 Disconnection to others | D | 125 | -0.02 | 0.7627 | 0.8998 | 77 | -0.02 | 0.8008 | 0.9192 |
|  | 12 Social difficulty | D | 126 | -0.02 | 0.7349 | 0.8887 | 76 | 0.06 | 0.4113 | 0.6758 |
| 22 Dealing with school tasks | 1 Bad dreams | C | 127 | -0.04 | 0.5386 | 0.7728 | 73 | 0.16 | 0.0425 | 0.2122 |
|  | 2 Reliving events in mind | C | 126 | -0.03 | 0.6088 | 0.8195 | 73 | 0.11 | 0.1869 | 0.4553 |
|  | 3 Avoiding thoughts | C | 124 | 0.08 | 0.1906 | 0.4598 | 72 | 0.17 | 0.0394 | 0.2025 |
|  | 4 Avoiding physically | C | 124 | 0.03 | 0.5671 | 0.7896 | 72 | 0.33 | <0.0001 | 0.0028 |
|  | 5 Overly cautious | C | 125 | -0.01 | 0.9310 | 0.9748 | 71 | -0.07 | 0.3711 | 0.6432 |
|  | 6 Nervousness | C | 127 | -0.06 | 0.3391 | 0.6148 | 72 | -0.04 | 0.5853 | 0.8034 |
|  | 7 Calming difficulty | C | 126 | 0.01 | 0.9117 | 0.9667 | 71 | 0.12 | 0.1274 | 0.3800 |
|  | 8 Emotional numbness | C | 125 | 0.02 | 0.7070 | 0.8765 | 71 | 0.12 | 0.1295 | 0.3822 |
|  | 9 Sense of failure | C | 126 | -0.04 | 0.4781 | 0.7340 | 72 | 0.11 | 0.1624 | 0.4258 |
|  | 10 Self-doubt | C | 127 | -0.09 | 0.1289 | 0.3816 | 71 | 0.05 | 0.5012 | 0.7458 |
|  | 11 Disconnection to others | C | 126 | -0.01 | 0.9323 | 0.9748 | 72 | 0.03 | 0.7505 | 0.8934 |
|  | 12 Social difficulty | C | 127 | 0.02 | 0.6803 | 0.8596 | 71 | 0.05 | 0.5013 | 0.7458 |
| 23 Self-comparison to others | 1 Bad dreams | C | 127 | -0.03 | 0.6166 | 0.8256 | 79 | 0.00 | 0.9574 | 0.9877 |
|  | 2 Reliving events in mind | C | 126 | -0.09 | 0.1159 | 0.3656 | 79 | -0.09 | 0.2379 | 0.5148 |
|  | 3 Avoiding thoughts | C | 124 | 0.02 | 0.7889 | 0.9157 | 78 | -0.02 | 0.7902 | 0.9160 |
|  | 4 Avoiding physically | C | 124 | 0.00 | 0.9716 | 0.9945 | 78 | -0.04 | 0.5607 | 0.7858 |
|  | 5 Overly cautious | C | 125 | -0.06 | 0.3219 | 0.5983 | 77 | -0.09 | 0.2601 | 0.5368 |
|  | 6 Nervousness | C | 127 | -0.10 | 0.0986 | 0.3357 | 78 | -0.13 | 0.1023 | 0.3421 |
|  | 7 Calming difficulty | C | 126 | -0.03 | 0.6066 | 0.8181 | 77 | -0.08 | 0.2859 | 0.5611 |
|  | 8 Emotional numbness | C | 125 | -0.03 | 0.6282 | 0.8325 | 77 | -0.09 | 0.2287 | 0.5036 |
|  | 9 Sense of failure | C | 126 | -0.10 | 0.1080 | 0.3521 | 78 | -0.10 | 0.1871 | 0.4553 |
|  | 10 Self-doubt | C | 127 | -0.08 | 0.2071 | 0.4808 | 77 | -0.14 | 0.0805 | 0.2987 |
|  | 11 Disconnection to others | C | 126 | -0.02 | 0.6945 | 0.8682 | 78 | -0.07 | 0.3788 | 0.6503 |
|  | 12 Social difficulty | C | 127 | 0.05 | 0.4476 | 0.7064 | 77 | 0.01 | 0.8987 | 0.9603 |
| 24 Love-awareness | 1 Bad dreams | B | 127 | -0.18 | 0.0023 | 0.0377 | 80 | 0.14 | 0.0580 | 0.2499 |
|  | 2 Reliving events in mind | B | 126 | -0.10 | 0.1099 | 0.3549 | 80 | -0.01 | 0.9235 | 0.9720 |
|  | 3 Avoiding thoughts | B | 124 | -0.11 | 0.0688 | 0.2752 | 79 | 0.04 | 0.5665 | 0.7893 |
|  | 4 Avoiding physically | B | 124 | 0.01 | 0.8796 | 0.9497 | 79 | 0.07 | 0.3469 | 0.6210 |
|  | 5 Overly cautious | B | 125 | -0.06 | 0.3627 | 0.6355 | 78 | -0.06 | 0.4207 | 0.6851 |
|  | 6 Nervousness | B | 127 | -0.19 | 0.0015 | 0.0288 | 79 | -0.03 | 0.7169 | 0.8811 |
|  | 7 Calming difficulty | B | 126 | -0.10 | 0.0919 | 0.3238 | 78 | 0.05 | 0.4817 | 0.7363 |
|  | 8 Emotional numbness | B | 125 | -0.15 | 0.0106 | 0.1006 | 78 | 0.04 | 0.6177 | 0.8257 |
|  | 9 Sense of failure | B | 126 | -0.18 | 0.0036 | 0.0494 | 79 | -0.03 | 0.6772 | 0.8579 |
|  | 10 Self-doubt | B | 127 | -0.18 | 0.0026 | 0.0410 | 78 | -0.01 | 0.8995 | 0.9604 |
|  | 11 Disconnection to others | B | 126 | -0.09 | 0.1165 | 0.3656 | 79 | 0.04 | 0.5953 | 0.8098 |
|  | 12 Social difficulty | B | 127 | -0.06 | 0.3132 | 0.5903 | 78 | 0.02 | 0.8171 | 0.9281 |
| 25 Peer arguing | 1 Bad dreams | D | 127 | -0.11 | 0.0581 | 0.2499 | 80 | 0.04 | 0.6370 | 0.8355 |
|  | 2 Reliving events in mind | D | 126 | -0.12 | 0.0379 | 0.1972 | 80 | -0.08 | 0.2732 | 0.5496 |
|  | 3 Avoiding thoughts | D | 124 | -0.05 | 0.3656 | 0.6381 | 79 | 0.12 | 0.1113 | 0.3573 |
|  | 4 Avoiding physically | D | 124 | -0.06 | 0.3545 | 0.6286 | 79 | 0.03 | 0.7172 | 0.8812 |
|  | 5 Overly cautious | D | 125 | -0.14 | 0.0186 | 0.1363 | 78 | -0.02 | 0.8332 | 0.9313 |
|  | 6 Nervousness | D | 127 | -0.17 | 0.0056 | 0.0671 | 79 | -0.13 | 0.0976 | 0.3338 |
|  | 7 Calming difficulty | D | 126 | -0.07 | 0.2758 | 0.5511 | 78 | 0.05 | 0.5306 | 0.7684 |
|  | 8 Emotional numbness | D | 125 | -0.20 | 0.0012 | 0.0250 | 78 | 0.01 | 0.8899 | 0.9556 |
|  | 9 Sense of failure | D | 126 | -0.22 | 0.0002 | 0.0077 | 79 | 0.09 | 0.2600 | 0.5368 |
|  | 10 Self-doubt | D | 127 | -0.18 | 0.0033 | 0.0471 | 78 | 0.01 | 0.9453 | 0.9813 |
|  | 11 Disconnection to others | D | 126 | -0.17 | 0.0039 | 0.0520 | 79 | 0.11 | 0.1448 | 0.4029 |
|  | 12 Social difficulty | D | 127 | -0.16 | 0.0079 | 0.0830 | 78 | 0.23 | 0.0024 | 0.0388 |
| 26 Napping/dozing | 1 Bad dreams | A | 128 | -0.06 | 0.3063 | 0.5827 | 79 | 0.01 | 0.8573 | 0.9402 |
|  | 2 Reliving events in mind | A | 127 | -0.04 | 0.5005 | 0.7458 | 79 | -0.11 | 0.1647 | 0.4293 |
|  | 3 Avoiding thoughts | A | 125 | -0.09 | 0.1351 | 0.3900 | 78 | 0.04 | 0.5930 | 0.8079 |
|  | 4 Avoiding physically | A | 125 | 0.04 | 0.5440 | 0.7766 | 78 | 0.07 | 0.3618 | 0.6348 |
|  | 5 Overly cautious | A | 126 | -0.02 | 0.7645 | 0.9009 | 77 | 0.00 | 0.9885 | 1.0000 |
|  | 6 Nervousness | A | 128 | -0.08 | 0.1623 | 0.4258 | 78 | -0.04 | 0.5849 | 0.8033 |
|  | 7 Calming difficulty | A | 127 | 0.00 | 0.9381 | 0.9762 | 77 | 0.00 | 0.9538 | 0.9857 |
|  | 8 Emotional numbness | A | 126 | -0.02 | 0.7283 | 0.8850 | 77 | -0.06 | 0.4076 | 0.6718 |
|  | 9 Sense of failure | A | 127 | -0.11 | 0.0750 | 0.2900 | 78 | -0.14 | 0.0702 | 0.2789 |
|  | 10 Self-doubt | A | 128 | -0.06 | 0.3061 | 0.5827 | 77 | -0.14 | 0.0758 | 0.2916 |
|  | 11 Disconnection to others | A | 127 | -0.02 | 0.7008 | 0.8728 | 78 | -0.17 | 0.0264 | 0.1643 |
|  | 12 Social difficulty | A | 128 | 0.02 | 0.7144 | 0.8798 | 77 | 0.03 | 0.6668 | 0.8526 |
| 27 Eating problems | 1 Bad dreams | A | 124 | 0.05 | 0.4108 | 0.6750 | 80 | 0.00 | 0.9792 | 0.9995 |
|  | 2 Reliving events in mind | A | 124 | 0.07 | 0.2600 | 0.5368 | 80 | -0.05 | 0.4813 | 0.7363 |
|  | 3 Avoiding thoughts | A | 122 | 0.10 | 0.1079 | 0.3521 | 79 | 0.10 | 0.1736 | 0.4408 |
|  | 4 Avoiding physically | A | 122 | 0.16 | 0.0096 | 0.0937 | 79 | 0.06 | 0.4688 | 0.7250 |
|  | 5 Overly cautious | A | 123 | 0.10 | 0.1037 | 0.3446 | 78 | -0.10 | 0.2107 | 0.4851 |
|  | 6 Nervousness | A | 124 | 0.05 | 0.3670 | 0.6392 | 79 | -0.13 | 0.0820 | 0.3025 |
|  | 7 Calming difficulty | A | 123 | 0.14 | 0.0243 | 0.1602 | 78 | -0.01 | 0.8838 | 0.9524 |
|  | 8 Emotional numbness | A | 122 | 0.14 | 0.0255 | 0.1630 | 78 | -0.01 | 0.8578 | 0.9402 |
|  | 9 Sense of failure | A | 123 | -0.01 | 0.8750 | 0.9478 | 79 | 0.02 | 0.7582 | 0.8974 |
|  | 10 Self-doubt | A | 124 | -0.04 | 0.4636 | 0.7211 | 78 | -0.03 | 0.6557 | 0.8447 |
|  | 11 Disconnection to others | A | 123 | 0.02 | 0.7190 | 0.8824 | 79 | 0.07 | 0.3855 | 0.6552 |
|  | 12 Social difficulty | A | 124 | 0.19 | 0.0015 | 0.0296 | 78 | 0.04 | 0.6104 | 0.8209 |
| 28 Memorization | 1 Bad dreams | C | 128 | -0.16 | 0.0063 | 0.0722 | 80 | 0.11 | 0.1396 | 0.3957 |
|  | 2 Reliving events in mind | C | 127 | -0.07 | 0.2298 | 0.5056 | 80 | -0.09 | 0.2639 | 0.5427 |
|  | 3 Avoiding thoughts | C | 125 | -0.09 | 0.1403 | 0.3963 | 79 | 0.08 | 0.3030 | 0.5804 |
|  | 4 Avoiding physically | C | 125 | -0.07 | 0.2550 | 0.5324 | 79 | 0.10 | 0.2077 | 0.4812 |
|  | 5 Overly cautious | C | 126 | -0.12 | 0.0434 | 0.2146 | 78 | 0.02 | 0.8070 | 0.9224 |
|  | 6 Nervousness | C | 128 | -0.17 | 0.0049 | 0.0607 | 79 | 0.00 | 0.9895 | 1.0000 |
|  | 7 Calming difficulty | C | 127 | -0.09 | 0.1434 | 0.3998 | 78 | 0.18 | 0.0174 | 0.1309 |
|  | 8 Emotional numbness | C | 126 | -0.05 | 0.3939 | 0.6621 | 78 | 0.07 | 0.3965 | 0.6647 |
|  | 9 Sense of failure | C | 127 | -0.15 | 0.0115 | 0.1046 | 79 | 0.04 | 0.6187 | 0.8266 |
|  | 10 Self-doubt | C | 128 | -0.18 | 0.0033 | 0.0473 | 78 | -0.06 | 0.4356 | 0.6975 |
|  | 11 Disconnection to others | C | 127 | -0.15 | 0.0124 | 0.1087 | 79 | -0.05 | 0.4769 | 0.7329 |
|  | 12 Social difficulty | C | 128 | -0.13 | 0.0272 | 0.1669 | 78 | 0.06 | 0.4480 | 0.7068 |

*B-H* – Benjamini-Hochberg correction for multiple comparisons, *p*-values < 0.05 are indicated in red, N – number of participants, Tau – Tau correlation coefficient
