# Supplementary material for: Demographic predictors of trauma and depression in war-affected children from Poland and Ukraine: Implications for prevention of mental health problems
Source: Prev Med Rep. 2026 Feb 20;63:103422. doi: 10.1016/j.pmedr.2026.103422 (PMC12945576; doi:10.1016/j.pmedr.2026.103422)
Supplement: Supplementary file 4 — Supplementary material 4 [file mmc4.docx]

**CDI (panels A-D) and ITQ-CA correlations in country return dependent groups**

**CDI2 – group B**

In the group of children for whom the home country return was not applicable (Polish citizens or Ukrainian-born in Poland), we observed 33 significant, negative, very weak or weak correlations in CDI2–group A between data obtained from CDI2 and ITQ-CA questionnaires (Figure S4). Most of the correlations were between CDI2-17 “Eating attitude”, CDI2-18 “Pain thoughts” and CDI2-5 “Bad mood” with 8, 7 and 5 ITQ-CA categories, respectively. After the Benjamini-Hochberg correction, 13 significant correlations remained (Table S4). The correlations were between CDI2-10 “Bad mood” with ITQ-CA “Nervousness”, “Sense of failure”, Self-doubt”, CDI2-17 “Eating attitude” with ITQ-CA “Nervousness” and “Sense of failure”, CDI2-18 “Pain thoughts” with ITQ-CA “Bad dreams”, Reliving thoughts in mind”, “Avoiding thoughts”, “Avoiding physically”, “Overlay cautions” and “Nervousness”, CDI2-26 “Napping/dozing” and CDI2-27 “Eating problems” both with ITQ-CA “Sense of failure” (Table S4). Among the Ukrainian children who did not return to their homes, we observed 24 significant, positive, and moderate to strong correlations between CDI2 and ITQ-C (Figure S4). Most of them were between CDI2-10 “Bad mood”, CDI2-17 “Eating attitude”, and CDI2-26 “Napping/dozing” with 6, 6, and 4 ITQ-CA categories. After Benjamini-Hochberg correction, three correlations remain significant between CDI2-17 “Eating attitude” with ITQ-CA “Overly cautious” and CDI2-26 “Napping/dozing” with ITQ-CA “Reliving events in mind” and “Avoiding physically” (Table S4). Among the Ukrainian children who returned to their homes, we observed 16 significant correlations. Ten correlations were positive and six negative (all very weak or weak) (Figure S4). Most positive correlations were in the ITQ-CA category “Social difficulty” and negative with “Nervousness”. After correction for multiple comparisons, the negative correlation between CDI2-15 “Sleep quality” with ITQ-CA “Nervousness” and CDI2-27 “Eating problems” with ITQ-CA “Social difficulty” remained significant (Table S4).

**CDI2 – group B**

In the CDI2 – group B among the children for whom the home country return was not applicable, we observed 38 significant, negative, very weak or weak in strength correlations between data obtained from CDI2 and ITQ-CA questionnaires (Figure S4). Most of the correlations were between CDI2-13 “Self-perception”, CDI2-2 “Hopeful”, CDI2-24 “Love-awareness”, and CDI2-7 “Blame” with 11, 8, 7, and 6 ITQ-CA categories, respectively. After the Benjamini-Hochberg correction, 15 correlations remained significant (Table S4). The correlations were between CDI2-2 “Hopeful” with ITQ-CA “Nervousness”, “Calming difficulty”, “Sense of failure”, Self-doubt”, CDI2-7 “Blame” with ITQ-CA “Nervousness”, CDI2-13 “Self-perception” with ITQ-CA “Bad dreams”, “Avoiding physically”, “Nervousness”, “Calming difficulty”, “Emotional numbness”, “Sense of failure”, and Self-doubt”, CDI2-24 “Love-awareness” with “Bad dreams”, “Avoiding thoughts”, and “Sense of failure” (Table S4). Among the Ukrainian children who did not return to their homes, we observed 18 significant, positive, and moderate to strong correlations between CDI2 and ITQ-CA (Figure S4). Most of them were between CDI2-6 “Self-acceptance” with ITQ-CA 8 categories, and CDI2-2 “Hopeful” and CDI-24 “Love-awareness” with ITQ-CA 4 categories. After Benjamini-Hochberg correction, we observed six significant, positive and moderate to strong correlations between CDI2 and ITQ-C (Table S4) between CDI2-6 “Self-acceptance” with ITQ-CA “Calming difficulty”, CDI2-17 “Emotional numbness” and “Social difficulty”, and CDI2-24 “Love awareness” with ITQ-CA “Reliving events in mind”, “Avoiding physically”, and “Overly cautions” (Table S4). We observed eight significant correlations among the Ukrainian children who returned to their homes. Four correlations were positive and four negative (all very weak or weak) (Figure S4). After correction for multiple comparisons, the positive correlation between CDI2-8 “Suicide” and ITQ-CA “Social difficulty” remained significant (Table S4).

**CDI2 – group C**

In the CDI2 – group C among the children for whom the home country return was not applicable, we observed 42 significant, negative, very weak or weak in strength correlations between data obtained from CDI2 and ITQ-CA questionnaires (Figure S4). Most of the correlations were between CDI2-20 “School fun”, CDI2-12 “Self-determination”, CDI2-28 “Memorization”, and CDI2-4 “Memorization” with 11, 8, 8, and 7 ITQ-CA categories, respectively. After the Benjamini-Hochberg correction, 16 correlations remained significant (Table S4). The correlations were between CDI2-3 “Self-confidence” with ITQ-CA “Nervousness”, “Sense of failure” and “Self-doubt”, CDI2-4 “Enjoyment” with ITQ-CA “Nervousness”, “Sense of failure” and “Social difficulty”, CDI-12 “Self-determination” and CDI2-20 “School fun” with ITQ-CA “Nervousness”, “Calming difficulty”, “Sense of failure” and “Self-doubt”, CDI2-28 “Memorization” with ITQ-CA “Sense of failure” and “Self-doubt” (Table S4). Among the Ukrainian children who did not return to their homes, we observed 25 significant, positive, and moderate to strong correlations between CDI2 and ITQ-CA (Figure S4). Most of them were between CDI2-3 “Self-comparison” with ITQ-CA 6 categories, CDI2-3 “Self-confidence” with ITQ-CA 5 categories, and CDI2-12 “Self-determination, CDI2-22 “Dealing with school tasks” and CDI2-28 “Memorization”, all with ITQ-CA 4 categories. After Benjamini-Hochberg correction, we observed two significant correlations between CDI2 and ITQ-CA (Table S4). The correlations were between CDI2-12 “Self-determination” with ITQ-CA “Nervousness”, and CDI2-23 “Self-comparison to others” with “Calming difficulty” (Table S4). Among the Ukrainian children returning home, we observed 11 significant correlations (very weak to moderate). One correlation was negative (between CDI2-20 “School fun” with ITQ-CA “Overlay cautions”. Ten remaining correlations were positive (Figure S4). After correction for multiple comparisons, a significant correlation remained between CDI2-22 “Dealing with school tasks” with ITQ-CA “Avoiding physically” and CDI-28 “Memorization” with ITQ-CA “Calming difficulty” (Table S4).

**CDI2 – group D**

Among the children in CDI2–group D for whom the home country return was not applicable, 28 significant, negative, very weak or weak in strength correlations between data obtained from CDI2 and ITQ-CA questionnaires (Figure S4). Most correlations were between CDI2-5 “Importance for family” and all 12 ITQ-CA categories. The remain correlations were between CDI2-11 “Company” and ITQ-CA “Avoiding thoughts”, “Sense of failure” and “Self-doubt”, CDI2-19 “Loneliness feeling” and CDI2-21 “Friends”, and CDI2-25 “Peer arguing” with ITQ-CA “Nervousness”, “Emotional numbness” (for CDI2-21 and CDI2-25) or “Calming difficulty” (for CDI2-21), “Sense of failure” and “Self-doubt”. After the Benjamini-Hochberg correction, 15 correlations remained significant (Table S4). In eight correlations, CDI2-5 “Importance for family” remains significant. In other correlations, the most abundant was the ITQ-CA category “Sense of failure” correlated with CDI2-11 “Company”, CDI2-19 “Loneliness feeling”, CDI2-21 “Friends”, and CDI2-25 “Peer arguing”. The CDI2-19 was also correlated with ITQ-CA “Nervousness”, and CDI2-25 with both ITQ-CA “Emotional numbness” and “Self-doubt” (Table S4). Among the Ukrainian children who did not return to their homes, we observed 21 significant, positive, and moderate to strong correlations between CDI2 and ITQ-CA (Figure S4). Most of them were between CDI2-25 “Peer arguing” with 6 ITQ-CA categories, CDI2-5 “Importance for family”, CDI2-11 “Company”, CDI2-21 “Friends” all with 4 ITQ-CA categories, and CDI2-19 “Loneliness feeling” with 3 ITQ-CA categories. After Benjamini-Hochberg correction, we observed seven significant correlations between CDI2 and ITQ-CA (Table S4). Correlations that remained significant were between CDI2-5 “Importance for family” with ITQ-CA “Emotional numbness” and “Social difficulty”, CDI2-11 with ITQ-CA “Bad dreams”, “Avoiding thoughts”, and “Overly cautious”, and CDI2-25 with ITQ-CA “Avoiding physically”, and “Overly cautious” (Table S4). We observed six significant correlations among the Ukrainian children who returned to their homes (very weak to weak). One correlation was positive (between CDI2-11 “Company” with ITQ-CA “Avoiding thoughts”). The remaining correlations were negative, between CDI2-21 “Friends” and CDI2-25 “Peer arguing” with ITQ-CA “Relieving events in mind”, and “Nervousness”, and additionally CDI2-25 with ITQ-CA “Social difficulty” (Figure S4). After correction for multiple comparisons, none of the correlations remained significant (Table S4).


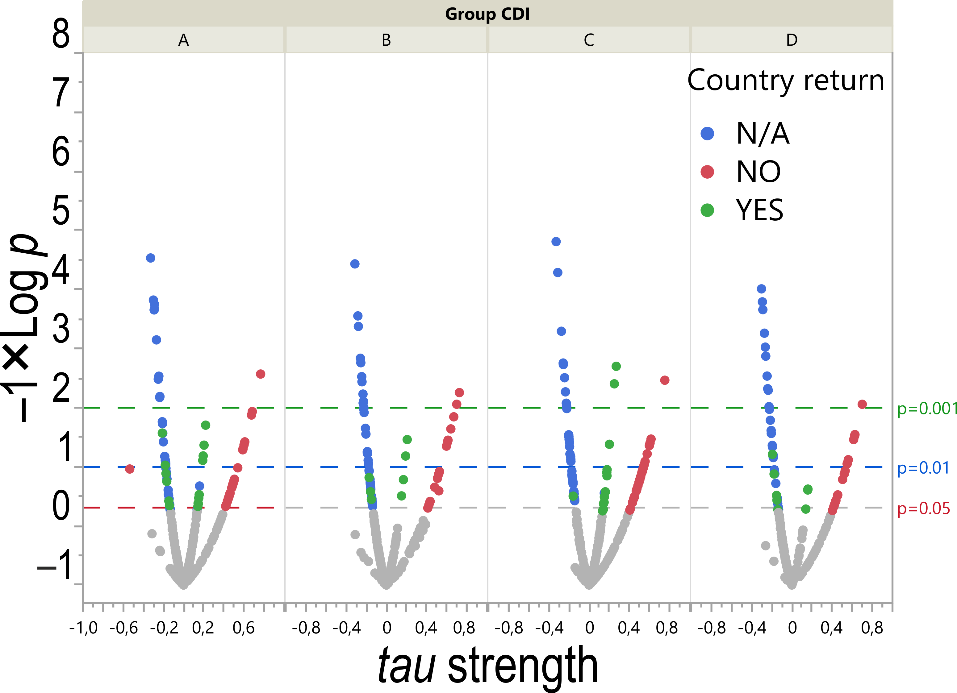


**Figure S4.** Correlations between single questions of CDI-2 (Children's Depression Inventory 2) and ITQ-CA (International Trauma Questionnaire – Child and Adolescent Version) in the country of origin return dependent groups of children and adolescents from Poland and Ukraine. Volcano plot of significant correlations between single questions of CDI and ITQ-CA in the CDI A, B, C, and D groups. The strength of *tau* correlation coefficient (x-axis) and the significance as the unadjusted *p*-values (shown as −1×log *p*; y-axis). The dashed horizontal lines represent the *p* = 0.05 (red), *p* = 0.01 (blue), and *p* = 0.001 (green). Significant correlations are shown as colored spots, and non-significant correlations are grey. March 2024-March 2025, Poland and Ukraine.

**Table S4.** Children's Depression Inventory 2 (CDI-2) (panels A-D) and International Trauma Questionnaire – Child and Adolescent Version (ITQ-CA) correlations in country return dependent groups of children and adolescents from Poland and Ukraine, March 2024-March 2025, Poland and Ukraine.

| **CDI2** | **ITQ-CA** | **Group CDI** | **N** | ***Tau*** | ***p*-value** | ***p*-value ^BH^** | **N** | ***Tau*** | ***p*-value** | ***p*-value ^BH^** | **N** | ***Tau*** | ***p*-value** | ***p*-value ^BH^** |
| --- | --- | --- | --- | --- | --- | --- | --- | --- | --- | --- | --- | --- | --- | --- |
|  |  | **Country of origin return - Yes** | | | | | **Country of origin return - NO** | | | | **Country of origin return – N/A** | | | |
| 1 Sadness | 1 Bad dreams | A | 92 | 0.05 | 0.4684 | 0.7249 | 13 | N/A | N/A | N/A | 97 | 0.10 | 0.1494 | 0.4072 |
|  | 2 Reliving events in mind | A | 92 | -0.09 | 0.2040 | 0.4781 | 13 | N/A | N/A | N/A | 96 | 0.01 | 0.8579 | 0.9402 |
|  | 3 Avoiding thoughts | A | 91 | 0.20 | 0.0043 | 0.0561 | 13 | N/A | N/A | N/A | 95 | 0.11 | 0.1235 | 0.3736 |
|  | 4 Avoiding physically | A | 91 | 0.09 | 0.2103 | 0.4849 | 13 | N/A | N/A | N/A | 94 | 0.04 | 0.5482 | 0.7772 |
|  | 5 Overly cautious | A | 90 | -0.05 | 0.4969 | 0.7437 | 13 | N/A | N/A | N/A | 95 | 0.09 | 0.1873 | 0.4556 |
|  | 6 Nervousness | A | 91 | 0.00 | 0.9640 | 0.9911 | 13 | N/A | N/A | N/A | 97 | 0.09 | 0.1863 | 0.4546 |
|  | 7 Calming difficulty | A | 90 | 0.14 | 0.0468 | 0.2227 | 13 | N/A | N/A | N/A | 96 | 0.13 | 0.0626 | 0.2606 |
|  | 8 Emotional numbness | A | 91 | 0.06 | 0.3926 | 0.6605 | 13 | N/A | N/A | N/A | 95 | 0.13 | 0.0531 | 0.2374 |
|  | 9 Sense of failure | A | 90 | 0.19 | 0.0078 | 0.0821 | 13 | N/A | N/A | N/A | 97 | 0.02 | 0.8243 | 0.9290 |
|  | 10 Self-doubt | A | 91 | 0.11 | 0.1183 | 0.3679 | 13 | N/A | N/A | N/A | 96 | -0.01 | 0.9404 | 0.9778 |
|  | 11 Disconnection to others | A | 91 | 0.03 | 0.6870 | 0.8631 | 13 | N/A | N/A | N/A | 96 | 0.07 | 0.3069 | 0.5830 |
|  | 12 Social difficulty | A | 90 | 0.15 | 0.0425 | 0.2122 | 13 | N/A | N/A | N/A | 97 | 0.16 | 0.0212 | 0.1471 |
| 2 Hopeful | 1 Bad dreams | B | 94 | 0.02 | 0.7237 | 0.8834 | 13 | 0.41 | 0.0498 | 0.2293 | 101 | -0.16 | 0.0162 | 0.1274 |
|  | 2 Reliving events in mind | B | 94 | -0.11 | 0.1307 | 0.3834 | 13 | -0.09 | 0.6743 | 0.8556 | 100 | -0.15 | 0.0308 | 0.1773 |
|  | 3 Avoiding thoughts | B | 93 | 0.02 | 0.7836 | 0.9118 | 13 | 0.37 | 0.0813 | 0.3009 | 98 | -0.15 | 0.0242 | 0.1594 |
|  | 4 Avoiding physically | B | 93 | 0.19 | 0.0066 | 0.0737 | 13 | 0.25 | 0.2422 | 0.5195 | 98 | -0.13 | 0.0627 | 0.2609 |
|  | 5 Overly cautious | B | 92 | -0.15 | 0.0356 | 0.1905 | 13 | 0.42 | 0.0440 | 0.2156 | 99 | -0.15 | 0.0314 | 0.1782 |
|  | 6 Nervousness | B | 93 | -0.10 | 0.1373 | 0.3925 | 13 | 0.34 | 0.1070 | 0.3506 | 101 | -0.25 | 0.0002 | 0.0072 |
|  | 7 Calming difficulty | B | 92 | 0.10 | 0.1702 | 0.4367 | 13 | 0.60 | 0.0045 | 0.0570 | 100 | -0.20 | 0.0028 | 0.0428 |
|  | 8 Emotional numbness | B | 92 | 0.03 | 0.6955 | 0.8688 | 13 | 0.48 | 0.0221 | 0.1519 | 99 | -0.11 | 0.1222 | 0.3724 |
|  | 9 Sense of failure | B | 92 | 0.00 | 0.9463 | 0.9819 | 13 | -0.04 | 0.8350 | 0.9318 | 101 | -0.28 | <0.0001 | 0.0023 |
|  | 10 Self-doubt | B | 93 | -0.01 | 0.8789 | 0.9495 | 13 | 0.23 | 0.2819 | 0.5582 | 100 | -0.23 | 0.0008 | 0.0196 |
|  | 11 Disconnection to others | B | 93 | -0.05 | 0.4549 | 0.7138 | 13 | 0.07 | 0.7390 | 0.8895 | 100 | -0.12 | 0.0795 | 0.2983 |
|  | 12 Social difficulty | B | 92 | 0.02 | 0.8047 | 0.9214 | 13 | 0.23 | 0.2760 | 0.5511 | 101 | -0.06 | 0.4074 | 0.6718 |
| 3 Self-confidence | 1 Bad dreams | C | 94 | 0.08 | 0.2643 | 0.5431 | 13 | 0.53 | 0.0121 | 0.1073 | 101 | 0.02 | 0.7730 | 0.9056 |
|  | 2 Reliving events in mind | C | 94 | -0.05 | 0.5000 | 0.7458 | 13 | 0.14 | 0.5016 | 0.7459 | 100 | -0.05 | 0.4434 | 0.7031 |
|  | 3 Avoiding thoughts | C | 93 | 0.14 | 0.0421 | 0.2112 | 13 | 0.47 | 0.0258 | 0.1638 | 98 | -0.09 | 0.1676 | 0.4340 |
|  | 4 Avoiding physically | C | 93 | 0.10 | 0.1465 | 0.4044 | 13 | 0.16 | 0.4545 | 0.7135 | 98 | -0.03 | 0.6687 | 0.8530 |
|  | 5 Overly cautious | C | 92 | -0.09 | 0.2132 | 0.4868 | 13 | 0.51 | 0.0147 | 0.1196 | 99 | -0.06 | 0.3586 | 0.6326 |
|  | 6 Nervousness | C | 93 | -0.03 | 0.6444 | 0.8393 | 13 | 0.32 | 0.1221 | 0.3723 | 101 | -0.20 | 0.0031 | 0.0452 |
|  | 7 Calming difficulty | C | 92 | 0.12 | 0.0808 | 0.2993 | 13 | 0.55 | 0.0094 | 0.0923 | 100 | -0.09 | 0.1991 | 0.4705 |
|  | 8 Emotional numbness | C | 92 | 0.03 | 0.6561 | 0.8449 | 13 | 0.38 | 0.0700 | 0.2785 | 99 | -0.05 | 0.4886 | 0.7396 |
|  | 9 Sense of failure | C | 92 | 0.10 | 0.1487 | 0.4061 | 13 | 0.20 | 0.3511 | 0.6253 | 101 | -0.22 | 0.0010 | 0.0221 |
|  | 10 Self-doubt | C | 93 | 0.00 | 0.9913 | 1.0000 | 13 | 0.45 | 0.0322 | 0.1813 | 100 | -0.20 | 0.0031 | 0.0451 |
|  | 11 Disconnection to others | C | 93 | 0.01 | 0.8594 | 0.9402 | 13 | 0.30 | 0.1554 | 0.4152 | 100 | -0.09 | 0.1902 | 0.4598 |
|  | 12 Social difficulty | C | 92 | 0.12 | 0.0981 | 0.3347 | 13 | 0.38 | 0.0700 | 0.2785 | 101 | -0.06 | 0.3734 | 0.6451 |
| 4 Enjoyment | 1 Bad dreams | C | 94 | 0.13 | 0.0556 | 0.2436 | 13 | N/A | N/A | N/A | 100 | -0.09 | 0.1619 | 0.4254 |
|  | 2 Reliving events in mind | C | 94 | -0.04 | 0.5718 | 0.7928 | 13 | N/A | N/A | N/A | 99 | 0.04 | 0.5851 | 0.8034 |
|  | 3 Avoiding thoughts | C | 93 | 0.18 | 0.0125 | 0.1090 | 13 | N/A | N/A | N/A | 97 | 0.02 | 0.8178 | 0.9281 |
|  | 4 Avoiding physically | C | 93 | 0.20 | 0.0042 | 0.0548 | 13 | N/A | N/A | N/A | 97 | 0.10 | 0.1420 | 0.3984 |
|  | 5 Overly cautious | C | 92 | -0.03 | 0.6595 | 0.8480 | 13 | N/A | N/A | N/A | 98 | -0.02 | 0.7497 | 0.8934 |
|  | 6 Nervousness | C | 93 | -0.03 | 0.6544 | 0.8440 | 13 | N/A | N/A | N/A | 100 | -0.27 | 0.0001 | 0.0034 |
|  | 7 Calming difficulty | C | 92 | 0.08 | 0.2738 | 0.5496 | 13 | N/A | N/A | N/A | 99 | -0.19 | 0.0046 | 0.0579 |
|  | 8 Emotional numbness | C | 92 | -0.03 | 0.7073 | 0.8765 | 13 | N/A | N/A | N/A | 98 | -0.17 | 0.0142 | 0.1170 |
|  | 9 Sense of failure | C | 92 | 0.06 | 0.4092 | 0.6738 | 13 | N/A | N/A | N/A | 100 | -0.23 | 0.0008 | 0.0204 |
|  | 10 Self-doubt | C | 93 | 0.11 | 0.1085 | 0.3530 | 13 | N/A | N/A | N/A | 99 | -0.18 | 0.0081 | 0.0842 |
|  | 11 Disconnection to others | C | 93 | 0.09 | 0.2142 | 0.4875 | 13 | N/A | N/A | N/A | 99 | -0.16 | 0.0168 | 0.1294 |
|  | 12 Social difficulty | C | 92 | 0.12 | 0.0829 | 0.3044 | 13 | N/A | N/A | N/A | 100 | -0.22 | 0.0009 | 0.0215 |
| 5 Importance for family | 1 Bad dreams | D | 93 | 0.04 | 0.6132 | 0.8237 | 13 | 0.40 | 0.0545 | 0.2401 | 101 | -0.26 | 0.0001 | 0.0050 |
|  | 2 Reliving events in mind | D | 93 | -0.11 | 0.1349 | 0.3900 | 13 | 0.17 | 0.4205 | 0.6851 | 100 | -0.25 | 0.0003 | 0.0102 |
|  | 3 Avoiding thoughts | D | 92 | -0.05 | 0.5034 | 0.7466 | 13 | 0.38 | 0.0734 | 0.2872 | 98 | -0.14 | 0.0480 | 0.2250 |
|  | 4 Avoiding physically | D | 92 | 0.08 | 0.2595 | 0.5368 | 13 | 0.40 | 0.0547 | 0.2406 | 98 | -0.24 | 0.0005 | 0.0142 |
|  | 5 Overly cautious | D | 91 | -0.10 | 0.1771 | 0.4434 | 13 | 0.24 | 0.2456 | 0.5243 | 99 | -0.17 | 0.0131 | 0.1111 |
|  | 6 Nervousness | D | 92 | -0.07 | 0.3306 | 0.6074 | 13 | 0.42 | 0.0470 | 0.2227 | 101 | -0.18 | 0.0065 | 0.0732 |
|  | 7 Calming difficulty | D | 91 | 0.02 | 0.7991 | 0.9192 | 13 | 0.56 | 0.0076 | 0.0810 | 100 | -0.16 | 0.0195 | 0.1387 |
|  | 8 Emotional numbness | D | 91 | -0.10 | 0.1713 | 0.4379 | 13 | 0.63 | 0.0028 | 0.0428 | 99 | -0.30 | <0.0001 | 0.0014 |
|  | 9 Sense of failure | D | 91 | -0.01 | 0.8638 | 0.9421 | 13 | 0.24 | 0.2539 | 0.5324 | 101 | -0.27 | 0.0001 | 0.0036 |
|  | 10 Self-doubt | D | 92 | 0.03 | 0.7134 | 0.8796 | 13 | 0.39 | 0.0667 | 0.2719 | 100 | -0.29 | <0.0001 | 0.0019 |
|  | 11 Disconnection to others | D | 92 | 0.11 | 0.1177 | 0.3671 | 13 | 0.23 | 0.2735 | 0.5496 | 100 | -0.26 | 0.0001 | 0.0062 |
|  | 12 Social difficulty | D | 91 | 0.11 | 0.1322 | 0.3857 | 13 | 0.63 | 0.0028 | 0.0428 | 101 | -0.23 | 0.0005 | 0.0144 |
| 6 Self-acceptance | 1 Bad dreams | B | 94 | 0.06 | 0.4286 | 0.6917 | 13 | 0.52 | 0.0132 | 0.1111 | 100 | -0.10 | 0.1305 | 0.3834 |
|  | 2 Reliving events in mind | B | 94 | -0.08 | 0.2477 | 0.5264 | 13 | 0.00 | 1.0000 | 1.0000 | 99 | -0.08 | 0.2548 | 0.5324 |
|  | 3 Avoiding thoughts | B | 93 | 0.04 | 0.6078 | 0.8192 | 13 | 0.43 | 0.0385 | 0.1992 | 97 | -0.08 | 0.2495 | 0.5280 |
|  | 4 Avoiding physically | B | 93 | 0.07 | 0.3312 | 0.6078 | 13 | 0.25 | 0.2422 | 0.5195 | 97 | -0.14 | 0.0420 | 0.2110 |
|  | 5 Overly cautious | B | 92 | -0.07 | 0.3353 | 0.6116 | 13 | 0.42 | 0.0440 | 0.2156 | 98 | -0.05 | 0.4270 | 0.6909 |
|  | 6 Nervousness | B | 93 | -0.17 | 0.0152 | 0.1220 | 13 | 0.51 | 0.0156 | 0.1241 | 100 | -0.17 | 0.0116 | 0.1052 |
|  | 7 Calming difficulty | B | 92 | -0.16 | 0.0264 | 0.1643 | 13 | 0.73 | 0.0006 | 0.0152 | 99 | -0.10 | 0.1440 | 0.4012 |
|  | 8 Emotional numbness | B | 92 | 0.00 | 0.9913 | 1.0000 | 13 | 0.64 | 0.0023 | 0.0377 | 98 | -0.13 | 0.0671 | 0.2721 |
|  | 9 Sense of failure | B | 92 | -0.08 | 0.2583 | 0.5367 | 13 | 0.24 | 0.2520 | 0.5314 | 100 | -0.18 | 0.0078 | 0.0821 |
|  | 10 Self-doubt | B | 93 | 0.02 | 0.8281 | 0.9297 | 13 | 0.53 | 0.0120 | 0.1071 | 99 | -0.09 | 0.1688 | 0.4361 |
|  | 11 Disconnection to others | B | 93 | -0.01 | 0.8923 | 0.9570 | 13 | 0.33 | 0.1200 | 0.3698 | 99 | -0.10 | 0.1274 | 0.3800 |
|  | 12 Social difficulty | B | 92 | 0.05 | 0.4983 | 0.7449 | 13 | 0.64 | 0.0023 | 0.0377 | 100 | -0.06 | 0.3620 | 0.6348 |
| 7 Blame | 1 Bad dreams | B | 94 | 0.09 | 0.2239 | 0.4974 | 12 | -0.02 | 0.9249 | 0.9720 | 100 | -0.14 | 0.0351 | 0.1886 |
|  | 2 Reliving events in mind | B | 94 | -0.02 | 0.7703 | 0.9039 | 12 | -0.02 | 0.9256 | 0.9720 | 99 | -0.14 | 0.0392 | 0.2016 |
|  | 3 Avoiding thoughts | B | 93 | -0.02 | 0.7233 | 0.8834 | 12 | 0.06 | 0.7727 | 0.9054 | 97 | -0.07 | 0.2931 | 0.5685 |
|  | 4 Avoiding physically | B | 93 | 0.10 | 0.1604 | 0.4238 | 12 | 0.07 | 0.7571 | 0.8969 | 97 | -0.12 | 0.0802 | 0.2987 |
|  | 5 Overly cautious | B | 92 | 0.06 | 0.3763 | 0.6477 | 12 | 0.23 | 0.2946 | 0.5703 | 98 | -0.11 | 0.0996 | 0.3377 |
|  | 6 Nervousness | B | 93 | -0.08 | 0.2346 | 0.5117 | 12 | 0.08 | 0.7142 | 0.8798 | 100 | -0.22 | 0.0010 | 0.0228 |
|  | 7 Calming difficulty | B | 92 | -0.05 | 0.5196 | 0.7599 | 12 | 0.06 | 0.7815 | 0.9110 | 99 | -0.11 | 0.1113 | 0.3573 |
|  | 8 Emotional numbness | B | 92 | -0.01 | 0.8458 | 0.9343 | 12 | 0.02 | 0.9225 | 0.9718 | 98 | -0.07 | 0.2752 | 0.5508 |
|  | 9 Sense of failure | B | 92 | -0.09 | 0.2231 | 0.4966 | 12 | 0.15 | 0.5003 | 0.7458 | 100 | -0.18 | 0.0089 | 0.0892 |
|  | 10 Self-doubt | B | 93 | -0.01 | 0.9200 | 0.9709 | 12 | 0.07 | 0.7378 | 0.8895 | 99 | -0.15 | 0.0232 | 0.1563 |
|  | 11 Disconnection to others | B | 93 | -0.10 | 0.1392 | 0.3957 | 12 | -0.02 | 0.9216 | 0.9716 | 99 | -0.14 | 0.0335 | 0.1848 |
|  | 12 Social difficulty | B | 92 | -0.07 | 0.3166 | 0.5923 | 12 | 0.02 | 0.9225 | 0.9718 | 100 | -0.12 | 0.0883 | 0.3164 |
| 8 Suicide | 1 Bad dreams | B | 94 | 0.11 | 0.1096 | 0.3547 | 11 | 0.36 | 0.1194 | 0.3686 | 100 | -0.05 | 0.4314 | 0.6944 |
|  | 2 Reliving events in mind | B | 94 | -0.12 | 0.0856 | 0.3114 | 11 | -0.25 | 0.2813 | 0.5578 | 99 | -0.04 | 0.5622 | 0.7861 |
|  | 3 Avoiding thoughts | B | 93 | 0.09 | 0.1995 | 0.4708 | 11 | 0.33 | 0.1560 | 0.4164 | 97 | -0.06 | 0.4140 | 0.6781 |
|  | 4 Avoiding physically | B | 93 | 0.07 | 0.3455 | 0.6205 | 11 | 0.12 | 0.6138 | 0.8239 | 97 | -0.08 | 0.2552 | 0.5324 |
|  | 5 Overly cautious | B | 92 | -0.11 | 0.1299 | 0.3830 | 11 | 0.52 | 0.0255 | 0.1630 | 98 | -0.01 | 0.8746 | 0.9478 |
|  | 6 Nervousness | B | 93 | -0.09 | 0.1883 | 0.4572 | 11 | 0.29 | 0.2182 | 0.4909 | 100 | -0.10 | 0.1487 | 0.4061 |
|  | 7 Calming difficulty | B | 92 | -0.02 | 0.7341 | 0.8884 | 11 | 0.39 | 0.0942 | 0.3269 | 99 | -0.07 | 0.2819 | 0.5582 |
|  | 8 Emotional numbness | B | 92 | 0.06 | 0.3820 | 0.6530 | 11 | 0.15 | 0.5180 | 0.7583 | 98 | -0.14 | 0.0464 | 0.2210 |
|  | 9 Sense of failure | B | 92 | -0.03 | 0.7076 | 0.8767 | 11 | -0.22 | 0.3443 | 0.6190 | 100 | -0.16 | 0.0216 | 0.1493 |
|  | 10 Self-doubt | B | 93 | 0.05 | 0.5021 | 0.7460 | 11 | 0.08 | 0.7170 | 0.8811 | 99 | -0.19 | 0.0060 | 0.0703 |
|  | 11 Disconnection to others | B | 93 | 0.00 | 1.0000 | 1.0000 | 11 | -0.11 | 0.6278 | 0.8325 | 99 | 0.01 | 0.8872 | 0.9537 |
|  | 12 Social difficulty | B | 92 | 0.21 | 0.0035 | 0.0481 | 11 | 0.15 | 0.5180 | 0.7583 | 100 | 0.01 | 0.8464 | 0.9343 |
| 9 Tendency to cry | 1 Bad dreams | A | 94 | 0.09 | 0.1942 | 0.4646 | 13 | 0.19 | 0.3780 | 0.6493 | 99 | -0.06 | 0.3510 | 0.6253 |
|  | 2 Reliving events in mind | A | 94 | -0.18 | 0.0094 | 0.0923 | 13 | -0.04 | 0.8420 | 0.9329 | 98 | -0.08 | 0.2401 | 0.5170 |
|  | 3 Avoiding thoughts | A | 93 | 0.08 | 0.2555 | 0.5326 | 13 | 0.22 | 0.3014 | 0.5794 | 96 | -0.09 | 0.1809 | 0.4483 |
|  | 4 Avoiding physically | A | 93 | 0.02 | 0.7769 | 0.9079 | 13 | 0.35 | 0.0961 | 0.3316 | 96 | -0.05 | 0.4761 | 0.7324 |
|  | 5 Overly cautious | A | 92 | -0.06 | 0.3792 | 0.6504 | 13 | 0.42 | 0.0443 | 0.2160 | 97 | -0.05 | 0.4963 | 0.7437 |
|  | 6 Nervousness | A | 93 | -0.17 | 0.0176 | 0.1319 | 13 | 0.28 | 0.1810 | 0.4483 | 99 | -0.19 | 0.0066 | 0.0741 |
|  | 7 Calming difficulty | A | 92 | -0.01 | 0.9191 | 0.9705 | 13 | 0.49 | 0.0208 | 0.1455 | 98 | -0.13 | 0.0639 | 0.2647 |
|  | 8 Emotional numbness | A | 92 | 0.02 | 0.7677 | 0.9031 | 13 | 0.28 | 0.1791 | 0.4465 | 97 | -0.03 | 0.6855 | 0.8623 |
|  | 9 Sense of failure | A | 92 | -0.01 | 0.8600 | 0.9402 | 13 | 0.19 | 0.3739 | 0.6451 | 99 | -0.17 | 0.0138 | 0.1147 |
|  | 10 Self-doubt | A | 93 | 0.04 | 0.5689 | 0.7903 | 13 | 0.10 | 0.6500 | 0.8413 | 98 | -0.15 | 0.0304 | 0.1771 |
|  | 11 Disconnection to others | A | 93 | -0.01 | 0.9241 | 0.9720 | 13 | 0.18 | 0.3993 | 0.6665 | 98 | 0.00 | 0.9500 | 0.9833 |
|  | 12 Social difficulty | A | 92 | 0.07 | 0.3565 | 0.6307 | 13 | 0.28 | 0.1791 | 0.4465 | 99 | 0.05 | 0.5072 | 0.7497 |
| 10 Bad mood | 1 Bad dreams | A | 94 | 0.12 | 0.0974 | 0.3336 | 13 | 0.45 | 0.0317 | 0.1794 | 101 | -0.08 | 0.2348 | 0.5118 |
|  | 2 Reliving events in mind | A | 94 | -0.09 | 0.2075 | 0.4812 | 13 | 0.29 | 0.1620 | 0.4254 | 100 | -0.18 | 0.0082 | 0.0848 |
|  | 3 Avoiding thoughts | A | 93 | 0.09 | 0.2179 | 0.4907 | 13 | 0.46 | 0.0296 | 0.1754 | 98 | -0.07 | 0.2739 | 0.5496 |
|  | 4 Avoiding physically | A | 93 | 0.10 | 0.1524 | 0.4114 | 13 | 0.22 | 0.2845 | 0.5587 | 98 | -0.10 | 0.1370 | 0.3923 |
|  | 5 Overly cautious | A | 92 | 0.01 | 0.9107 | 0.9661 | 13 | 0.50 | 0.0173 | 0.1306 | 99 | -0.08 | 0.2350 | 0.5118 |
|  | 6 Nervousness | A | 93 | -0.17 | 0.0130 | 0.1107 | 13 | 0.25 | 0.2407 | 0.5177 | 101 | -0.21 | 0.0019 | 0.0331 |
|  | 7 Calming difficulty | A | 92 | 0.07 | 0.3133 | 0.5903 | 13 | 0.46 | 0.0280 | 0.1698 | 100 | -0.15 | 0.0308 | 0.1773 |
|  | 8 Emotional numbness | A | 92 | 0.04 | 0.5834 | 0.8021 | 13 | 0.59 | 0.0050 | 0.0609 | 99 | -0.13 | 0.0621 | 0.2592 |
|  | 9 Sense of failure | A | 92 | 0.06 | 0.3654 | 0.6381 | 13 | 0.09 | 0.6648 | 0.8526 | 101 | -0.29 | <0.0001 | 0.0019 |
|  | 10 Self-doubt | A | 93 | 0.10 | 0.1546 | 0.4146 | 13 | 0.42 | 0.0466 | 0.2219 | 100 | -0.29 | <0.0001 | 0.0020 |
|  | 11 Disconnection to others | A | 93 | 0.02 | 0.7988 | 0.9192 | 13 | 0.17 | 0.4056 | 0.6705 | 100 | -0.09 | 0.1927 | 0.4624 |
|  | 12 Social difficulty | A | 92 | 0.13 | 0.0574 | 0.2483 | 13 | 0.11 | 0.5865 | 0.8044 | 101 | -0.03 | 0.6153 | 0.8243 |
| 11 Company | 1 Bad dreams | D | 94 | 0.08 | 0.2372 | 0.5141 | 13 | 0.70 | 0.0009 | 0.0207 | 100 | -0.08 | 0.2163 | 0.4892 |
|  | 2 Reliving events in mind | D | 94 | 0.03 | 0.7090 | 0.8769 | 13 | 0.24 | 0.2465 | 0.5250 | 99 | -0.06 | 0.3415 | 0.6166 |
|  | 3 Avoiding thoughts | D | 93 | 0.16 | 0.0247 | 0.1602 | 13 | 0.70 | 0.0009 | 0.0207 | 97 | -0.14 | 0.0419 | 0.2107 |
|  | 4 Avoiding physically | D | 93 | 0.07 | 0.3144 | 0.5907 | 13 | 0.44 | 0.0372 | 0.1950 | 97 | 0.00 | 0.9770 | 0.9985 |
|  | 5 Overly cautious | D | 92 | -0.09 | 0.2215 | 0.4949 | 13 | 0.62 | 0.0030 | 0.0440 | 98 | -0.04 | 0.5776 | 0.7976 |
|  | 6 Nervousness | D | 93 | -0.01 | 0.8715 | 0.9465 | 13 | 0.23 | 0.2665 | 0.5466 | 100 | -0.08 | 0.2474 | 0.5260 |
|  | 7 Calming difficulty | D | 92 | 0.01 | 0.8758 | 0.9480 | 13 | 0.36 | 0.0848 | 0.3089 | 99 | -0.09 | 0.1886 | 0.4575 |
|  | 8 Emotional numbness | D | 92 | 0.03 | 0.6224 | 0.8291 | 13 | 0.27 | 0.1956 | 0.4653 | 98 | -0.04 | 0.5117 | 0.7538 |
|  | 9 Sense of failure | D | 92 | -0.01 | 0.8981 | 0.9599 | 13 | -0.26 | 0.2160 | 0.4890 | 100 | -0.29 | <0.0001 | 0.0020 |
|  | 10 Self-doubt | D | 93 | 0.01 | 0.8378 | 0.9329 | 13 | 0.00 | 1.0000 | 1.0000 | 99 | -0.14 | 0.0340 | 0.1860 |
|  | 11 Disconnection to others | D | 93 | 0.04 | 0.5620 | 0.7861 | 13 | -0.18 | 0.3964 | 0.6647 | 99 | -0.03 | 0.7057 | 0.8759 |
|  | 12 Social difficulty | D | 92 | 0.03 | 0.6950 | 0.8686 | 13 | 0.00 | 1.0000 | 1.0000 | 100 | -0.02 | 0.8164 | 0.9276 |
| 12 Self-determination | 1 Bad dreams | C | 93 | 0.00 | 0.9597 | 0.9888 | 13 | 0.23 | 0.2752 | 0.5508 | 101 | -0.12 | 0.0775 | 0.2952 |
|  | 2 Reliving events in mind | C | 93 | 0.01 | 0.8805 | 0.9501 | 13 | 0.25 | 0.2258 | 0.4997 | 100 | -0.15 | 0.0288 | 0.1726 |
|  | 3 Avoiding thoughts | C | 92 | -0.01 | 0.9432 | 0.9800 | 13 | 0.07 | 0.7535 | 0.8947 | 98 | -0.17 | 0.0151 | 0.1219 |
|  | 4 Avoiding physically | C | 92 | 0.09 | 0.1918 | 0.4615 | 13 | 0.02 | 0.9105 | 0.9661 | 98 | -0.11 | 0.1008 | 0.3400 |
|  | 5 Overly cautious | C | 91 | 0.04 | 0.5495 | 0.7783 | 13 | 0.00 | 1.0000 | 1.0000 | 99 | -0.15 | 0.0259 | 0.1643 |
|  | 6 Nervousness | C | 92 | -0.10 | 0.1702 | 0.4367 | 13 | 0.75 | 0.0003 | 0.0110 | 101 | -0.22 | 0.0010 | 0.0233 |
|  | 7 Calming difficulty | C | 91 | 0.03 | 0.7050 | 0.8754 | 13 | 0.41 | 0.0510 | 0.2326 | 100 | -0.20 | 0.0034 | 0.0480 |
|  | 8 Emotional numbness | C | 91 | 0.06 | 0.3718 | 0.6438 | 13 | 0.53 | 0.0120 | 0.1069 | 99 | -0.16 | 0.0194 | 0.1384 |
|  | 9 Sense of failure | C | 91 | 0.10 | 0.1589 | 0.4217 | 13 | 0.50 | 0.0163 | 0.1275 | 101 | -0.27 | 0.0001 | 0.0033 |
|  | 10 Self-doubt | C | 92 | 0.00 | 1.0000 | 1.0000 | 13 | 0.24 | 0.2507 | 0.5296 | 100 | -0.20 | 0.0029 | 0.0430 |
|  | 11 Disconnection to others | C | 92 | 0.11 | 0.1255 | 0.3772 | 13 | 0.61 | 0.0040 | 0.0527 | 100 | -0.12 | 0.0774 | 0.2952 |
|  | 12 Social difficulty | C | 91 | 0.12 | 0.1056 | 0.3487 | 13 | 0.13 | 0.5300 | 0.7684 | 101 | -0.12 | 0.0788 | 0.2970 |
| 13 Self-perception | 1 Bad dreams | B | 93 | 0.10 | 0.1449 | 0.4031 | 13 | -0.02 | 0.9280 | 0.9738 | 101 | -0.23 | 0.0006 | 0.0158 |
|  | 2 Reliving events in mind | B | 93 | -0.02 | 0.7998 | 0.9192 | 13 | 0.27 | 0.1980 | 0.4692 | 100 | -0.16 | 0.0182 | 0.1346 |
|  | 3 Avoiding thoughts | B | 92 | 0.09 | 0.1898 | 0.4591 | 13 | 0.12 | 0.5673 | 0.7897 | 98 | -0.19 | 0.0058 | 0.0687 |
|  | 4 Avoiding physically | B | 92 | 0.17 | 0.0165 | 0.1281 | 13 | 0.43 | 0.0407 | 0.2064 | 98 | -0.22 | 0.0010 | 0.0233 |
|  | 5 Overly cautious | B | 91 | -0.04 | 0.5801 | 0.7994 | 13 | 0.31 | 0.1378 | 0.3936 | 99 | -0.14 | 0.0406 | 0.2064 |
|  | 6 Nervousness | B | 92 | -0.07 | 0.3359 | 0.6122 | 13 | 0.07 | 0.7244 | 0.8839 | 101 | -0.24 | 0.0003 | 0.0102 |
|  | 7 Calming difficulty | B | 91 | 0.04 | 0.6041 | 0.8171 | 13 | 0.07 | 0.7222 | 0.8832 | 100 | -0.21 | 0.0022 | 0.0377 |
|  | 8 Emotional numbness | B | 91 | 0.02 | 0.8321 | 0.9307 | 13 | 0.28 | 0.1819 | 0.4495 | 99 | -0.22 | 0.0012 | 0.0254 |
|  | 9 Sense of failure | B | 91 | 0.02 | 0.8317 | 0.9305 | 13 | 0.23 | 0.2741 | 0.5497 | 101 | -0.31 | <0.0001 | 0.0011 |
|  | 10 Self-doubt | B | 92 | 0.09 | 0.1845 | 0.4535 | 13 | 0.18 | 0.4025 | 0.6689 | 100 | -0.24 | 0.0004 | 0.0115 |
|  | 11 Disconnection to others | B | 92 | 0.05 | 0.4844 | 0.7376 | 13 | 0.16 | 0.4368 | 0.6984 | 100 | -0.17 | 0.0115 | 0.1047 |
|  | 12 Social difficulty | B | 91 | 0.08 | 0.2827 | 0.5585 | 13 | 0.28 | 0.1819 | 0.4495 | 101 | -0.10 | 0.1395 | 0.3957 |
| 14 Learning attitude | 1 Bad dreams | C | 93 | 0.18 | 0.0112 | 0.1039 | 13 | -0.14 | 0.5078 | 0.7502 | 101 | 0.00 | 0.9787 | 0.9993 |
|  | 2 Reliving events in mind | C | 93 | 0.01 | 0.8474 | 0.9350 | 13 | 0.07 | 0.7362 | 0.8887 | 100 | 0.04 | 0.5258 | 0.7646 |
|  | 3 Avoiding thoughts | C | 92 | 0.15 | 0.0324 | 0.1817 | 13 | -0.04 | 0.8613 | 0.9405 | 98 | 0.04 | 0.5166 | 0.7574 |
|  | 4 Avoiding physically | C | 92 | -0.03 | 0.6502 | 0.8413 | 13 | -0.12 | 0.5737 | 0.7947 | 98 | 0.15 | 0.0280 | 0.1697 |
|  | 5 Overly cautious | C | 91 | 0.03 | 0.6396 | 0.8362 | 13 | 0.29 | 0.1739 | 0.4408 | 99 | 0.04 | 0.5365 | 0.7712 |
|  | 6 Nervousness | C | 92 | -0.12 | 0.1031 | 0.3436 | 13 | 0.07 | 0.7467 | 0.8928 | 101 | -0.05 | 0.4414 | 0.7014 |
|  | 7 Calming difficulty | C | 91 | 0.09 | 0.1930 | 0.4631 | 13 | 0.07 | 0.7447 | 0.8918 | 100 | -0.02 | 0.7343 | 0.8884 |
|  | 8 Emotional numbness | C | 91 | -0.09 | 0.2327 | 0.5089 | 13 | 0.02 | 0.9304 | 0.9748 | 99 | 0.04 | 0.5205 | 0.7601 |
|  | 9 Sense of failure | C | 91 | -0.02 | 0.8107 | 0.9242 | 13 | 0.23 | 0.2777 | 0.5528 | 101 | 0.04 | 0.6008 | 0.8155 |
|  | 10 Self-doubt | C | 92 | -0.03 | 0.7167 | 0.8811 | 13 | 0.22 | 0.2916 | 0.5668 | 100 | -0.17 | 0.0118 | 0.1066 |
|  | 11 Disconnection to others | C | 92 | -0.09 | 0.1854 | 0.4535 | 13 | 0.11 | 0.5932 | 0.8080 | 100 | 0.12 | 0.0858 | 0.3116 |
|  | 12 Social difficulty | C | 91 | 0.00 | 0.9882 | 1.0000 | 13 | -0.11 | 0.6002 | 0.8149 | 101 | 0.05 | 0.4374 | 0.6985 |
| 15 Sleep quality | 1 Bad dreams | A | 94 | 0.06 | 0.3925 | 0.6605 | 13 | 0.18 | 0.3903 | 0.6589 | 100 | 0.00 | 0.9826 | 1.0000 |
|  | 2 Reliving events in mind | A | 94 | 0.02 | 0.7249 | 0.8841 | 13 | 0.59 | 0.0052 | 0.0626 | 99 | -0.03 | 0.6975 | 0.8704 |
|  | 3 Avoiding thoughts | A | 93 | 0.05 | 0.5078 | 0.7502 | 13 | 0.30 | 0.1470 | 0.4047 | 97 | -0.07 | 0.2892 | 0.5637 |
|  | 4 Avoiding physically | A | 93 | -0.07 | 0.3462 | 0.6210 | 13 | 0.33 | 0.1195 | 0.3686 | 97 | -0.07 | 0.3178 | 0.5932 |
|  | 5 Overly cautious | A | 92 | 0.04 | 0.5871 | 0.8047 | 13 | 0.39 | 0.0640 | 0.2648 | 98 | -0.07 | 0.2739 | 0.5496 |
|  | 6 Nervousness | A | 93 | -0.21 | 0.0027 | 0.0421 | 13 | 0.30 | 0.1542 | 0.4144 | 100 | -0.11 | 0.1159 | 0.3656 |
|  | 7 Calming difficulty | A | 92 | -0.05 | 0.4981 | 0.7449 | 13 | 0.05 | 0.7999 | 0.9192 | 99 | -0.11 | 0.1137 | 0.3613 |
|  | 8 Emotional numbness | A | 92 | 0.03 | 0.6891 | 0.8645 | 13 | 0.04 | 0.8561 | 0.9402 | 98 | -0.04 | 0.5235 | 0.7626 |
|  | 9 Sense of failure | A | 92 | -0.07 | 0.2926 | 0.5679 | 13 | 0.22 | 0.2984 | 0.5753 | 100 | -0.18 | 0.0088 | 0.0890 |
|  | 10 Self-doubt | A | 93 | 0.10 | 0.1746 | 0.4408 | 13 | -0.31 | 0.1356 | 0.3910 | 99 | -0.17 | 0.0126 | 0.1090 |
|  | 11 Disconnection to others | A | 93 | -0.08 | 0.2378 | 0.5148 | 13 | 0.33 | 0.1162 | 0.3656 | 99 | -0.07 | 0.2916 | 0.5668 |
|  | 12 Social difficulty | A | 92 | 0.06 | 0.4290 | 0.6920 | 13 | -0.13 | 0.5257 | 0.7646 | 100 | -0.07 | 0.2815 | 0.5578 |
| 16 Tiredness level | 1 Bad dreams | A | 93 | 0.07 | 0.3051 | 0.5821 | 13 | 0.13 | 0.5437 | 0.7766 | 99 | -0.05 | 0.4827 | 0.7368 |
|  | 2 Reliving events in mind | A | 93 | -0.15 | 0.0379 | 0.1972 | 13 | 0.50 | 0.0172 | 0.1306 | 98 | -0.05 | 0.4866 | 0.7382 |
|  | 3 Avoiding thoughts | A | 92 | 0.14 | 0.0413 | 0.2090 | 13 | 0.31 | 0.1431 | 0.3993 | 96 | -0.08 | 0.2648 | 0.5437 |
|  | 4 Avoiding physically | A | 92 | 0.03 | 0.6450 | 0.8394 | 13 | 0.25 | 0.2384 | 0.5154 | 96 | -0.07 | 0.3056 | 0.5824 |
|  | 5 Overly cautious | A | 91 | -0.07 | 0.3217 | 0.5983 | 13 | 0.51 | 0.0162 | 0.1274 | 97 | -0.11 | 0.1152 | 0.3644 |
|  | 6 Nervousness | A | 92 | -0.04 | 0.5569 | 0.7821 | 13 | -0.12 | 0.5536 | 0.7815 | 99 | -0.15 | 0.0247 | 0.1602 |
|  | 7 Calming difficulty | A | 91 | 0.01 | 0.8911 | 0.9564 | 13 | -0.02 | 0.9320 | 0.9748 | 98 | -0.13 | 0.0534 | 0.2377 |
|  | 8 Emotional numbness | A | 91 | -0.04 | 0.5457 | 0.7767 | 13 | -0.08 | 0.7143 | 0.8798 | 97 | 0.01 | 0.8520 | 0.9373 |
|  | 9 Sense of failure | A | 91 | -0.02 | 0.7266 | 0.8841 | 13 | 0.13 | 0.5402 | 0.7732 | 99 | -0.13 | 0.0510 | 0.2326 |
|  | 10 Self-doubt | A | 92 | -0.05 | 0.4417 | 0.7016 | 13 | -0.06 | 0.7631 | 0.9000 | 98 | -0.18 | 0.0094 | 0.0923 |
|  | 11 Disconnection to others | A | 92 | -0.03 | 0.6465 | 0.8395 | 13 | 0.04 | 0.8520 | 0.9373 | 98 | -0.07 | 0.3336 | 0.6097 |
|  | 12 Social difficulty | A | 91 | 0.14 | 0.0419 | 0.2107 | 13 | -0.23 | 0.2720 | 0.5491 | 99 | -0.10 | 0.1404 | 0.3963 |
| 17 Eating attitude | 1 Bad dreams | A | 92 | 0.14 | 0.0566 | 0.2460 | 13 | 0.61 | 0.0038 | 0.0516 | 100 | -0.13 | 0.0522 | 0.2360 |
|  | 2 Reliving events in mind | A | 92 | -0.03 | 0.6583 | 0.8474 | 13 | 0.38 | 0.0710 | 0.2806 | 99 | -0.14 | 0.0442 | 0.2158 |
|  | 3 Avoiding thoughts | A | 91 | 0.11 | 0.1177 | 0.3671 | 13 | 0.60 | 0.0043 | 0.0557 | 97 | -0.19 | 0.0067 | 0.0744 |
|  | 4 Avoiding physically | A | 91 | 0.13 | 0.0663 | 0.2706 | 13 | 0.49 | 0.0200 | 0.1406 | 97 | -0.10 | 0.1547 | 0.4146 |
|  | 5 Overly cautious | A | 90 | 0.07 | 0.3315 | 0.6078 | 13 | 0.77 | 0.0003 | 0.0097 | 98 | -0.11 | 0.1070 | 0.3506 |
|  | 6 Nervousness | A | 91 | 0.05 | 0.4494 | 0.7081 | 13 | 0.34 | 0.1010 | 0.3404 | 100 | -0.27 | 0.0001 | 0.0042 |
|  | 7 Calming difficulty | A | 90 | 0.04 | 0.5455 | 0.7767 | 13 | 0.44 | 0.0346 | 0.1870 | 99 | -0.14 | 0.0345 | 0.1870 |
|  | 8 Emotional numbness | A | 90 | 0.05 | 0.4485 | 0.7072 | 13 | 0.43 | 0.0385 | 0.1993 | 98 | -0.17 | 0.0160 | 0.1263 |
|  | 9 Sense of failure | A | 90 | 0.01 | 0.8604 | 0.9403 | 13 | -0.08 | 0.7063 | 0.8762 | 100 | -0.25 | 0.0003 | 0.0102 |
|  | 10 Self-doubt | A | 91 | 0.10 | 0.1735 | 0.4408 | 13 | 0.18 | 0.3869 | 0.6557 | 99 | -0.18 | 0.0078 | 0.0821 |
|  | 11 Disconnection to others | A | 91 | 0.09 | 0.1985 | 0.4696 | 13 | 0.00 | 1.0000 | 1.0000 | 99 | -0.14 | 0.0438 | 0.2153 |
|  | 12 Social difficulty | A | 90 | 0.19 | 0.0065 | 0.0737 | 13 | -0.06 | 0.7675 | 0.9031 | 100 | -0.09 | 0.1771 | 0.4434 |
| 18 Pain thoughts | 1 Bad dreams | A | 92 | -0.04 | 0.5495 | 0.7783 | 13 | 0.04 | 0.8364 | 0.9327 | 97 | -0.29 | <0.0001 | 0.0020 |
|  | 2 Reliving events in mind | A | 92 | 0.05 | 0.4927 | 0.7423 | 13 | 0.11 | 0.5994 | 0.8142 | 96 | -0.30 | <0.0001 | 0.0018 |
|  | 3 Avoiding thoughts | A | 91 | -0.01 | 0.8810 | 0.9504 | 13 | 0.14 | 0.5133 | 0.7548 | 94 | -0.33 | <0.0001 | 0.0008 |
|  | 4 Avoiding physically | A | 91 | 0.15 | 0.0337 | 0.1852 | 13 | 0.49 | 0.0193 | 0.1384 | 94 | -0.25 | 0.0003 | 0.0108 |
|  | 5 Overly cautious | A | 90 | -0.06 | 0.3827 | 0.6534 | 13 | 0.24 | 0.2437 | 0.5213 | 95 | -0.24 | 0.0007 | 0.0175 |
|  | 6 Nervousness | A | 91 | 0.09 | 0.2104 | 0.4849 | 13 | 0.17 | 0.4204 | 0.6851 | 97 | -0.24 | 0.0006 | 0.0171 |
|  | 7 Calming difficulty | A | 90 | 0.10 | 0.1760 | 0.4418 | 13 | 0.30 | 0.1551 | 0.4149 | 96 | -0.12 | 0.0815 | 0.3012 |
|  | 8 Emotional numbness | A | 90 | 0.02 | 0.7452 | 0.8919 | 13 | 0.23 | 0.2760 | 0.5511 | 95 | -0.14 | 0.0499 | 0.2293 |
|  | 9 Sense of failure | A | 90 | 0.00 | 0.9550 | 0.9861 | 13 | 0.18 | 0.4048 | 0.6700 | 97 | -0.11 | 0.1105 | 0.3559 |
|  | 10 Self-doubt | A | 91 | -0.11 | 0.1139 | 0.3613 | 13 | -0.08 | 0.7198 | 0.8824 | 97 | -0.13 | 0.0542 | 0.2394 |
|  | 11 Disconnection to others | A | 91 | 0.06 | 0.4390 | 0.6995 | 13 | 0.07 | 0.7390 | 0.8895 | 96 | -0.08 | 0.2522 | 0.5314 |
|  | 12 Social difficulty | A | 90 | -0.10 | 0.1595 | 0.4224 | 13 | 0.23 | 0.2760 | 0.5511 | 97 | -0.08 | 0.2376 | 0.5148 |
| 19 Loneliness feeling | 1 Bad dreams | D | 90 | 0.10 | 0.1635 | 0.4272 | 13 | 0.20 | 0.3391 | 0.6148 | 100 | -0.13 | 0.0531 | 0.2374 |
|  | 2 Reliving events in mind | D | 90 | -0.11 | 0.1104 | 0.3559 | 13 | 0.55 | 0.0086 | 0.0879 | 99 | -0.12 | 0.0905 | 0.3215 |
|  | 3 Avoiding thoughts | D | 89 | 0.08 | 0.2521 | 0.5314 | 13 | 0.34 | 0.1066 | 0.3502 | 97 | -0.12 | 0.0740 | 0.2883 |
|  | 4 Avoiding physically | D | 89 | 0.06 | 0.4074 | 0.6718 | 13 | 0.46 | 0.0304 | 0.1771 | 97 | -0.10 | 0.1545 | 0.4146 |
|  | 5 Overly cautious | D | 88 | -0.09 | 0.2000 | 0.4715 | 13 | 0.45 | 0.0309 | 0.1773 | 98 | -0.10 | 0.1336 | 0.3886 |
|  | 6 Nervousness | D | 89 | -0.04 | 0.5561 | 0.7819 | 13 | 0.16 | 0.4556 | 0.7146 | 100 | -0.21 | 0.0024 | 0.0392 |
|  | 7 Calming difficulty | D | 88 | 0.02 | 0.7914 | 0.9160 | 13 | 0.18 | 0.3975 | 0.6656 | 99 | -0.10 | 0.1303 | 0.3832 |
|  | 8 Emotional numbness | D | 88 | -0.01 | 0.8821 | 0.9513 | 13 | 0.32 | 0.1303 | 0.3832 | 98 | -0.14 | 0.0361 | 0.1926 |
|  | 9 Sense of failure | D | 89 | -0.07 | 0.3565 | 0.6307 | 13 | 0.26 | 0.2101 | 0.4849 | 100 | -0.21 | 0.0016 | 0.0306 |
|  | 10 Self-doubt | D | 89 | -0.06 | 0.3890 | 0.6579 | 13 | 0.14 | 0.5066 | 0.7497 | 99 | -0.19 | 0.0043 | 0.0561 |
|  | 11 Disconnection to others | D | 89 | -0.08 | 0.2743 | 0.5498 | 13 | 0.15 | 0.4717 | 0.7282 | 99 | -0.06 | 0.4008 | 0.6678 |
|  | 12 Social difficulty | D | 88 | 0.11 | 0.1168 | 0.3656 | 13 | 0.08 | 0.6866 | 0.8628 | 100 | -0.12 | 0.0672 | 0.2723 |
| 20 School fun | 1 Bad dreams | C | 88 | 0.15 | 0.0399 | 0.2042 | 13 | 0.38 | 0.0712 | 0.2807 | 99 | -0.10 | 0.1309 | 0.3834 |
|  | 2 Reliving events in mind | C | 88 | -0.09 | 0.1956 | 0.4653 | 13 | 0.50 | 0.0183 | 0.1348 | 98 | -0.14 | 0.0375 | 0.1959 |
|  | 3 Avoiding thoughts | C | 87 | 0.03 | 0.6338 | 0.8355 | 13 | 0.51 | 0.0144 | 0.1179 | 96 | -0.19 | 0.0061 | 0.0707 |
|  | 4 Avoiding physically | C | 87 | 0.16 | 0.0265 | 0.1643 | 13 | 0.14 | 0.4958 | 0.7437 | 96 | -0.17 | 0.0137 | 0.1141 |
|  | 5 Overly cautious | C | 86 | -0.16 | 0.0313 | 0.1782 | 13 | 0.04 | 0.8600 | 0.9402 | 97 | -0.17 | 0.0150 | 0.1212 |
|  | 6 Nervousness | C | 87 | -0.12 | 0.0967 | 0.3328 | 13 | 0.04 | 0.8669 | 0.9443 | 99 | -0.31 | <0.0001 | 0.0011 |
|  | 7 Calming difficulty | C | 86 | 0.09 | 0.2173 | 0.4904 | 13 | 0.21 | 0.3106 | 0.5874 | 98 | -0.26 | 0.0002 | 0.0075 |
|  | 8 Emotional numbness | C | 86 | 0.01 | 0.9438 | 0.9803 | 13 | 0.32 | 0.1233 | 0.3736 | 97 | -0.16 | 0.0173 | 0.1306 |
|  | 9 Sense of failure | C | 86 | 0.07 | 0.3094 | 0.5859 | 13 | -0.07 | 0.7289 | 0.8853 | 99 | -0.33 | <0.0001 | 0.0008 |
|  | 10 Self-doubt | C | 87 | 0.00 | 0.9786 | 0.9993 | 13 | 0.04 | 0.8423 | 0.9329 | 98 | -0.26 | 0.0002 | 0.0072 |
|  | 11 Disconnection to others | C | 87 | 0.05 | 0.5318 | 0.7689 | 13 | -0.06 | 0.7816 | 0.9110 | 98 | -0.16 | 0.0170 | 0.1302 |
|  | 12 Social difficulty | C | 86 | -0.03 | 0.6350 | 0.8355 | 13 | 0.13 | 0.5257 | 0.7646 | 99 | -0.14 | 0.0353 | 0.1890 |
| 21 Friends | 1 Bad dreams | D | 92 | 0.04 | 0.5849 | 0.8033 | 13 | 0.46 | 0.0299 | 0.1757 | 99 | 0.01 | 0.8299 | 0.9297 |
|  | 2 Reliving events in mind | D | 92 | -0.15 | 0.0367 | 0.1939 | 13 | 0.21 | 0.3078 | 0.5841 | 98 | -0.14 | 0.0484 | 0.2253 |
|  | 3 Avoiding thoughts | D | 91 | 0.14 | 0.0519 | 0.2354 | 13 | 0.43 | 0.0426 | 0.2123 | 96 | -0.10 | 0.1696 | 0.4367 |
|  | 4 Avoiding physically | D | 91 | 0.07 | 0.3306 | 0.6074 | 13 | 0.42 | 0.0469 | 0.2227 | 96 | -0.05 | 0.4906 | 0.7410 |
|  | 5 Overly cautious | D | 90 | -0.14 | 0.0532 | 0.2374 | 13 | 0.50 | 0.0164 | 0.1275 | 97 | -0.08 | 0.2734 | 0.5496 |
|  | 6 Nervousness | D | 91 | -0.18 | 0.0132 | 0.1112 | 13 | 0.34 | 0.1031 | 0.3436 | 99 | -0.19 | 0.0046 | 0.0581 |
|  | 7 Calming difficulty | D | 90 | -0.07 | 0.3243 | 0.6013 | 13 | 0.36 | 0.0845 | 0.3081 | 98 | -0.17 | 0.0112 | 0.1040 |
|  | 8 Emotional numbness | D | 90 | -0.06 | 0.4252 | 0.6902 | 13 | 0.33 | 0.1126 | 0.3588 | 97 | -0.10 | 0.1583 | 0.4207 |
|  | 9 Sense of failure | D | 90 | -0.01 | 0.8843 | 0.9527 | 13 | 0.11 | 0.6132 | 0.8237 | 99 | -0.22 | 0.0011 | 0.0235 |
|  | 10 Self-doubt | D | 91 | -0.05 | 0.4861 | 0.7382 | 13 | 0.16 | 0.4390 | 0.6995 | 98 | -0.18 | 0.0070 | 0.0768 |
|  | 11 Disconnection to others | D | 91 | -0.02 | 0.7571 | 0.8969 | 13 | 0.08 | 0.7193 | 0.8824 | 98 | -0.02 | 0.7281 | 0.8850 |
|  | 12 Social difficulty | D | 90 | 0.01 | 0.8976 | 0.9599 | 13 | 0.11 | 0.5969 | 0.8115 | 99 | -0.02 | 0.8049 | 0.9214 |
| 22 Dealing with school tasks | 1 Bad dreams | C | 89 | 0.11 | 0.1370 | 0.3923 | 13 | 0.33 | 0.1184 | 0.3679 | 98 | -0.03 | 0.6767 | 0.8577 |
|  | 2 Reliving events in mind | C | 89 | -0.02 | 0.7835 | 0.9118 | 13 | 0.56 | 0.0081 | 0.0842 | 97 | 0.00 | 0.9471 | 0.9824 |
|  | 3 Avoiding thoughts | C | 88 | 0.18 | 0.0142 | 0.1170 | 13 | 0.58 | 0.0060 | 0.0707 | 95 | 0.00 | 0.9875 | 1.0000 |
|  | 4 Avoiding physically | C | 88 | 0.27 | 0.0002 | 0.0079 | 13 | 0.47 | 0.0238 | 0.1583 | 95 | -0.05 | 0.5178 | 0.7583 |
|  | 5 Overly cautious | C | 87 | -0.10 | 0.1726 | 0.4398 | 13 | 0.60 | 0.0044 | 0.0563 | 96 | -0.02 | 0.8080 | 0.9228 |
|  | 6 Nervousness | C | 88 | -0.03 | 0.7040 | 0.8749 | 13 | -0.04 | 0.8656 | 0.9435 | 98 | -0.08 | 0.2151 | 0.4876 |
|  | 7 Calming difficulty | C | 87 | 0.14 | 0.0524 | 0.2364 | 13 | 0.16 | 0.4426 | 0.7023 | 97 | -0.04 | 0.5933 | 0.8080 |
|  | 8 Emotional numbness | C | 87 | 0.13 | 0.0832 | 0.3052 | 13 | 0.00 | 1.0000 | 1.0000 | 96 | 0.03 | 0.6153 | 0.8243 |
|  | 9 Sense of failure | C | 87 | 0.11 | 0.1218 | 0.3723 | 13 | 0.04 | 0.8611 | 0.9404 | 98 | -0.07 | 0.2833 | 0.5585 |
|  | 10 Self-doubt | C | 88 | 0.10 | 0.1848 | 0.4535 | 13 | -0.13 | 0.5467 | 0.7767 | 97 | -0.15 | 0.0334 | 0.1848 |
|  | 11 Disconnection to others | C | 88 | 0.07 | 0.3054 | 0.5824 | 13 | 0.00 | 1.0000 | 1.0000 | 97 | -0.01 | 0.8652 | 0.9433 |
|  | 12 Social difficulty | C | 87 | 0.10 | 0.1748 | 0.4408 | 13 | 0.10 | 0.6472 | 0.8398 | 98 | 0.02 | 0.7947 | 0.9175 |
| 23 Self-comparison to others | 1 Bad dreams | C | 92 | 0.04 | 0.5663 | 0.7892 | 13 | 0.41 | 0.0539 | 0.2392 | 101 | -0.13 | 0.0611 | 0.2567 |
|  | 2 Reliving events in mind | C | 92 | -0.12 | 0.0840 | 0.3072 | 13 | 0.07 | 0.7214 | 0.8829 | 100 | -0.05 | 0.4839 | 0.7375 |
|  | 3 Avoiding thoughts | C | 91 | 0.05 | 0.4472 | 0.7064 | 13 | 0.49 | 0.0208 | 0.1455 | 98 | -0.11 | 0.1216 | 0.3723 |
|  | 4 Avoiding physically | C | 91 | -0.02 | 0.7945 | 0.9175 | 13 | 0.33 | 0.1124 | 0.3588 | 98 | -0.05 | 0.5050 | 0.7485 |
|  | 5 Overly cautious | C | 90 | -0.10 | 0.1794 | 0.4465 | 13 | 0.55 | 0.0091 | 0.0904 | 99 | -0.03 | 0.7138 | 0.8798 |
|  | 6 Nervousness | C | 91 | -0.12 | 0.0797 | 0.2986 | 13 | 0.22 | 0.3051 | 0.5821 | 101 | -0.13 | 0.0583 | 0.2502 |
|  | 7 Calming difficulty | C | 90 | -0.03 | 0.6468 | 0.8395 | 13 | 0.62 | 0.0034 | 0.0478 | 100 | -0.11 | 0.0969 | 0.3328 |
|  | 8 Emotional numbness | C | 90 | -0.11 | 0.1316 | 0.3849 | 13 | 0.52 | 0.0126 | 0.1090 | 99 | -0.06 | 0.3605 | 0.6335 |
|  | 9 Sense of failure | C | 90 | -0.02 | 0.7579 | 0.8973 | 13 | 0.19 | 0.3769 | 0.6485 | 101 | -0.18 | 0.0067 | 0.0747 |
|  | 10 Self-doubt | C | 91 | -0.08 | 0.2433 | 0.5211 | 13 | 0.53 | 0.0112 | 0.1040 | 100 | -0.16 | 0.0166 | 0.1283 |
|  | 11 Disconnection to others | C | 91 | -0.04 | 0.5898 | 0.8064 | 13 | 0.18 | 0.3964 | 0.6647 | 100 | -0.03 | 0.6448 | 0.8394 |
|  | 12 Social difficulty | C | 90 | 0.07 | 0.3464 | 0.6210 | 13 | 0.52 | 0.0126 | 0.1090 | 101 | -0.03 | 0.6150 | 0.8243 |
| 24 Love-awareness | 1 Bad dreams | B | 94 | 0.15 | 0.0311 | 0.1781 | 13 | 0.29 | 0.1703 | 0.4367 | 100 | -0.28 | <0.0001 | 0.0031 |
|  | 2 Reliving events in mind | B | 94 | -0.06 | 0.3706 | 0.6425 | 13 | 0.67 | 0.0014 | 0.0283 | 99 | -0.16 | 0.0194 | 0.1384 |
|  | 3 Avoiding thoughts | B | 93 | 0.01 | 0.8407 | 0.9329 | 13 | 0.61 | 0.0038 | 0.0517 | 97 | -0.23 | 0.0008 | 0.0204 |
|  | 4 Avoiding physically | B | 93 | 0.08 | 0.2758 | 0.5511 | 13 | 0.70 | 0.0009 | 0.0207 | 97 | -0.12 | 0.0886 | 0.3170 |
|  | 5 Overly cautious | B | 92 | -0.05 | 0.4646 | 0.7224 | 13 | 0.61 | 0.0035 | 0.0489 | 98 | -0.08 | 0.2308 | 0.5064 |
|  | 6 Nervousness | B | 93 | -0.06 | 0.3838 | 0.6546 | 13 | -0.08 | 0.7023 | 0.8742 | 100 | -0.17 | 0.0119 | 0.1066 |
|  | 7 Calming difficulty | B | 92 | 0.03 | 0.6481 | 0.8407 | 13 | 0.06 | 0.7726 | 0.9054 | 99 | -0.12 | 0.0694 | 0.2767 |
|  | 8 Emotional numbness | B | 92 | 0.02 | 0.7694 | 0.9034 | 13 | 0.00 | 1.0000 | 1.0000 | 98 | -0.16 | 0.0216 | 0.1495 |
|  | 9 Sense of failure | B | 92 | 0.03 | 0.6710 | 0.8541 | 13 | -0.08 | 0.6927 | 0.8667 | 100 | -0.26 | 0.0001 | 0.0065 |
|  | 10 Self-doubt | B | 93 | 0.01 | 0.9302 | 0.9748 | 13 | -0.31 | 0.1403 | 0.3963 | 99 | -0.19 | 0.0055 | 0.0660 |
|  | 11 Disconnection to others | B | 93 | 0.07 | 0.3364 | 0.6128 | 13 | -0.18 | 0.3993 | 0.6665 | 99 | -0.10 | 0.1266 | 0.3796 |
|  | 12 Social difficulty | B | 92 | 0.09 | 0.1831 | 0.4512 | 13 | 0.00 | 1.0000 | 1.0000 | 100 | -0.11 | 0.1047 | 0.3472 |
| 25 Peer arguing | 1 Bad dreams | D | 94 | -0.03 | 0.6512 | 0.8421 | 13 | 0.52 | 0.0132 | 0.1111 | 100 | -0.09 | 0.1638 | 0.4274 |
|  | 2 Reliving events in mind | D | 94 | -0.15 | 0.0305 | 0.1771 | 13 | 0.53 | 0.0117 | 0.1059 | 99 | -0.12 | 0.0783 | 0.2962 |
|  | 3 Avoiding thoughts | D | 93 | 0.08 | 0.2594 | 0.5368 | 13 | 0.55 | 0.0089 | 0.0894 | 97 | -0.11 | 0.1054 | 0.3484 |
|  | 4 Avoiding physically | D | 93 | -0.01 | 0.9155 | 0.9682 | 13 | 0.61 | 0.0035 | 0.0481 | 97 | -0.09 | 0.1870 | 0.4553 |
|  | 5 Overly cautious | D | 92 | -0.13 | 0.0595 | 0.2536 | 13 | 0.62 | 0.0030 | 0.0440 | 98 | -0.09 | 0.1811 | 0.4484 |
|  | 6 Nervousness | D | 93 | -0.19 | 0.0061 | 0.0711 | 13 | 0.34 | 0.1070 | 0.3506 | 100 | -0.16 | 0.0191 | 0.1375 |
|  | 7 Calming difficulty | D | 92 | 0.02 | 0.7614 | 0.8994 | 13 | 0.30 | 0.1551 | 0.4149 | 99 | -0.05 | 0.4579 | 0.7166 |
|  | 8 Emotional numbness | D | 92 | -0.02 | 0.7555 | 0.8966 | 13 | 0.43 | 0.0385 | 0.1992 | 98 | -0.23 | 0.0010 | 0.0221 |
|  | 9 Sense of failure | D | 92 | 0.05 | 0.5125 | 0.7538 | 13 | -0.04 | 0.8350 | 0.9318 | 100 | -0.21 | 0.0016 | 0.0305 |
|  | 10 Self-doubt | D | 93 | -0.01 | 0.8626 | 0.9413 | 13 | 0.10 | 0.6325 | 0.8345 | 99 | -0.20 | 0.0027 | 0.0422 |
|  | 11 Disconnection to others | D | 93 | 0.05 | 0.5215 | 0.7611 | 13 | -0.05 | 0.8242 | 0.9290 | 99 | -0.10 | 0.1630 | 0.4268 |
|  | 12 Social difficulty | D | 92 | 0.16 | 0.0238 | 0.1583 | 13 | -0.07 | 0.7438 | 0.8912 | 100 | -0.09 | 0.1709 | 0.4373 |
| 26 Napping/dozing | 1 Bad dreams | A | 93 | 0.04 | 0.5288 | 0.7679 | 13 | 0.04 | 0.8484 | 0.9350 | 101 | -0.09 | 0.1783 | 0.4454 |
|  | 2 Reliving events in mind | A | 93 | -0.10 | 0.1392 | 0.3957 | 13 | 0.67 | 0.0013 | 0.0270 | 100 | -0.12 | 0.0878 | 0.3160 |
|  | 3 Avoiding thoughts | A | 92 | 0.04 | 0.5558 | 0.7819 | 13 | 0.34 | 0.1066 | 0.3502 | 98 | -0.18 | 0.0106 | 0.1007 |
|  | 4 Avoiding physically | A | 92 | 0.07 | 0.3405 | 0.6160 | 13 | 0.68 | 0.0012 | 0.0249 | 98 | -0.07 | 0.3169 | 0.5927 |
|  | 5 Overly cautious | A | 91 | 0.01 | 0.9381 | 0.9762 | 13 | 0.45 | 0.0309 | 0.1773 | 99 | -0.05 | 0.4261 | 0.6905 |
|  | 6 Nervousness | A | 92 | -0.02 | 0.7285 | 0.8850 | 13 | 0.00 | 1.0000 | 1.0000 | 101 | -0.13 | 0.0605 | 0.2555 |
|  | 7 Calming difficulty | A | 91 | 0.05 | 0.5001 | 0.7458 | 13 | -0.24 | 0.2592 | 0.5368 | 100 | -0.02 | 0.8089 | 0.9228 |
|  | 8 Emotional numbness | A | 91 | 0.00 | 1.0000 | 1.0000 | 13 | -0.11 | 0.6140 | 0.8239 | 99 | -0.03 | 0.6808 | 0.8599 |
|  | 9 Sense of failure | A | 91 | -0.04 | 0.5994 | 0.8142 | 13 | -0.10 | 0.6297 | 0.8331 | 101 | -0.21 | 0.0017 | 0.0315 |
|  | 10 Self-doubt | A | 92 | -0.02 | 0.8273 | 0.9295 | 13 | -0.54 | 0.0109 | 0.1020 | 100 | -0.11 | 0.1021 | 0.3421 |
|  | 11 Disconnection to others | A | 92 | -0.07 | 0.3031 | 0.5804 | 13 | -0.04 | 0.8371 | 0.9327 | 100 | -0.03 | 0.6367 | 0.8355 |
|  | 12 Social difficulty | A | 91 | 0.15 | 0.0296 | 0.1754 | 13 | -0.11 | 0.6140 | 0.8239 | 101 | -0.05 | 0.4274 | 0.6911 |
| 27 Eating problems | 1 Bad dreams | A | 94 | -0.02 | 0.8160 | 0.9273 | 13 | 0.37 | 0.0760 | 0.2919 | 97 | -0.05 | 0.5009 | 0.7458 |
|  | 2 Reliving events in mind | A | 94 | -0.14 | 0.0455 | 0.2194 | 13 | 0.48 | 0.0226 | 0.1534 | 97 | 0.05 | 0.4715 | 0.7281 |
|  | 3 Avoiding thoughts | A | 93 | 0.13 | 0.0675 | 0.2732 | 13 | 0.54 | 0.0104 | 0.0994 | 95 | -0.05 | 0.4961 | 0.7437 |
|  | 4 Avoiding physically | A | 93 | 0.09 | 0.2208 | 0.4938 | 13 | 0.38 | 0.0721 | 0.2835 | 95 | 0.03 | 0.6373 | 0.8355 |
|  | 5 Overly cautious | A | 92 | -0.04 | 0.5968 | 0.8115 | 13 | 0.36 | 0.0844 | 0.3081 | 96 | -0.01 | 0.8393 | 0.9329 |
|  | 6 Nervousness | A | 93 | -0.07 | 0.2874 | 0.5622 | 13 | 0.00 | 1.0000 | 1.0000 | 97 | -0.07 | 0.2790 | 0.5544 |
|  | 7 Calming difficulty | A | 92 | 0.07 | 0.2978 | 0.5743 | 13 | 0.25 | 0.2324 | 0.5088 | 96 | -0.04 | 0.5744 | 0.7951 |
|  | 8 Emotional numbness | A | 92 | -0.01 | 0.8687 | 0.9452 | 13 | 0.33 | 0.1149 | 0.3641 | 95 | 0.01 | 0.8484 | 0.9350 |
|  | 9 Sense of failure | A | 92 | 0.11 | 0.1194 | 0.3686 | 13 | -0.12 | 0.5719 | 0.7928 | 97 | -0.21 | 0.0027 | 0.0422 |
|  | 10 Self-doubt | A | 93 | 0.00 | 0.9595 | 0.9888 | 13 | 0.05 | 0.8288 | 0.9297 | 96 | -0.20 | 0.0038 | 0.0516 |
|  | 11 Disconnection to others | A | 93 | 0.12 | 0.0767 | 0.2941 | 13 | -0.11 | 0.6155 | 0.8243 | 96 | -0.15 | 0.0293 | 0.1748 |
|  | 12 Social difficulty | A | 92 | 0.22 | 0.0020 | 0.0346 | 13 | 0.12 | 0.5543 | 0.7815 | 97 | -0.04 | 0.5893 | 0.8061 |
| 28 Memorization | 1 Bad dreams | C | 94 | 0.10 | 0.1602 | 0.4237 | 13 | 0.25 | 0.2422 | 0.5195 | 101 | -0.16 | 0.0182 | 0.1343 |
|  | 2 Reliving events in mind | C | 94 | -0.03 | 0.7203 | 0.8824 | 13 | 0.36 | 0.0892 | 0.3184 | 100 | -0.09 | 0.1659 | 0.4306 |
|  | 3 Avoiding thoughts | C | 93 | 0.10 | 0.1494 | 0.4072 | 13 | 0.31 | 0.1340 | 0.3889 | 98 | -0.15 | 0.0273 | 0.1670 |
|  | 4 Avoiding physically | C | 93 | 0.06 | 0.3606 | 0.6335 | 13 | 0.54 | 0.0106 | 0.1008 | 98 | -0.06 | 0.3464 | 0.6210 |
|  | 5 Overly cautious | C | 92 | -0.04 | 0.5305 | 0.7684 | 13 | 0.54 | 0.0101 | 0.0970 | 99 | -0.09 | 0.1636 | 0.4272 |
|  | 6 Nervousness | C | 93 | -0.02 | 0.7838 | 0.9118 | 13 | 0.39 | 0.0609 | 0.2563 | 101 | -0.20 | 0.0039 | 0.0520 |
|  | 7 Calming difficulty | C | 92 | 0.25 | 0.0004 | 0.0120 | 13 | 0.43 | 0.0400 | 0.2042 | 100 | -0.18 | 0.0075 | 0.0804 |
|  | 8 Emotional numbness | C | 92 | 0.11 | 0.1140 | 0.3615 | 13 | 0.44 | 0.0344 | 0.1870 | 99 | -0.09 | 0.2005 | 0.4717 |
|  | 9 Sense of failure | C | 92 | 0.13 | 0.0698 | 0.2781 | 13 | 0.35 | 0.0920 | 0.3238 | 101 | -0.24 | 0.0003 | 0.0105 |
|  | 10 Self-doubt | C | 93 | 0.00 | 0.9511 | 0.9838 | 13 | 0.28 | 0.1756 | 0.4415 | 100 | -0.23 | 0.0005 | 0.0151 |
|  | 11 Disconnection to others | C | 93 | -0.01 | 0.9175 | 0.9693 | 13 | 0.15 | 0.4723 | 0.7286 | 100 | -0.15 | 0.0286 | 0.1723 |
|  | 12 Social difficulty | C | 92 | 0.09 | 0.2226 | 0.4961 | 13 | 0.20 | 0.3323 | 0.6085 | 101 | -0.15 | 0.0261 | 0.1643 |

B-H – Benjamini-Hochberg correction for multiple comparisons, p-values < 0.05 are indicated in red, N – number of participants, Tau – Tau correlation coefficient
