# Supplementary material for: Demographic predictors of trauma and depression in war-affected children from Poland and Ukraine: Implications for prevention of mental health problems
Source: Prev Med Rep. 2026 Feb 20;63:103422. doi: 10.1016/j.pmedr.2026.103422 (PMC12945576; doi:10.1016/j.pmedr.2026.103422)
Supplement: Supplementary file 5 — Supplementary material 5 [file mmc5.docx]

**CDI (panels A-D) and ITQ-CA correlations concerning return-home reason dependent groups**

**CDI2 – group A**

In CDI2 – group A among children for whom the reason to return to the home country was not applicable (Polish citizens or Ukrainian-born in Poland), we observed 26 significant, very weak to moderate correlations (14 negative and 12 positive and strong) between data obtained from CDI2 and ITQ-CA questionnaires (Figure S5). The positive correlations were observed between CDI2-1 “Sadness” with all 12 ITQ-CA categories. The negative correlations were observed between CDI2-18 “Pain thoughts” with 9 ITQ-CA categories, CDI2-17 “Eating problems” with 3 ITQ-CA categories, CDI-9 “Tendency to cry” with ITQ-CA “Nervousness”, and CDI2-27 “Eating problems” with ITQ-CA “Sense of failure”. After the Benjamini-Hochberg correction, 19 correlations remained significant. The CDI2-1 loses significance with ITQ-CA “Reliving events in mind”. The remain significant correlations were between CDI2-17 “Eating problems” with ITQ-CA “Disconnection to others” and “Sense of failure”, and CDI-2-18 “Pain thoughts” with ITQ-CA “Nervousness”, “Sense of failure”, “Bad dreams”, “Relieving events in mind”, “Avoiding thoughts”, “Overly caution”, “Nervousness”, and “Self-doubt” (Table S5). Among the Ukrainian children who had reason to return to their home, but it was not the home itself, we observed 14 significant correlations (8 positive and six negative, all very weak or weak) between CDI2 and ITQ-C (Figure S5). The positive correlations were between CDI2-1 “Sadness” with ITQ-CA “Avoiding thoughts”, “Sense of failure”, “Self-doubt” and “Social difficulty”, CDI2-17 “Eating attitude” with ITQ-CA “Avoiding thoughts” and “Overlay cautions”, CDI2-26 “Napping//dozing” with ITQ-CA “Avoiding physically”, and CDI-27 “Eating problems” with ITQ-CA “Social difficulty”. The negative correlations were between CDI2-9 “Tendency to cry”, CDI2-10 “Bad mood” and CDI2-15 “Sleep quality” with ITQ-CA “Nervousness”, and CDI2-9 with ITQ-CA “Reliving events in mind”, CDI2-25 with ITQ-CA “Sense of failure”, and “CDI2-16 “Tiredness level” with ITQ-CA “Self-doubt” (Table S5). After Benjamini-Hochberg correction three correlation remain significant, CDI2-1 “Sadness” with ITQ-CA “Avoiding thoughts”, CDI2-10 “Bad mood” and CDI-15 “Sleep quality” with ITQ-CA “Nervousness” (Table S5). Among the Ukrainian children who returned to their homeland, the reason was their home; we observed six significant, negative, and one positive strong correlations between data obtained from CDI2 and ITQ-CA questionnaires (Figure S5). The positive correlation was between CDI2-16 “Tiredness level with ITQ-CA “Disconnection to others”. The negative correlations were between CDI2-9 “Tendency to cry”, CDI2-10 “Bad mood”, CDI2-15 “Sleep quality”, CDI2-17 “Eating attitude”, CDI2-18 “Pain thoughts”, and CDI2-26 “Napping/dozing” with ITQ-CA “Overly caution”. After Benjamini-Hochberg, none of the correlations remain significant (Table S5).

**CDI2 – group B**

In the CDI2 – group B among the children for whom the home country return reason was not applicable, we observed 19 significant, negative, very weak or weak in strength correlations between data obtained from CDI2 and ITQ-CA questionnaires (Figure S5). Most correlations were between CDI2-13 “Self-perception” and CDI2-24 “Love-awareness” with 8 and 7 ITQ-CA categories, respectively. Additional correlations were between CDI2-2 “Hopeful”, CDI-6 “Self-acceptance”, and CDI-7 “Blame” with ITQ-CA “Nervousness” and CDI2-2 with ITQ-CA “Sense of failure”. After the Benjamini-Hochberg correction, nine correlations remained significant (Table S5). The correlations were between CDI2-2 “Hopeful” with ITQ-CA “Nervousness” and “Sense of failure”, CDI2-13 “Self-perception” with ITQ-CA “Bad dreams”, “Nervousness”, “Calming difficulty”, “Sense of failure”, and Self-doubt” and “Bad dreams”, and CDI2-24 “Love-awareness” with ITQ-CA “Bad dreams” and “Sense of failure” (Table S5). Among the Ukrainian children whose return reason was not their home, we observed five significant, very weak, or weak correlations between CDI2 and ITQ-CA (Figure S5). Negative correlations were observed between CDI2-2 “Hopeful” with ITQ-CA “Relieving events in mind”, CDI2-7 “Blame” with ITQ-CA “Sense of failure” and “Disconnection to others”, CDI2-8 “Suicide” with ITQ-CA “Social difficulty”, and CDI2-24 “Love awareness” with ITQ-CA “Bad dreams”. After correction for multiple comparisons, none of the correlations remained significant (Table S5). We observed three significant correlations among the Ukrainian children whose return reason was their home (Figure S5). CDI2-6 “Self-acceptance” and CDI2-24 “Love awareness” were correlated with ITQ-CA “Overlay cautions”, and CDI2-13 “Self-perception” with ITQ-CA “Disconnection to others” (Table S5).

**CDI2 – group C**

In the CDI2 – group C among the children for whom the home country reason to return was not applicable, we observed 38 significant, negative, very weak or weak in strength correlations between data obtained from CDI2 and ITQ-CA questionnaires (Figure S5). Most of the correlations were between CDI2-20 “School fun”, and CDI2-28 “Memorization”, CDI2-4 “Enjoyment”, and CDI2-2 “Self-confidence” with 8, 8, 7, and 4 ITQ-CA categories, respectively. After the Benjamini-Hochberg correction, 14 correlations remained significant (Table S5). The correlations were between CDI2-3 “Self-confidence” with ITQ-CA “Nervousness”, “Sense of failure” and “Self-doubt”, CDI2-4 “Enjoyment” with ITQ-CA “Nervousness”, “Emotional numbness”, and “Sense of failure”, CDI2-20 “School fun” with ITQ-CA “Nervousness”, “Sense of failure” and “Self-doubt”, CDI2-28 “Memorization” with ITQ-CA “Bad dreams”, “Nervousness”, “Self-doubt”, and “Social difficulty” (Table S5). We observed 13 significant, positive, and two negative (very weak or weak) correlations between CDI2 and ITQ-CA (Figure S5) among the Ukrainian children who had reason to return, but it was not their home. Most were between CDI2-28 “Memorization” with ITQ-CA 6 categories and CDI2-12 “Self-determination” with 3 ITQ-CA categories. After Benjamini-Hochberg correction, we observed four significant correlations between CDI2 and ITQ-CA (Table S5). The correlations were between CDI2-14 “Learning attitudes” with ITQ-CA “Nervousness”, and CDI2-22 “Dealing with school tasks” with ITQ-CA “Avoiding thoughts” and “Avoiding physically”, and CDI2-28 “Memorization” with ITQ-CA “Calming difficulty” (Table S5).

We observed five significant, strong correlations among the Ukrainian children whose reason to return was their home. One correlation was negative (CDI2-12 “Self-determination” with ITQ-CA “Avoiding thoughts”). The remaining positive correlations were between CDI2-2 “Enjoyment” with ITQ-CA “Self-doubt and “Social difficulty” and CDI2-20 “School fun” with ITQ-CA “Social difficulty” (Figure S5). After correction for multiple comparisons, none of the correlations remain significant (Table S5).

**CDI2 – group D**

In the group of children for whom the reason to return to the home country was not applicable (Polish citizens or Ukrainian-born in Poland), we observed 18 significant, negative, very weak or weak correlations in CDI2–group D between data obtained from CDI2 and ITQ-CA questionnaires (Figure S5). Most correlations were between CDI2-5 “Importance for family” and 11 ITQ-CA categories. The remaining were between CDI2-11 “Company”, CDI2-19 “Loneliness feeling”, and “CDI2-25 “Peer arguing”. After Benjamini-Hochberg correction, 10 correlations remain significant (Table S5). The correlations were between CDI2-5 “Importance for family” with ITQ-CA “Bad dreams”, “Relieving events in mind”, “Overlay caution”, “Nervousness”, “Emotional numbness”, ”Sense of failure”, “Self-doubt”, “Disconnection to others”, and “Social difficulties”. The additional one correlation was observed between CDI2-11 “Company” with ITQ-CA “Sense of failure” (Table S5). Among the Ukrainian children who had reason to return to their home, it was not the home itself that we observed eight significant, four positive, and four negative, all very weak or weak between CDI2 and ITQ-C (Figure S5). Three positive correlations were between CDI2-11 “Company”, CDI2-21 “Friends”, and CDI2-25 “Peer arguing” with ITQ-CA “Avoiding thoughts”; the last positive correlation was between CDI2-25 and “Social difficulty”. Additionally, three negative correlations were observed between CDI2-21 “Friends” with ITQ-CA “Reliving events in mind, “Nervousness” and “Self-doubt”. The last negative correlation was observed between CDI2-25 with ITQ-CA “Nervousness” (Table S5). After Benjamini-Hochberg correction, only CDI2-25 “Peer arguing” with ITQ-CA “Avoiding thoughts” remain significant. Among the Ukrainian children who returned to their homeland, the reason was their home; we observed two significant correlations. Ten correlations were positive and strong (Figure S5). The CDI2-21 “Friends” category was correlated with ITQ-CA “Disconnection to others” and “Social difficulties”.


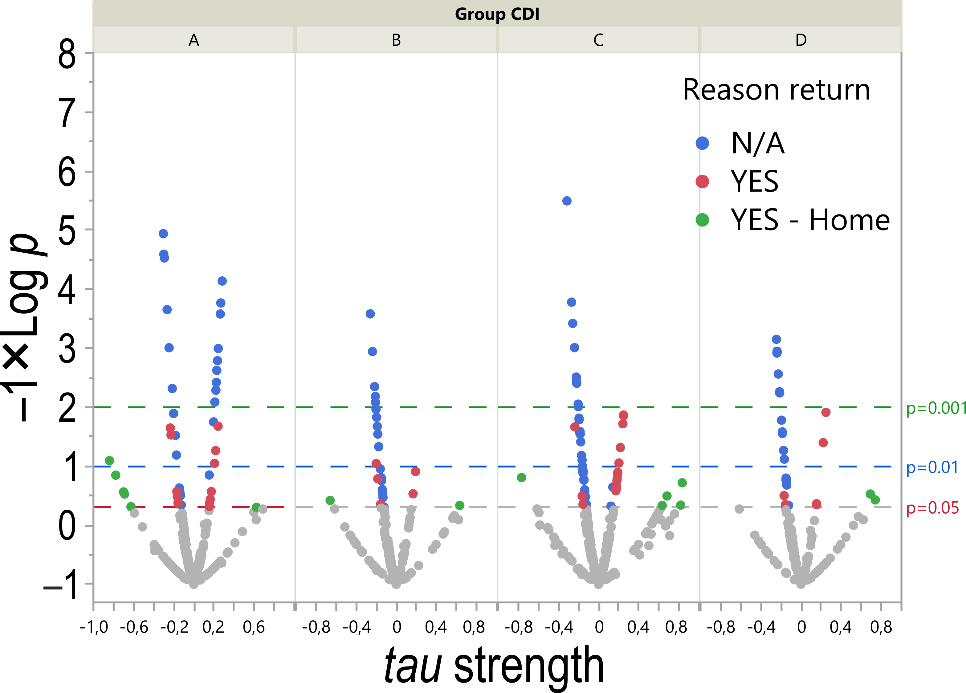


**Figure S5.** Correlations between single questions of CDI-2 (Children's Depression Inventory 2) and ITQ-CA (International Trauma Questionnaire – Child and Adolescent Version) in the reason for the country of origin return dependent groups of children and adolescents from Poland and Ukraine. Volcano plot of significant correlations between single questions of CDI and ITQ-CA in the CDI A, B, C, and D groups. The strength of *tau* correlation coefficient (x-axis) and the significance as the unadjusted *p*-values (shown as −1×log *p*; y-axis). The dashed horizontal lines represent the *p* = 0.05 (red), *p* = 0.01 (blue), and *p* = 0.001 (green). Significant correlations are shown as colored spots, and non-significant correlations are grey. March 2024-March 2025, Poland and Ukraine.

**Table S5.** Children's Depression Inventory 2 (CDI-2) (panels A-D) and International Trauma Questionnaire – Child and Adolescent Version (ITQ-CA) correlations in reason to return dependent groups of children and adolescents from Poland and Ukraine, March 2024-March 2025, Poland and Ukraine.

| **CDI2** | **ITQ-CA** | **Group CDI** | **N** | ***Tau*** | ***p*-value** | ***p*-value ^BH^** | **N** | ***Tau*** | ***p*-value** | ***p*-value ^BH^** | **N** | ***Tau*** | ***p*-value** | ***p*-value ^BH^** |
| --- | --- | --- | --- | --- | --- | --- | --- | --- | --- | --- | --- | --- | --- | --- |
|  |  | **Reason to return - Yes** | | | | | **Reason to return – YES - Home** | | | | **Reason to return – N/A** | | | |
| 1 Sadness | 1 Bad dreams | A | 76 | 0.06 | 0.4598 | 0.7177 | 7 | -0.59 | 0.0621 | 0.2592 | 119 | 0.23 | 0.0002 | 0.0089 |
|  | 2 Reliving events in mind | A | 76 | 0.00 | 0.9504 | 0.9833 | 7 | -0.40 | 0.2125 | 0.4863 | 118 | 0.15 | 0.0141 | 0.1164 |
|  | 3 Avoiding thoughts | A | 76 | 0.24 | 0.0021 | 0.0362 | 7 | -0.38 | 0.2265 | 0.5004 | 116 | 0.24 | 0.0002 | 0.0069 |
|  | 4 Avoiding physically | A | 76 | 0.09 | 0.2411 | 0.5184 | 7 | -0.22 | 0.4924 | 0.7423 | 115 | 0.22 | 0.0005 | 0.0145 |
|  | 5 Overly cautious | A | 75 | 0.01 | 0.9322 | 0.9748 | 7 | -0.08 | 0.8089 | 0.9228 | 116 | 0.22 | 0.0004 | 0.0117 |
|  | 6 Nervousness | A | 76 | 0.03 | 0.7155 | 0.8802 | 7 | -0.31 | 0.3333 | 0.6095 | 118 | 0.23 | 0.0002 | 0.0069 |
|  | 7 Calming difficulty | A | 75 | 0.15 | 0.0564 | 0.2458 | 7 | -0.22 | 0.4807 | 0.7357 | 117 | 0.26 | <0.0001 | 0.0022 |
|  | 8 Emotional numbness | A | 76 | 0.13 | 0.0965 | 0.3328 | 7 | -0.30 | 0.3471 | 0.6210 | 116 | 0.28 | <0.0001 | 0.0012 |
|  | 9 Sense of failure | A | 75 | 0.21 | 0.0089 | 0.0894 | 7 | -0.22 | 0.4924 | 0.7423 | 118 | 0.21 | 0.0008 | 0.0203 |
|  | 10 Self-doubt | A | 75 | 0.16 | 0.0367 | 0.1939 | 7 | -0.30 | 0.3471 | 0.6210 | 118 | 0.24 | 0.0001 | 0.0052 |
|  | 11 Disconnection to others | A | 76 | 0.06 | 0.4374 | 0.6985 | 7 | -0.16 | 0.6065 | 0.8181 | 117 | 0.20 | 0.0018 | 0.0322 |
|  | 12 Social difficulty | A | 76 | 0.22 | 0.0054 | 0.0649 | 6 | -0.39 | 0.2690 | 0.5470 | 118 | 0.27 | <0.0001 | 0.0019 |
| 2 Hopeful | 1 Bad dreams | B | 79 | 0.01 | 0.8848 | 0.9529 | 7 | -0.23 | 0.4654 | 0.7227 | 122 | -0.10 | 0.0981 | 0.3347 |
|  | 2 Reliving events in mind | B | 79 | -0.20 | 0.0090 | 0.0897 | 7 | 0.00 | 1.0000 | 1.0000 | 121 | -0.07 | 0.2447 | 0.5230 |
|  | 3 Avoiding thoughts | B | 79 | 0.01 | 0.8715 | 0.9465 | 7 | -0.42 | 0.1852 | 0.4535 | 118 | -0.03 | 0.6432 | 0.8389 |
|  | 4 Avoiding physically | B | 79 | 0.15 | 0.0542 | 0.2394 | 7 | 0.00 | 1.0000 | 1.0000 | 118 | -0.02 | 0.7251 | 0.8841 |
|  | 5 Overly cautious | B | 78 | -0.12 | 0.1272 | 0.3800 | 7 | -0.35 | 0.2695 | 0.5470 | 119 | -0.10 | 0.0942 | 0.3269 |
|  | 6 Nervousness | B | 79 | -0.10 | 0.1743 | 0.4408 | 7 | -0.35 | 0.2695 | 0.5470 | 121 | -0.20 | 0.0015 | 0.0288 |
|  | 7 Calming difficulty | B | 78 | 0.08 | 0.3013 | 0.5794 | 7 | 0.07 | 0.8301 | 0.9297 | 120 | -0.11 | 0.0671 | 0.2721 |
|  | 8 Emotional numbness | B | 78 | 0.02 | 0.8065 | 0.9222 | 7 | 0.14 | 0.6678 | 0.8526 | 119 | -0.06 | 0.3007 | 0.5789 |
|  | 9 Sense of failure | B | 78 | -0.03 | 0.7353 | 0.8887 | 7 | -0.07 | 0.8345 | 0.9318 | 121 | -0.19 | 0.0021 | 0.0362 |
|  | 10 Self-doubt | B | 78 | -0.08 | 0.3045 | 0.5817 | 7 | -0.14 | 0.6678 | 0.8526 | 121 | -0.08 | 0.1787 | 0.4461 |
|  | 11 Disconnection to others | B | 79 | -0.06 | 0.4050 | 0.6700 | 7 | 0.22 | 0.4807 | 0.7357 | 120 | -0.08 | 0.1814 | 0.4489 |
|  | 12 Social difficulty | B | 79 | 0.03 | 0.7070 | 0.8765 | 6 | 0.10 | 0.7823 | 0.9110 | 121 | -0.03 | 0.6543 | 0.8440 |
| 3 Self-confidence | 1 Bad dreams | C | 79 | 0.12 | 0.1090 | 0.3535 | 7 | 0.11 | 0.7308 | 0.8864 | 122 | -0.02 | 0.7906 | 0.9160 |
|  | 2 Reliving events in mind | C | 79 | -0.02 | 0.7525 | 0.8945 | 7 | 0.00 | 1.0000 | 1.0000 | 121 | -0.08 | 0.2151 | 0.4876 |
|  | 3 Avoiding thoughts | C | 79 | 0.17 | 0.0252 | 0.1621 | 7 | 0.00 | 1.0000 | 1.0000 | 118 | -0.04 | 0.5165 | 0.7574 |
|  | 4 Avoiding physically | C | 79 | 0.11 | 0.1464 | 0.4044 | 7 | 0.47 | 0.1397 | 0.3957 | 118 | -0.03 | 0.5893 | 0.8061 |
|  | 5 Overly cautious | C | 78 | 0.02 | 0.7688 | 0.9034 | 7 | -0.20 | 0.5323 | 0.7689 | 119 | -0.09 | 0.1527 | 0.4116 |
|  | 6 Nervousness | C | 79 | 0.02 | 0.8381 | 0.9329 | 7 | 0.40 | 0.2116 | 0.4861 | 121 | -0.22 | 0.0004 | 0.0120 |
|  | 7 Calming difficulty | C | 78 | 0.15 | 0.0603 | 0.2553 | 7 | 0.48 | 0.1292 | 0.3816 | 120 | -0.09 | 0.1405 | 0.3965 |
|  | 8 Emotional numbness | C | 78 | -0.02 | 0.7782 | 0.9089 | 7 | 0.48 | 0.1292 | 0.3816 | 119 | -0.05 | 0.4584 | 0.7167 |
|  | 9 Sense of failure | C | 78 | 0.11 | 0.1704 | 0.4367 | 7 | 0.56 | 0.0763 | 0.2928 | 121 | -0.18 | 0.0028 | 0.0428 |
|  | 10 Self-doubt | C | 78 | 0.01 | 0.8874 | 0.9537 | 7 | 0.58 | 0.0686 | 0.2751 | 121 | -0.19 | 0.0026 | 0.0410 |
|  | 11 Disconnection to others | C | 79 | 0.08 | 0.3204 | 0.5964 | 7 | 0.63 | 0.0461 | 0.2200 | 120 | -0.12 | 0.0435 | 0.2148 |
|  | 12 Social difficulty | C | 79 | 0.19 | 0.0158 | 0.1251 | 6 | 0.62 | 0.0805 | 0.2987 | 121 | -0.09 | 0.1547 | 0.4146 |
| 4 Enjoyment | 1 Bad dreams | C | 79 | 0.01 | 0.9103 | 0.9661 | 7 | 0.62 | 0.0516 | 0.2347 | 121 | -0.10 | 0.0903 | 0.3215 |
|  | 2 Reliving events in mind | C | 79 | -0.14 | 0.0684 | 0.2750 | 7 | 0.36 | 0.2551 | 0.5324 | 120 | -0.06 | 0.3549 | 0.6290 |
|  | 3 Avoiding thoughts | C | 79 | 0.07 | 0.3686 | 0.6400 | 7 | 0.35 | 0.2695 | 0.5470 | 117 | -0.01 | 0.9183 | 0.9700 |
|  | 4 Avoiding physically | C | 79 | 0.11 | 0.1518 | 0.4098 | 7 | 0.53 | 0.0947 | 0.3285 | 117 | 0.00 | 0.9467 | 0.9822 |
|  | 5 Overly cautious | C | 78 | -0.05 | 0.5338 | 0.7697 | 7 | -0.07 | 0.8252 | 0.9290 | 118 | -0.12 | 0.0578 | 0.2497 |
|  | 6 Nervousness | C | 79 | -0.09 | 0.2338 | 0.5106 | 7 | 0.56 | 0.0773 | 0.2952 | 120 | -0.32 | <0.0001 | <0.0001 |
|  | 7 Calming difficulty | C | 78 | -0.07 | 0.3915 | 0.6601 | 7 | 0.54 | 0.0860 | 0.3116 | 119 | -0.16 | 0.0079 | 0.0826 |
|  | 8 Emotional numbness | C | 78 | -0.16 | 0.0440 | 0.2156 | 7 | 0.61 | 0.0534 | 0.2377 | 118 | -0.22 | 0.0003 | 0.0111 |
|  | 9 Sense of failure | C | 78 | -0.07 | 0.3419 | 0.6169 | 7 | 0.60 | 0.0601 | 0.2550 | 120 | -0.19 | 0.0016 | 0.0305 |
|  | 10 Self-doubt | C | 78 | 0.02 | 0.7967 | 0.9189 | 7 | 0.68 | 0.0319 | 0.1800 | 120 | -0.22 | 0.0003 | 0.0105 |
|  | 11 Disconnection to others | C | 79 | 0.07 | 0.3659 | 0.6383 | 7 | 0.60 | 0.0600 | 0.2549 | 119 | -0.14 | 0.0251 | 0.1619 |
|  | 12 Social difficulty | C | 79 | 0.01 | 0.8516 | 0.9373 | 6 | 0.83 | 0.0190 | 0.1375 | 120 | -0.18 | 0.0039 | 0.0520 |
| 5 Importance for family | 1 Bad dreams | D | 79 | 0.11 | 0.1513 | 0.4095 | 7 | -0.34 | 0.2863 | 0.5611 | 121 | -0.24 | 0.0001 | 0.0042 |
|  | 2 Reliving events in mind | D | 79 | -0.11 | 0.1644 | 0.4289 | 7 | -0.40 | 0.2125 | 0.4863 | 120 | -0.19 | 0.0017 | 0.0307 |
|  | 3 Avoiding thoughts | D | 79 | 0.02 | 0.7923 | 0.9160 | 7 | -0.46 | 0.1467 | 0.4044 | 117 | -0.09 | 0.1485 | 0.4061 |
|  | 4 Avoiding physically | D | 79 | 0.07 | 0.3663 | 0.6386 | 7 | 0.00 | 1.0000 | 1.0000 | 117 | -0.15 | 0.0171 | 0.1305 |
|  | 5 Overly cautious | D | 78 | 0.00 | 0.9536 | 0.9857 | 7 | -0.61 | 0.0530 | 0.2374 | 118 | -0.19 | 0.0028 | 0.0428 |
|  | 6 Nervousness | D | 79 | 0.03 | 0.6916 | 0.8663 | 7 | -0.15 | 0.6285 | 0.8325 | 120 | -0.21 | 0.0005 | 0.0151 |
|  | 7 Calming difficulty | D | 78 | 0.07 | 0.3342 | 0.6104 | 7 | 0.00 | 1.0000 | 1.0000 | 119 | -0.17 | 0.0076 | 0.0808 |
|  | 8 Emotional numbness | D | 78 | -0.11 | 0.1461 | 0.4044 | 7 | 0.00 | 1.0000 | 1.0000 | 118 | -0.24 | 0.0001 | 0.0057 |
|  | 9 Sense of failure | D | 78 | -0.01 | 0.8786 | 0.9495 | 7 | 0.07 | 0.8190 | 0.9285 | 120 | -0.21 | 0.0006 | 0.0155 |
|  | 10 Self-doubt | D | 78 | 0.06 | 0.4013 | 0.6682 | 7 | 0.15 | 0.6382 | 0.8355 | 120 | -0.24 | 0.0001 | 0.0055 |
|  | 11 Disconnection to others | D | 79 | 0.10 | 0.1752 | 0.4408 | 7 | 0.57 | 0.0714 | 0.2810 | 119 | -0.23 | 0.0003 | 0.0098 |
|  | 12 Social difficulty | D | 79 | 0.13 | 0.0832 | 0.3052 | 6 | 0.62 | 0.0805 | 0.2987 | 120 | -0.19 | 0.0026 | 0.0413 |
| 6 Self-acceptance | 1 Bad dreams | B | 79 | 0.12 | 0.1107 | 0.3561 | 7 | -0.40 | 0.2038 | 0.4778 | 121 | -0.04 | 0.5454 | 0.7767 |
|  | 2 Reliving events in mind | B | 79 | -0.11 | 0.1654 | 0.4301 | 7 | -0.45 | 0.1537 | 0.4133 | 120 | 0.02 | 0.7616 | 0.8994 |
|  | 3 Avoiding thoughts | B | 79 | 0.06 | 0.4252 | 0.6902 | 7 | -0.51 | 0.1064 | 0.3501 | 117 | 0.06 | 0.3276 | 0.6038 |
|  | 4 Avoiding physically | B | 79 | 0.04 | 0.5602 | 0.7857 | 7 | -0.07 | 0.8273 | 0.9295 | 117 | 0.00 | 0.9612 | 0.9896 |
|  | 5 Overly cautious | B | 78 | 0.04 | 0.5625 | 0.7863 | 7 | -0.66 | 0.0379 | 0.1972 | 118 | -0.03 | 0.6823 | 0.8604 |
|  | 6 Nervousness | B | 79 | -0.08 | 0.3196 | 0.5955 | 7 | -0.22 | 0.4890 | 0.7396 | 120 | -0.14 | 0.0263 | 0.1643 |
|  | 7 Calming difficulty | B | 78 | -0.07 | 0.3722 | 0.6442 | 7 | -0.07 | 0.8226 | 0.9290 | 119 | -0.05 | 0.4380 | 0.6992 |
|  | 8 Emotional numbness | B | 78 | 0.00 | 0.9598 | 0.9888 | 7 | -0.07 | 0.8226 | 0.9290 | 118 | -0.01 | 0.8906 | 0.9560 |
|  | 9 Sense of failure | B | 78 | -0.05 | 0.5109 | 0.7530 | 7 | 0.00 | 1.0000 | 1.0000 | 120 | -0.11 | 0.0737 | 0.2881 |
|  | 10 Self-doubt | B | 78 | -0.02 | 0.7613 | 0.8994 | 7 | 0.07 | 0.8226 | 0.9290 | 120 | 0.02 | 0.7110 | 0.8782 |
|  | 11 Disconnection to others | B | 79 | 0.01 | 0.8459 | 0.9343 | 7 | 0.47 | 0.1407 | 0.3965 | 119 | -0.07 | 0.2545 | 0.5324 |
|  | 12 Social difficulty | B | 79 | 0.10 | 0.2048 | 0.4789 | 6 | 0.62 | 0.0805 | 0.2987 | 120 | -0.02 | 0.7353 | 0.8887 |
| 7 Blame | 1 Bad dreams | B | 79 | 0.04 | 0.6303 | 0.8332 | 7 | 0.15 | 0.6265 | 0.8319 | 120 | -0.06 | 0.2927 | 0.5679 |
|  | 2 Reliving events in mind | B | 79 | -0.09 | 0.2364 | 0.5132 | 7 | 0.36 | 0.2551 | 0.5324 | 119 | -0.07 | 0.2279 | 0.5027 |
|  | 3 Avoiding thoughts | B | 79 | 0.02 | 0.7572 | 0.8969 | 7 | -0.07 | 0.8252 | 0.9290 | 116 | -0.04 | 0.5695 | 0.7908 |
|  | 4 Avoiding physically | B | 79 | 0.07 | 0.3578 | 0.6319 | 7 | 0.00 | 1.0000 | 1.0000 | 116 | -0.03 | 0.6484 | 0.8407 |
|  | 5 Overly cautious | B | 78 | 0.07 | 0.3379 | 0.6136 | 7 | -0.35 | 0.2695 | 0.5470 | 117 | -0.05 | 0.3989 | 0.6665 |
|  | 6 Nervousness | B | 79 | -0.11 | 0.1395 | 0.3957 | 7 | -0.35 | 0.2695 | 0.5470 | 119 | -0.12 | 0.0489 | 0.2270 |
|  | 7 Calming difficulty | B | 78 | -0.05 | 0.4909 | 0.7412 | 7 | 0.07 | 0.8301 | 0.9297 | 118 | -0.06 | 0.3396 | 0.6154 |
|  | 8 Emotional numbness | B | 78 | -0.12 | 0.1242 | 0.3748 | 7 | 0.14 | 0.6678 | 0.8526 | 117 | 0.02 | 0.7089 | 0.8769 |
|  | 9 Sense of failure | B | 78 | -0.16 | 0.0437 | 0.2153 | 7 | -0.26 | 0.4034 | 0.6689 | 119 | -0.06 | 0.3685 | 0.6400 |
|  | 10 Self-doubt | B | 78 | -0.11 | 0.1699 | 0.4367 | 7 | -0.27 | 0.3907 | 0.6589 | 119 | -0.03 | 0.6360 | 0.8355 |
|  | 11 Disconnection to others | B | 79 | -0.18 | 0.0163 | 0.1275 | 7 | -0.15 | 0.6382 | 0.8355 | 118 | -0.03 | 0.6696 | 0.8533 |
|  | 12 Social difficulty | B | 79 | -0.13 | 0.0881 | 0.3163 | 6 | -0.20 | 0.5805 | 0.7994 | 119 | -0.02 | 0.7692 | 0.9034 |
| 8 Suicide | 1 Bad dreams | B | 79 | 0.10 | 0.2131 | 0.4868 | 7 | -0.34 | 0.2863 | 0.5611 | 119 | 0.00 | 0.9373 | 0.9762 |
|  | 2 Reliving events in mind | B | 79 | -0.15 | 0.0513 | 0.2334 | 7 | -0.40 | 0.2125 | 0.4863 | 118 | -0.02 | 0.7209 | 0.8824 |
|  | 3 Avoiding thoughts | B | 79 | 0.13 | 0.1013 | 0.3406 | 7 | -0.46 | 0.1467 | 0.4044 | 115 | 0.04 | 0.5594 | 0.7852 |
|  | 4 Avoiding physically | B | 79 | 0.01 | 0.8607 | 0.9404 | 7 | 0.00 | 1.0000 | 1.0000 | 115 | 0.01 | 0.8347 | 0.9318 |
|  | 5 Overly cautious | B | 78 | -0.01 | 0.8478 | 0.9350 | 7 | -0.61 | 0.0530 | 0.2374 | 116 | 0.00 | 0.9823 | 1.0000 |
|  | 6 Nervousness | B | 79 | -0.06 | 0.4625 | 0.7204 | 7 | -0.15 | 0.6285 | 0.8325 | 118 | -0.07 | 0.2600 | 0.5368 |
|  | 7 Calming difficulty | B | 78 | 0.01 | 0.9318 | 0.9748 | 7 | 0.00 | 1.0000 | 1.0000 | 117 | -0.03 | 0.6278 | 0.8325 |
|  | 8 Emotional numbness | B | 78 | -0.04 | 0.6046 | 0.8171 | 7 | 0.00 | 1.0000 | 1.0000 | 116 | -0.01 | 0.8288 | 0.9297 |
|  | 9 Sense of failure | B | 78 | -0.04 | 0.6318 | 0.8338 | 7 | 0.07 | 0.8190 | 0.9285 | 118 | -0.12 | 0.0559 | 0.2446 |
|  | 10 Self-doubt | B | 78 | -0.05 | 0.5122 | 0.7538 | 7 | 0.15 | 0.6382 | 0.8355 | 118 | -0.06 | 0.3621 | 0.6348 |
|  | 11 Disconnection to others | B | 79 | -0.04 | 0.5632 | 0.7869 | 7 | 0.57 | 0.0714 | 0.2810 | 117 | 0.04 | 0.5683 | 0.7898 |
|  | 12 Social difficulty | B | 79 | 0.17 | 0.0293 | 0.1748 | 6 | 0.62 | 0.0805 | 0.2987 | 118 | 0.08 | 0.2258 | 0.4997 |
| 9 Tendency to cry | 1 Bad dreams | A | 79 | 0.08 | 0.3039 | 0.5806 | 7 | 0.00 | 1.0000 | 1.0000 | 120 | -0.02 | 0.8050 | 0.9214 |
|  | 2 Reliving events in mind | A | 79 | -0.17 | 0.0268 | 0.1655 | 7 | -0.13 | 0.6734 | 0.8553 | 119 | -0.06 | 0.3024 | 0.5801 |
|  | 3 Avoiding thoughts | A | 79 | 0.02 | 0.7653 | 0.9014 | 7 | -0.19 | 0.5397 | 0.7729 | 116 | 0.02 | 0.7294 | 0.8858 |
|  | 4 Avoiding physically | A | 79 | 0.00 | 0.9548 | 0.9861 | 7 | 0.18 | 0.5618 | 0.7861 | 116 | 0.04 | 0.5597 | 0.7853 |
|  | 5 Overly cautious | A | 78 | 0.02 | 0.7892 | 0.9157 | 7 | -0.78 | 0.0142 | 0.1169 | 117 | 0.01 | 0.9204 | 0.9709 |
|  | 6 Nervousness | A | 79 | -0.16 | 0.0431 | 0.2142 | 7 | 0.00 | 1.0000 | 1.0000 | 119 | -0.13 | 0.0314 | 0.1782 |
|  | 7 Calming difficulty | A | 78 | 0.02 | 0.8451 | 0.9342 | 7 | 0.19 | 0.5511 | 0.7796 | 118 | -0.06 | 0.3177 | 0.5932 |
|  | 8 Emotional numbness | A | 78 | 0.01 | 0.9172 | 0.9693 | 7 | 0.19 | 0.5511 | 0.7796 | 117 | 0.03 | 0.6114 | 0.8219 |
|  | 9 Sense of failure | A | 78 | -0.10 | 0.2140 | 0.4875 | 7 | 0.12 | 0.6989 | 0.8711 | 119 | -0.04 | 0.5509 | 0.7796 |
|  | 10 Self-doubt | A | 78 | 0.05 | 0.4842 | 0.7376 | 7 | 0.25 | 0.4268 | 0.6907 | 119 | -0.10 | 0.0974 | 0.3336 |
|  | 11 Disconnection to others | A | 79 | -0.05 | 0.4882 | 0.7393 | 7 | 0.48 | 0.1276 | 0.3800 | 118 | 0.03 | 0.5816 | 0.8003 |
|  | 12 Social difficulty | A | 79 | 0.00 | 1.0000 | 1.0000 | 6 | 0.69 | 0.0531 | 0.2374 | 119 | 0.10 | 0.0968 | 0.3328 |
| 10 Bad mood | 1 Bad dreams | A | 79 | 0.11 | 0.1457 | 0.4043 | 7 | -0.23 | 0.4654 | 0.7227 | 122 | 0.03 | 0.6753 | 0.8565 |
|  | 2 Reliving events in mind | A | 79 | -0.11 | 0.1397 | 0.3957 | 7 | -0.36 | 0.2551 | 0.5324 | 121 | -0.04 | 0.4882 | 0.7393 |
|  | 3 Avoiding thoughts | A | 79 | 0.07 | 0.3550 | 0.6290 | 7 | -0.35 | 0.2695 | 0.5470 | 118 | 0.07 | 0.2937 | 0.5689 |
|  | 4 Avoiding physically | A | 79 | 0.08 | 0.3079 | 0.5841 | 7 | 0.00 | 1.0000 | 1.0000 | 118 | 0.02 | 0.7063 | 0.8762 |
|  | 5 Overly cautious | A | 78 | 0.07 | 0.3374 | 0.6134 | 7 | -0.84 | 0.0081 | 0.0839 | 119 | 0.03 | 0.6466 | 0.8395 |
|  | 6 Nervousness | A | 79 | -0.23 | 0.0029 | 0.0437 | 7 | -0.07 | 0.8252 | 0.9290 | 121 | -0.10 | 0.0929 | 0.3249 |
|  | 7 Calming difficulty | A | 78 | 0.10 | 0.2077 | 0.4812 | 7 | 0.00 | 1.0000 | 1.0000 | 120 | -0.06 | 0.3686 | 0.6400 |
|  | 8 Emotional numbness | A | 78 | 0.01 | 0.8834 | 0.9521 | 7 | 0.00 | 1.0000 | 1.0000 | 119 | 0.03 | 0.6783 | 0.8586 |
|  | 9 Sense of failure | A | 78 | -0.04 | 0.6128 | 0.8235 | 7 | -0.07 | 0.8345 | 0.9318 | 121 | -0.10 | 0.1127 | 0.3589 |
|  | 10 Self-doubt | A | 78 | 0.06 | 0.4615 | 0.7195 | 7 | 0.14 | 0.6678 | 0.8526 | 121 | -0.08 | 0.2025 | 0.4754 |
|  | 11 Disconnection to others | A | 79 | -0.05 | 0.5141 | 0.7555 | 7 | 0.15 | 0.6382 | 0.8355 | 120 | 0.06 | 0.3541 | 0.6284 |
|  | 12 Social difficulty | A | 79 | 0.07 | 0.3313 | 0.6078 | 6 | 0.28 | 0.4345 | 0.6972 | 121 | 0.06 | 0.3131 | 0.5903 |
| 11 Company | 1 Bad dreams | D | 79 | 0.06 | 0.4427 | 0.7023 | 7 | 0.17 | 0.5940 | 0.8084 | 121 | -0.02 | 0.7078 | 0.8767 |
|  | 2 Reliving events in mind | D | 79 | -0.01 | 0.8861 | 0.9534 | 7 | 0.40 | 0.2125 | 0.4863 | 120 | -0.06 | 0.3507 | 0.6253 |
|  | 3 Avoiding thoughts | D | 79 | 0.22 | 0.0040 | 0.0528 | 7 | 0.00 | 1.0000 | 1.0000 | 117 | -0.06 | 0.3315 | 0.6078 |
|  | 4 Avoiding physically | D | 79 | 0.07 | 0.3567 | 0.6309 | 7 | 0.36 | 0.2526 | 0.5318 | 117 | 0.02 | 0.7925 | 0.9160 |
|  | 5 Overly cautious | D | 78 | -0.07 | 0.3661 | 0.6385 | 7 | 0.08 | 0.8089 | 0.9228 | 118 | -0.03 | 0.5914 | 0.8075 |
|  | 6 Nervousness | D | 79 | -0.03 | 0.7415 | 0.8903 | 7 | 0.08 | 0.8089 | 0.9228 | 120 | -0.06 | 0.2962 | 0.5719 |
|  | 7 Calming difficulty | D | 78 | -0.02 | 0.7751 | 0.9066 | 7 | 0.45 | 0.1584 | 0.4207 | 119 | -0.05 | 0.4394 | 0.6995 |
|  | 8 Emotional numbness | D | 78 | -0.06 | 0.4665 | 0.7237 | 7 | 0.52 | 0.0999 | 0.3380 | 118 | -0.02 | 0.7113 | 0.8782 |
|  | 9 Sense of failure | D | 78 | -0.09 | 0.2588 | 0.5368 | 7 | 0.29 | 0.3601 | 0.6333 | 120 | -0.24 | 0.0001 | 0.0055 |
|  | 10 Self-doubt | D | 78 | -0.12 | 0.1080 | 0.3521 | 7 | 0.15 | 0.6382 | 0.8355 | 120 | -0.06 | 0.3584 | 0.6325 |
|  | 11 Disconnection to others | D | 79 | -0.02 | 0.7532 | 0.8947 | 7 | 0.16 | 0.6065 | 0.8181 | 119 | 0.01 | 0.8993 | 0.9604 |
|  | 12 Social difficulty | D | 79 | -0.03 | 0.7203 | 0.8824 | 6 | 0.10 | 0.7823 | 0.9110 | 120 | 0.01 | 0.8871 | 0.9537 |
| 12 Self-determination | 1 Bad dreams | C | 78 | 0.12 | 0.1057 | 0.3487 | 7 | -0.59 | 0.0621 | 0.2592 | 122 | -0.11 | 0.0712 | 0.2807 |
|  | 2 Reliving events in mind | C | 78 | 0.06 | 0.4315 | 0.6944 | 7 | -0.40 | 0.2125 | 0.4863 | 121 | -0.08 | 0.1904 | 0.4598 |
|  | 3 Avoiding thoughts | C | 78 | 0.06 | 0.4631 | 0.7210 | 7 | -0.77 | 0.0156 | 0.1240 | 118 | -0.04 | 0.5407 | 0.7736 |
|  | 4 Avoiding physically | C | 78 | 0.14 | 0.0747 | 0.2894 | 7 | -0.51 | 0.1092 | 0.3538 | 118 | -0.03 | 0.6201 | 0.8276 |
|  | 5 Overly cautious | C | 77 | 0.08 | 0.3179 | 0.5932 | 7 | -0.31 | 0.3333 | 0.6095 | 119 | -0.11 | 0.0643 | 0.2648 |
|  | 6 Nervousness | C | 78 | 0.04 | 0.5927 | 0.8079 | 7 | -0.61 | 0.0530 | 0.2374 | 121 | -0.15 | 0.0172 | 0.1306 |
|  | 7 Calming difficulty | C | 77 | 0.10 | 0.2093 | 0.4842 | 7 | -0.45 | 0.1584 | 0.4207 | 120 | -0.10 | 0.0915 | 0.3236 |
|  | 8 Emotional numbness | C | 77 | 0.13 | 0.1050 | 0.3476 | 7 | -0.30 | 0.3471 | 0.6210 | 119 | -0.08 | 0.1802 | 0.4472 |
|  | 9 Sense of failure | C | 77 | 0.18 | 0.0229 | 0.1550 | 7 | -0.44 | 0.1698 | 0.4367 | 121 | -0.13 | 0.0329 | 0.1827 |
|  | 10 Self-doubt | C | 77 | -0.07 | 0.3865 | 0.6557 | 7 | -0.45 | 0.1584 | 0.4207 | 121 | -0.05 | 0.4213 | 0.6855 |
|  | 11 Disconnection to others | C | 78 | 0.20 | 0.0088 | 0.0890 | 7 | -0.16 | 0.6065 | 0.8181 | 120 | -0.06 | 0.3440 | 0.6188 |
|  | 12 Social difficulty | C | 78 | 0.17 | 0.0260 | 0.1643 | 6 | -0.10 | 0.7823 | 0.9110 | 121 | -0.09 | 0.1364 | 0.3921 |
| 13 Self-perception | 1 Bad dreams | B | 78 | 0.06 | 0.4275 | 0.6911 | 7 | 0.11 | 0.7308 | 0.8864 | 122 | -0.21 | 0.0007 | 0.0173 |
|  | 2 Reliving events in mind | B | 78 | -0.06 | 0.4580 | 0.7166 | 7 | 0.00 | 1.0000 | 1.0000 | 121 | -0.12 | 0.0581 | 0.2499 |
|  | 3 Avoiding thoughts | B | 78 | 0.09 | 0.2205 | 0.4938 | 7 | 0.00 | 1.0000 | 1.0000 | 118 | -0.12 | 0.0539 | 0.2392 |
|  | 4 Avoiding physically | B | 78 | 0.10 | 0.2135 | 0.4868 | 7 | 0.47 | 0.1397 | 0.3957 | 118 | -0.13 | 0.0338 | 0.1853 |
|  | 5 Overly cautious | B | 77 | -0.01 | 0.9230 | 0.9719 | 7 | -0.20 | 0.5323 | 0.7689 | 119 | -0.14 | 0.0248 | 0.1604 |
|  | 6 Nervousness | B | 78 | -0.11 | 0.1613 | 0.4248 | 7 | 0.40 | 0.2116 | 0.4861 | 121 | -0.24 | 0.0001 | 0.0055 |
|  | 7 Calming difficulty | B | 77 | 0.02 | 0.7480 | 0.8934 | 7 | 0.48 | 0.1292 | 0.3816 | 120 | -0.21 | 0.0008 | 0.0203 |
|  | 8 Emotional numbness | B | 77 | -0.10 | 0.2144 | 0.4875 | 7 | 0.48 | 0.1292 | 0.3816 | 119 | -0.15 | 0.0155 | 0.1240 |
|  | 9 Sense of failure | B | 77 | -0.03 | 0.6536 | 0.8438 | 7 | 0.56 | 0.0763 | 0.2928 | 121 | -0.26 | <0.0001 | 0.0022 |
|  | 10 Self-doubt | B | 77 | -0.02 | 0.8123 | 0.9255 | 7 | 0.58 | 0.0686 | 0.2751 | 121 | -0.18 | 0.0029 | 0.0428 |
|  | 11 Disconnection to others | B | 78 | 0.03 | 0.7185 | 0.8823 | 7 | 0.63 | 0.0461 | 0.2200 | 120 | -0.11 | 0.0653 | 0.2678 |
|  | 12 Social difficulty | B | 78 | 0.03 | 0.7310 | 0.8864 | 6 | 0.62 | 0.0805 | 0.2987 | 121 | -0.07 | 0.2830 | 0.5585 |
| 14 Learning attitude | 1 Bad dreams | C | 78 | 0.02 | 0.7491 | 0.8934 | 7 | 0.15 | 0.6265 | 0.8319 | 122 | 0.08 | 0.2104 | 0.4849 |
|  | 2 Reliving events in mind | C | 78 | -0.09 | 0.2553 | 0.5325 | 7 | 0.36 | 0.2551 | 0.5324 | 121 | 0.07 | 0.2785 | 0.5538 |
|  | 3 Avoiding thoughts | C | 78 | 0.01 | 0.9250 | 0.9720 | 7 | -0.07 | 0.8252 | 0.9290 | 118 | 0.09 | 0.1457 | 0.4043 |
|  | 4 Avoiding physically | C | 78 | -0.08 | 0.2899 | 0.5645 | 7 | 0.00 | 1.0000 | 1.0000 | 118 | 0.11 | 0.0776 | 0.2953 |
|  | 5 Overly cautious | C | 77 | -0.07 | 0.3481 | 0.6224 | 7 | -0.35 | 0.2695 | 0.5470 | 119 | 0.12 | 0.0474 | 0.2241 |
|  | 6 Nervousness | C | 78 | -0.24 | 0.0022 | 0.0367 | 7 | -0.35 | 0.2695 | 0.5470 | 121 | -0.01 | 0.8858 | 0.9533 |
|  | 7 Calming difficulty | C | 77 | 0.01 | 0.9285 | 0.9738 | 7 | 0.07 | 0.8301 | 0.9297 | 120 | 0.01 | 0.8108 | 0.9242 |
|  | 8 Emotional numbness | C | 77 | -0.09 | 0.2361 | 0.5132 | 7 | 0.14 | 0.6678 | 0.8526 | 119 | -0.01 | 0.9354 | 0.9757 |
|  | 9 Sense of failure | C | 77 | -0.07 | 0.3825 | 0.6534 | 7 | -0.26 | 0.4034 | 0.6689 | 121 | 0.09 | 0.1464 | 0.4044 |
|  | 10 Self-doubt | C | 77 | -0.09 | 0.2220 | 0.4957 | 7 | -0.27 | 0.3907 | 0.6589 | 121 | -0.06 | 0.3373 | 0.6134 |
|  | 11 Disconnection to others | C | 78 | -0.13 | 0.0922 | 0.3244 | 7 | -0.15 | 0.6382 | 0.8355 | 120 | 0.14 | 0.0225 | 0.1534 |
|  | 12 Social difficulty | C | 78 | -0.04 | 0.5888 | 0.8061 | 6 | -0.20 | 0.5805 | 0.7994 | 121 | 0.05 | 0.3925 | 0.6605 |
| 15 Sleep quality | 1 Bad dreams | A | 79 | 0.00 | 0.9904 | 1.0000 | 7 | 0.15 | 0.6265 | 0.8319 | 121 | 0.05 | 0.4204 | 0.6851 |
|  | 2 Reliving events in mind | A | 79 | 0.02 | 0.7683 | 0.9034 | 7 | 0.00 | 1.0000 | 1.0000 | 120 | 0.04 | 0.5093 | 0.7515 |
|  | 3 Avoiding thoughts | A | 79 | -0.05 | 0.5100 | 0.7521 | 7 | -0.07 | 0.8252 | 0.9290 | 117 | 0.04 | 0.4825 | 0.7368 |
|  | 4 Avoiding physically | A | 79 | -0.10 | 0.1953 | 0.4653 | 7 | 0.33 | 0.2963 | 0.5719 | 117 | 0.00 | 0.9969 | 1.0000 |
|  | 5 Overly cautious | A | 78 | 0.06 | 0.4156 | 0.6799 | 7 | -0.70 | 0.0272 | 0.1669 | 118 | 0.01 | 0.8726 | 0.9471 |
|  | 6 Nervousness | A | 79 | -0.23 | 0.0022 | 0.0377 | 7 | 0.14 | 0.6587 | 0.8474 | 120 | -0.07 | 0.2876 | 0.5623 |
|  | 7 Calming difficulty | A | 78 | -0.09 | 0.2601 | 0.5368 | 7 | 0.34 | 0.2833 | 0.5585 | 119 | -0.07 | 0.2923 | 0.5678 |
|  | 8 Emotional numbness | A | 78 | -0.06 | 0.4307 | 0.6935 | 7 | 0.34 | 0.2833 | 0.5585 | 118 | 0.03 | 0.6814 | 0.8601 |
|  | 9 Sense of failure | A | 78 | -0.16 | 0.0329 | 0.1827 | 7 | 0.26 | 0.4034 | 0.6689 | 120 | -0.05 | 0.3770 | 0.6485 |
|  | 10 Self-doubt | A | 78 | -0.01 | 0.9174 | 0.9693 | 7 | 0.41 | 0.1979 | 0.4691 | 120 | -0.08 | 0.1854 | 0.4535 |
|  | 11 Disconnection to others | A | 79 | -0.14 | 0.0683 | 0.2748 | 7 | 0.60 | 0.0600 | 0.2549 | 119 | 0.02 | 0.7384 | 0.8895 |
|  | 12 Social difficulty | A | 79 | -0.06 | 0.4028 | 0.6689 | 6 | 0.69 | 0.0531 | 0.2374 | 120 | 0.04 | 0.5516 | 0.7797 |
| 16 Tiredness level | 1 Bad dreams | A | 78 | 0.05 | 0.5083 | 0.7505 | 7 | -0.24 | 0.4458 | 0.7058 | 120 | 0.00 | 0.9696 | 0.9929 |
|  | 2 Reliving events in mind | A | 78 | -0.11 | 0.1652 | 0.4300 | 7 | -0.30 | 0.3416 | 0.6166 | 119 | 0.00 | 0.9502 | 0.9833 |
|  | 3 Avoiding thoughts | A | 78 | 0.12 | 0.1219 | 0.3723 | 7 | -0.37 | 0.2488 | 0.5271 | 116 | 0.01 | 0.8626 | 0.9413 |
|  | 4 Avoiding physically | A | 78 | 0.02 | 0.7998 | 0.9192 | 7 | 0.07 | 0.8273 | 0.9295 | 116 | -0.01 | 0.8288 | 0.9297 |
|  | 5 Overly cautious | A | 77 | -0.03 | 0.7098 | 0.8776 | 7 | -0.51 | 0.1064 | 0.3501 | 117 | -0.04 | 0.4864 | 0.7382 |
|  | 6 Nervousness | A | 78 | -0.06 | 0.4050 | 0.6700 | 7 | -0.07 | 0.8176 | 0.9281 | 119 | -0.11 | 0.0778 | 0.2955 |
|  | 7 Calming difficulty | A | 77 | -0.02 | 0.8146 | 0.9264 | 7 | 0.07 | 0.8226 | 0.9290 | 118 | -0.08 | 0.1832 | 0.4512 |
|  | 8 Emotional numbness | A | 77 | -0.12 | 0.1380 | 0.3942 | 7 | 0.07 | 0.8226 | 0.9290 | 117 | 0.03 | 0.5917 | 0.8076 |
|  | 9 Sense of failure | A | 77 | -0.08 | 0.2790 | 0.5544 | 7 | 0.14 | 0.6626 | 0.8508 | 119 | -0.04 | 0.4740 | 0.7306 |
|  | 10 Self-doubt | A | 77 | -0.16 | 0.0417 | 0.2104 | 7 | 0.21 | 0.5013 | 0.7458 | 119 | -0.08 | 0.2115 | 0.4861 |
|  | 11 Disconnection to others | A | 78 | -0.10 | 0.2138 | 0.4873 | 7 | 0.62 | 0.0495 | 0.2288 | 118 | 0.00 | 0.9451 | 0.9813 |
|  | 12 Social difficulty | A | 78 | 0.08 | 0.3048 | 0.5820 | 6 | 0.62 | 0.0805 | 0.2987 | 119 | -0.05 | 0.3889 | 0.6578 |
| 17 Eating attitude | 1 Bad dreams | A | 77 | 0.10 | 0.2098 | 0.4847 | 7 | 0.15 | 0.6265 | 0.8319 | 121 | -0.05 | 0.3981 | 0.6658 |
|  | 2 Reliving events in mind | A | 77 | -0.04 | 0.5894 | 0.8061 | 7 | 0.00 | 1.0000 | 1.0000 | 120 | -0.08 | 0.1807 | 0.4481 |
|  | 3 Avoiding thoughts | A | 77 | 0.16 | 0.0363 | 0.1931 | 7 | -0.07 | 0.8252 | 0.9290 | 117 | -0.10 | 0.1058 | 0.3489 |
|  | 4 Avoiding physically | A | 77 | 0.13 | 0.0919 | 0.3238 | 7 | 0.33 | 0.2963 | 0.5719 | 117 | -0.04 | 0.5653 | 0.7884 |
|  | 5 Overly cautious | A | 76 | 0.17 | 0.0268 | 0.1655 | 7 | -0.70 | 0.0272 | 0.1669 | 118 | -0.08 | 0.2125 | 0.4863 |
|  | 6 Nervousness | A | 77 | 0.00 | 0.9642 | 0.9911 | 7 | 0.14 | 0.6587 | 0.8474 | 120 | -0.22 | 0.0005 | 0.0142 |
|  | 7 Calming difficulty | A | 76 | 0.04 | 0.6543 | 0.8440 | 7 | 0.34 | 0.2833 | 0.5585 | 119 | -0.12 | 0.0453 | 0.2192 |
|  | 8 Emotional numbness | A | 76 | -0.05 | 0.4956 | 0.7436 | 7 | 0.34 | 0.2833 | 0.5585 | 118 | -0.08 | 0.1825 | 0.4502 |
|  | 9 Sense of failure | A | 76 | -0.07 | 0.3983 | 0.6660 | 7 | 0.26 | 0.4034 | 0.6689 | 120 | -0.18 | 0.0030 | 0.0440 |
|  | 10 Self-doubt | A | 76 | 0.00 | 0.9629 | 0.9902 | 7 | 0.41 | 0.1979 | 0.4691 | 120 | -0.08 | 0.2104 | 0.4849 |
|  | 11 Disconnection to others | A | 77 | 0.04 | 0.6466 | 0.8395 | 7 | 0.60 | 0.0600 | 0.2549 | 119 | -0.08 | 0.2182 | 0.4909 |
|  | 12 Social difficulty | A | 77 | 0.09 | 0.2265 | 0.5004 | 6 | 0.69 | 0.0531 | 0.2374 | 120 | -0.03 | 0.6631 | 0.8510 |
| 18 Pain thoughts | 1 Bad dreams | A | 78 | -0.02 | 0.7922 | 0.9160 | 7 | 0.00 | 1.0000 | 1.0000 | 117 | -0.30 | <0.0001 | 0.0005 |
|  | 2 Reliving events in mind | A | 78 | 0.08 | 0.3300 | 0.6072 | 7 | 0.13 | 0.6839 | 0.8607 | 116 | -0.29 | <0.0001 | 0.0008 |
|  | 3 Avoiding thoughts | A | 78 | 0.05 | 0.4763 | 0.7326 | 7 | -0.25 | 0.4295 | 0.6920 | 113 | -0.30 | <0.0001 | 0.0008 |
|  | 4 Avoiding physically | A | 78 | 0.12 | 0.1190 | 0.3686 | 7 | 0.12 | 0.7087 | 0.8769 | 113 | -0.17 | 0.0065 | 0.0732 |
|  | 5 Overly cautious | A | 77 | 0.03 | 0.6972 | 0.8702 | 7 | -0.63 | 0.0483 | 0.2250 | 114 | -0.25 | 0.0001 | 0.0051 |
|  | 6 Nervousness | A | 78 | 0.11 | 0.1480 | 0.4061 | 7 | -0.25 | 0.4295 | 0.6920 | 116 | -0.27 | <0.0001 | 0.0020 |
|  | 7 Calming difficulty | A | 77 | 0.06 | 0.4501 | 0.7089 | 7 | 0.18 | 0.5647 | 0.7879 | 115 | -0.14 | 0.0278 | 0.1693 |
|  | 8 Emotional numbness | A | 77 | -0.01 | 0.8531 | 0.9378 | 7 | 0.24 | 0.4426 | 0.7023 | 114 | -0.14 | 0.0233 | 0.1566 |
|  | 9 Sense of failure | A | 77 | 0.01 | 0.8525 | 0.9376 | 7 | -0.06 | 0.8518 | 0.9373 | 116 | -0.12 | 0.0625 | 0.2604 |
|  | 10 Self-doubt | A | 77 | -0.03 | 0.6671 | 0.8526 | 7 | 0.00 | 1.0000 | 1.0000 | 117 | -0.20 | 0.0013 | 0.0260 |
|  | 11 Disconnection to others | A | 78 | -0.02 | 0.7849 | 0.9125 | 7 | 0.27 | 0.4003 | 0.6675 | 115 | -0.08 | 0.1992 | 0.4706 |
|  | 12 Social difficulty | A | 78 | -0.10 | 0.1745 | 0.4408 | 6 | 0.28 | 0.4345 | 0.6972 | 116 | -0.12 | 0.0655 | 0.2682 |
| 19 Loneliness feeling | 1 Bad dreams | D | 76 | 0.04 | 0.6500 | 0.8413 | 7 | -0.34 | 0.2863 | 0.5611 | 120 | -0.05 | 0.3814 | 0.6525 |
|  | 2 Reliving events in mind | D | 76 | -0.12 | 0.1346 | 0.3900 | 7 | -0.40 | 0.2125 | 0.4863 | 119 | -0.05 | 0.4583 | 0.7167 |
|  | 3 Avoiding thoughts | D | 76 | 0.10 | 0.2204 | 0.4938 | 7 | -0.46 | 0.1467 | 0.4044 | 116 | -0.08 | 0.2063 | 0.4804 |
|  | 4 Avoiding physically | D | 76 | 0.03 | 0.7268 | 0.8841 | 7 | 0.00 | 1.0000 | 1.0000 | 116 | -0.03 | 0.6526 | 0.8429 |
|  | 5 Overly cautious | D | 75 | -0.06 | 0.4766 | 0.7326 | 7 | -0.61 | 0.0530 | 0.2374 | 117 | -0.07 | 0.2971 | 0.5731 |
|  | 6 Nervousness | D | 76 | -0.09 | 0.2284 | 0.5034 | 7 | -0.15 | 0.6285 | 0.8325 | 119 | -0.15 | 0.0191 | 0.1375 |
|  | 7 Calming difficulty | D | 75 | -0.05 | 0.5018 | 0.7460 | 7 | 0.00 | 1.0000 | 1.0000 | 118 | -0.06 | 0.3491 | 0.6235 |
|  | 8 Emotional numbness | D | 75 | -0.05 | 0.5333 | 0.7697 | 7 | 0.00 | 1.0000 | 1.0000 | 117 | -0.10 | 0.1152 | 0.3644 |
|  | 9 Sense of failure | D | 76 | -0.15 | 0.0547 | 0.2406 | 7 | 0.07 | 0.8190 | 0.9285 | 119 | -0.11 | 0.0731 | 0.2863 |
|  | 10 Self-doubt | D | 75 | -0.10 | 0.2130 | 0.4868 | 7 | 0.15 | 0.6382 | 0.8355 | 119 | -0.12 | 0.0459 | 0.2197 |
|  | 11 Disconnection to others | D | 76 | -0.15 | 0.0615 | 0.2581 | 7 | 0.57 | 0.0714 | 0.2810 | 118 | 0.01 | 0.8767 | 0.9486 |
|  | 12 Social difficulty | D | 76 | 0.06 | 0.4352 | 0.6975 | 6 | 0.62 | 0.0805 | 0.2987 | 119 | -0.07 | 0.2568 | 0.5346 |
| 20 School fun | 1 Bad dreams | C | 76 | 0.15 | 0.0553 | 0.2427 | 6 | -0.21 | 0.5480 | 0.7772 | 118 | -0.08 | 0.1772 | 0.4434 |
|  | 2 Reliving events in mind | C | 76 | 0.01 | 0.8868 | 0.9537 | 6 | -0.36 | 0.3055 | 0.5824 | 117 | -0.16 | 0.0131 | 0.1111 |
|  | 3 Avoiding thoughts | C | 76 | 0.06 | 0.4244 | 0.6895 | 6 | -0.44 | 0.2201 | 0.4938 | 114 | -0.10 | 0.1039 | 0.3449 |
|  | 4 Avoiding physically | C | 76 | 0.14 | 0.0643 | 0.2648 | 6 | -0.16 | 0.6497 | 0.8413 | 114 | -0.10 | 0.1003 | 0.3389 |
|  | 5 Overly cautious | C | 75 | -0.13 | 0.0982 | 0.3349 | 6 | -0.59 | 0.0990 | 0.3367 | 115 | -0.16 | 0.0122 | 0.1081 |
|  | 6 Nervousness | C | 76 | -0.12 | 0.1172 | 0.3662 | 6 | -0.17 | 0.6374 | 0.8355 | 117 | -0.27 | <0.0001 | 0.0019 |
|  | 7 Calming difficulty | C | 75 | 0.03 | 0.7504 | 0.8934 | 6 | -0.17 | 0.6374 | 0.8355 | 116 | -0.17 | 0.0066 | 0.0737 |
|  | 8 Emotional numbness | C | 75 | 0.01 | 0.9317 | 0.9748 | 6 | -0.08 | 0.8137 | 0.9264 | 115 | -0.14 | 0.0269 | 0.1656 |
|  | 9 Sense of failure | C | 75 | 0.05 | 0.5546 | 0.7815 | 6 | 0.00 | 1.0000 | 1.0000 | 117 | -0.26 | <0.0001 | 0.0029 |
|  | 10 Self-doubt | C | 75 | -0.03 | 0.6698 | 0.8533 | 6 | 0.09 | 0.8062 | 0.9222 | 117 | -0.19 | 0.0027 | 0.0422 |
|  | 11 Disconnection to others | C | 76 | 0.02 | 0.7995 | 0.9192 | 6 | 0.52 | 0.1411 | 0.3971 | 116 | -0.16 | 0.0119 | 0.1068 |
|  | 12 Social difficulty | C | 76 | -0.08 | 0.3276 | 0.6038 | 5 | 0.82 | 0.0455 | 0.2194 | 117 | -0.12 | 0.0564 | 0.2458 |
| 21 Friends | 1 Bad dreams | D | 77 | 0.00 | 0.9580 | 0.9881 | 7 | -0.14 | 0.6523 | 0.8429 | 120 | 0.10 | 0.1230 | 0.3736 |
|  | 2 Reliving events in mind | D | 77 | -0.15 | 0.0482 | 0.2250 | 7 | -0.27 | 0.3993 | 0.6665 | 119 | -0.06 | 0.3294 | 0.6066 |
|  | 3 Avoiding thoughts | D | 77 | 0.16 | 0.0449 | 0.2177 | 7 | -0.32 | 0.3067 | 0.5827 | 116 | 0.04 | 0.5638 | 0.7871 |
|  | 4 Avoiding physically | D | 77 | 0.11 | 0.1748 | 0.4408 | 7 | 0.00 | 1.0000 | 1.0000 | 116 | 0.01 | 0.8253 | 0.9290 |
|  | 5 Overly cautious | D | 76 | -0.13 | 0.0919 | 0.3238 | 7 | -0.32 | 0.3067 | 0.5827 | 117 | 0.00 | 0.9843 | 1.0000 |
|  | 6 Nervousness | D | 77 | -0.17 | 0.0306 | 0.1773 | 7 | 0.00 | 1.0000 | 1.0000 | 119 | -0.11 | 0.0808 | 0.2993 |
|  | 7 Calming difficulty | D | 76 | -0.07 | 0.3688 | 0.6400 | 7 | 0.00 | 1.0000 | 1.0000 | 118 | -0.08 | 0.1948 | 0.4646 |
|  | 8 Emotional numbness | D | 76 | -0.11 | 0.1760 | 0.4418 | 7 | 0.06 | 0.8425 | 0.9329 | 117 | -0.03 | 0.6349 | 0.8355 |
|  | 9 Sense of failure | D | 76 | -0.10 | 0.1863 | 0.4546 | 7 | 0.18 | 0.5618 | 0.7861 | 119 | -0.09 | 0.1631 | 0.4269 |
|  | 10 Self-doubt | D | 76 | -0.17 | 0.0314 | 0.1782 | 7 | 0.25 | 0.4268 | 0.6907 | 119 | -0.02 | 0.7120 | 0.8786 |
|  | 11 Disconnection to others | D | 77 | -0.11 | 0.1506 | 0.4086 | 7 | 0.69 | 0.0295 | 0.1754 | 118 | 0.06 | 0.3600 | 0.6333 |
|  | 12 Social difficulty | D | 77 | -0.09 | 0.2489 | 0.5271 | 6 | 0.74 | 0.0371 | 0.1950 | 119 | 0.06 | 0.3697 | 0.6413 |
| 22 Dealing with school tasks | 1 Bad dreams | C | 77 | 0.09 | 0.2539 | 0.5324 | 5 | 0.41 | 0.3173 | 0.5928 | 118 | 0.03 | 0.6793 | 0.8593 |
|  | 2 Reliving events in mind | C | 77 | 0.06 | 0.4212 | 0.6854 | 5 | 0.41 | 0.3173 | 0.5928 | 117 | 0.02 | 0.7608 | 0.8994 |
|  | 3 Avoiding thoughts | C | 77 | 0.24 | 0.0019 | 0.0336 | 5 | 0.17 | 0.6831 | 0.8604 | 114 | 0.07 | 0.2636 | 0.5424 |
|  | 4 Avoiding physically | C | 77 | 0.25 | 0.0014 | 0.0280 | 5 | 0.67 | 0.1025 | 0.3421 | 114 | 0.09 | 0.1725 | 0.4398 |
|  | 5 Overly cautious | C | 76 | -0.04 | 0.6306 | 0.8333 | 5 | -0.17 | 0.6831 | 0.8604 | 115 | 0.04 | 0.4779 | 0.7340 |
|  | 6 Nervousness | C | 77 | -0.12 | 0.1384 | 0.3948 | 5 | 0.50 | 0.2207 | 0.4938 | 117 | -0.02 | 0.8050 | 0.9214 |
|  | 7 Calming difficulty | C | 76 | 0.08 | 0.3182 | 0.5935 | 5 | 0.67 | 0.1025 | 0.3421 | 116 | 0.04 | 0.5189 | 0.7592 |
|  | 8 Emotional numbness | C | 76 | 0.05 | 0.4855 | 0.7382 | 5 | 0.71 | 0.0833 | 0.3052 | 115 | 0.07 | 0.2670 | 0.5470 |
|  | 9 Sense of failure | C | 76 | 0.04 | 0.6355 | 0.8355 | 5 | 0.67 | 0.1025 | 0.3421 | 117 | 0.01 | 0.8372 | 0.9327 |
|  | 10 Self-doubt | C | 76 | 0.03 | 0.7263 | 0.8841 | 5 | 0.67 | 0.1025 | 0.3421 | 117 | -0.05 | 0.3936 | 0.6618 |
|  | 11 Disconnection to others | C | 77 | 0.09 | 0.2569 | 0.5346 | 5 | 0.76 | 0.0641 | 0.2648 | 116 | 0.02 | 0.8030 | 0.9205 |
|  | 12 Social difficulty | C | 77 | 0.07 | 0.3788 | 0.6503 | 4 | 0.71 | 0.1495 | 0.4073 | 117 | 0.04 | 0.4982 | 0.7449 |
| 23 Self-comparison to others | 1 Bad dreams | C | 79 | 0.06 | 0.4322 | 0.6949 | 7 | -0.11 | 0.7308 | 0.8864 | 120 | -0.10 | 0.1070 | 0.3506 |
|  | 2 Reliving events in mind | C | 79 | -0.16 | 0.0321 | 0.1807 | 7 | 0.00 | 1.0000 | 1.0000 | 119 | -0.04 | 0.5010 | 0.7458 |
|  | 3 Avoiding thoughts | C | 79 | 0.10 | 0.1884 | 0.4573 | 7 | -0.50 | 0.1184 | 0.3679 | 116 | -0.05 | 0.4476 | 0.7064 |
|  | 4 Avoiding physically | C | 79 | 0.03 | 0.7150 | 0.8800 | 7 | -0.19 | 0.5547 | 0.7815 | 116 | -0.05 | 0.3831 | 0.6535 |
|  | 5 Overly cautious | C | 78 | -0.09 | 0.2697 | 0.5471 | 7 | -0.59 | 0.0610 | 0.2565 | 117 | -0.01 | 0.8296 | 0.9297 |
|  | 6 Nervousness | C | 79 | -0.11 | 0.1365 | 0.3921 | 7 | -0.40 | 0.2116 | 0.4861 | 119 | -0.10 | 0.0976 | 0.3338 |
|  | 7 Calming difficulty | C | 78 | 0.06 | 0.4609 | 0.7190 | 7 | -0.10 | 0.7615 | 0.8994 | 118 | -0.12 | 0.0456 | 0.2197 |
|  | 8 Emotional numbness | C | 78 | -0.10 | 0.1948 | 0.4646 | 7 | 0.10 | 0.7615 | 0.8994 | 117 | -0.07 | 0.2888 | 0.5637 |
|  | 9 Sense of failure | C | 78 | -0.01 | 0.9284 | 0.9738 | 7 | -0.28 | 0.3755 | 0.6470 | 119 | -0.16 | 0.0115 | 0.1047 |
|  | 10 Self-doubt | C | 78 | -0.14 | 0.0668 | 0.2719 | 7 | -0.19 | 0.5439 | 0.7766 | 119 | -0.07 | 0.2743 | 0.5498 |
|  | 11 Disconnection to others | C | 79 | -0.05 | 0.5275 | 0.7666 | 7 | -0.11 | 0.7395 | 0.8895 | 118 | -0.02 | 0.7752 | 0.9066 |
|  | 12 Social difficulty | C | 79 | 0.08 | 0.3084 | 0.5847 | 6 | 0.12 | 0.7267 | 0.8841 | 119 | -0.01 | 0.8548 | 0.9390 |
| 24 Love-awareness | 1 Bad dreams | B | 79 | 0.19 | 0.0123 | 0.1081 | 7 | -0.40 | 0.2038 | 0.4778 | 121 | -0.22 | 0.0004 | 0.0134 |
|  | 2 Reliving events in mind | B | 79 | 0.03 | 0.6861 | 0.8627 | 7 | -0.45 | 0.1537 | 0.4133 | 120 | -0.13 | 0.0297 | 0.1754 |
|  | 3 Avoiding thoughts | B | 79 | 0.13 | 0.0846 | 0.3083 | 7 | -0.51 | 0.1064 | 0.3501 | 117 | -0.16 | 0.0111 | 0.1033 |
|  | 4 Avoiding physically | B | 79 | 0.14 | 0.0739 | 0.2882 | 7 | -0.07 | 0.8273 | 0.9295 | 117 | -0.04 | 0.4930 | 0.7423 |
|  | 5 Overly cautious | B | 78 | 0.03 | 0.6553 | 0.8447 | 7 | -0.66 | 0.0379 | 0.1972 | 118 | -0.08 | 0.1994 | 0.4708 |
|  | 6 Nervousness | B | 79 | -0.05 | 0.5384 | 0.7728 | 7 | -0.22 | 0.4890 | 0.7396 | 120 | -0.17 | 0.0047 | 0.0586 |
|  | 7 Calming difficulty | B | 78 | 0.08 | 0.2791 | 0.5544 | 7 | -0.07 | 0.8226 | 0.9290 | 119 | -0.14 | 0.0284 | 0.1714 |
|  | 8 Emotional numbness | B | 78 | -0.06 | 0.4714 | 0.7281 | 7 | -0.07 | 0.8226 | 0.9290 | 118 | -0.10 | 0.0933 | 0.3254 |
|  | 9 Sense of failure | B | 78 | 0.02 | 0.7492 | 0.8934 | 7 | 0.00 | 1.0000 | 1.0000 | 120 | -0.20 | 0.0011 | 0.0237 |
|  | 10 Self-doubt | B | 78 | -0.06 | 0.4342 | 0.6972 | 7 | 0.07 | 0.8226 | 0.9290 | 120 | -0.15 | 0.0176 | 0.1318 |
|  | 11 Disconnection to others | B | 79 | 0.02 | 0.8198 | 0.9290 | 7 | 0.47 | 0.1407 | 0.3965 | 119 | -0.08 | 0.1794 | 0.4465 |
|  | 12 Social difficulty | B | 79 | 0.02 | 0.7943 | 0.9175 | 6 | 0.62 | 0.0805 | 0.2987 | 120 | -0.06 | 0.3552 | 0.6291 |
| 25 Peer arguing | 1 Bad dreams | D | 79 | -0.01 | 0.9077 | 0.9652 | 7 | -0.23 | 0.4654 | 0.7227 | 121 | -0.07 | 0.2605 | 0.5372 |
|  | 2 Reliving events in mind | D | 79 | -0.14 | 0.0593 | 0.2531 | 7 | 0.00 | 1.0000 | 1.0000 | 120 | -0.07 | 0.2457 | 0.5243 |
|  | 3 Avoiding thoughts | D | 79 | 0.25 | 0.0012 | 0.0254 | 7 | -0.42 | 0.1852 | 0.4535 | 117 | -0.09 | 0.1557 | 0.4156 |
|  | 4 Avoiding physically | D | 79 | 0.05 | 0.5371 | 0.7716 | 7 | 0.00 | 1.0000 | 1.0000 | 117 | -0.06 | 0.3529 | 0.6270 |
|  | 5 Overly cautious | D | 78 | -0.07 | 0.3441 | 0.6188 | 7 | -0.35 | 0.2695 | 0.5470 | 118 | -0.10 | 0.1244 | 0.3751 |
|  | 6 Nervousness | D | 79 | -0.15 | 0.0445 | 0.2166 | 7 | -0.35 | 0.2695 | 0.5470 | 120 | -0.15 | 0.0159 | 0.1253 |
|  | 7 Calming difficulty | D | 78 | 0.04 | 0.5802 | 0.7994 | 7 | 0.07 | 0.8301 | 0.9297 | 119 | -0.06 | 0.3523 | 0.6267 |
|  | 8 Emotional numbness | D | 78 | -0.08 | 0.3296 | 0.6067 | 7 | 0.14 | 0.6678 | 0.8526 | 118 | -0.17 | 0.0054 | 0.0649 |
|  | 9 Sense of failure | D | 78 | -0.01 | 0.8707 | 0.9460 | 7 | -0.07 | 0.8345 | 0.9318 | 120 | -0.15 | 0.0187 | 0.1367 |
|  | 10 Self-doubt | D | 78 | -0.05 | 0.4800 | 0.7357 | 7 | -0.14 | 0.6678 | 0.8526 | 120 | -0.14 | 0.0208 | 0.1455 |
|  | 11 Disconnection to others | D | 79 | -0.03 | 0.7310 | 0.8864 | 7 | 0.22 | 0.4807 | 0.7357 | 119 | -0.08 | 0.2008 | 0.4723 |
|  | 12 Social difficulty | D | 79 | 0.16 | 0.0431 | 0.2142 | 6 | 0.10 | 0.7823 | 0.9110 | 120 | -0.11 | 0.0772 | 0.2952 |
| 26 Napping/dozing | 1 Bad dreams | A | 78 | 0.10 | 0.2164 | 0.4892 | 7 | -0.17 | 0.5940 | 0.8084 | 122 | -0.08 | 0.1697 | 0.4367 |
|  | 2 Reliving events in mind | A | 78 | 0.01 | 0.8568 | 0.9402 | 7 | 0.00 | 1.0000 | 1.0000 | 121 | -0.09 | 0.1295 | 0.3822 |
|  | 3 Avoiding thoughts | A | 78 | 0.09 | 0.2228 | 0.4963 | 7 | -0.38 | 0.2265 | 0.5004 | 118 | -0.10 | 0.0992 | 0.3369 |
|  | 4 Avoiding physically | A | 78 | 0.15 | 0.0479 | 0.2250 | 7 | 0.00 | 1.0000 | 1.0000 | 118 | -0.01 | 0.8635 | 0.9421 |
|  | 5 Overly cautious | A | 77 | 0.08 | 0.3266 | 0.6032 | 7 | -0.69 | 0.0295 | 0.1754 | 119 | 0.00 | 0.9938 | 1.0000 |
|  | 6 Nervousness | A | 78 | -0.03 | 0.6788 | 0.8591 | 7 | -0.31 | 0.3333 | 0.6095 | 121 | -0.05 | 0.3864 | 0.6557 |
|  | 7 Calming difficulty | A | 77 | -0.02 | 0.8436 | 0.9336 | 7 | 0.07 | 0.8141 | 0.9264 | 120 | 0.02 | 0.7893 | 0.9157 |
|  | 8 Emotional numbness | A | 77 | -0.01 | 0.9379 | 0.9762 | 7 | 0.15 | 0.6382 | 0.8355 | 119 | -0.05 | 0.4525 | 0.7112 |
|  | 9 Sense of failure | A | 77 | -0.09 | 0.2501 | 0.5291 | 7 | -0.22 | 0.4924 | 0.7423 | 121 | -0.10 | 0.1213 | 0.3718 |
|  | 10 Self-doubt | A | 77 | -0.09 | 0.2359 | 0.5129 | 7 | -0.15 | 0.6382 | 0.8355 | 121 | -0.03 | 0.5760 | 0.7960 |
|  | 11 Disconnection to others | A | 78 | -0.08 | 0.3271 | 0.6038 | 7 | -0.16 | 0.6065 | 0.8181 | 120 | -0.03 | 0.5995 | 0.8142 |
|  | 12 Social difficulty | A | 78 | 0.12 | 0.1163 | 0.3656 | 6 | -0.10 | 0.7823 | 0.9110 | 121 | -0.01 | 0.8877 | 0.9537 |
| 27 Eating problems | 1 Bad dreams | A | 79 | -0.03 | 0.6887 | 0.8644 | 7 | -0.14 | 0.6523 | 0.8429 | 118 | -0.02 | 0.8005 | 0.9192 |
|  | 2 Reliving events in mind | A | 79 | -0.09 | 0.2600 | 0.5368 | 7 | -0.27 | 0.3993 | 0.6665 | 118 | 0.00 | 0.9422 | 0.9795 |
|  | 3 Avoiding thoughts | A | 79 | 0.09 | 0.2232 | 0.4966 | 7 | 0.06 | 0.8380 | 0.9329 | 115 | 0.02 | 0.7979 | 0.9192 |
|  | 4 Avoiding physically | A | 79 | 0.12 | 0.1260 | 0.3784 | 7 | 0.12 | 0.6989 | 0.8711 | 115 | 0.01 | 0.8425 | 0.9329 |
|  | 5 Overly cautious | A | 78 | -0.08 | 0.3158 | 0.5922 | 7 | 0.00 | 1.0000 | 1.0000 | 116 | 0.02 | 0.7426 | 0.8909 |
|  | 6 Nervousness | A | 79 | -0.13 | 0.0786 | 0.2967 | 7 | 0.19 | 0.5397 | 0.7729 | 117 | -0.07 | 0.2826 | 0.5585 |
|  | 7 Calming difficulty | A | 78 | 0.08 | 0.3257 | 0.6025 | 7 | 0.06 | 0.8425 | 0.9329 | 116 | -0.04 | 0.4940 | 0.7426 |
|  | 8 Emotional numbness | A | 78 | 0.00 | 0.9898 | 1.0000 | 7 | -0.06 | 0.8425 | 0.9329 | 115 | 0.00 | 0.9654 | 0.9914 |
|  | 9 Sense of failure | A | 78 | 0.01 | 0.9135 | 0.9670 | 7 | 0.25 | 0.4392 | 0.6995 | 117 | -0.13 | 0.0453 | 0.2192 |
|  | 10 Self-doubt | A | 78 | -0.08 | 0.2774 | 0.5528 | 7 | 0.25 | 0.4268 | 0.6907 | 117 | -0.10 | 0.1285 | 0.3812 |
|  | 11 Disconnection to others | A | 79 | 0.03 | 0.7236 | 0.8834 | 7 | 0.48 | 0.1276 | 0.3800 | 116 | -0.09 | 0.1464 | 0.4044 |
|  | 12 Social difficulty | A | 79 | 0.16 | 0.0420 | 0.2110 | 6 | 0.28 | 0.4345 | 0.6972 | 117 | 0.02 | 0.7482 | 0.8934 |
| 28 Memorization | 1 Bad dreams | C | 79 | 0.19 | 0.0127 | 0.1095 | 7 | 0.15 | 0.6265 | 0.8319 | 122 | -0.20 | 0.0009 | 0.0208 |
|  | 2 Reliving events in mind | C | 79 | 0.00 | 0.9743 | 0.9962 | 7 | 0.36 | 0.2551 | 0.5324 | 121 | -0.12 | 0.0544 | 0.2399 |
|  | 3 Avoiding thoughts | C | 79 | 0.19 | 0.0131 | 0.1111 | 7 | -0.07 | 0.8252 | 0.9290 | 118 | -0.16 | 0.0099 | 0.0961 |
|  | 4 Avoiding physically | C | 79 | 0.18 | 0.0175 | 0.1311 | 7 | 0.00 | 1.0000 | 1.0000 | 118 | -0.11 | 0.0780 | 0.2956 |
|  | 5 Overly cautious | C | 78 | 0.08 | 0.3113 | 0.5884 | 7 | -0.35 | 0.2695 | 0.5470 | 119 | -0.12 | 0.0564 | 0.2458 |
|  | 6 Nervousness | C | 79 | 0.05 | 0.5351 | 0.7703 | 7 | -0.35 | 0.2695 | 0.5470 | 121 | -0.19 | 0.0015 | 0.0296 |
|  | 7 Calming difficulty | C | 78 | 0.25 | 0.0014 | 0.0274 | 7 | 0.07 | 0.8301 | 0.9297 | 120 | -0.16 | 0.0119 | 0.1066 |
|  | 8 Emotional numbness | C | 78 | 0.18 | 0.0219 | 0.1511 | 7 | 0.14 | 0.6678 | 0.8526 | 119 | -0.14 | 0.0257 | 0.1637 |
|  | 9 Sense of failure | C | 78 | 0.10 | 0.1752 | 0.4408 | 7 | -0.26 | 0.4034 | 0.6689 | 121 | -0.15 | 0.0167 | 0.1289 |
|  | 10 Self-doubt | C | 78 | 0.08 | 0.2841 | 0.5586 | 7 | -0.27 | 0.3907 | 0.6589 | 121 | -0.24 | 0.0001 | 0.0051 |
|  | 11 Disconnection to others | C | 79 | -0.04 | 0.5605 | 0.7858 | 7 | -0.15 | 0.6382 | 0.8355 | 120 | -0.11 | 0.0704 | 0.2791 |
|  | 12 Social difficulty | C | 79 | 0.22 | 0.0048 | 0.0600 | 6 | -0.20 | 0.5805 | 0.7994 | 121 | -0.20 | 0.0010 | 0.0224 |

*B-H* – Benjamini-Hochberg correction for multiple comparisons, *p*-values < 0.05 are indicated in red, N – number of participants, Tau – Tau correlation coefficient
