# Supplementary material for: Demographic predictors of trauma and depression in war-affected children from Poland and Ukraine: Implications for prevention of mental health problems
Source: Prev Med Rep. 2026 Feb 20;63:103422. doi: 10.1016/j.pmedr.2026.103422 (PMC12945576; doi:10.1016/j.pmedr.2026.103422)
Supplement: Supplementary file 6 — Supplementary material 6 [file mmc6.docx]

**CDI (panels A-D) and ITQ-CA correlations in interests/hobbies dependent groups**

**CDI2 –** **group A**

In CDI2 – group A, among the children who did not declare hobbies/interests, 27 significant and moderate correlations were observed, 23 of them were negative and four positive (Figure S6). The observed positive correlations were between CDI2-1 “Sandess” with ITQ-CA “Emotional numbness”, and CDI2-27 “Eating problems” with ITQ-CA “Relieving events in mind”, “Avoiding physically”, and “Overly cautions”. Among the negative correlations, most of them were between CDI2-18 “Pain thoughts” and CDI-17 “Eating attitude” with 10 and 9 ITQ-CA categories, respectively (Table S6). After Benjamini-Hochberg correction, nine significant correlations remain. One was positive CDI2-27 “Eating problems” with ITQ-CA “Relieving events in mind” The negative correlations were observed between CDI2-16 “Tiredness” with ITQ-CA “Social difficulty”, CDI2-17 “Eating attitude” with ITQ-CA “Avoiding thoughts” and CDI2-18 “Pain thoughts” with ITQ-CA “Bad dreams”, “Relieving events in mind”, “Avoiding thoughts”, “Avoiding physically”, “Overlay cautions”, and “Nervousness” (Table S6). In the group of children with hobbies, we observed 21 significant, very weak, or weak in strength, positive correlations between CDI2 and ITQ-CA responses. The correlations were between CDI2-1 “Sadness” with ITQ-CA “Bad dreams”, Avoiding thoughts”, “Nervousness”, “Calming difficulty”, “Emotional numbness”, “Sense of failure”, ”Self-doubt”, “Disconnection to others” and “Social difficulty, CDI2-9 “Tendency to cry” with ITQ-CA “Social difficulty”, CDI2-10 “Bad mood” and CDI2-17 “Eating attitude” with ITQ-CA “Bad dreams”, “Avoiding thoughts”, “Emotional numbness” or “Avoiding physically”, and “Social difficulty”. Additionally, CDI2-26 “Napping/dozing) was correlated with ITQ-CA “Avoiding physically”, and “Social difficulty”, and CDI-27 “Eating problems” with ITQ-CA “Social difficulty” (Table S6). All the correlation remain significant after correction for multiple comparisons (Table S6)

**CDI2 –** **group B**

In the CDI2 – group B among the children with no hobbies, we observed 29 significant correlations, negative, very weak, or weak correlations (Figure S6). Most significant correlations between CDI2-13 “Self-perception” (correlated with all ITQ-CA categories), CDI2-7 “Blame” with 8 ITQ-CA categories, and “Love awareness” with 5 ITQ-CA categories. (Table S6). After Benjamini-Hochberg correction, 11 correlations remains significant, CDI2-13 “Self-perception” with ITQ-CA “Bad dreams”, “Avoiding thoughts”, “Avoiding physically”, “Nervousness”, “Calming difficulty”, “Emotional numbness”, “Sense of failure”, ”Self-doubt”, “Disconnection to others”, and “Social difficulties”, and additionally CDI2-24 “Love-awareness” with ITQ-CA “Avoiding thoughts” (Table S6). In the group of children with hobbies, we observed 11 significant, positive, weak, or very weak correlations. Three of them remain significant after Benjamini-Hochberg correction: CDI2-2 “Hopeful” with ITQ-CA “Avoiding physically”, and CDI2-8 “Suicide” with ITQ-CA “Avoiding thoughts” and “Social difficulties” (Table S6).

**CDI2 –** **group C**

In the CDI2–group C among the children without hobbies, 25 significant correlations were observed (Figure S6). All of them are of weak to moderate strength and negative. Most significant correlations were between CDI2-20 “School fun”, CDI2-28 “Memorization”, and CDI2-4 “Enjoyment” with 8, 7, and 5 ITQ-CA categories, respectively (Table S6). After the Benjamini-Hochberg correction, 11 significant correlations remained (Table S6). The CDI-4 was correlated with OTQ-CA “Emotional numbness”, “Sense of failure”, “Disconnection to others”, and “Social difficulty”, CDI2-20 with ITQ-CA “Avoiding thoughts”, “Nervousness”, “Calming difficulty”, “Sense of failure”, “ Disconnection to others”, and “Social difficulties”. CDI2-28 “Memorization” was also correlated with ITQ-CA “Social difficulty”. In the group with hobbies, we observed five significant correlations (1 negative and four positive). The negative correlation was between CDI2-4 “Enjoyment” with ITQ-CA “Nervousness”, and positive between CDI2-3 “Self-confidence” with “Social difficulties”, and CDI2-22 “Dealing with friends” with ITQ-CA “Avoiding thoughts”, “Avoiding physically”, and “Emotional numbness” (Table S6).

**CDI2 –** **group D**

Among the children without hobbies/interests, 15 significant, weak correlations in CDI2–group D were observed (Figure S6), most negative and only one positive (between CDI2-21 “Friends” and ITQ-CA “Self-doubt”. Still, the negative correlations were between CDI2-5 “Importance for family”, CDI2-11 “Company”, CDI2-19 “Loneliness feeling”, and CDI2-21 “Friends” (with three ITQ-CA categories each), and CDI2-25 “Peer arguing”. After Benjamini-Hochberg correction, two significant correlations remain (CDI2-11 “Company” with ITQ-CA “Sense of failure” and CDI2-21 “Friends” with ITQ-CA “Bad dreams” (Table S6). In the group with hobbies, we observed nine significant, positive, and very weak or weak correlations. After Benjamini-Hochberg correction, four of them remain significant: CDI2-11 “Company”, CDI2-21 “Friends”, and “CDI-25 “Peer arguing” all with ITQ-CA “Avoiding thoughts”. Additionally, CDI2-21 was correlated significantly with ITQ-CA “Bad dreams” (Table S6).


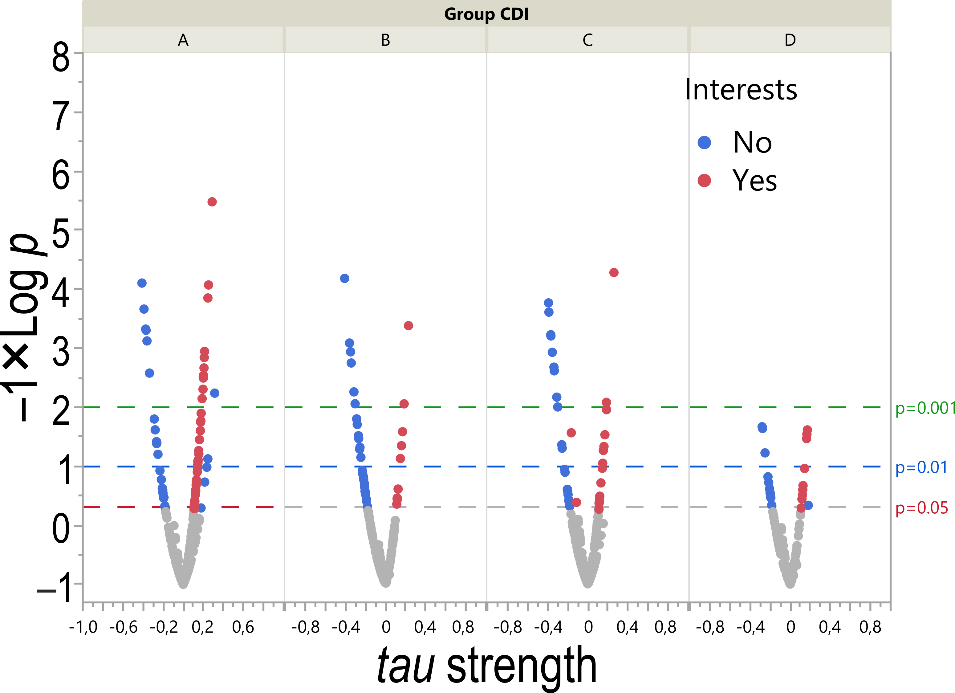


**Figure S6.** Correlations between single questions of CDI-2 (Children's Depression Inventory 2) and ITQ-CA (International Trauma Questionnaire – Child and Adolescent Version) in hobbies/interests dependent groups of children and adolescents from Poland and Ukraine. Volcano plot of significant correlations between single questions of CDI and ITQ-CA in the CDI A, B, C, and D groups. The strength of *tau* correlation coefficient (x-axis) and the significance as the unadjusted *p*-values (shown as −1×log *p*; y-axis). The dashed horizontal lines represent the *p* = 0.05 (red), *p* = 0.01 (blue), and *p* = 0.001 (green). Significant correlations are shown as colored spots, and non-significant correlations are grey. March 2024-March 2025, Poland and Ukraine.

**Table S6**. CDI-2 (Children's Depression Inventory 2) and ITQ-CA (International Trauma Questionnaire – Child and Adolescent Version) correlations in interests/hobbies dependent groups of children and adolescents from Poland and Ukraine, March 2024-March 2025, Poland and Ukraine.

| **CDI2** | **ITQ-CA** | **Group CDI** | **N** | ***Tau*** | ***p*-value** | ***p*-value ^BH^** | **N** | ***Tau*** | ***p*-value** | ***p*-value ^BH^** |
| --- | --- | --- | --- | --- | --- | --- | --- | --- | --- | --- |
|  |  | **Interests/Hobbies - YES** | | | | | **Interests/Hobbies - NO** | | | |
| 1 Sadness | 1 Bad dreams | A | 144 | 0.18 | 0.0018 | 0.0324 | 58 | 0.04 | 0.6486 | 0.8407 |
|  | 2 Reliving events in mind | A | 143 | 0.10 | 0.0648 | 0.2664 | 58 | 0.07 | 0.4633 | 0.7211 |
|  | 3 Avoiding thoughts | A | 142 | 0.25 | <0.0001 | 0.0018 | 57 | 0.13 | 0.1616 | 0.4252 |
|  | 4 Avoiding physically | A | 141 | 0.16 | 0.0062 | 0.0715 | 57 | 0.18 | 0.0509 | 0.2326 |
|  | 5 Overly cautious | A | 141 | 0.11 | 0.0520 | 0.2356 | 57 | 0.06 | 0.4881 | 0.7393 |
|  | 6 Nervousness | A | 144 | 0.19 | 0.0007 | 0.0182 | 57 | 0.06 | 0.5335 | 0.7697 |
|  | 7 Calming difficulty | A | 142 | 0.25 | <0.0001 | 0.0012 | 57 | 0.08 | 0.3854 | 0.6552 |
|  | 8 Emotional numbness | A | 142 | 0.20 | 0.0005 | 0.0142 | 57 | 0.21 | 0.0185 | 0.1359 |
|  | 9 Sense of failure | A | 143 | 0.18 | 0.0017 | 0.0310 | 57 | 0.12 | 0.2063 | 0.4804 |
|  | 10 Self-doubt | A | 142 | 0.20 | 0.0005 | 0.0143 | 58 | 0.16 | 0.0774 | 0.2952 |
|  | 11 Disconnection to others | A | 143 | 0.17 | 0.0025 | 0.0400 | 57 | -0.04 | 0.6242 | 0.8303 |
|  | 12 Social difficulty | A | 143 | 0.29 | <0.0001 | <0.0001 | 57 | 0.00 | 0.9869 | 1.0000 |
| 2 Hopeful | 1 Bad dreams | B | 150 | 0.06 | 0.2962 | 0.5719 | 58 | -0.15 | 0.0924 | 0.3244 |
|  | 2 Reliving events in mind | B | 149 | -0.05 | 0.4097 | 0.6739 | 58 | -0.08 | 0.3633 | 0.6360 |
|  | 3 Avoiding thoughts | B | 147 | 0.07 | 0.2160 | 0.4890 | 57 | -0.15 | 0.1087 | 0.3531 |
|  | 4 Avoiding physically | B | 147 | 0.19 | 0.0009 | 0.0207 | 57 | -0.05 | 0.6031 | 0.8164 |
|  | 5 Overly cautious | B | 147 | -0.03 | 0.5943 | 0.8087 | 57 | -0.10 | 0.2640 | 0.5428 |
|  | 6 Nervousness | B | 150 | -0.07 | 0.2146 | 0.4875 | 57 | -0.11 | 0.2251 | 0.4988 |
|  | 7 Calming difficulty | B | 148 | 0.08 | 0.1339 | 0.3889 | 57 | -0.08 | 0.3670 | 0.6392 |
|  | 8 Emotional numbness | B | 147 | 0.11 | 0.0438 | 0.2153 | 57 | -0.12 | 0.1943 | 0.4646 |
|  | 9 Sense of failure | B | 149 | 0.01 | 0.8988 | 0.9603 | 57 | -0.18 | 0.0485 | 0.2256 |
|  | 10 Self-doubt | B | 148 | 0.04 | 0.5070 | 0.7497 | 58 | -0.21 | 0.0196 | 0.1389 |
|  | 11 Disconnection to others | B | 149 | 0.02 | 0.7187 | 0.8824 | 57 | -0.15 | 0.0968 | 0.3328 |
|  | 12 Social difficulty | B | 149 | 0.12 | 0.0345 | 0.1870 | 57 | -0.10 | 0.2737 | 0.5496 |
| 3 Self-confidence | 1 Bad dreams | C | 150 | 0.11 | 0.0423 | 0.2118 | 58 | 0.00 | 1.0000 | 1.0000 |
|  | 2 Reliving events in mind | C | 149 | -0.01 | 0.9129 | 0.9669 | 58 | -0.02 | 0.7991 | 0.9192 |
|  | 3 Avoiding thoughts | C | 147 | 0.11 | 0.0406 | 0.2064 | 57 | -0.05 | 0.5608 | 0.7858 |
|  | 4 Avoiding physically | C | 147 | 0.09 | 0.1011 | 0.3404 | 57 | 0.08 | 0.3542 | 0.6284 |
|  | 5 Overly cautious | C | 147 | 0.00 | 0.9730 | 0.9954 | 57 | -0.07 | 0.4475 | 0.7064 |
|  | 6 Nervousness | C | 150 | 0.01 | 0.8568 | 0.9402 | 57 | -0.15 | 0.0942 | 0.3269 |
|  | 7 Calming difficulty | C | 148 | 0.15 | 0.0054 | 0.0649 | 57 | -0.11 | 0.2329 | 0.5091 |
|  | 8 Emotional numbness | C | 147 | 0.07 | 0.1795 | 0.4465 | 57 | 0.01 | 0.9441 | 0.9804 |
|  | 9 Sense of failure | C | 149 | 0.05 | 0.3683 | 0.6400 | 57 | -0.09 | 0.3434 | 0.6186 |
|  | 10 Self-doubt | C | 148 | -0.01 | 0.7954 | 0.9181 | 58 | -0.05 | 0.5551 | 0.7816 |
|  | 11 Disconnection to others | C | 149 | 0.09 | 0.1167 | 0.3656 | 57 | -0.26 | 0.0043 | 0.0561 |
|  | 12 Social difficulty | C | 149 | 0.18 | 0.0008 | 0.0203 | 57 | -0.23 | 0.0113 | 0.1040 |
| 4 Enjoyment | 1 Bad dreams | C | 150 | 0.01 | 0.8415 | 0.9329 | 57 | -0.12 | 0.1697 | 0.4367 |
|  | 2 Reliving events in mind | C | 149 | -0.08 | 0.1275 | 0.3800 | 57 | 0.07 | 0.4204 | 0.6851 |
|  | 3 Avoiding thoughts | C | 147 | 0.07 | 0.2265 | 0.5004 | 56 | -0.06 | 0.5403 | 0.7732 |
|  | 4 Avoiding physically | C | 147 | 0.10 | 0.0724 | 0.2839 | 56 | 0.03 | 0.7118 | 0.8786 |
|  | 5 Overly cautious | C | 147 | -0.08 | 0.1741 | 0.4408 | 56 | 0.06 | 0.4819 | 0.7364 |
|  | 6 Nervousness | C | 150 | -0.17 | 0.0027 | 0.0422 | 56 | -0.10 | 0.2772 | 0.5526 |
|  | 7 Calming difficulty | C | 148 | -0.04 | 0.4999 | 0.7458 | 56 | -0.12 | 0.1869 | 0.4553 |
|  | 8 Emotional numbness | C | 147 | -0.07 | 0.1943 | 0.4646 | 56 | -0.39 | <0.0001 | 0.0021 |
|  | 9 Sense of failure | C | 149 | -0.02 | 0.7371 | 0.8895 | 56 | -0.37 | 0.0001 | 0.0037 |
|  | 10 Self-doubt | C | 148 | -0.03 | 0.6496 | 0.8413 | 57 | -0.20 | 0.0302 | 0.1769 |
|  | 11 Disconnection to others | C | 149 | 0.02 | 0.7779 | 0.9088 | 56 | -0.37 | 0.0001 | 0.0038 |
|  | 12 Social difficulty | C | 149 | 0.00 | 0.9322 | 0.9748 | 56 | -0.35 | 0.0001 | 0.0055 |
| 5 Importance for family | 1 Bad dreams | D | 150 | 0.01 | 0.7886 | 0.9157 | 57 | -0.25 | 0.0059 | 0.0700 |
|  | 2 Reliving events in mind | D | 149 | -0.07 | 0.1768 | 0.4434 | 57 | -0.12 | 0.1701 | 0.4367 |
|  | 3 Avoiding thoughts | D | 147 | 0.06 | 0.3038 | 0.5806 | 56 | -0.17 | 0.0591 | 0.2525 |
|  | 4 Avoiding physically | D | 147 | 0.06 | 0.2714 | 0.5485 | 56 | -0.12 | 0.1961 | 0.4658 |
|  | 5 Overly cautious | D | 147 | -0.05 | 0.3598 | 0.6333 | 56 | -0.12 | 0.2106 | 0.4851 |
|  | 6 Nervousness | D | 150 | -0.02 | 0.7232 | 0.8834 | 56 | -0.13 | 0.1659 | 0.4306 |
|  | 7 Calming difficulty | D | 148 | 0.04 | 0.4260 | 0.6905 | 56 | -0.09 | 0.3126 | 0.5900 |
|  | 8 Emotional numbness | D | 147 | -0.09 | 0.1208 | 0.3707 | 56 | -0.10 | 0.2676 | 0.5470 |
|  | 9 Sense of failure | D | 149 | -0.02 | 0.7237 | 0.8834 | 56 | -0.18 | 0.0457 | 0.2197 |
|  | 10 Self-doubt | D | 148 | 0.00 | 0.9347 | 0.9757 | 57 | -0.22 | 0.0151 | 0.1215 |
|  | 11 Disconnection to others | D | 149 | 0.04 | 0.4800 | 0.7357 | 56 | -0.16 | 0.0889 | 0.3176 |
|  | 12 Social difficulty | D | 149 | 0.05 | 0.4121 | 0.6764 | 56 | -0.11 | 0.2349 | 0.5118 |
| 6 Self-acceptance | 1 Bad dreams | B | 150 | 0.12 | 0.0350 | 0.1884 | 57 | -0.16 | 0.0746 | 0.2893 |
|  | 2 Reliving events in mind | B | 149 | -0.02 | 0.6925 | 0.8667 | 57 | 0.01 | 0.9019 | 0.9616 |
|  | 3 Avoiding thoughts | B | 147 | 0.10 | 0.0829 | 0.3044 | 56 | -0.10 | 0.2867 | 0.5616 |
|  | 4 Avoiding physically | B | 147 | 0.07 | 0.2180 | 0.4909 | 56 | -0.02 | 0.8283 | 0.9297 |
|  | 5 Overly cautious | B | 147 | 0.04 | 0.5091 | 0.7514 | 56 | -0.05 | 0.5735 | 0.7946 |
|  | 6 Nervousness | B | 150 | -0.05 | 0.3572 | 0.6314 | 56 | -0.13 | 0.1462 | 0.4044 |
|  | 7 Calming difficulty | B | 148 | -0.02 | 0.6756 | 0.8567 | 56 | -0.09 | 0.3542 | 0.6284 |
|  | 8 Emotional numbness | B | 147 | 0.03 | 0.5741 | 0.7949 | 56 | 0.04 | 0.6831 | 0.8604 |
|  | 9 Sense of failure | B | 149 | -0.02 | 0.7274 | 0.8846 | 56 | -0.04 | 0.6707 | 0.8540 |
|  | 10 Self-doubt | B | 148 | 0.08 | 0.1703 | 0.4367 | 57 | -0.02 | 0.7924 | 0.9160 |
|  | 11 Disconnection to others | B | 149 | 0.08 | 0.1547 | 0.4146 | 56 | -0.26 | 0.0051 | 0.0625 |
|  | 12 Social difficulty | B | 149 | 0.15 | 0.0074 | 0.0799 | 56 | -0.23 | 0.0134 | 0.1120 |
| 7 Blame | 1 Bad dreams | B | 149 | 0.07 | 0.2077 | 0.4812 | 57 | -0.21 | 0.0226 | 0.1534 |
|  | 2 Reliving events in mind | B | 148 | -0.01 | 0.8438 | 0.9336 | 57 | -0.15 | 0.0934 | 0.3254 |
|  | 3 Avoiding thoughts | B | 146 | 0.09 | 0.1023 | 0.3421 | 56 | -0.20 | 0.0287 | 0.1726 |
|  | 4 Avoiding physically | B | 146 | 0.09 | 0.1205 | 0.3707 | 56 | -0.05 | 0.6233 | 0.8295 |
|  | 5 Overly cautious | B | 146 | 0.08 | 0.1510 | 0.4091 | 56 | -0.14 | 0.1192 | 0.3686 |
|  | 6 Nervousness | B | 149 | -0.03 | 0.5354 | 0.7703 | 56 | -0.19 | 0.0387 | 0.2001 |
|  | 7 Calming difficulty | B | 147 | 0.02 | 0.6678 | 0.8526 | 56 | -0.20 | 0.0264 | 0.1643 |
|  | 8 Emotional numbness | B | 146 | 0.05 | 0.3884 | 0.6574 | 56 | -0.09 | 0.3499 | 0.6243 |
|  | 9 Sense of failure | B | 148 | -0.01 | 0.9172 | 0.9693 | 56 | -0.23 | 0.0118 | 0.1066 |
|  | 10 Self-doubt | B | 147 | 0.00 | 0.9526 | 0.9852 | 57 | -0.19 | 0.0365 | 0.1938 |
|  | 11 Disconnection to others | B | 148 | -0.06 | 0.3017 | 0.5794 | 56 | -0.20 | 0.0297 | 0.1754 |
|  | 12 Social difficulty | B | 148 | 0.00 | 0.9353 | 0.9757 | 56 | -0.18 | 0.0566 | 0.2460 |
| 8 Suicide | 1 Bad dreams | B | 147 | 0.13 | 0.0243 | 0.1602 | 58 | -0.15 | 0.1006 | 0.3398 |
|  | 2 Reliving events in mind | B | 146 | -0.05 | 0.3881 | 0.6573 | 58 | -0.06 | 0.5308 | 0.7684 |
|  | 3 Avoiding thoughts | B | 144 | 0.17 | 0.0026 | 0.0412 | 57 | -0.13 | 0.1625 | 0.4258 |
|  | 4 Avoiding physically | B | 144 | 0.08 | 0.1752 | 0.4408 | 57 | -0.06 | 0.4801 | 0.7357 |
|  | 5 Overly cautious | B | 144 | 0.01 | 0.9202 | 0.9709 | 57 | -0.03 | 0.7209 | 0.8824 |
|  | 6 Nervousness | B | 147 | -0.01 | 0.7945 | 0.9175 | 57 | -0.07 | 0.4688 | 0.7250 |
|  | 7 Calming difficulty | B | 145 | 0.02 | 0.6914 | 0.8663 | 57 | -0.04 | 0.6682 | 0.8529 |
|  | 8 Emotional numbness | B | 144 | 0.07 | 0.1981 | 0.4692 | 57 | -0.17 | 0.0586 | 0.2513 |
|  | 9 Sense of failure | B | 146 | -0.02 | 0.7183 | 0.8823 | 57 | -0.16 | 0.0786 | 0.2967 |
|  | 10 Self-doubt | B | 145 | 0.03 | 0.6152 | 0.8243 | 58 | -0.21 | 0.0197 | 0.1398 |
|  | 11 Disconnection to others | B | 146 | 0.06 | 0.2705 | 0.5476 | 57 | -0.03 | 0.7038 | 0.8749 |
|  | 12 Social difficulty | B | 146 | 0.23 | <0.0001 | 0.0031 | 57 | -0.08 | 0.4096 | 0.6739 |
| 9 Tendency to cry | 1 Bad dreams | A | 149 | 0.11 | 0.0381 | 0.1977 | 57 | -0.13 | 0.1503 | 0.4083 |
|  | 2 Reliving events in mind | A | 148 | -0.08 | 0.1614 | 0.4248 | 57 | -0.10 | 0.2838 | 0.5586 |
|  | 3 Avoiding thoughts | A | 146 | 0.11 | 0.0560 | 0.2449 | 56 | -0.13 | 0.1715 | 0.4381 |
|  | 4 Avoiding physically | A | 146 | 0.08 | 0.1402 | 0.3963 | 56 | -0.01 | 0.9276 | 0.9737 |
|  | 5 Overly cautious | A | 146 | 0.06 | 0.2693 | 0.5470 | 56 | -0.13 | 0.1650 | 0.4298 |
|  | 6 Nervousness | A | 149 | -0.06 | 0.2957 | 0.5716 | 56 | -0.18 | 0.0471 | 0.2227 |
|  | 7 Calming difficulty | A | 147 | 0.04 | 0.4266 | 0.6907 | 56 | -0.10 | 0.2739 | 0.5496 |
|  | 8 Emotional numbness | A | 146 | 0.10 | 0.0785 | 0.2967 | 56 | -0.04 | 0.6416 | 0.8375 |
|  | 9 Sense of failure | A | 148 | 0.01 | 0.8903 | 0.9559 | 56 | -0.10 | 0.2726 | 0.5496 |
|  | 10 Self-doubt | A | 147 | 0.05 | 0.3869 | 0.6557 | 57 | -0.14 | 0.1329 | 0.3867 |
|  | 11 Disconnection to others | A | 148 | 0.08 | 0.1568 | 0.4180 | 56 | -0.11 | 0.2241 | 0.4977 |
|  | 12 Social difficulty | A | 148 | 0.18 | 0.0013 | 0.0260 | 56 | -0.11 | 0.2379 | 0.5148 |
| 10 Bad mood | 1 Bad dreams | A | 150 | 0.21 | 0.0001 | 0.0064 | 58 | -0.19 | 0.0336 | 0.1848 |
|  | 2 Reliving events in mind | A | 149 | 0.00 | 0.9360 | 0.9758 | 58 | 0.01 | 0.8705 | 0.9460 |
|  | 3 Avoiding thoughts | A | 147 | 0.20 | 0.0003 | 0.0102 | 57 | -0.04 | 0.6493 | 0.8413 |
|  | 4 Avoiding physically | A | 147 | 0.15 | 0.0054 | 0.0649 | 57 | 0.10 | 0.2718 | 0.5489 |
|  | 5 Overly cautious | A | 147 | 0.12 | 0.0305 | 0.1771 | 57 | 0.04 | 0.6988 | 0.8711 |
|  | 6 Nervousness | A | 150 | -0.02 | 0.6727 | 0.8552 | 57 | -0.07 | 0.4193 | 0.6842 |
|  | 7 Calming difficulty | A | 148 | 0.12 | 0.0251 | 0.1619 | 57 | -0.02 | 0.8394 | 0.9329 |
|  | 8 Emotional numbness | A | 147 | 0.17 | 0.0018 | 0.0323 | 57 | -0.14 | 0.1126 | 0.3588 |
|  | 9 Sense of failure | A | 149 | 0.06 | 0.2483 | 0.5265 | 57 | -0.12 | 0.1800 | 0.4469 |
|  | 10 Self-doubt | A | 148 | 0.09 | 0.1217 | 0.3723 | 58 | 0.05 | 0.5610 | 0.7860 |
|  | 11 Disconnection to others | A | 149 | 0.06 | 0.2460 | 0.5243 | 57 | 0.07 | 0.4140 | 0.6781 |
|  | 12 Social difficulty | A | 149 | 0.21 | 0.0001 | 0.0055 | 57 | 0.00 | 0.9848 | 1.0000 |
| 11 Company | 1 Bad dreams | D | 149 | 0.10 | 0.0624 | 0.2600 | 58 | -0.11 | 0.2388 | 0.5157 |
|  | 2 Reliving events in mind | D | 148 | 0.06 | 0.2594 | 0.5368 | 58 | -0.11 | 0.2146 | 0.4875 |
|  | 3 Avoiding thoughts | D | 146 | 0.17 | 0.0028 | 0.0428 | 57 | -0.20 | 0.0282 | 0.1703 |
|  | 4 Avoiding physically | D | 146 | 0.12 | 0.0359 | 0.1914 | 57 | 0.07 | 0.4722 | 0.7286 |
|  | 5 Overly cautious | D | 146 | 0.02 | 0.6777 | 0.8583 | 57 | -0.02 | 0.8144 | 0.9264 |
|  | 6 Nervousness | D | 149 | 0.01 | 0.8592 | 0.9402 | 57 | 0.00 | 0.9798 | 0.9999 |
|  | 7 Calming difficulty | D | 147 | 0.04 | 0.4405 | 0.7004 | 57 | -0.03 | 0.7808 | 0.9108 |
|  | 8 Emotional numbness | D | 146 | 0.10 | 0.0668 | 0.2719 | 57 | -0.20 | 0.0296 | 0.1754 |
|  | 9 Sense of failure | D | 148 | -0.09 | 0.1097 | 0.3548 | 57 | -0.28 | 0.0021 | 0.0366 |
|  | 10 Self-doubt | D | 147 | -0.02 | 0.7431 | 0.8910 | 58 | -0.10 | 0.2483 | 0.5265 |
|  | 11 Disconnection to others | D | 148 | 0.04 | 0.4612 | 0.7193 | 57 | -0.04 | 0.6768 | 0.8577 |
|  | 12 Social difficulty | D | 148 | 0.07 | 0.2208 | 0.4938 | 57 | 0.02 | 0.8572 | 0.9402 |
| 12 Self-determination | 1 Bad dreams | C | 149 | 0.02 | 0.7109 | 0.8782 | 58 | -0.06 | 0.4864 | 0.7382 |
|  | 2 Reliving events in mind | C | 148 | -0.01 | 0.8501 | 0.9364 | 58 | -0.02 | 0.7991 | 0.9192 |
|  | 3 Avoiding thoughts | C | 146 | -0.01 | 0.8461 | 0.9343 | 57 | -0.05 | 0.5637 | 0.7871 |
|  | 4 Avoiding physically | C | 146 | 0.08 | 0.1419 | 0.3983 | 57 | 0.00 | 0.9572 | 0.9877 |
|  | 5 Overly cautious | C | 146 | 0.03 | 0.6410 | 0.8373 | 57 | -0.08 | 0.4037 | 0.6692 |
|  | 6 Nervousness | C | 149 | 0.00 | 0.9285 | 0.9738 | 57 | -0.12 | 0.1964 | 0.4662 |
|  | 7 Calming difficulty | C | 147 | 0.04 | 0.4359 | 0.6976 | 57 | -0.11 | 0.2352 | 0.5120 |
|  | 8 Emotional numbness | C | 146 | 0.04 | 0.4440 | 0.7039 | 57 | -0.07 | 0.4554 | 0.7144 |
|  | 9 Sense of failure | C | 148 | 0.06 | 0.2417 | 0.5195 | 57 | -0.07 | 0.4673 | 0.7243 |
|  | 10 Self-doubt | C | 147 | -0.01 | 0.8699 | 0.9460 | 58 | -0.11 | 0.2301 | 0.5056 |
|  | 11 Disconnection to others | C | 148 | 0.12 | 0.0363 | 0.1931 | 57 | -0.14 | 0.1316 | 0.3849 |
|  | 12 Social difficulty | C | 148 | 0.12 | 0.0321 | 0.1806 | 57 | -0.19 | 0.0373 | 0.1953 |
| 13 Self-perception | 1 Bad dreams | B | 149 | 0.02 | 0.7268 | 0.8841 | 58 | -0.35 | 0.0001 | 0.0055 |
|  | 2 Reliving events in mind | B | 148 | -0.01 | 0.9076 | 0.9652 | 58 | -0.25 | 0.0048 | 0.0596 |
|  | 3 Avoiding thoughts | B | 146 | 0.07 | 0.2068 | 0.4808 | 57 | -0.34 | 0.0002 | 0.0073 |
|  | 4 Avoiding physically | B | 146 | 0.12 | 0.0367 | 0.1939 | 57 | -0.30 | 0.0009 | 0.0207 |
|  | 5 Overly cautious | B | 146 | 0.01 | 0.8272 | 0.9295 | 57 | -0.25 | 0.0071 | 0.0774 |
|  | 6 Nervousness | B | 149 | -0.06 | 0.2771 | 0.5526 | 57 | -0.29 | 0.0016 | 0.0299 |
|  | 7 Calming difficulty | B | 147 | 0.00 | 0.9588 | 0.9886 | 57 | -0.27 | 0.0030 | 0.0445 |
|  | 8 Emotional numbness | B | 146 | 0.01 | 0.9121 | 0.9667 | 57 | -0.31 | 0.0005 | 0.0152 |
|  | 9 Sense of failure | B | 148 | 0.01 | 0.8705 | 0.9460 | 57 | -0.36 | 0.0001 | 0.0047 |
|  | 10 Self-doubt | B | 147 | 0.04 | 0.4868 | 0.7383 | 58 | -0.41 | <0.0001 | 0.0012 |
|  | 11 Disconnection to others | B | 148 | 0.04 | 0.4616 | 0.7195 | 57 | -0.27 | 0.0034 | 0.0478 |
|  | 12 Social difficulty | B | 148 | 0.12 | 0.0345 | 0.1870 | 57 | -0.28 | 0.0020 | 0.0344 |
| 14 Learning attitude | 1 Bad dreams | C | 149 | 0.09 | 0.0912 | 0.3230 | 58 | 0.05 | 0.5694 | 0.7908 |
|  | 2 Reliving events in mind | C | 148 | 0.01 | 0.9197 | 0.9709 | 58 | 0.06 | 0.4787 | 0.7347 |
|  | 3 Avoiding thoughts | C | 146 | 0.10 | 0.0884 | 0.3165 | 57 | 0.09 | 0.3345 | 0.6107 |
|  | 4 Avoiding physically | C | 146 | 0.04 | 0.4427 | 0.7023 | 57 | 0.08 | 0.3817 | 0.6528 |
|  | 5 Overly cautious | C | 146 | 0.04 | 0.5284 | 0.7675 | 57 | 0.13 | 0.1482 | 0.4061 |
|  | 6 Nervousness | C | 149 | -0.11 | 0.0408 | 0.2067 | 57 | 0.00 | 0.9806 | 1.0000 |
|  | 7 Calming difficulty | C | 147 | 0.03 | 0.5551 | 0.7816 | 57 | 0.06 | 0.4838 | 0.7375 |
|  | 8 Emotional numbness | C | 146 | 0.00 | 0.9689 | 0.9929 | 57 | -0.03 | 0.7234 | 0.8834 |
|  | 9 Sense of failure | C | 148 | 0.03 | 0.5465 | 0.7767 | 57 | 0.11 | 0.2124 | 0.4863 |
|  | 10 Self-doubt | C | 147 | -0.06 | 0.2913 | 0.5665 | 58 | -0.04 | 0.6619 | 0.8501 |
|  | 11 Disconnection to others | C | 148 | -0.03 | 0.5894 | 0.8061 | 57 | 0.15 | 0.0932 | 0.3254 |
|  | 12 Social difficulty | C | 148 | 0.04 | 0.4816 | 0.7363 | 57 | -0.03 | 0.7080 | 0.8767 |
| 15 Sleep quality | 1 Bad dreams | A | 149 | 0.15 | 0.0082 | 0.0848 | 58 | 0.00 | 0.9893 | 1.0000 |
|  | 2 Reliving events in mind | A | 148 | 0.14 | 0.0098 | 0.0954 | 58 | -0.02 | 0.8609 | 0.9404 |
|  | 3 Avoiding thoughts | A | 146 | 0.13 | 0.0179 | 0.1324 | 57 | 0.03 | 0.7231 | 0.8834 |
|  | 4 Avoiding physically | A | 146 | 0.04 | 0.4967 | 0.7437 | 57 | 0.07 | 0.4356 | 0.6975 |
|  | 5 Overly cautious | A | 146 | 0.13 | 0.0208 | 0.1455 | 57 | -0.07 | 0.4513 | 0.7100 |
|  | 6 Nervousness | A | 149 | -0.01 | 0.7889 | 0.9157 | 57 | -0.09 | 0.3317 | 0.6078 |
|  | 7 Calming difficulty | A | 147 | 0.06 | 0.3094 | 0.5859 | 57 | -0.09 | 0.3163 | 0.5923 |
|  | 8 Emotional numbness | A | 146 | 0.10 | 0.0703 | 0.2791 | 57 | 0.05 | 0.5681 | 0.7898 |
|  | 9 Sense of failure | A | 148 | 0.00 | 0.9629 | 0.9902 | 57 | 0.05 | 0.5911 | 0.8075 |
|  | 10 Self-doubt | A | 147 | 0.08 | 0.1515 | 0.4095 | 58 | 0.00 | 1.0000 | 1.0000 |
|  | 11 Disconnection to others | A | 148 | 0.02 | 0.7421 | 0.8906 | 57 | -0.08 | 0.3902 | 0.6589 |
|  | 12 Social difficulty | A | 148 | 0.13 | 0.0171 | 0.1302 | 57 | -0.16 | 0.0751 | 0.2901 |
| 16 Tiredness level | 1 Bad dreams | A | 147 | 0.10 | 0.0691 | 0.2761 | 58 | -0.03 | 0.7432 | 0.8910 |
|  | 2 Reliving events in mind | A | 146 | -0.01 | 0.8284 | 0.9297 | 58 | -0.05 | 0.5619 | 0.7861 |
|  | 3 Avoiding thoughts | A | 144 | 0.12 | 0.0265 | 0.1643 | 57 | -0.05 | 0.6048 | 0.8171 |
|  | 4 Avoiding physically | A | 144 | 0.08 | 0.1820 | 0.4495 | 57 | -0.02 | 0.8002 | 0.9192 |
|  | 5 Overly cautious | A | 144 | 0.01 | 0.8070 | 0.9224 | 57 | -0.09 | 0.3409 | 0.6161 |
|  | 6 Nervousness | A | 147 | 0.00 | 0.9783 | 0.9993 | 57 | -0.15 | 0.1080 | 0.3521 |
|  | 7 Calming difficulty | A | 145 | 0.07 | 0.2391 | 0.5161 | 57 | -0.18 | 0.0510 | 0.2326 |
|  | 8 Emotional numbness | A | 144 | 0.04 | 0.5068 | 0.7497 | 57 | -0.03 | 0.7313 | 0.8866 |
|  | 9 Sense of failure | A | 146 | 0.01 | 0.9226 | 0.9718 | 57 | -0.04 | 0.6707 | 0.8540 |
|  | 10 Self-doubt | A | 145 | -0.05 | 0.3802 | 0.6513 | 58 | -0.05 | 0.5678 | 0.7898 |
|  | 11 Disconnection to others | A | 146 | 0.05 | 0.3826 | 0.6534 | 57 | -0.22 | 0.0168 | 0.1294 |
|  | 12 Social difficulty | A | 146 | 0.14 | 0.0108 | 0.1018 | 57 | -0.29 | 0.0016 | 0.0299 |
| 17 Eating attitude | 1 Bad dreams | A | 148 | 0.16 | 0.0035 | 0.0489 | 57 | -0.26 | 0.0039 | 0.0523 |
|  | 2 Reliving events in mind | A | 147 | 0.06 | 0.3137 | 0.5903 | 57 | -0.20 | 0.0254 | 0.1630 |
|  | 3 Avoiding thoughts | A | 145 | 0.21 | 0.0002 | 0.0083 | 56 | -0.41 | <0.0001 | 0.0012 |
|  | 4 Avoiding physically | A | 145 | 0.18 | 0.0017 | 0.0312 | 56 | -0.17 | 0.0588 | 0.2517 |
|  | 5 Overly cautious | A | 145 | 0.15 | 0.0079 | 0.0826 | 56 | -0.20 | 0.0334 | 0.1848 |
|  | 6 Nervousness | A | 148 | 0.05 | 0.4117 | 0.6759 | 56 | -0.23 | 0.0119 | 0.1066 |
|  | 7 Calming difficulty | A | 146 | 0.07 | 0.2176 | 0.4906 | 56 | -0.17 | 0.0642 | 0.2648 |
|  | 8 Emotional numbness | A | 145 | 0.10 | 0.0686 | 0.2751 | 56 | -0.25 | 0.0063 | 0.0720 |
|  | 9 Sense of failure | A | 147 | 0.00 | 0.9918 | 1.0000 | 56 | -0.26 | 0.0041 | 0.0546 |
|  | 10 Self-doubt | A | 146 | 0.06 | 0.2692 | 0.5470 | 57 | -0.06 | 0.4847 | 0.7376 |
|  | 11 Disconnection to others | A | 147 | 0.08 | 0.1361 | 0.3919 | 56 | -0.19 | 0.0346 | 0.1870 |
|  | 12 Social difficulty | A | 147 | 0.19 | 0.0007 | 0.0184 | 56 | -0.21 | 0.0235 | 0.1573 |
| 18 Pain thoughts | 1 Bad dreams | A | 145 | -0.09 | 0.1013 | 0.3406 | 57 | -0.37 | <0.0001 | 0.0033 |
|  | 2 Reliving events in mind | A | 144 | -0.05 | 0.3977 | 0.6656 | 57 | -0.36 | 0.0001 | 0.0044 |
|  | 3 Avoiding thoughts | A | 142 | -0.05 | 0.3368 | 0.6131 | 56 | -0.39 | <0.0001 | 0.0020 |
|  | 4 Avoiding physically | A | 142 | 0.09 | 0.1221 | 0.3723 | 56 | -0.34 | 0.0003 | 0.0096 |
|  | 5 Overly cautious | A | 142 | -0.05 | 0.3376 | 0.6134 | 56 | -0.37 | <0.0001 | 0.0033 |
|  | 6 Nervousness | A | 145 | -0.01 | 0.9011 | 0.9613 | 56 | -0.28 | 0.0024 | 0.0392 |
|  | 7 Calming difficulty | A | 143 | 0.08 | 0.1429 | 0.3993 | 56 | -0.27 | 0.0038 | 0.0519 |
|  | 8 Emotional numbness | A | 142 | 0.03 | 0.5533 | 0.7815 | 56 | -0.14 | 0.1401 | 0.3963 |
|  | 9 Sense of failure | A | 144 | 0.02 | 0.6719 | 0.8546 | 56 | -0.19 | 0.0347 | 0.1870 |
|  | 10 Self-doubt | A | 144 | -0.08 | 0.1727 | 0.4398 | 57 | -0.12 | 0.1725 | 0.4398 |
|  | 11 Disconnection to others | A | 144 | 0.06 | 0.3037 | 0.5806 | 56 | -0.20 | 0.0284 | 0.1714 |
|  | 12 Social difficulty | A | 144 | -0.03 | 0.5402 | 0.7732 | 56 | -0.18 | 0.0478 | 0.2250 |
| 19 Loneliness feeling | 1 Bad dreams | D | 145 | 0.13 | 0.0250 | 0.1617 | 58 | -0.16 | 0.0676 | 0.2732 |
|  | 2 Reliving events in mind | D | 144 | -0.01 | 0.8806 | 0.9501 | 58 | -0.03 | 0.7593 | 0.8984 |
|  | 3 Avoiding thoughts | D | 142 | 0.14 | 0.0109 | 0.1020 | 57 | -0.19 | 0.0328 | 0.1824 |
|  | 4 Avoiding physically | D | 142 | 0.10 | 0.0669 | 0.2720 | 57 | -0.06 | 0.4889 | 0.7396 |
|  | 5 Overly cautious | D | 142 | 0.00 | 0.9912 | 1.0000 | 57 | -0.09 | 0.3078 | 0.5841 |
|  | 6 Nervousness | D | 145 | 0.01 | 0.8012 | 0.9195 | 57 | -0.20 | 0.0280 | 0.1697 |
|  | 7 Calming difficulty | D | 143 | 0.08 | 0.1512 | 0.4095 | 57 | -0.16 | 0.0759 | 0.2919 |
|  | 8 Emotional numbness | D | 142 | 0.05 | 0.3942 | 0.6621 | 57 | -0.15 | 0.1081 | 0.3523 |
|  | 9 Sense of failure | D | 145 | 0.00 | 0.9610 | 0.9896 | 57 | -0.21 | 0.0188 | 0.1369 |
|  | 10 Self-doubt | D | 143 | -0.02 | 0.7515 | 0.8937 | 58 | -0.05 | 0.5612 | 0.7860 |
|  | 11 Disconnection to others | D | 144 | 0.03 | 0.6441 | 0.8391 | 57 | -0.13 | 0.1464 | 0.4044 |
|  | 12 Social difficulty | D | 144 | 0.11 | 0.0510 | 0.2326 | 57 | -0.12 | 0.1743 | 0.4408 |
| 20 School fun | 1 Bad dreams | C | 142 | 0.15 | 0.0089 | 0.0893 | 58 | -0.20 | 0.0247 | 0.1602 |
|  | 2 Reliving events in mind | C | 141 | 0.01 | 0.9138 | 0.9671 | 58 | -0.17 | 0.0649 | 0.2664 |
|  | 3 Avoiding thoughts | C | 139 | 0.11 | 0.0533 | 0.2377 | 57 | -0.30 | 0.0010 | 0.0228 |
|  | 4 Avoiding physically | C | 139 | 0.09 | 0.1016 | 0.3412 | 57 | -0.09 | 0.3225 | 0.5992 |
|  | 5 Overly cautious | C | 139 | -0.08 | 0.1484 | 0.4061 | 57 | -0.17 | 0.0649 | 0.2664 |
|  | 6 Nervousness | C | 142 | -0.05 | 0.3751 | 0.6466 | 57 | -0.39 | <0.0001 | 0.0019 |
|  | 7 Calming difficulty | C | 140 | 0.07 | 0.2326 | 0.5088 | 57 | -0.35 | 0.0001 | 0.0055 |
|  | 8 Emotional numbness | C | 139 | 0.07 | 0.2146 | 0.4875 | 57 | -0.26 | 0.0049 | 0.0608 |
|  | 9 Sense of failure | C | 141 | 0.03 | 0.5726 | 0.7936 | 57 | -0.34 | 0.0002 | 0.0081 |
|  | 10 Self-doubt | C | 140 | -0.04 | 0.4357 | 0.6975 | 58 | 0.02 | 0.7908 | 0.9160 |
|  | 11 Disconnection to others | C | 141 | 0.07 | 0.2001 | 0.4715 | 57 | -0.30 | 0.0010 | 0.0225 |
|  | 12 Social difficulty | C | 141 | 0.08 | 0.1731 | 0.4404 | 57 | -0.33 | 0.0002 | 0.0090 |
| 21 Friends | 1 Bad dreams | D | 146 | 0.16 | 0.0034 | 0.0478 | 58 | -0.28 | 0.0023 | 0.0377 |
|  | 2 Reliving events in mind | D | 145 | -0.07 | 0.2203 | 0.4938 | 58 | -0.12 | 0.1958 | 0.4653 |
|  | 3 Avoiding thoughts | D | 143 | 0.17 | 0.0024 | 0.0392 | 57 | -0.21 | 0.0241 | 0.1594 |
|  | 4 Avoiding physically | D | 143 | 0.12 | 0.0307 | 0.1773 | 57 | -0.03 | 0.7652 | 0.9014 |
|  | 5 Overly cautious | D | 143 | -0.02 | 0.7800 | 0.9102 | 57 | -0.13 | 0.1653 | 0.4300 |
|  | 6 Nervousness | D | 146 | -0.04 | 0.4836 | 0.7375 | 57 | -0.19 | 0.0341 | 0.1862 |
|  | 7 Calming difficulty | D | 144 | -0.02 | 0.7031 | 0.8747 | 57 | -0.11 | 0.2392 | 0.5162 |
|  | 8 Emotional numbness | D | 143 | -0.02 | 0.7891 | 0.9157 | 57 | 0.03 | 0.7122 | 0.8786 |
|  | 9 Sense of failure | D | 145 | -0.02 | 0.7609 | 0.8994 | 57 | -0.07 | 0.4482 | 0.7069 |
|  | 10 Self-doubt | D | 144 | -0.06 | 0.2889 | 0.5637 | 58 | 0.18 | 0.0459 | 0.2197 |
|  | 11 Disconnection to others | D | 145 | 0.04 | 0.5197 | 0.7599 | 57 | -0.10 | 0.2691 | 0.5470 |
|  | 12 Social difficulty | D | 145 | 0.09 | 0.0956 | 0.3301 | 57 | -0.11 | 0.2071 | 0.4808 |
| 22 Dealing with school tasks | 1 Bad dreams | C | 143 | 0.14 | 0.0108 | 0.1018 | 57 | -0.01 | 0.8956 | 0.9593 |
|  | 2 Reliving events in mind | C | 142 | 0.05 | 0.3806 | 0.6515 | 57 | 0.06 | 0.4925 | 0.7423 |
|  | 3 Avoiding thoughts | C | 140 | 0.19 | 0.0011 | 0.0240 | 56 | 0.07 | 0.4147 | 0.6790 |
|  | 4 Avoiding physically | C | 140 | 0.26 | <0.0001 | 0.0011 | 56 | -0.03 | 0.7655 | 0.9014 |
|  | 5 Overly cautious | C | 140 | 0.03 | 0.6105 | 0.8209 | 56 | 0.00 | 0.9897 | 1.0000 |
|  | 6 Nervousness | C | 143 | -0.01 | 0.9251 | 0.9720 | 56 | 0.02 | 0.8495 | 0.9360 |
|  | 7 Calming difficulty | C | 141 | 0.15 | 0.0091 | 0.0904 | 56 | 0.00 | 0.9901 | 1.0000 |
|  | 8 Emotional numbness | C | 140 | 0.17 | 0.0029 | 0.0436 | 56 | 0.00 | 1.0000 | 1.0000 |
|  | 9 Sense of failure | C | 142 | 0.11 | 0.0567 | 0.2460 | 56 | 0.02 | 0.8442 | 0.9338 |
|  | 10 Self-doubt | C | 141 | 0.05 | 0.3828 | 0.6534 | 57 | -0.05 | 0.5824 | 0.8009 |
|  | 11 Disconnection to others | C | 142 | 0.10 | 0.0687 | 0.2751 | 56 | -0.09 | 0.3380 | 0.6136 |
|  | 12 Social difficulty | C | 142 | 0.16 | 0.0046 | 0.0582 | 56 | -0.15 | 0.1072 | 0.3506 |
| 23 Self-comparison to others | 1 Bad dreams | C | 148 | 0.04 | 0.4997 | 0.7458 | 58 | -0.08 | 0.3579 | 0.6319 |
|  | 2 Reliving events in mind | C | 147 | -0.09 | 0.0902 | 0.3212 | 58 | 0.05 | 0.6042 | 0.8171 |
|  | 3 Avoiding thoughts | C | 145 | 0.06 | 0.3251 | 0.6024 | 57 | -0.07 | 0.4258 | 0.6905 |
|  | 4 Avoiding physically | C | 145 | 0.02 | 0.7101 | 0.8778 | 57 | 0.00 | 0.9623 | 0.9901 |
|  | 5 Overly cautious | C | 145 | -0.03 | 0.5455 | 0.7767 | 57 | 0.05 | 0.5485 | 0.7773 |
|  | 6 Nervousness | C | 148 | -0.08 | 0.1569 | 0.4180 | 57 | -0.03 | 0.7734 | 0.9057 |
|  | 7 Calming difficulty | C | 146 | 0.00 | 0.9388 | 0.9764 | 57 | 0.02 | 0.8546 | 0.9390 |
|  | 8 Emotional numbness | C | 145 | -0.02 | 0.7565 | 0.8969 | 57 | -0.07 | 0.4563 | 0.7150 |
|  | 9 Sense of failure | C | 147 | -0.02 | 0.6799 | 0.8596 | 57 | -0.14 | 0.1318 | 0.3850 |
|  | 10 Self-doubt | C | 146 | -0.05 | 0.3847 | 0.6551 | 58 | -0.14 | 0.1208 | 0.3707 |
|  | 11 Disconnection to others | C | 147 | 0.04 | 0.5028 | 0.7466 | 57 | -0.20 | 0.0246 | 0.1602 |
|  | 12 Social difficulty | C | 147 | 0.13 | 0.0191 | 0.1375 | 57 | -0.18 | 0.0440 | 0.2156 |
| 24 Love-awareness | 1 Bad dreams | B | 149 | 0.09 | 0.1038 | 0.3446 | 58 | -0.22 | 0.0152 | 0.1222 |
|  | 2 Reliving events in mind | B | 148 | 0.04 | 0.4955 | 0.7436 | 58 | -0.15 | 0.0927 | 0.3246 |
|  | 3 Avoiding thoughts | B | 146 | 0.09 | 0.1251 | 0.3762 | 57 | -0.27 | 0.0035 | 0.0483 |
|  | 4 Avoiding physically | B | 146 | 0.16 | 0.0045 | 0.0570 | 57 | -0.14 | 0.1287 | 0.3814 |
|  | 5 Overly cautious | B | 146 | 0.05 | 0.3272 | 0.6038 | 57 | -0.11 | 0.2223 | 0.4961 |
|  | 6 Nervousness | B | 149 | -0.02 | 0.7709 | 0.9043 | 57 | -0.17 | 0.0624 | 0.2600 |
|  | 7 Calming difficulty | B | 147 | 0.07 | 0.1850 | 0.4535 | 57 | -0.16 | 0.0706 | 0.2799 |
|  | 8 Emotional numbness | B | 146 | 0.04 | 0.4284 | 0.6917 | 57 | -0.22 | 0.0167 | 0.1291 |
|  | 9 Sense of failure | B | 148 | 0.01 | 0.8573 | 0.9402 | 57 | -0.21 | 0.0229 | 0.1549 |
|  | 10 Self-doubt | B | 147 | 0.01 | 0.8997 | 0.9604 | 58 | -0.23 | 0.0116 | 0.1052 |
|  | 11 Disconnection to others | B | 148 | 0.06 | 0.2826 | 0.5585 | 57 | -0.14 | 0.1235 | 0.3736 |
|  | 12 Social difficulty | B | 148 | 0.08 | 0.1309 | 0.3834 | 57 | -0.13 | 0.1417 | 0.3983 |
| 25 Peer arguing | 1 Bad dreams | D | 149 | 0.04 | 0.4474 | 0.7064 | 58 | -0.13 | 0.1382 | 0.3945 |
|  | 2 Reliving events in mind | D | 148 | -0.05 | 0.3306 | 0.6074 | 58 | -0.12 | 0.1684 | 0.4353 |
|  | 3 Avoiding thoughts | D | 146 | 0.17 | 0.0028 | 0.0428 | 57 | -0.17 | 0.0578 | 0.2497 |
|  | 4 Avoiding physically | D | 146 | 0.05 | 0.3255 | 0.6025 | 57 | -0.01 | 0.9107 | 0.9661 |
|  | 5 Overly cautious | D | 146 | -0.01 | 0.8802 | 0.9501 | 57 | -0.14 | 0.1309 | 0.3834 |
|  | 6 Nervousness | D | 149 | -0.09 | 0.1152 | 0.3644 | 57 | -0.10 | 0.2909 | 0.5660 |
|  | 7 Calming difficulty | D | 147 | 0.09 | 0.1164 | 0.3656 | 57 | -0.10 | 0.2873 | 0.5622 |
|  | 8 Emotional numbness | D | 146 | -0.01 | 0.8406 | 0.9329 | 57 | -0.19 | 0.0331 | 0.1839 |
|  | 9 Sense of failure | D | 148 | 0.03 | 0.6358 | 0.8355 | 57 | -0.18 | 0.0467 | 0.2219 |
|  | 10 Self-doubt | D | 147 | -0.01 | 0.9065 | 0.9652 | 58 | -0.16 | 0.0755 | 0.2906 |
|  | 11 Disconnection to others | D | 148 | 0.02 | 0.6601 | 0.8484 | 57 | -0.07 | 0.4399 | 0.6998 |
|  | 12 Social difficulty | D | 148 | 0.13 | 0.0212 | 0.1473 | 57 | -0.13 | 0.1402 | 0.3963 |
| 26 Napping/dozing | 1 Bad dreams | A | 149 | 0.11 | 0.0455 | 0.2194 | 58 | -0.02 | 0.8234 | 0.9290 |
|  | 2 Reliving events in mind | A | 148 | 0.05 | 0.3807 | 0.6515 | 58 | -0.07 | 0.4542 | 0.7133 |
|  | 3 Avoiding thoughts | A | 146 | 0.11 | 0.0519 | 0.2354 | 57 | -0.10 | 0.2947 | 0.5703 |
|  | 4 Avoiding physically | A | 146 | 0.17 | 0.0018 | 0.0327 | 57 | 0.02 | 0.8001 | 0.9192 |
|  | 5 Overly cautious | A | 146 | 0.09 | 0.1033 | 0.3438 | 57 | 0.05 | 0.6198 | 0.8276 |
|  | 6 Nervousness | A | 149 | 0.06 | 0.2458 | 0.5243 | 57 | 0.01 | 0.8934 | 0.9577 |
|  | 7 Calming difficulty | A | 147 | 0.13 | 0.0188 | 0.1369 | 57 | -0.01 | 0.9108 | 0.9661 |
|  | 8 Emotional numbness | A | 146 | 0.11 | 0.0485 | 0.2256 | 57 | -0.12 | 0.1700 | 0.4367 |
|  | 9 Sense of failure | A | 148 | 0.01 | 0.8391 | 0.9329 | 57 | -0.04 | 0.6298 | 0.8331 |
|  | 10 Self-doubt | A | 147 | 0.04 | 0.4595 | 0.7175 | 58 | -0.02 | 0.8476 | 0.9350 |
|  | 11 Disconnection to others | A | 148 | 0.02 | 0.6665 | 0.8526 | 57 | 0.03 | 0.7475 | 0.8932 |
|  | 12 Social difficulty | A | 148 | 0.20 | 0.0003 | 0.0105 | 57 | -0.07 | 0.4374 | 0.6985 |
| 27 Eating problems | 1 Bad dreams | A | 147 | 0.05 | 0.3253 | 0.6025 | 57 | 0.16 | 0.0821 | 0.3029 |
|  | 2 Reliving events in mind | A | 147 | -0.03 | 0.6335 | 0.8355 | 57 | 0.31 | 0.0006 | 0.0155 |
|  | 3 Avoiding thoughts | A | 145 | 0.15 | 0.0087 | 0.0886 | 56 | 0.14 | 0.1164 | 0.3656 |
|  | 4 Avoiding physically | A | 145 | 0.14 | 0.0125 | 0.1090 | 56 | 0.24 | 0.0104 | 0.0993 |
|  | 5 Overly cautious | A | 145 | 0.00 | 0.9329 | 0.9751 | 56 | 0.25 | 0.0075 | 0.0804 |
|  | 6 Nervousness | A | 147 | -0.01 | 0.8779 | 0.9493 | 56 | 0.16 | 0.0861 | 0.3116 |
|  | 7 Calming difficulty | A | 145 | 0.11 | 0.0412 | 0.2088 | 56 | 0.16 | 0.0815 | 0.3012 |
|  | 8 Emotional numbness | A | 144 | 0.12 | 0.0393 | 0.2019 | 56 | 0.12 | 0.1891 | 0.4582 |
|  | 9 Sense of failure | A | 146 | 0.09 | 0.1191 | 0.3686 | 56 | -0.05 | 0.6209 | 0.8284 |
|  | 10 Self-doubt | A | 145 | 0.01 | 0.8694 | 0.9458 | 57 | 0.07 | 0.4179 | 0.6828 |
|  | 11 Disconnection to others | A | 146 | 0.08 | 0.1467 | 0.4044 | 56 | -0.01 | 0.9503 | 0.9833 |
|  | 12 Social difficulty | A | 146 | 0.20 | 0.0003 | 0.0106 | 56 | 0.10 | 0.2803 | 0.5563 |
| 28 Memorization | 1 Bad dreams | C | 150 | 0.02 | 0.7341 | 0.8884 | 58 | -0.20 | 0.0264 | 0.1643 |
|  | 2 Reliving events in mind | C | 149 | -0.03 | 0.6441 | 0.8391 | 58 | -0.15 | 0.0954 | 0.3301 |
|  | 3 Avoiding thoughts | C | 147 | 0.05 | 0.3508 | 0.6253 | 57 | -0.20 | 0.0256 | 0.1634 |
|  | 4 Avoiding physically | C | 147 | 0.07 | 0.2094 | 0.4842 | 57 | -0.07 | 0.4578 | 0.7166 |
|  | 5 Overly cautious | C | 147 | 0.00 | 0.9355 | 0.9757 | 57 | -0.15 | 0.0886 | 0.3170 |
|  | 6 Nervousness | C | 150 | -0.04 | 0.4670 | 0.7242 | 57 | -0.19 | 0.0397 | 0.2035 |
|  | 7 Calming difficulty | C | 148 | 0.12 | 0.0366 | 0.1939 | 57 | -0.13 | 0.1414 | 0.3975 |
|  | 8 Emotional numbness | C | 147 | 0.08 | 0.1660 | 0.4307 | 57 | -0.18 | 0.0441 | 0.2156 |
|  | 9 Sense of failure | C | 149 | 0.01 | 0.7973 | 0.9192 | 57 | -0.18 | 0.0477 | 0.2247 |
|  | 10 Self-doubt | C | 148 | -0.10 | 0.0795 | 0.2983 | 58 | -0.08 | 0.3646 | 0.6377 |
|  | 11 Disconnection to others | C | 149 | -0.03 | 0.5295 | 0.7684 | 57 | -0.23 | 0.0126 | 0.1092 |
|  | 12 Social difficulty | C | 149 | 0.06 | 0.3172 | 0.5928 | 57 | -0.31 | 0.0007 | 0.0175 |

*B-H* – Benjamini-Hochberg correction for multiple comparisons, *p*-values < 0.05 are indicated in red, N – number of participants, Tau – Tau correlation coefficient
